# Molecular Evolution of the Two-Component System BvgAS Involved in Virulence Regulation in *Bordetella*

**DOI:** 10.1371/journal.pone.0006996

**Published:** 2009-09-14

**Authors:** Julien Herrou, Anne-Sophie Debrie, Eve Willery, Geneviève Renaud-Mongénie, Camille Locht, Frits Mooi, Françoise Jacob-Dubuisson, Rudy Antoine

**Affiliations:** 1 Institut National de la Santé Et de la Recherche Médicale (INSERM-U629), Lille, France; 2 Institut Fédératif de Recherche (IFR142), Lille, France; 3 Institut Pasteur de Lille, Lille, France; 4 Sanofi-Pasteur, Marcy l'Etoile, France; 5 National Institute for Public Health and the Environment, Bilthoven, The Netherlands; Charité-Universitätsmedizin Berlin, Germany

## Abstract

The whooping cough agent *Bordetella pertussis* is closely related to *Bordetella bronchiseptica*, which is responsible for chronic respiratory infections in various mammals and is occasionally found in humans, and to *Bordetella parapertussis*, one lineage of which causes mild whooping cough in humans and the other ovine respiratory infections. All three species produce similar sets of virulence factors that are co-regulated by the two-component system BvgAS. We characterized the molecular diversity of BvgAS in *Bordetella* by sequencing the two genes from a large number of diverse isolates. The response regulator BvgA is virtually invariant, indicating strong functional constraints. In contrast, the multi-domain sensor kinase BvgS has evolved into two different types. The *pertussis* type is found in *B. pertussis* and in a lineage of essentially human-associated *B. bronchiseptica*, while the *bronchiseptica* type is associated with the majority of *B. bronchiseptica* and both ovine and human *B. parapertussis*. BvgS is monomorphic in *B. pertussis*, suggesting optimal adaptation or a recent population bottleneck. The degree of diversity of the bronchiseptica type BvgS is markedly different between domains, indicating distinct evolutionary pressures. Thus, absolute conservation of the putative solute-binding cavities of the two periplasmic Venus Fly Trap (VFT) domains suggests that common signals are perceived in all three species, while the external surfaces of these domains vary more extensively. Co-evolution of the surfaces of the two VFT domains in each type and domain swapping experiments indicate that signal transduction in the periplasmic region may be type-specific. The two distinct evolutionary solutions for BvgS confirm that *B. pertussis* has emerged from a specific *B. bronchiseptica* lineage. The invariant regions of BvgS point to essential parts for its molecular mechanism, while the variable regions may indicate adaptations to different lifestyles. The repertoire of BvgS sequences will pave the way for functional analyses of this prototypic system.

## Introduction

*Bordetella pertussis*, the whooping cough agent, is an extremely contagious pathogen that infects the upper respiratory tract of humans and causes an acute infection [1]. The expression of most virulence factors of this Gram-negative bacterium, including adhesins and toxins, is controlled by the BvgAS two-component system (TCS) [2]–[5]. TCSs regulate major physiological responses in bacteria [6]–[9]. They are composed of two proteins, a sensor kinase that perceives (a) signal(s) and autophosphorylates a conserved histidine residue, and a response regulator that becomes activated upon phosphorylation by its cognate sensor kinase and often serves as a transcriptional activator [7], [10], [11].

BvgS is an “unorthodox” sensor kinase composed of three domains potentially involved in signal perception: two tandem periplasmic Venus Fly Trap (VFT) domains, proteins composed of two lobes with a solute-binding cavity between them [12] and a cytoplasmic PAS (Per-ARNT-Sim) domain [13]. They are followed by several domains participating to a phosphorylation cascade: a histidine kinase (His-kinase) domain, an Asp-containing receiver domain and a His phosphotransfer domain (Hpt) [14]. When phosphorylated, the response regulator BvgA activates the transcription of the virulence-activated genes (*vags*) [15].

By controlling the intracellular concentration of phosphorylated BvgA, this system mediates a progressive transition between three phenotypic phases, Bvg^−^ (avirulent), Bvg^i^ (intermediate) and Bvg^+^ (virulent) [16]–[20]. The Bvg^+^ phase occurs at 37°C and is necessary for *B. pertussis* to cause respiratory infections in animal models [16], [21]. The Bvg^i^ phase, in which some adhesins are produced, has been proposed to play a role in transmission and in the initial stages of infection [22]. Under laboratory conditions, negative signals such as nicotinate, MgSO_4_ or low temperature trigger modulation to the Bvg^−^ phase [23]. A set of virulence-repressed genes (*vrg*s) is expressed in that phase [24].

*Bordetella parapertussis* and *Bordetella bronchiseptica* are close relatives of *B. pertussis* [25]–[28]. Two distinct lineages of *B. parapertussis* cause either a generally milder form of whooping cough in humans or ovine respiratory infections [29]–[33], and *B. bronchiseptica* causes chronic respiratory infections in various mammals and has also been occasionally isolated from humans [34], [35]. *B. pertussis* and *B. parapertussis* are thought to have derived from distinct *B. bronchiseptica* clones [28], [33], [36]. The virulence regulons of the three species are similar. They are also controlled by BvgAS and respond to the same negative modulators. *B. bronchiseptica* can survive outside its hosts in the Bvg^−^ phase, which promotes motility and survival under nutrient-limiting conditions [37]–[39]. In contrast, *B. pertussis* and *B. parapertussis_hu_* have no known reservoir other than humans, and the function of their Bvg^−^ phase is unclear. While *B. bronchiseptica* is fairly responsive to negative modulators, the sensitivity of *B. pertussis* to modulators appears to vary between isolates, which might suggest that the ability to down-modulate virulence has become dispensable for this species [16].

Because of the diversity of hosts and the different types of infection caused by these three *Bordetella* species, we investigated here the molecular diversity and evolution of BvgAS among the three *Bordetella* species. Sequencing of *bvgA* and *bvgS* from a number of isolates revealed that BvgA is almost invariant, while BvgS shows marked divergences between phylogenetic groups. Two evolutionary solutions for BvgS clearly appear from the analysis. The VFT2 domain is totally conserved within each of the two major BvgS types, indicating its pivotal role for the function of the protein.

## Results

### Genotyping of *bvgA and bvgS* in *Bordetella*

Full genomic sequences are available thus far for *B. pertussis* Tohama I, the *B. bronchiseptica* rabbit isolate RB50, and the human *B. parapertussis* isolate Bpp12822 [26]. The three genomes are closely related, with orthologous genes displaying little diversity between the three species. However, the *bvgS* genes markedly differ between them, while in contrast the *bvgA* genes are highly similar, harbouring small numbers of substitutions all of which are synonymous. To characterize the diversity of *bvgA and bvgS*, these genes were sequenced from a number of isolates selected from a collection described earlier [28]. In that previous phylogenetic study based on the sequences of housekeeping gene fragments, 4 major complexes of *Bordetella* were identified [28]. Complex I includes *B. bronchiseptica* isolates mainly of animal origin and the *B. parapertussis* ovine isolates (*Bpp_ov_*), complex II all *B. pertussis* isolates, complex III all *B. parapertussis* human isolates (*Bpp_hu_*) and complex IV *B. bronchiseptica* isolates mainly of human origin. We thus selected isolates to represent these 4 complexes and all the sequence types identified in that study. We attempted to maximize the diversity of hosts, geographic origins and times of isolation.

In the 82 *bvgS* sequences, 247 distinct single nucleotide polymorphisms (SNPs) on a total of 3,717 bp were found (6.6%). Among these, 147 SNPs are synonymous substitutions (3.8%) and 100 non-synonymous substitutions (2.6%). This apparent diversity masks major differences between the *Bordetella* complexes (Table 1), as well as between domains of the protein (see below). Thus, *bvgS* is remarkably conserved in *B. pertussis* (complex II) with only two -non silent- SNPs among all 29 isolates. This is illustrated by a very low diversity index (0.15), which is yet lower than that calculated from the set of housekeeping genes sequenced in [28] (0.65). In contrast, the genetic diversity indices of both *bvgS* and the housekeeping genes calculated for complex IV and for complex I are significantly higher than those of *B. pertussis* (Table 1). Although *B. parapertussis_hu_* is thought to have emerged more recently than *B. pertussis* [28], *bvgS* is more diverse in complex III than in complex II, while the contrary is true for the housekeeping genes (Table 1).

**Table 1:** Numerical analyses of the sequence data.

	*bvgA* (630 bp)	*bvgS* (3,717 bp)	HKG (2,912 bp)
Complexes	**I**	**II**	**III**	**IV**	Total	**I**	**II**	**III**	**IV**	Total	**I**	**II**	**III**	**IV**	Total
Number of isolates	38	28	6	13	85	35	29	6	12	82	35	22	6	12	75
Number of alleles	7	2	2	6	16	12	3	3	11	28	13	3	1	11	28
Total SNPs	6	1	2	6	15	68	2	20	41	247	51	2	0	20	83
Non synonymous SNPs	2	0	0	1	3	38	2	6	14	100	26	0	0	12	45
Synonymous SNPs	4	1	2	5	12	30	0	14	27	147	25	2	0	8	38
Shannon-Wiener index	1.18	0.15	0.63	1.52	2	1.72	0.29	0.86	2.37	2.42	2.34	0.65	0	2.37	2.87

The sequences of *bvgA* and *bvgS* originate from this study, while those of the 7 housekeeping gene fragments (HKG) were obtained from a previous study [28] and concatenated for the analyses. All three sets of sequences were obtained from similar sets of isolates. The Shannon-Wiener index represents the genetic diversity. Complex I includes *B. bronchiseptica* isolates mainly of animal origin and the *B. parapertussis* ovine isolates (*Bpp_ov_*), complex II all *B. pertussis* isolates, complex III all *B. parapertussis* human isolates (*Bpp_hu_*) and complex IV *B. bronchiseptica* isolates mainly of human origin.

*bvgA* was found to be almost invariant, with 15 SNPs in total (Table 1). Only three SNPs are non-synonymous, but each is found in only one isolate. This indicates an extremely strong pressure on *bvgA* in *Bordetellae*. Accordingly, genetic diversity in all 4 complexes is lower for *bvgA* than for housekeeping genes or *bvgS*. Both *bvgA* and *bvgS* are strikingly conserved in *B. pertussis* (complex II).

**Figure 1:**
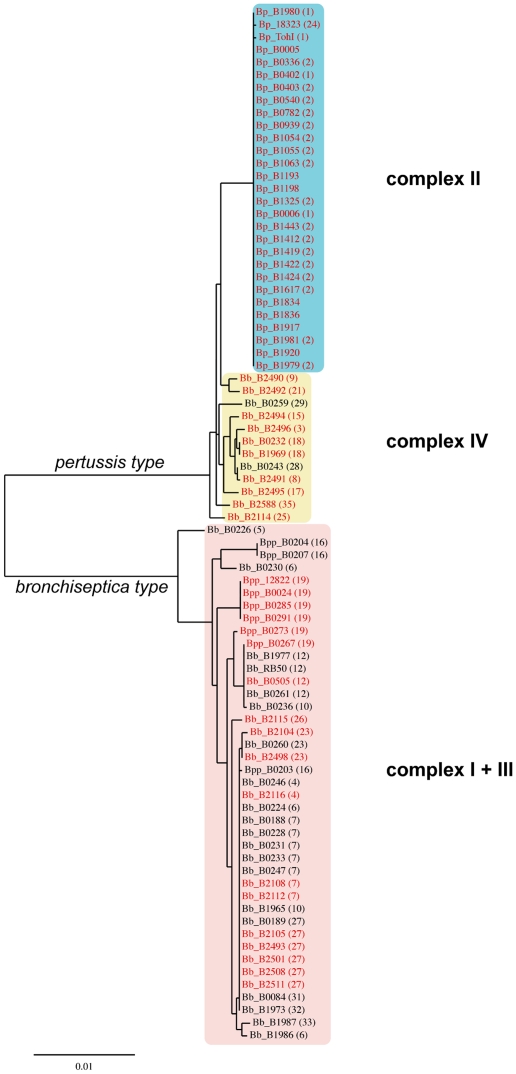
Phylogenetic tree based on the DNA sequences of *bvgS*. The lengths of the branches represent the phylogenetic distances. All human *B. pertussis* (Bp), *B. bronchiseptica* (Bb) and *B. parapertussis* (Bpp) isolates are shown in red. The major complexes identified in an earlier study [28] are boxed in colors, and the numbers in parentheses correspond to the sequence types defined in that study.

A phylogenetic tree was constructed based on the *bvgS* sequences (Fig. 1). This analysis clearly distinguishes two lineages of *bvgS*. The “*pertussis* type” (BvgS_Bp_) encompasses *bvgS* from the 29 *B. pertussis* isolates as well as from the 12 *B. bronchiseptica* complex IV isolates. The other type, referred to hereafter as the “*bronchiseptica* type” (BvgS_Bb_) encompasses *bvgS* from all 32 isolates that belong to the complex I of *B. bronchiseptica* and from the 9 isolates of *B. parapertussis*. Interestingly, the ovine and human *B. parapertussis* isolates do not form separate clusters but are included in the *B. bronchiseptica* complex I group, in contrast with previous studies [28], [40]. Similarly, the human isolates of *B. bronchiseptica* complex I are found interspersed among the animal isolates and do not form a separate group.

A comparison of the *bvgS*-based phylogeny with that based on concatenated fragments from 7 different housekeeping genes described earlier [28] showed that while the overall topology of the two trees are similar, details differ. In addition to the absence of a separate complex III encompassing the *B. parapertussis* human isolates in the *bvgS*-based tree, some of the sequence types (ST) defined based on the housekeeping genes are split between several different branches of the *bvgS*-based tree, e.g. ST6, ST16 and ST19 (Fig. 1). These observations suggests that *bvgS* has evolved somewhat differentially from the housekeeping genes in the various branches.

### Amino acid sequence analyses

**Figure 2:**
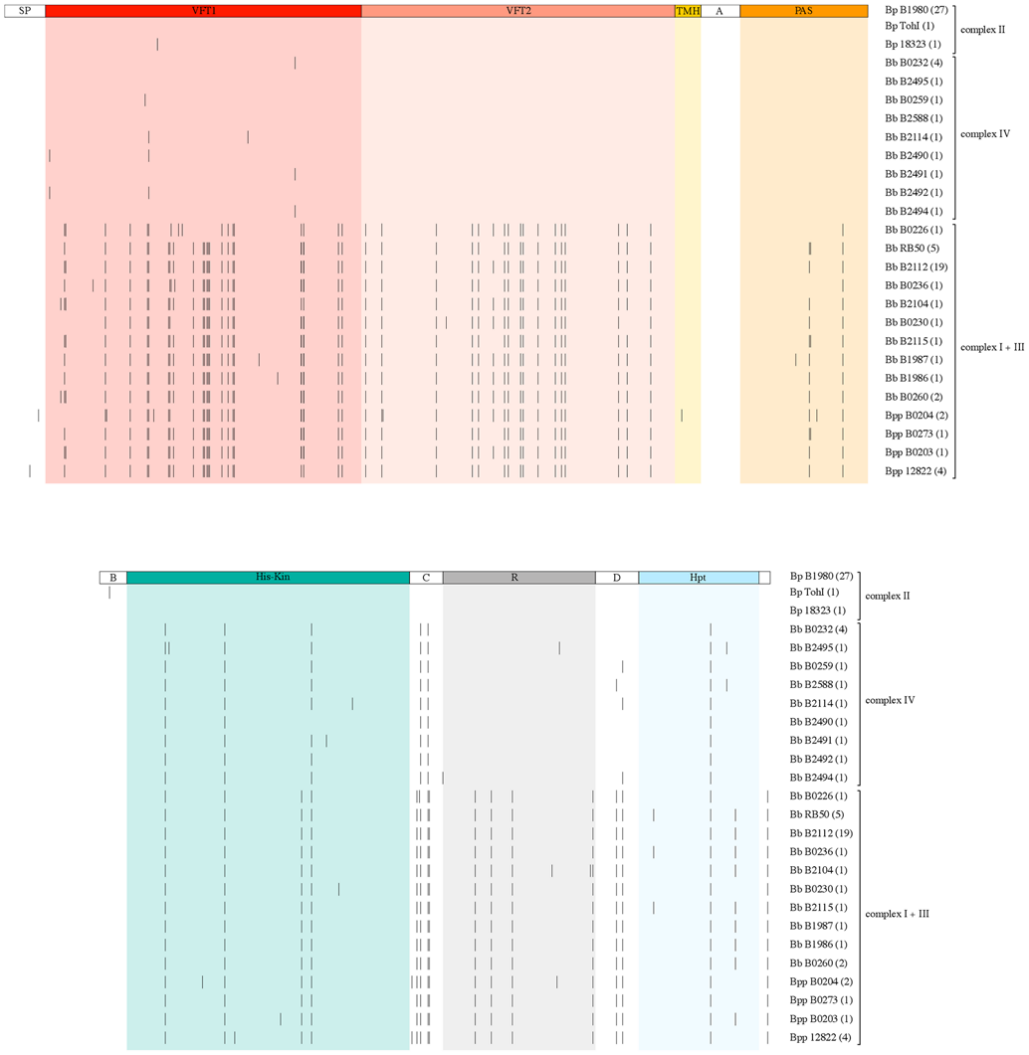
Schematic representation of BvgS variability at the amino acid level. The complexes I+III, II and IV are defined as in Fig. 1. The various domains of BvgS drawn to scale are boxed in colors. The top line represents the prototype BvgS of all *B. pertussis* isolates but Tohama I and 18323. Each line below represents a distinct BvgS sequence, with each substitution relative to the reference sequence shown as a small vertical bar at the corresponding position in the protein. The same substitution is systematically found at any given position. Some of the BvgS variants displayed represent several isolates, the number of which is given in parentheses. SP represents the signal peptide, TMH the transmembrane helix, R the receiver domain, and A, B, C and D the four linkers connecting the domains (see text).

To gain insight into the evolution of the functional domains of BvgS, the predicted amino acid sequences were aligned (Fig. 2). The two types of BvgS, BvgS_Bb_ and BvgS_Bp_, appear distinctly from this analysis. BvgS is identical among 27 *B. pertussis* isolates, while the two strains widely used in laboratories harbour each one substitution, E_705_K for Tohama I and I_124_T for strain 18323, respectively. BvgS of *B. bronchiseptica* complex IV is most similar to BvgS_Bp_ in the three domains potentially involved in signal perception, while the picture is slightly more blurred in the region corresponding to the phosphotransfer domains. BvgS from *B. parapertussis* resembles BvgS_Bb_ all along the sequence.

Remarkably, no more than two different residues are present at any given sequence position of the protein in all 82 isolates (Supporting information). Thus, in spite of the divergent evolution of BvgS between *B. bronchiseptica* complex I on the one hand, and *B. pertussis* and *B. bronchiseptica* complex IV on the other hand, selective pressure appears to limit the possible substitutions at the variable positions.

Our analyses revealed significant differences with respect to domain conservation. The first periplasmic domain, VFT1 totals the largest number of variable positions, although it is relatively well conserved within each type. Among the 82 isolates, 37 positions are variable in VFT1 over a total of 255 residues (14.5%). The overwhelming majority of the substitutions between the BvgS_Bb_ and the BvgS_Bp_ VFT1 are conservative in nature. In very rare instances, more drastic changes were observed, such as a Glu to Lys substitution at position 83 for two *B. parapertussis* ovine isolates and a Val to Glu substitution at position 206 for a single complex I isolate (Supporting information). Unlike for *B. pertussis*, some degree of variation is found within the complex IV VFT1s. Intriguingly, 4 of the substitutions identified in complex IV are not at the positions that differentiate BvgS_Bp_ from BvgS_Bb._ They are thus specific of complex IV.

One isolate, B0226, stands out from the *B. bronchiseptica* complex I by a small portion of VFT1. At 7 positions between residues 133 and 166 it shares *B. pertussis* sequences, although it also harbours unique substitutions in that region.

For VFT2 essentially two types of sequence have evolved. VFT2 of BvgS_Bp_ is invariant for all 41 *B. pertussis* and *B. bronchiseptica* complex IV isolates, suggesting that this domain is under strong stabilizing selection. VFT2 of BvgS_Bb_ also displays a very limited degree of variation. Furthermore, only conservative substitutions are found between the two types except for one at position 404, where Ala in BvgS_Bp_ is replaced by Asp in BvgS_Bb._ In total, 19 positions are different between the *pertussis* type and the *bronchiseptica* type VFT2s over a total of 254 residues (7.5%). There are no intermediates between the two VFT2 types. All these observations argue that VFT2 is pivotal to the function of BvgS and is under strong selective pressure in each lineage.

The PAS domain is well conserved among all isolates, suggesting that it is also under stabilizing pressure. In total only 5 positions are variable, and the few substitutions found in this domain are all conservative. Similar to VFT1 and VFT2, the PAS domain distinguishes *B. bronchiseptica* complex I from *B. pertussis* and *B. bronchiseptica* complex IV. Thus, for all three putative perception domains, *B. bronchiseptica* complex IV clearly sides with *B. pertussis*.

For the His-kinase domain, in contrast, *B. bronchiseptica* complex IV sequences appear to be more similar to BvgS_Bb_ than to BvgS_Bp_. Altogether the His-kinase domain is also much more conserved than the two VFT domains, with only 11 variable positions harbouring essentially conservative substitutions over a total of 223 residues (4.9%). This probably points to strong functional constraints on this domain.

The receiver domain is more diverse, with 9 variable positions over a total of 122 residues (7.3%). Intriguingly, the receiver domain of *B. bronchiseptica* complex IV is of the BvgS_Bp_ type, unlike the His-kinase domain.

The Hpt domain is well conserved, with only 4 variable positions for a total of 98 residues (4.1%). Intriguingly, at position 1190, the conservative Val to Ala substitution differentiates *B. pertussis* from *B*. *bronchiseptica* of both complexes I and IV. Altogether, thus, complex IV appears to be intermediate between the BvgS_Bb_ and the BvgS_Bp_ types for the His-kinase and Hpt domains, in contrast with the VFT1, VFT2, PAS and receiver domains. This suggests that the distinct domains have evolved at different rates.

Distinct functional constraints appear to operate also on the various linkers that join the domains. The transmembrane helix is invariant, except for a unique conservative Gly to Ala change. Similarly, the linkers A and B between the membrane and the PAS domain and between the PAS and His-kinase domains respectively are invariant, indicating that they are under strong selective pressure (Fig. 2). One striking exception to this rule is that a Glu residue is replaced by a Lys residue in Tohama I at position 705 (see below). In marked contrast with the strict conservation of linkers A and B, the linker C between the His-kinase and receiver domains seems to accumulate a disproportionate number of mutations, with 20% positions that are variable. Linker D between the receiver and Hpt domains harbours 2 variable positions over 36 residues.

### Mapping of the substitutions

**Figure 3:**
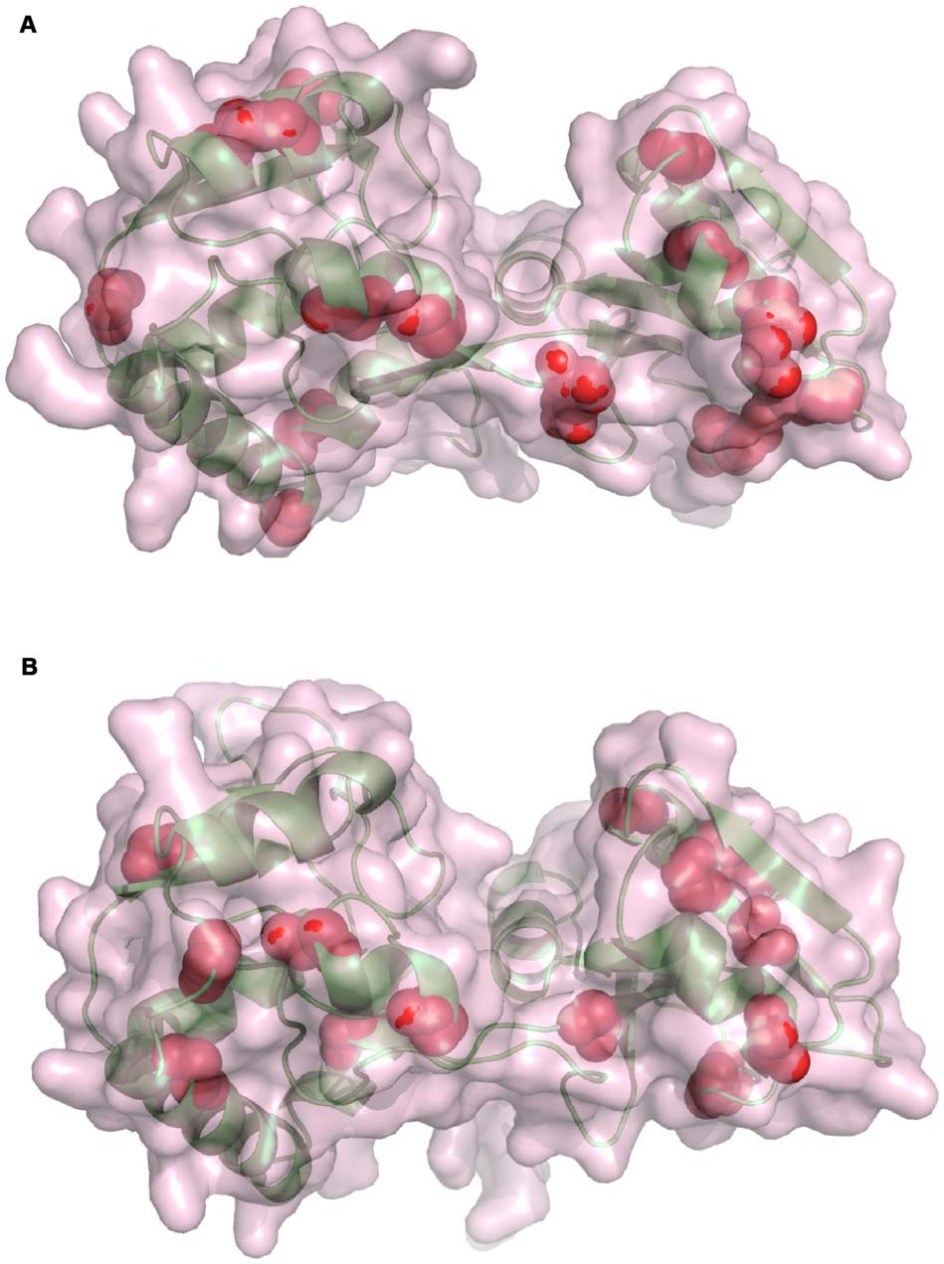
Structural models of VFT1 and VFT2. The models of VFT1 (A) and VFT2 (B) are based on the structure of the glutamine binding protein in an unliganded, open conformation (PDB 1GGG). The elements of secondary structure are shown in ribbon representation in lime green, and the surfaces of the proteins are shown in semi-transparency. The cavity of each of the two domains corresponds to the groove between the two lobes. The positions of the substitutions that distinguish the BvgS_Bp_ and BvgS_Bb_ types are depicted as red balls. Only the main chain atoms are shown. The rare substitutions have not been represented.

In order to map the positions of the substitutions, structural models were constructed for several domains of BvgS based on available X-ray structures of homologous proteins, since the crystal structure of BvgS is not available. VFT1 and VFT2 were modelled on the glutamine-binding protein, a bacterial periplasmic binding protein of an ABC transporter. The overwhelming majority of the substitutions between the two broad phylogenetic groups map to the external surfaces of both VFT1 and VFT2, leaving the potential solute-binding cavities conserved (Fig. 3). In addition, a few substitutions appear to be in the hydrophobic core of VFTs, but they are highly conservative and thus unlikely to affect the structure or stability of the proteins. Thus, the tertiary structures of the two VFT domains and their potential ligand-binding pockets are likely to be totally conserved, while the external surfaces of the proteins are quite different between the two BvgS types. The more drastic substitution between BvgS_Bp_ (Asp_404_) and BvgS_Bb_ (Ala_404_) is located in an external loop in the second lobe of VFT2. Unlike non-synonymous substitutions, synonymous substitutions are found both in the ligand binding cavities and on the surfaces of the VFTs, indicating differential selective pressure between cavities and surfaces.

A model of the His-kinase domain of BvgS was also constructed. The ATP-binding pocket appeared to be conserved among all isolates, as expected for the activity of the protein (not shown). A drastic substitution located in the linker B that precedes the His-kinase domain distinguishes *B. pertussis* Tohama I from all other *B. pertussis*, *B. bronchiseptica* and *B. parapertussis* isolates. Tohama I harbours a Lys at position 705 instead of a Glu in the 81 others. This linker is predicted to form an α helix immediately preceding the dimerisation helix of the His-kinase domain. Conceivably, this substitution might affect the conformation of this region most likely crucial for signal transduction (see below).

The structure of an Hpt domain in complex with its cognate phosphorylation substrate has been reported [41], [42], showing the interface between the two proteins. The BvgS Hpt domain has two interaction partners, the receiver domain and BvgA. The surfaces of Hpt predicted to form interfaces with its partners appear to be conserved as well (not shown). Similarly, neither of the three frequent substitutions between the receiver domains of the two BvgS types map to the predicted interface with Hpt (not shown).

Regarding BvgA, only 3 unique substitutions are found each in one isolate, namely Glu to Gly, Asp to Gly and Val to Glu at positions 64, 137 and 197, respectively. Sequence alignments of response regulators homologous to BvgA as well as the X-ray structure of one such protein indicate that these three substitutions are at non-conserved positions in the superfamily (not shown). The rarity and the positions of non-synonymous replacements in BvgA strongly indicate that the protein is under stringent selection.

### Functional implications of sequence variations

**Figure 4:**
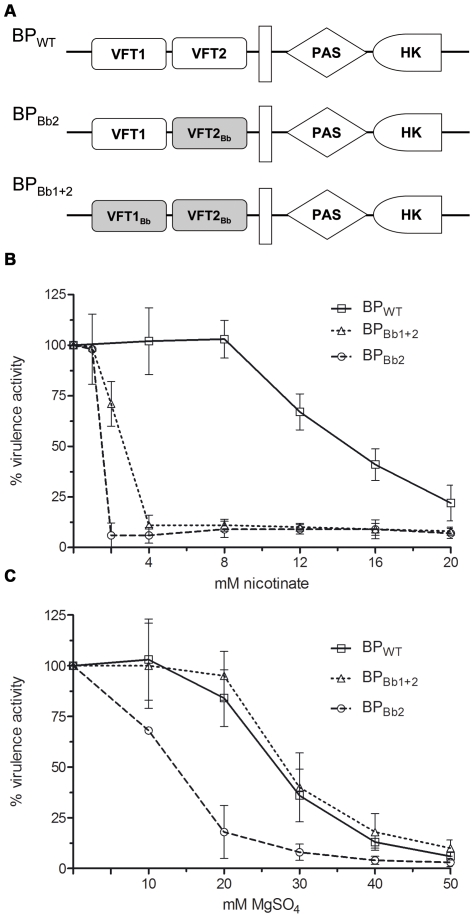
ß-galactosidase activities of the recombinant BPSM(*ptx::lacZ*) strains harbouring BvgS variants. (A) Schematic representation of the chimeric BvgS, with the white boxes representing BvgS_Bp_ domains and the shaded boxes BvgS_Bb_ domains. BP_WT_ corresponds to BPSM carrying the reporter gene. HK represents the His-kinase domain. (B) and (C) The ß-gal activities were measured as a function of increasing concentrations of nicotinate or MgSO_4_. The measurements were performed in triplicate using different clones for each recombinant strain. The level of ß-gal activity in non-modulated medium was taken as 100%.

Thus the vast majority of the predicted amino acid differences between *pertussis* type and *bronchiseptica* type BvgS are located in the periplasmic domains, essentially at the surface of the two VFT domains that appear to have co-evolved within each lineage. This suggests that the two domains operate together through inter-domain interactions, most likely for proper signal transduction. To test this hypothesis, we determined whether the sequence variations identified between the VFT1 and VFT2 of the two types of BvgS affect its activity or its level of sensitivity to the known negative virulence modulators. BvgS harbouring the BvgS_Bp_ periplasmic domain of our laboratory strain *B. pertussis* BPSM, a Tohama I derivative, was substituted with BvgS harbouring the BvgS_Bb_ periplasmic domain from *B. bronchiseptica* RB50, a typical complex I isolate (Fig. 4A). We also performed single domain exchanges. The activities of chimeric BvgS proteins and their responses to modulation were determined using the reporter gene *lacZ* placed under the control of the Bvg-regulated promoter of the pertussis toxin operon.

The replacement of both VFT1 and VFT2 of the BPSM BvgS by those of RB50 (BP_Bb1+2_) did not affect the level of BvgS activity in the absence of modulation, but it made the recombinant bacteria much more sensitive to nicotinic acid than their parent (Fig. 4B,C). Thus, the BvgS_Bb_ and BvgS_Bp_ periplasmic domains of BvgS influence the response to this negative modulator.

Similarly, the replacement of the BPSM VFT2 by that of RB50 (BP_Bb2_), yielding a VFT1_Bp_-VFT2_Bb_ chimera strongly enhanced the sensitivity of BvgS to both nicotinic acid and MgSO_4_ (Fig. 4B,C). In contrast, introduction of the RB50 VFT1 into the BPSM BvgS (BP_Bb1_) did not modify the response of the recombinant bacteria to either modulator (not shown). Thus, VFT2 is essential in determining sensitivity to modulation.

**Figure 5:**
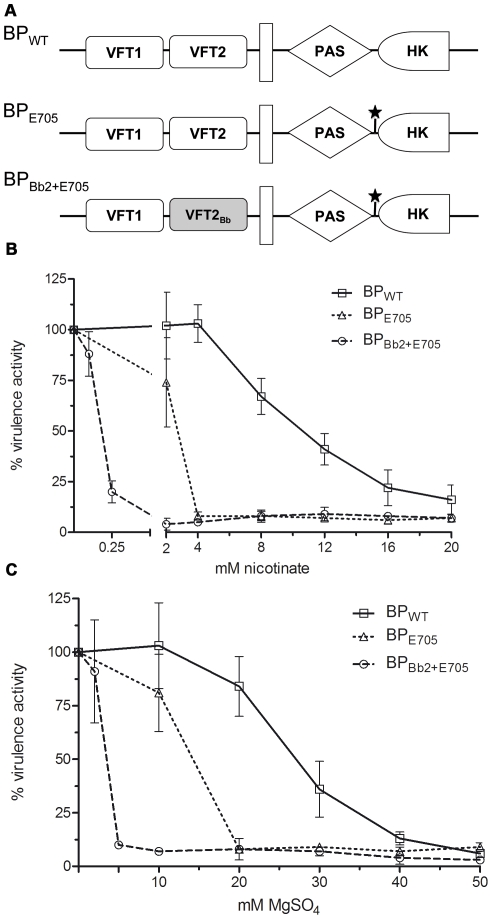
ß-galactosidase activities of the recombinant BPSM(*ptx::lacZ*) strains harbouring BvgS variants. (A) Schematic representation of the chimeric BvgS. The BvgS domains are represented as in Fig. 4. The star indicates that the Lys705 residue found in Tohama I was replaced by Glu as in all other isolates. (B) and (C) The ß-gal activities were measured as a function of increasing concentrations of nicotinate or MgSO_4_. The measurements were performed as in Fig. 4.

Because of its drastic character in an otherwise totally invariant linker segment, the substitution identified in the Tohama I BvgS was also investigated. Tohama I has been described as relatively insensitive to modulation compared with other *B. pertussis* isolates [16], and thus we tested whether Lys_705_ is the cause for this lack of sensitivity (Fig. 5A). Glu at position 705 in the BPSM BvgS made the recombinant strain more responsive to nicotinate than its parent, with 4 mM nicotinic acid causing the complete loss of BvgS activity (Fig. 5B). The BP_E705_ substitution also enhanced the responsiveness to MgSO_4_ (Fig. 5C). Thus, the presence of a Lys at position 705 is responsible for the lack of sensitivity to modulation of Tohama I. This substitution is quite rare in *Bordetella*, suggesting that the mutation acquired in Tohama I is not particularly advantageous. The other unique substitution identified in Bp18323, I_124_T, was also introduced into BPSM (BP_T124_). The level of ß-gal activity and the responsiveness to modulators of BP_T124_ was identical to that of BPSM, indicating that this is likely a neutral substitution (not shown).

Since both VFT2 and the linker preceding the HK domain determine the sensitivity to modulators, we tested whether their effects are cumulative. Therefore, a new chimeric strain, BP_Bb2+E705_ was constructed that harbours both the VFT2_Bb_ domain and Glu at position 705 (Fig. 5A). BP_Bb2+E705_ was hypersensitive to modulators, with as low as 0.25 mM nicotinic acid (Fig. 5B) or 5 mM MgSO_4_ (Fig. 5C) being sufficient for complete modulation.

### Hypermodulator *B. pertussis* in an animal model of infection

**Figure 6:**
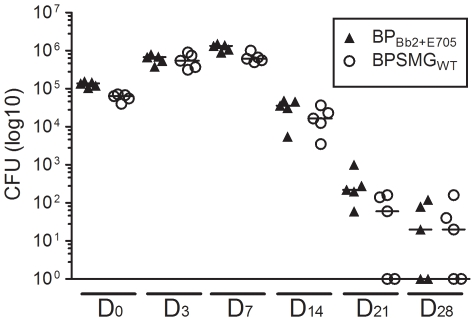
Colonization of mice lungs by two strains in a mixed infection. Balb/c mice were infected intranasally by 1.6×10^5^ CFU of each BPSMG_WT_ and BP_Bb2+E705_. At the indicated time points, mice were killed, and the viable bacteria present in the lungs were counted. 5 mice were analyzed per time point. The two bacterial strains were distinguished by an antibiotic marker. Medians for each group are shown.

No such hypersensitive variant has been characterized in natural isolates so far [16]. Thus, it was interesting to determine whether this phenotype might affect *B. pertussis*'s ability to colonize the respiratory tract in an animal model of infection. BPSM or BP_Bb2+E705_ were instilled intranasally to two groups of mice, and the numbers of bacteria recovered from the lungs were counted after various periods of time. No significant difference was observed between the two strains (not shown). To determine whether BP_Bb2+E705_ might be disadvantaged in a mixed infection, mice were also inoculated intranasally with equal numbers of the two bacterial strains together. The two strains colonized the lungs of mice in the same manner (Fig. 6). Thus, in this model of experimental infection, *B. pertussis* hypersensitive to modulation does not appear to be defective for colonization.

## Discussion

In this work, we investigated the molecular evolution of the BvgAS two-component system, which is central to the regulation of *Bordetella* virulence. BvgA is remarkably conserved. In contrast, the sequences of BvgS differ markedly between species, and in particular can be separated into two lineages of *Bordetella*. Interestingly, one of the two lineages includes all *B. pertussis* isolates and a subset of *B. bronchiseptica* isolates previously identified as forming a distinct *B. bronchiseptica* complex (called complex IV), mainly isolated from humans [28]. Our results confirm the hypothesis that complex IV strains are an evolutionary intermediate between animal and human adapted *Bordetellae* from which *B. pertussis* evolved [28]. The degree of divergence of BvgS varies significantly between lineages, as shown by the genetic diversity indices. In *B. pertussis*, BvgS displays an extremely low level of diversity, indicating that it has reached equilibrium in this highly successful human colonizer or has undergone a recent selective sweep. With a broad range of hosts, complex IV may be using a different evolutionary strategy, and its BvgS may still be in a process of diversification.

Our analyses do not provide evidence of an absolute correlation between the type of *bvgS* allele found in a given *Bordetella* lineage and the hosts of its members. Thus, two *bona fide* human pathogens, *B. pertussis* and *B. parapertussis_hu_,* harbour BvgS of the BvgS_Bp_ type and of the BvgS_Bb_ type, respectively, although the latter type otherwise corresponds to *B. bronchiseptica* complex I, with isolates of essentially animal origin. Similarly, the ovine and human isolates of *B. parapertussis* have the same BvgS type although they infect distinct hosts. It is interesting that BvgS is monomorphic in *B. pertussis* but not in *B. parapertussis_hu_,* as *B. pertussis* is assumed to be older [28]. This may reflect that *B. parapertussis_hu_* is not yet optimally adapted to its host, unlike *B. pertussis* [33].

Remarkably, the various domains of BvgS display widely different degrees of diversity, which shows that they are under different functional constraints, and/or parts of the protein are subject to diversifying selection in some lineages. The periplasmic domains of BvgS appear to have evolved most extensively. However, their evolution has been restricted to external surfaces, strongly arguing that the binding paradigm for Venus Fly Trap domains applies to BvgS. Purifying selective pressure on the cavities argues that they bind specific ligands. The prevalent model proposes that BvgS is in a permanently activated state in the absence of ligand, unlike classical sensors of TCS. If this is correct, then the conservation of the cavities is important essentially for the perception of negative signals. However, we have obtained preliminary data that specific substitutions in the VFTs cavities markedly decrease the activity of BvgS (our unpublished data). This supports the idea that the cavities of the VFT domains also perceive positive signals, at odds with the generally accepted model.

The external surfaces of the VFT domains have evolved considerably, arguing that they undergo a lesser selective pressure or are even diversifying. However, because there are no intermediate types between the BvgS_Bb_ and BvgS_Bp_ types, the evolutionary pressure must have selected for functional combinations of the two VFT surfaces. In other words, the surfaces may have co-evolved in each of the two major lineages because interactions between the two domains must be fine-tuned for optimal function. The combination of VFT1_Bp_ and VFT2_Bb_ in particular appears to significantly increase the sensitivity of BvgS to modulators. We thus propose that the sensitivity to modulators depend on the efficiency of signal transduction between the two VFT domains and between VFT2 and the cytoplasmic portion through the membrane segment. The membrane-proximal position of VFT2 most likely makes it especially important in this respect. Similarly, the two types of BvgS harbour distinct sets of cytoplasmic domains, although the situation is more blurred in the case of complex IV. This suggests that there may be small functional differences in the phosphorylation cascade between the two types.

The region of the protein with the lowest degree of diversity encompasses the transmembrane segment, the following cytoplasmic linker and the PAS and His-kinase domains, emphasizing the functional constraints on that region. The transmembrane segment must transmit signals perceived by the periplasmic domain to the cytoplasmic domain in a purely mechanical manner, such as a rotational or a piston-like motion. This signal must then be conveyed mechanically by the linker to the PAS domain, and then from the PAS domain to the His-kinase domain. PAS is thus central to transduction between periplasmic and His-kinase domains. In agreement with this model, several mutations that make BvgS insensitive to negative modulating signals map to the linker and PAS domains [43], [44]. Following phosphorylation of the His-kinase, signal transmission to BvgA is enzymatic, and thus functional constraints are exerted on the interfaces between domains, for interactions in trans and/or in cis, rather than on linkers [45]. In agreement with this idea, the linkers between the domains involved in the phosphorylation cascade have diverged more rapidly, most likely because they do not undergo strong selective pressures.

Only 3 rare substitutions were identified in BvgA. BvgA forms many interactions that are all important for its function. It forms homodimers, interacts with the Hpt domain for phosphotranfer and with its target DNA sequences and the RNA polymerase. This most likely explains why BvgA undergoes such a strong stabilizing pressure in pathogenic *Bordetellae*.

The importance of responsiveness to modulating agents for the function of BvgS *in vivo*, and the relevance of nicotinate and MgSO_4_ are unclear. The Bvg^−^ phase is most likely more important for *B. bronchiseptica* to survive outside the host and cause chronic infections [46], [47] than it is for the lifestyle of *B. pertussis*. Nevertheless, the fact that both types of BvgS have retained similar sensitivities to negative modulators argues that this phenotype is nevertheless relevant for both species. Thus, no natural *B. pertussis* isolates appear to be “locked” in the Bvg^+^ phase, even though such strains can arise by simple point mutations in the laboratory [43], [44], [48]. Therefore, the ability to down-modulate virulence factor expression must confer some advantage upon the bacterium, which is supported by the fact that Tohama I, with its low sensitivity to modulators, is the exception among *B. pertussis*. Conversely, we wondered whether *B. pertussis* with a BvgS variant hypersensitive to modulation would be impaired for colonisation. Surprisingly, this strain appeared to be functional in the mouse model of infection. The observation that no naturally occurring *Bordetella* isolate has been described to harbour such a hyper-responsive BvgS nevertheless suggests that this phenotype might be detrimental. The animal model used in this study does not reproduce all the features of a human infection, and in particular bacterial transmission. Better models may be needed to detect subtle regulation defects.

## Materials and Methods

### Ethics statement

All animal experiments were performed at the animal facility of the Institut Pasteur de Lille (number A59-35-064, Lille, France) according to the rules of the European Community Council guidelines (86/609/EEC) for laboratory animal experimentation. The animal protocol was approved by the local institutional review board (**C**omité d'**E**thique en **E**xpérimentation **A**nimale Nord-Pas-De-Calais, CEEA 03/2009).

### Sequencing of the circulating infectious *Bordetella* isolates

The *B. pertussis, B. bronchiseptica* and *B. parapertussis* isolates were selected from the collection described in [28]. *bvgS* was sequenced for 28 isolates of *B. pertussis,* 8 of *B. parapertussis* (5 *Bpp_hu_* and 3 *Bpp_ov_*), 31 of *B. bronchiseptica* from complex I and 12 of *B. bronchiseptica* from complex IV [28] (Table S2). *bvgA* was sequenced from 27 isolates of *B. pertussis*, 8 *B. parapertussis* isolates (5 *Bpp_hu_* and 3 *Bpp_ov_*), 34 *B. bronchiseptica* isolates from complex I and 13 from complex IV (Table S3). The strains were cultivated on BG blood agar medium during 16 hours for *B. bronchiseptica* and 48 hours for *B. pertussis* and *B. parapertussis*. Chromosomal DNA was extracted using the Illustra™ bacterial genomic Prep Mini Spin Kit (GE Healthcare) according to the manufacturer's instructions. For PCR amplification, several partially overlapping PCRs were performed by using several pairs of primers, bvgA′-Up and bvgA′-Lo, VFT1′-Up and VFT1′-Lo, VFT2′-Up and VFT2′-Lo, PAS/HisKin-Up and PAS/HisKin-Lo, R-Up and R-Lo and Hpt-Up and Hpt-Lo (Table S1). The following conditions for the mixes were used: HotStar Taq DNA polymerase in the presence of Q buffer and MgCl_2_ (Qiagen), 30 cycles of 1 min at 95°C, 1 min at 57°C and 1 min at 72°C. The PCR products were purified by using the PCR Purification Kit or the Gel Extraction Kit (Qiagen), depending on the degree of purity of the amplicons. The DNA fragments were sequenced by Genoscreen using an ABI 377 sequencer (Lille, France). The sequences were reassembled and compared by using the DNAstar software. A number of targets were amplified and sequenced twice to determine the level of error introduced by the PCR and sequencing steps. In particular, all fragments with a unique sequence type were checked in this manner. No discrepancy was obtained in any case.

### Sequence data analysis

The genetic diversity for each complex was calculated using the Shannon-Wiener index of diversity using the following formula in which *pi* is the frequency of the *i*th type:
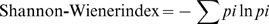



Single nucleotide polymorphisms (SNP) and their synonymous or non-synonymous characteristics were evaluated by using the DnaSP software. Neighbour-joining trees were constructed by using the www.phylogeny.fr software.

### Models of the BvgS domains

The PDB codes of the X-ray structures used to build models for the various domains of BvgS are 1GGG (VFTs), 2C2A (His-kinase), 2AYX (receiver domain) and 2AOB (Hpt domain), and 3C3W for BvgA. The Modeller software was used on the following portions of BvgS: residues 33–287 for VFT1, 288–541 for VFT2, 726–946 for the His-kinase domain, 974–1095 for the receiver domain and 1133–1228 for the Hpt domain.

### Construction of chimeric *B. pertussis* strains

*B. pertussis* BPSM is a Tohama I-derivative that is resistant to streptomycin [49]. The *bvgAS* deletion strain BPSM_Δ*bvgAS*_ was constructed as follows. The 5′ and 3′ extremities of the *bvgAS* locus were amplified by PCR using the BPSM chromosome as template and the oligonucleotides bvgA-Up and bvgA″-Lo, and bvgS-Up and bvgS-Lo as primers, respectively (Table S1). The amplicons were inserted directly into pCR®II-TOPO (Invitrogen) and sequenced. They were then successively introduced as *Eco*RI*-Kpn*I and *Xba*I*-Hin*dIII fragments into pUC19, yielding pUC19_Δ*bvgAS*_. The resulting 1.0 kb *Eco*RI*-Hin*dIII fragment was introduced into the *Eco*RI*-Hin*dIII sites of pSORTP1, a mobilizable plasmid used for conjugation [50]. Conjugation was performed on BG-blood agar plates containing 10 mg/ml MgCl_2_ for 6–7 hours, and co-integrates were selected on BG-blood agar plates containing 10 µg/ml gentamycin and 30 µg/ml nalidixic acid to prevent growth of the *E. coli* donor. Allelic exchange was selected by two successive steps as described [50]. After 4 to 5 days growth on selective media, isolated non-haemolytic streptomycin-resistant colonies, characteristic of the Bvg^−^ phase were analysed by PCR to confirm the deletion.

Recombinant BPSM strains containing chimeric *bvgS* genes were constructed as follows. Successive portions of the *bvgAS* locus of BPSM were amplified by PCR in order to introduce restriction sites using silent mutations at specific sites corresponding to junctions between structural domains. The sequences of the oligonucleotides used as primers are given in Table S1. In addition, an *Eco*RI restriction site naturally occurring in the region of the gene coding for the PAS/His-kinase portion of *bvgS* was removed by overlapping PCR using the pairs of primers ΔEcoRI-Up and PAS/HisKin-Lo, and ΔEcoRI-Lo and PAS/HisKin-Up. The various amplicons were sequenced and introduced successively into pUC19. The resulting “mosaic” *bvgAS* locus includes the 5′ and 3′ extremities of the operon remaining in BPSM_Δ*bvgAS*_ and necessary for allelic replacement in that strain. The mosaic *bvgAS* locus allowed us to replace selectively the genetic cassettes encoding each of the two VFT-like domains and the PAS/His-kinase domains of the BPSM *bvgS* by the corresponding cassettes of *B. pertussis* 18323 (to introduce Glu705) or *B. bronchiseptica* RB50. This procedure generated a number of *bvgS* variants that were each excised from pUC19 by restriction with *Eco*RI and *Hin*dIII and introduced into the *Eco*RI and *Hin*dIII sites of pSORTP1. Each *bvgS* variant was then introduced into BPSM_Δ*bvgAS*_ by allelic exchange, using conjugation as described above. Our criterion for the selection of recombinant clones was the restoration of hemolysis, which can be easily detected on BG-blood agar.

### Construction of *ptx-lacZ* transcriptional fusions and measurement of ß-galactosidase activity

A recombinant pFUS plasmid, harbouring *lacZ* in transcriptional fusion with the sequence of the first gene of the pertussis toxin operon [51], was introduced into the different strains by conjugation, and the integrants were selected on BG blood agar containing 100 µg/ml streptomycin and 10 µg/ml gentamycin. The recombinant strains were grown in modified Stainer-Scholte medium (SS) [52] containing the relevant antibiotics. After overnight growth at 37°C under rotating agitation, the bacterial suspension was used to initiate cultures in 10 ml of SS medium containing increasing concentrations of nicotinate or MgSO_4_. The inoculation volume was adapted to compensate for slower growth in the presence of high concentrations of nicotinate. The bacteria were grown until the cultures reached an OD_600_ of 1.5. They were harvested by centrifugation and broken by using a Hybaid Ribolyser apparatus (35 s at speed 6 in tubes containing 0.1 mm silica spheres as the lysing matrix). ß-galactosidase activities were measured as described [51]. The experiments were performed in triplicate.

### Murine respiratory tract infections

Experimental infections were performed at biosafety level 2 facilities with 8 week-old female BALB/c mice purchased from Charles River Laboratories.

Two groups of mice slightly sedated by intraperitoneal pentobarbital injection were inoculated with 1.6×10^6^ bacteria from BPSM and BP_Bb2+E705_ suspensions, by depositing 20 µl droplets into the nostrils. Groups of four animals per strain were sacrificed at days 0, 7, 14, 21 and 28 post-inoculation, and lungs were removed. Lung colonization was quantified by homogenizing the entire lungs in 2 to 5 ml phosphate-buffered saline (PBS), plating 100 µl aliquots of serial dilutions of the lung suspensions onto BG blood agar with 100 µg/ml of streptomycin, and counting the colonies after 4 days of incubation at 37°C.

Mixed infection essays were performed with BPSM carrying a gentamycin resistance marker at the 3′ end of the *bctCBA* operon [53] to distinguish between this strain (called hereafter BPSMG_WT_) and BP_Bb2+E705_. In this case, the inoculum consisted of 1.6×10^5^ CFU of each bacterial strain suspended in a final volume of 20 µl. Five animals per time point were sacrificed at days 0, 3, 7, 14, 21 and 28 post-inoculation and lung colonization was quantified as described above. Serial dilutions of each suspension were plated onto BG blood agar with 100 µg/ml of streptomycin or with 10 µg/ml of gentamycin. Colonies grown on gentamycin (corresponding to BPSMG_WT_) were subtracted from the number of colonies present on the streptomycin-containing medium (corresponding to both BPSMG_WT_ and BP_Bb2+E705_) to determine the number of BP_Bb2+E705_ bacteria present in the mice lungs.

## Supporting Information

Table S1 Oligonucleotides used in this study
(0.07 MB PDF)

Table S2 bvgS sequences
(0.35 MB RTF)

Table S3 bvgA sequences
(0.04 MB RTF)
