# Correction: Molecular Evolution of the Two-Component System BvgAS Involved in Virulence Regulation in *Bordetella*

**DOI:** 10.1371/annotation/ae7f86ff-0aca-4411-aed3-3eeea9303abb

**Published:** 2009-11-06

**Authors:** Julien Herrou, Anne-Sophie Debrie, Eve Willery, Geneviève Renauld-Mongénie, Camille Locht, Frits Mooi, Françoise Jacob-Dubuisson, Rudy Antoine

The fourth author's name was spelled incorrectly. The correct name is: Geneviève Renauld-Mongénie. The correct citation is: Herrou J, Debrie A-S, Willery E, Renauld-Mongénie G, Locht C, et al. (2009) Molecular Evolution of the Two-Component System BvgAS Involved in Virulence Regulation in Bordetella. PLoS ONE 4(9): e6996. doi:10.1371/journal.pone.0006996

