# Supplementary material for: Molecular Evolution of the Two-Component System BvgAS Involved in Virulence Regulation in Bordetella
Source: PLoS One. 2009 Sep 14;4(9):e6996. doi: 10.1371/journal.pone.0006996 (PMC2737282; doi:10.1371/journal.pone.0006996)
Supplement: Table S1 — Oligonucleotides used in this study (0.07 MB PDF) [file pone.0006996.s001.pdf]

Oligonucleotides	Sequences	Amplicons and comments
bvgS-Up<br>bvgS-Lo	5'- T**TCTAGA**CATTGCGAAGATCG -3'<br>5'- AT**AAGCTT**CATGGTCAGCCTCAGGTCC -3'	526 bp *Xba*I-*Hind*III *bvgS* fragment for construction of $BPSM_{\Delta bvgAS}$ and allelic exchange
bvgA-Up<br>bvgA''-Lo	5'-TA**GAATTC**AACGGCATTGACGGGCTCA-3'<br>5'-TA**GGTACC**CTAGTTGGACAACACGCTGATGA-3'	504 bp *EcoR*I-*Kpn*I *bvgA* fragment for construction of $BPSM_{\Delta bvgAS}$
bvgA-Lo	5'-AT**ACTAGT**GACGTGGCGTTCAGCTTCTG-3'	504 bp *EcoR*I-*Spe*I *bvgA* fragment for allelic exchange (used with bvgA-Up)
bvgA'-Up<br>bvgA'-Lo	5'-AGCCATTCCTTTGACGCATCA-3'<br>5'-CAGATCAGACTGCGGGGGTAC-3'	762 bp fragment coding for BvgA (sequencing)
VFT1-Up<br>VFT1-Lo	5'-AT**ACTAGT**GGAACTGATAGACCTCGCCAA-3'<br>5'-TA**GGATCC**CAGTCCCCAGCGGTAGATCA-3'	904 bp *Spe*I-*BamH*I fragment coding for VFT1 (cassette exchange)
VFT1'-Up<br>VFT1'-Lo	5'-GCAGAAGCTGAACGCCACGTC-3'<br>5'-ATTCAGGACCGCCACCTTGAC-3'	1020 bp fragment coding for VFT1 (sequencing)
VFT2-Up<br>VFT2-Lo	5'-TA**GGATCC**AGCATTTCGCTCGATTTCG-3'<br>5'-GGT**AGATCT**CGTTGCGGTAGG-3'	786 bp *BamH*I-*Bgl*II fragment coding for VFT2 (cassette exchange)
VFT2'-Up<br>VFT2'-Lo	5'-GCCTGCTGCGGGTGGTCA-3'<br>5'-TCGATGAGCACGCGCATGAAT-3'	1000 bp fragment coding for VFT2 (sequencing)
PAS/HisKin-Up<br>ΔEcoRI-Lo<br>ΔEcoRI-Up<br>PAS/HisKin-Lo	5'-ACCGCAACG**AGATCT**ACCTG-3'<br>5'-AAATTCCAGCTGGTCGTTC-3'<br>5'-GAACGACCAGCTGGAATTTATGCGCGTGCTCATCGACGG-3'<br>5'-TA**TCTAGA**ATGTCGCCTATCAGC-3'	708 bp *Bgl*II-*Xba*I fragment coding for the PAS and Histidine Kinase domains (overlapping PCR to remove EcoRI site from the cassette). PAS/HisKin-Up and Lo also used for sequencing
R-Up<br>R-Lo	5'-CCGGGCCAAGACCACGTTCCT-3'<br>5'-TGCACCGCGTACGTCCGTAAC-3'	1007 bp fragment coding for receiver domain of BvgS (sequencing)
Hpt-Up<br>Hpt-Lo	5'-ACTACCTGGGCCAGCGTGTC-3'<br>5'-CGGTATCTGCTGGGCGACCAT-3'	879 bp fragment coding for Hpt domain of BvgS (sequencing)
