# Supplementary material for: Molecular Evolution of the Two-Component System BvgAS Involved in Virulence Regulation in Bordetella
Source: PLoS One. 2009 Sep 14;4(9):e6996. doi: 10.1371/journal.pone.0006996 (PMC2737282; doi:10.1371/journal.pone.0006996)
Supplement: Table S2 — bvgS sequences (0.35 MB RTF) [file pone.0006996.s002.rtf]

>Bb_B0236.seq
ATGCCCGCCCCGCACCGCCTGTACCCCCGCAGTCTGATCTGCCTGGCTCAGGCGCTATTGGCATGGGCTTTGCTGGCATG
GGCGCCCGCGCAGGCAAGCCAGGAGCTGACCCTGGTCGGCAAGGCTGCCGTTCCCGACGTCGAGATCGCGCTCGACGGCG
ACGACTGGCGCTGGCTGGCGCGCAAGCGGGTGCTGACGCTGGGCGTGTACGCGCAGGACATTCCCCCGTTCGACGTCACC
TATGACGAGCGCTACGAAGGCCTGACGGCCGACTACATGGCGATCATCGCACACAACCTGGGCGTCCAGGCAAAAGTGCT
GCGCTACCCCACGCGCGAGCAAGCCGTCGGCGCACTGGAAAGCGGACAGATCGACCTCATCGGCACCGTCAATGGCATCG
AGGGCCGGCTGCAGAGCCTGCGCCTGAGTGTTCCCTACGCGGCCGACCACCCGGTGCTGGTCATGCCCATCGGCGCGCGC
CGCGCTCCGCCCGCGGACCTGGCAGGCCAGCGGTTGGCGGTTGACGCCAACTACTTGCCCAGGGAAACGCTGCAGCAGGC
CTATCCCCAGGCAACGCTGCATTACTTCCCATCGTCCGAACAGGCGCTGGCCGCGGTGGCCTATGGACAAGCCGACGTGT
TCATCGGCGATGCGCTGACCACCTCGCACCTCGTTTCGCAAAGCTACTTCAACGACGTTCGCGTCGTCGCCCCGGCCCAG
ATCGTGACGGGCGGGGAATCCTTCGGCGTGCGCGCCGACAATACCCGCCTGCTGCGGGTGGTCAATGCCGTACTCGAAGC
CATTCCGGCCTCCGAGCGCCGCAGCCTGATCTACCGCTGGGGCCTGGGCAGCAGCATTTCGCTCGATTTCGCGCGCCCCG
CCTATTCAGCGCGCGAGCAGCAGTGGATGGCAAACCATCCGGTCGTCAAGGTGGCGGTCCTGAACCTGTTCGCGCCCTTC
ACCCTGTTTCGCACCGATGAACAGTTCGGCGGCATCAGTGCCGCCGTGCTGCAACTGCTGCAGTTGCGCACCGGCCTGGA
TTTCCAGATCATCGGCGTCGACACGGTCGAGGAGCTGATCGCCAAGCTGCGCTCGGGCGAAGCCGACATGGCCGGCGCCC
TGTTCGTCAATGCCGCGCGGGAATCCGTCCTCAGCTTCAGCCGGCCGTATGTGCGCAATGGCATGGTGATCGTCACGCGC
CAGGACCCCGCCGCGCCCGCCGACGCCGATCACCTCGACGGCCGCACGATTGCGATGGTGCGCAACAGCGCCGCCATCCC
GCTCCTGCAGCAGCGCTATCCCCAGGCGAAGGTCGTGACCGCCGACAACCCGACCGAAGCCATGCTGCTGGTGGCCGATG
GCCAGGCCGACGCCGTCGTGCAGACGCAGATCAGCGCCAGCTACTACGTCAACCGCTACTTCGCCGGAAAACTGCGCATT
GCCTCGGCGCTGGACCTGCCGCCGGCCGAGATCGCGCTGGCGACGGCGCGCGGCCAGACCGAGCTGATATCCATCCTGAA
CAAGGCGCTCTACAGCATTTCGAACGACGAACTCGCCTCCATCGTCAGCCGCTGGCGCGGCAGCGACGGCGATCCGCGCA
CCTGGTACGCCTACCGCAACGAGATCTACCTGCTGATCGGGCTGGGCCTGTTGTCGGCCCTGCTGTTCCTGAGCTGGATC
GTCTACCTGCGGCGCCAGATCCGCCAGCGCAAGCGGGCCGAGCGGGCGCTGAACGACCAGCTGGAATTCATGCGCGTGCT
CATCGACGGCACGCCCAACCCCATCTATGTGCGCGATAAGGAAGGCCGCATGCTGTTGTGCAATGACGCCTACCTCGACA
CCTTTGGCGTGACTGCCGATGCGGTACTGGGCAAGACCATCCCGGAGGCCAACGTGGTGGGCGACCCGGCGCTGGCTCGC
GAGATGCACGAGTTCCTGCTCACGCGCATGTCCGCCGAGCGCGAGCCGCGCTTCGAGGACCGCGATGTCACGCTGCACGG
CCGCACCCGCCATGTCTACCAGTGGACGGTTCCGTACGGCGACTCGCTGGGCGAACTCAAGGGCATCATCGGCGGCTGGA
TCGACATTACCGAACGCGCCGAGCTGCTGCGCGAGCTGCACGACGCCAAGGAAAGCGCCGACGCCGCCAACCGGGCCAAG
ACCACGTTCCTGGCAACGATGAGCCACGAGATCCGCACGCCGATGAACGCGATCATCGGCATGCTGGAGCTGGCGCTGCT
CCGTCCGGCCGACCAGGAGCCGGACCGCCAGTCCATCCAGGTCGCGTACGACTCGGCCCGCAGCCTGCTGGAGCTGATAG
GCGACATCCTGGACATTGCGAAGATCGAGGCGGGAAAATTCGACCTGGCGCCGGTGCGCACGGCGCTGCGCGCCCTACCC
GAAGGGGCGATCCGCGTCTTCGACGGGTTGGCGCGCCAGAAAGGCATAGAGCTGGTATTGAAGACCGACATCGTGGGCGT
GGACGATGTATTGATAGACCCCTTGCGCATGAAGCAAGTGCTCTCGAACCTGGTGGGCAACGCCATCAAGTTCACCACCG
AAGGCCAGGTTGTCCTTACCGTGACCGCGCGCCCCGACGGCGAGGCCGCGCACGTGCAGTTCAGCGTGAGCGACACCGGC
TGCGGCATCAGCGAGGCCGACCAACGGCAGCTGTTCAAACCGTTCTCGCAGGTGGGCGGCAGCGCCGAGGCCGGGCCGGC
GCCGGGCACTGGCCTGGGCCTGTCCATCAGCCGCCGCCTCGTCGAATTGATGGGGGGAACGCTGGTCATGCGCAGCGCGC
CAGGGGTGGGCACAACGGTTTCGGTGGACCTGAGGCTGACCATGGTCGAAAAATCCGCGCAGGCCACGCCGCCCGCTGCG
GCCGCTCAGGCCACGCCATCCAAGCCGCAGGTATCGCTGCGCGTGCTGGTCGTCGATGACCACAAGCCCAACCTGATGCT
GCTGCGCCAGCAGCTGGACTACCTGGGCCAGCGTGTCGTCGCCGCCGACTCCGGCGAAGCCGCCCTGGCCCTGTGGCACG
AGCATGCGTTCGACGTCGTGATCACCGATTGCAACATGCCCGGTATCAACGGCTACGAATTGGCGCGCCGCATACGCGCC
GCCGAGGCCGCGCCCGGTTACGGACGTACGCGGTGCATTCTGTTCGGCTTCACGGCTTCGGCGCAGATGGACGAAGCGCA
GCGCTGCCGCGCCGCCGGCATGGACGACTGCCTGTTCAAGCCGATCGGCGTGGACGCCTTGCGGCAACGCTTGAACGAAG
CCGCGGCACGGGCCGCGCTCCCCACGCCCCCCTCGCCCCAGGCTGCCGCGCCGGCCACGCACGACGCCACCCCGGCGGCG
TTCTCGGCCGAGTCGATTCTTGCCCTGACGCAGAACGATGAGGCGCTCATCCGGCAATTGCTCGAAGAACTGATTCGCAC
CAACCGGGCGGACGTCGATCAATTGCAGAAGCTGCACCAGCAGGCCGATTGGCCGAAGGTCTCGGACATGGCGCACAGGC
TGGCCGGCGGCGCGCGCGTGGTCGATGCCAAGGCCATGATAGACACTGCGCTGGCGCTGGAAAAAAAAGCGCAAGGCCAG
GCTGGCCCCTCGCCCGAAATCGACGGCATGGTACGTACGCTTGCGGCGCAGTCCGCCGCGCTGGAGACGCAACTACGCGC
CTGGCTGGAGCAACGGCCGCATCAAGGCCAGCCCTGA
>Bb_B0247.seq
ATGCCCGCCCCGCACCGCCTGTACCCCCGCAGTCTGATCTGCCTGGCTCAGGCGCTATTGGCATGGGCTTTGCTGGCATG
GGCGCCCGCGCAGGCAAGCCAGGAGCTGACCCTGGTCGGCAAGGCTGCCGTTCCCGACGTCGAGATCACGCTCGACGGCG
ACGACTGGCGCTGGCTGGCGCGCAAGCGGGTGCTGACGCTGGGCGTGTACGCGCCGGACATTCCCCCGTTCGACGTCACC
TATGACGAGCGCTACGAAGGCCTGACGGCCGACTACATGGCGATCATCGCGCACAACCTGGGCGTCCAGGCAAAAGTGCT
GCGCTACCCCACGCGCGAGCAAGCCGTCGGCGCACTGGAAAGCGGACAGATCGACCTCATCGGCACCGTCAATGGCATCG
AGGGCCGGCTGCAGAGCCTGCGCCTGAGTGTTCCCTACGCGGCCGACCACCCGGTGCTGGTCATGCCCATCGGCGCGCGC
CGCGCTCCGCCCGCGGACCTGGCAGGCCAGCGGTTGGCGGTTGACGCCAACTACTTGCCCAGGGAAACGCTGCAGCAGGC
CTATCCCCAGGCAACGCTGCATTACTTCCCATCATCCGAACAGGCGCTGGCCGCGGTGGCCTATGGACAAGCCGACGTGT
TCATCGGCGATGCGCTGACCACCTCGCACCTCGTTTCGCAAAGCTACTTCAACGACGTTCGCGTCGTCGCCCCGGCCCAG
ATCGTGACGGGCGGGGAATCCTTCGGCGTGCGCGCCGACAATACCCGCCTGCTGCGGGTGGTCAATGCCGTGCTCGAAGC
CATTCCGGCCTcCGAGCGCCGCAGCCTGATCTACCGCTGGGGCCTGGGCAGCAGCATTTCGCTCGATTTCGCGCGCCCCG
CCTATTCAGCGCGCGAGCAGCAGTGGATGGCAAACCATCCGGTCGTCAAGGTGGCGGTCCTGAACCTGTTCGCGCCCTTC
ACCCTGTTTCGCACCGATGAACAGTTCGGCGGCATCAGTGCCGCCGTGCTGCAACTGCTGCAGTTGCGCACCGGCCTGGA
TTTCCAGATCATCGGCGTCGACACGGTCGAGGAGCTGATCGCCAAGCTGCGCTCGGGCGAAGCCGACATGGCCGGCGCCC
TGTTCGTCAATGCCGCGCGGGAATCCGTCCTCAGCTTCAGCCGGCCGTATGTGCGCAATGGTCTGGTGATCGTCACGCGC
CAGGACCCCGCCGCGCCCGCCGACGCCGATCACCTCGACGGCCGCACGATTGCGATGGTGCGCAACAGCGCCGCCATCCC
GCTCCTGCAGCAGCGCTATCCCCAGGCGAAGGTCGTGACCGCCGACAACCCGACCGAAGCCATGCTGCTGGTGGCCGATG
GCCAGGCCGACGCCGTCGTGCAGACGCAGATCAGCGCCAGCTACTACGTCAACCGCTACTTCGCCGGAAAACTGCGCATT
GCCTCGGCGCTGGACCTGCCGCCGGCCGAGATCGCGCTGGCGACGGCGCGCGGCCAGACCGAGCTGATATCCATCCTGAA
CAAGGCGCTCTACAGCATTTCGAACGACGAACTCGCCTCCATCGTCAGCCGCTGGCGCGGCAGCGACGGCGATCCGCGCA
CCTGGTACGCCTACCGCAACGAAATCTACCTGCTGATCGGGCTGGGCCTGTTGTCGGCCCTGCTGTTCCTGAGCTGGATC
GTCTACCTGCGGCGCCAGATCCGCCAGCGCAAGCGGGCCGAGCGGGCGCTGAACGACCAGCTGGAATTCATGCGCGTGCT
CATCGACGGCACGCCCAACCCCATCTATGTGCGCGATAAGGAAGGCCGCATGCTGTTGTGCAATGACGCCTACCTCGACA
CCTTTGGCGTGACTGCCGATGCGGTACTGGGCAAGACCATCCCGGAGGCCAACGTGGTGGGCGACCCGGCGCTGGCTCGC
GAGATGCACGAGTTCCTGCTCACGCGCATGGCCGCCGAGCGCGAGCCGCGCTTCGAGGACCGCGATGTCACGCTGCACGG
CCGCACCCGCCATGTCTACCAGTGGACGGTTCCGTACGGCGACTCGCTGGGCGAACTCAAGGGCATCATCGGCGGCTGGA
TCGACATTACCGAACGCGCCGAGCTGCTGCGCGAGCTGCACGACGCCAAGGAAAGCGCCGACGCCGCCAACCGGGCCAAG
ACCACGTTCCTGGCAACGATGAGCCACGAGATCCGCACGCCGATGAACGCGATCATCGGCATGCTGGAGCTGGCGCTGCT
CCGTCCGGCCGACCAGGAGCCGGACCGCCAGTCCATCCAGGTCGCGTACGACTCGGCCCGCAGCCTGCTGGAGCTGATAG
GCGACATCCTGGACATTGCGAAGATCGAGGCGGGAAAATTCGACCTGGCGCCGGTGCGCACGGCGCTGCGCGCCCTGCCC
GAAGGGGCGATCCGCGTCTTCGACGGGTTGGCGCGCCAGAAAGGCATAGAGCTGGTATTGAAGACCGACATCGTGGGCGT
GGACGATGTATTGATAGACCCCTTGCGCATGAAGCAAGTGCTCTCGAACCTGGTGGGCAACGCCATCAAGTTCACCACCG
AAGGCCAGGTTGTCCTTACCGTGACCGCGCGCCCCGACGGCGAGGCCGCGCACGTGCAGTTCAGCGTGAGCGACACCGGC
TGCGGCATCAGCGAGGCCGACCAACGGCAGCTGTTCAAACCGTTCTCGCAGGTGGGCGGCAGCGCCGAGGCCGGGCCGGC
GCCGGGCACTGGCCTGGGCCTGTCCATCAGCCGCCGCCTCGTCGAATTGATGGGGGGAACGCTGGTCATGCGCAGCGCGC
CAGGGGTGGGCACAACGGTTTCGGTGGACCTGAGGCTGACCATGGTCGAAAAATCCGCGCAGGCCACGCCGCCCGCTGCG
GCCGCTCAGGCCACGCCATCCAAGCCGCAGGTATCGCTGCGCGTGCTGGTCGTCGATGACCACAAGCCCAACCTGATGCT
GCTGCGCCAGCAGCTGGACTACCTGGGCCAGCGTGTCGTCGCCGCCGACTCCGGCGAAGCCGCCCTGGCCCTGTGGCACG
AGCATGCGTTCGACGTCGTGATCACCGATTGCAACATGCCCGGTATCAACGGCTACGAATTGGCGCGCCGCATACGCGCC
GCCGAGGCCGCGCCCGGTTACGGACGTACGCGGTGCATTCTGTTCGGCTTCACGGCTTCGGCGCAGATGGACGAAGCGCA
GCGCTGCCGCGCCGCCGGCATGGACGACTGCCTGTTCAAGCCGATCGGCGTGGACGCCTTGCGGCAACGCTTGAACGAAG
CCGCGGCACGGGCCGCGCTCCCCACGCCCCCCTCGCCCCAGGCTGCCGCGCCGGCCACGCACGACGCCACCCCGGCGGCG
TTCTCGGCCGAGTCGATTCTTGCCCTGACGCAGAACGATGAGGCGCTGATCCGGCAATTGCTCGAAGAAGTGATTCGCAC
CAACCGGGCGGACGTCGATCAATTGCAGAAGCTGCACCAGCAGGCCGATTGGCCGAAGGTCTCGGACATGGCGCACAGGC
TGGCCGGCGGCGCGCGCGTGGTCGATGCCAAGGCCATGATAGACACTGCGCTGGCGCTGGAGAAAAAAGCGCAAGGCCAG
GCTGGCCCCTCGCCCGAAATCGACGGCATGGTACGTACGCTTGCGGCGCAGTCCGCCGCGCTGGAGACGCAACTACGCGC
CTGGCTGGAGCAACGGCCGCATCAAGGCCAGCCCTGA
>Bb_B0259.seq
ATGCCCGCCCCGCACCGCCtgTACCCCCGCAGTCTGATCTGCCTGGCTCAGGCGCTTTTGGCATGGGCTTTGCTGGCATG
GGCGCCCGCGCAGGCAAGCCAGGAGCTGACCCTGGTCGGCAAGGCTGCCGTTCCCGACGTCGAGGTCGCGCTCGACGGCG
ACGACTGGCGTTGGCTGGCCCGCAAGCGGGTACTGACGCTGGGTGTGTACGCACCGGACATTCCTCCGTTCGACGTCACC
TATGGCGAACGCTACGAAGGCCTGACGGCCGACTACATGGCGATCATCGCGCACAACCTGGGGATGCAGGCGAAAGTGCT
GCGATACCCCACGCGCGAACGAGCCCTCAGCGCGCTGGAAAGCGGGCAGATCGACCTCATCGGCACCGTCAATGGCACCG
ACGGCCGGCAACAGAGTCTGCGTCTGAGCGTTCCCTACGCCGCCGACCACCCGGTGATCGTCATGCCTATCGGCGCACGC
CACGTTCCAGCCTCGAACCTGGCCGGCCAGCGGCTGGCGGTCGACATCAACTACCTGCCCAAGGAAACGCTCGCACGGGC
CTACCCGCAGGCGACGCTGCATTACTTTCCCTCATCCGAGCAGGCGCTGGCCGCGGTGGCCTATGGGCAGGCCGACGTAT
TCATCGGCGATGCCCTGACCACCTCGCACCTCGTATCGCAAAGCTATTTCAATGACGTTCGCGTAGTCGCCCCGGCCCAT
ATCGCGACGGGCGGAGAATCCTTCGGCGTGCGCGCCGACAACACCCGCCTGCTGCGGGTGGTCAACGCCGTACTCGAAGC
CATTCCGCCTTCCGAACACCGCAGCCTGATCTACCGCTGGGGACTGGGCAGCAGCATTTCGCTCGATTTCGCGCACCCCG
CGTATTCCGCGCGCGAGCAGCAATGGATGGCAGACCACCCCGTCGTCAAGGTGGCGGTCCTGAATCTGTTCGCGCCCTTC
ACCCTGTTCCGCACCGACGAACAGTTCGGCGGGATCAGCGCCGCCGTGCTGCAGCTGCTGCAATTGCGCACCGGCCTGGA
CTTCGAGATCATCGGCGTCGACACGGTCGAGGAACTGATAGCCAAGCTGCGTTCGGGCGAAGCCGACATGGCCGGCGCCC
TGTTCGTCAACAGCGCGCGGGAGTCCTTCCTCAGTTTCAGCCGGCCGTATGTGCGCAATGGCATGGTGATCGTCACGCGC
CAGGACCCCGACGCGCCCGTCGACGCCGATCATCTGGACGGCCGCACGGTCGCGTTGGTGCGCAACAGCGCCGCCATTCC
TCTGCTGCAGCGGCGCTACCCCCAGGCGAAGGTGGTGACCGCCGACAACCCGAGCGAGGCGATGCTGATGGTGGCCAATG
GACAGGCCGACGCCGTCGTGCAGACGCAGATCAGCGCCAGCTATTACGTCAACCGCTACTTCGCCGGCAAGCTGCGCATC
GCCTCGGCGCTGGACCTGCCCCCGGCCGAGATCGCGCTGGCGACGACGCGCGGCCAGACCGAACTGATGTCCATCCTGAA
CAAGGCGCTCTACAGCATTTCGAACGACGAGCTCGCCTCCATCATCAGCCGCTGGCGCGGCAGCGACGGCGATCCGCGCA
CCTGGTACGCCTACCGCAACGAGATCTACCTGCTGATCGGGCTGGGCCTGTTGTCGGCCCTGCTGTTCCTGAGCTGGATC
GTCTACCTGCGGCGCCaGATCCGCCAGCGCAAGCGGGCCGAGCGGGCGCTGAACGACCAGCTGGAATTCATGCGCGTGCT
CATCGACGGCACGCCCAACCCCATCTATGTGCGCGATAAGGAAGGCCGCATGCTGTTGTGCAATGACGCCTACCTCGACA
CCTTTGGCGTGACTGCCGATGCGGTACTGGGCAAGACCATTCCGGAAGCCAACGTGGTGGGCGACCCGGCGCTGGCCCGC
GAAATGCACGAGTTCCTGCTCACGCGCGTGGCCGCCGAGCGCGAGCCGCGCTTCGAGGACCGCGATGTCACGCTGCACGG
CCGCACCCGCCATGTCTACCAGTGGACGATTCCGTACGGCGACTCGCTGGGCGAACTCAAGGGCATCATCGGCGGCTGGA
TCGACATCACCGAACGCGCCGAGCTGCTGCGCGAGCTGCACGACGCCAAGGAAAGCGCCGACGCCGCCAACCGGGCCAAG
ACCACGTTCCTGGCAACGATGAGCCACGAGATCCGCACGCCGATGaACGCGATCATCGGCATGCTGGAGCTGGCGCTGCT
CCGTCCGGCCGACCAGGAGCCGGATCGCCAGTCCATCCAGGTCGCGTACGACTCGGCCCGCAGCCTGCTGGAGCTGATAG
GCGACATCCTCGACATTGCGAAGATCGAGGCGGGAAAATTCGACCTGGCGCCGGTGCGCACGGCGCTGCGCGCCCTGCCC
GAAGGGGCGATCCGCGTCTTCGACGGATTGGCGCGCCAAAAAGGCATAGAGCTGGTATTGAAGACCGACATCGTGGGCGT
CGACGATGTATTGATAGACCCCTTGCGCATGAAGCAAGTGCTCTCGAACCTGGTGGGTAACGCCATCAAGTTCACCACCG
AAGGCCAGGTTGTCCTTGCCGTGACCGCACGCCCCGACGGCGAGGCCGCGCACGTGCAGTTCAGCGTGAGCGACACCGGC
TGCGGCATCAGCGAGGCCGACCAACGGCAGCTGTTCAAACCGTTCTCGCAGGTGGGTGGTAGCGCCGAGGCCGGGCCGGC
GCCGGGCACCGGCCTGGGCCTGTCCATCAGCCGCCGCCTCGTCGAATTGATGGGGGGAACGCTGGTCATGCGCAGCGCGC
CAGGGGTGGGCACAACGGTTtCGGTGGACCTGAGGCTGACCATGGTCGAAAAATCCGTGCAGGCCACGCCGCCCGCTGCG
GCCGCTGCGGCCACGCCGTCCAAGCCGCAGGTATCGCTGCGCGTGCTGGTCGTCGATGACCACAAACCCAACCTGATGCT
GCTGCGCCAGCAGCTGGACTACCTGGGCCAGCGTGTCATCGCCGCCGACTCCGGCGAAGCCGCCCTGGCCCTGTGGCGCG
AGCATGCGTTCGACGTCGTGATCACCGATTGCAACATGCCCGGCATCAGCGGCTACGAATtGGCGCGCCGCATACGCGCC
GCCGAGGCCGCGCCCGGTTACGGACGTACGCGGTGCATTCTGTTCGGCTTCACGGCTTCGGCGCAGATGGACGAAGCGCA
GCGCTGCCGCGCCGCCGGCATGGACGACTGCCTGTTCAAGCCGATCGGCGTGGACGCCTTGCGGCAACGCTTGAACGAAG
CCGTGGCACGGGCCGCGCTCCCCACGCCCCCCTCGCCCCAGGCTGCCGCGCCGGCCACGGACGACGCCACCCCGGCGGCG
TTCTCGGCCGAGTCGATTCTTGCCTTGACGCAGAACGATGAGGCGCTGATCCGGCAATTGCTCGAAGAAGTGATTCGCAC
CAACCGGGCGGACGTCGACCAATTGCAAAAGCTGCACCAGCAGGCCGATTGGCCGAAGGTCTCGGACATGGCGCACAGGC
TGGCCGGCGGCGCGCGCGTGGTCGATGCCAAGGCCATGATAGACACTGCGCTGGCGCTGGAGAAAAAAGCGCAAGGCCAG
GCTGGCCCCTCACCCGAAATCGACGGCCTGGTACGTACGCTTGCGGCGCAGTCCGCCGCGCTGGAGACGCAACTGCGCGC
CTGGCTGGAGCAACGGCCGCATCAAGATCAGCCCTGA
>Bb_B0224.seq
ATGCCCGCCCCGCACCGCCTGTACCCCCGCAGTCTGATCTGCCTGGCTCAGGCGCTATTGGCATGGGCTTTGCTGGCATG
GGCGCCCGCGCAGGCAAGCCAGGAGCTGACCCTGGTCGGCAAGGCTGCCGTTCCCGACGTCGAGATCACGCTCGACGGCG
ACGACTGGCGCTGGCTGGCGCGCAAGCGGGTGCTGACGCTGGGCGTGTACGCGCCGGACATTCCCCCGTTCGACGTCACC
TATGACGAGCGCTACGAAGGCCTGACGGCCGACTACATGGCGATCATCGCGCACAACCTGGGCGTCCAGGCAAAAGTGCT
GCGCTACCCCACGCGCGAGCAAGCCGTCGGCGCACTGGAAAGCGGACAGATCGACCTCATCGGCACCGTCAATGGCATCG
AGGGCCGGCTGCAGAGCCTGCGCCTGAGTGTTCCCTACGCGGCCGACCACCCGGTGCTGGTCATGCCCATCGGCGCGCGC
CGCGCTCCGCCCGCGGACCTGGCAGGCCAGCGGTTGGCGGTTGACGCCAACTACTTGCCCAGGGAAACGCTGCAGCAGGC
CTATCCCCAGGCAACGCTGCATTACTTCCCATCATCCGAACAGGCGCTGGCCGCGGTGGCCTATGGACAAGCCGACGTGT
TCATCGGCGATGCGCTGACCACCTCGCACCTCGTTTCGCAAAGCTACTTCAACGACGTTCGCGTCGTCGCCCCGGCCCAG
ATCGTGACGGGCGGGGAATCCTTCGGCGTGCGCGCCGACAATACCCGCCTGCTGCGGGTGGTCAATGCCGTGCTCGAAGC
CATTCCGGCCTCCGAGCGCCGCAGCCTGATCTACCGCTGGGGCCTGGGCAGCAGCATTTCGCTCGATTTCGCGCGCCCCG
CCTATTCAGCGCGCGAGCAGCAGTGGATGGCAAACCATCCGGTCGTCAAGGTGGCGGTCCTGAACCTGTTCGCGCCCTTC
ACCCTGTTTCGCACCGATGAACAGTTCGGCGGCATCAGTGCCGCCGTGCTGCAACTGCTGCAGTTGCGCACCGGCCTGGA
TTTCCAGATCATCGGCGTCGACACGGTCGAGGAGCTGATCGCCAAGCTGCGCTCGGGCGAAGCCGACATGGCCGGCGCCC
TGTTCGTCAATGCCGCGCGGGAATCCGTCCTCAGCTTCAGCCGGCCGTATGTGCGCAATGGTCTGGTGATCGTCACGCGC
CAGGACCCCGCCGCGCCCGCCGACGCCGATCACCTCGACGGCCGCACGATTGCGATGGTGCGCAACAGCGCCGCCATCCC
GCTCCTGCAGCAGCGCTATCCCCAGGCGAAGGTCGTGACCGCCGACAACCCGACCGAAGCCATGCTGCTGGTGGCCGATG
GCCAGGCCGACGCCGTCGTGCAGACGCAGATCAGCGCCAGCTACTACGTCAACCGCTACTTCGCCGGAAAACTGCGCATT
GCCTCGGCGCTGGACCTGCCGCCGGCCGAGATCGCGCTGGCGACGGCGCGCGGCCAGACCGAGCTGATATCCATCCTGAA
CAAGGCGCTCTACAGCATTTCGAACGACGAACTCGCCTCCATCGTCAGCCGCTGGCGCGGCAGCGACGGCGATCCGCGCA
CCTGGTACGCCTACCGCAACGAAATCTACCTGCTGATCGGGCTGGGCCTGTTGTCGGCCCTGCTGTTCCTGAGCTGGATC
GTCTACCTGCGGCGCCAGATCCGCCAGCGCAAGCGGGCCGAGCGGGCGCTGAACGACCAGCTGGAATTCATGCGCGTGCT
CATCGACGGCACGCCCAACCCCATCTATGTGCGCGATAAGGAAGGCCGCATGCTGTTGTGCAATGACGCCTACCTCGACA
CCTTTGGCGTGACTGCCGATGCGGTACTGGGCAAGACCATCCCGGAGGCCAACGTGGTGGGCGACCCGGCGCTGGCTCGC
GAGATGCACGAGTTCCTGCTCACGCGCATGGCCGCCGAGCGCGAGCCGCGCTTCGAGGACCGCGATGTCACGCTGCACGG
CCGCACCCGCCATGTCTACCAGTGGACGGTTCCGTACGGCGACTCGCTGGGCGAACTCAAGGGCATCATCGGCGGCTGGA
TCGACATTACCGAACGCGCCGAGCTGCTGCGCGAGCTGCACGACGCCAAGGAAAGCGCCGACGCCGCCAACCGGGCCAAG
ACCACGTTCCTGGCAACGATGAGCCACGAGATCCGCACGCCGATGAACGCGATCATCGGCATGCTGGAGCTGGCGCTGCT
CCGTCCGGCCGACCAGGAGCCGGACCGCCAGTCCATCCAGGTCGCGTACGACTCGGCCCGCAGCCTGCTGGAGCTGATAG
GCGACATCCTGGACATTGCGAAGATCGAGGCGGGAAAATTCGACCTGGCGCCGGTGCGCACGGCGCTGCGCGCCCTGCCC
GAAGGGGCGATCCGCGTCTTCGACGGGTTGGCGCGCCAGAAAGGCATAGAGCTGGTATTGAAGACCGACATCGTGGGCGT
GGACGATGTATTGATAGACCCCTTGCGCATGAAGCAAGTGCTCTCGAACCTGGTGGGCAACGCCATCAAGTTCACCACCG
AAGGCCAGGTTGTCCTTACCGTGACCGCGCGCCCCGACGGCGAGGCCGCGCACGTGCAGTTCAGCGTGAGCGACACCGGC
TGCGGCATCAGCGAGGCCGACCAACGGCAGCTGTTCAAACCGTTCTCGCAGGTGGGCGGCAGCGCCGAGGCCGGGCCGGC
GCCGGGCACTGGCCTGGGCCTGTCCATCAGCCGCCGCCTCGTCGAATTGATGGGGGGAACGCTGGTCATGCGCAGCGCGC
CAGGGGTGGGCACAACGGTTTCGGTGGACCTGAGGCTGACCATGGTCGAAAAATCCGCGCAGGCCACGCCGCCCGCTGCG
GCCGCTCAGGCCACGCCATCCAAGCCGCAGGTATCGCTGCGCGTGCTGGTCGTCGATGACCACAAGCCCAACCTGATGCT
GCTGCGCCAGCAGCTGGACTACCTGGGCCAGCGTGTCGTCGCCGCCGACTCCGGCGAAGCCGCCCTGGCCCTGTGGCACG
AGCATGCGTTCGACGTCGTGATCACCGATTGCAACATGCCCGGTATCAACGGCTACGAATTGGCGCGCCGCATACGCGCC
GCCGAGGCCGCGCCCGGTTACGGACGTACGCGGTGCATTCTGTTCGGCTTCACGGCTTCGGCGCAGATGGACGAAGCGCA
GCGCTGCCGCGCCGCCGGCATGGACGACTGCCTGTTCAAGCCGATCGGCGTGGACGCCTTGCGGCAACGCTTGAACGAAG
CCGCGGCACGGGCCGCGCTCCCCACGCCCCCCTCGCCCCAGGCTGCCGCGCCGGCCACGCACGACGCCACCCCGGCGGCG
TTCTCGGCCGAGTCGATTCTTGCCCTGACGCAGAACGATGAGGCGCTGATCCGGCAATTGCTCGAAGAAGTGATTCGCAC
CAACCGGGCGGACGTCGATCAATTGCAGAAGCTGCACCAGCAGGCCGATTGGCCGAAGGTCTCGGACATGGCGCACAGGC
TGGCCGGCGGCGCGCGCGTGGTCGATGCCAAGGCCATGATAGACACTGCGCTGGCGCTGGAGAAAAAAGCGCAAGGCCAG
GCTGGCCCCTCGCCCGAAATCGACGGCATGGTACGTACGCTTGCGGCGCAGTCCGCCGCGCTGGAGACGCAACTACGCGC
CTGGCTGGAGCAACGGCCGCATCAAGGCCAGCCCTGA
>Bb_B0233.seq
ATGCCCGCCCCGCACCGCCTGTACCCCCGCAGTCTGATCTGCCTGGCTCAGGCGCTATTGGCATGGGCTTTGCTGGCATG
GGCGCCCGCGCAGGCAAGCCAGGAGCTGACCCTGGTCGGCAAGGCTGCCGTTCCCGACGTCGAGATCACGCTCGACGGCG
ACGACTGGCGCTGGCTGGCGCGCAAGCGGGTGCTGACGCTGGGCGTGTACGCGCCGGACATTCCCCCGTTCGACGTCACC
TATGACGAGCGCTACGAAGGCCTGACGGCCGACTACATGGCGATCATCGCGCACAACCTGGGCGTCCAGGCAAAAGTGCT
GCGCTACCCCACGCGCGAGCAAGCCGTCGGCGCACTGGAAAGCGGACAGATCGACCTCATCGGCACCGTCAATGGCATCG
AGGGCCGGCTGCAGAGCCTGCGCCTGAGTGTTCCCTACGCGGCCGACCACCCGGTGCTGGTCATGCCCATCGGCGCGCGC
CGCGCTCCGCCCGCGGACCTGGCAGGCCAGCGGTTGGCGGTTGACGCCAACTACTTGCCCAGGGAAACGCTGCAGCAGGC
CTATCCCCAGGCAACGCTGCATTACTTCCCATCATCCGAACAGGCGCTGGCCGCGGTGGCCTATGGACAAGCCGACGTGT
TCATCGGCGATGCGCTGACCACCTCGCACCTCGTTTCGCAAAGCTACTTCAACGACGTTCGCGTCGTCGCCCCGGCCCAG
ATCGTGACGGGCGGGGAATCCTTCGGCGTGCGCGCCGACAATACCCGCCTGCTGCGGGTGGTCAATGCCGTGCTCGAAGC
CATTCCGGCCTCCGAGCGCCGCAGCCTGATCTACCGCTGGGGCCTGGGCAGCAGCATTTCGCTCGATTTCGCGCGCCCCG
CCTATTCAGCGCGCGAGCAGCAGTGGATGGCAAACCATCCGGTCGTCAAGGTGGCGGTCCTGAACCTGTTCGCGCCCTTC
ACCCTGTTTCGCACCGATGAACAGTTCGGCGGCATCAGTGCCGCCGTGCTGCAACTGCTGCAGTTGCGCACCGGCCTGGA
TTTCCAGATCATCGGCGTCGACACGGTCGAGGAGCTGATCGCCAAGCTGCGCTCGGGCGAAGCCGACATGGCCGGCGCCC
TGTTCGTCAATGCCGCGCGGGAATCCGTCCTCAGCTTCAGCCGGCCGTATGTGCGCAATGGTCTGGTGATCGTCACGCGC
CAGGACCCCGCCGCGCCCGCCGACGCCGATCACCTCGACGGCCGCACGATTGCGATGGTGCGCAACAGCGCCGCCATCCC
GCTCCTGCAGCAGCGCTATCCCCAGGCGAAGGTCGTGACCGCCGACAACCCGACCGAAGCCATGCTGCTGGTGGCCGATG
GCCAGGCCGACGCCGTCGTGCAGACGCAGATCAGCGCCAGCTACTACGTCAACCGCTACTTCGCCGGAAAACTGCGCATT
GCCTCGGCGCTGGACCTGCCGCCGGCCGAGATCGCGCTGGCGACGGCGCGCGGCCAGACCGAGCTGATATCCATCCTGAA
CAAGGCGCTCTACAGCATTTCGAACGACGAACTCGCCTCCATCGTCAGCCGCTGGCGCGGCAGCGACGGCGATCCGCGCA
CCTGGTACGCCTACCGCAACGAAATCTACCTGCTGATCGGGCTGGGCCTGTTGTCGGCCCTGCTGTTCCTGAGCTGGATC
GTCTACCTGCGGCGCCAGATCCGCCAGCGCAAGCGGGCCGAGCGGGCGCTGAACGACCAGCTGGAATTCATGCGCGTGCT
CATCGACGGCACGCCCAACCCCATCTATGTGCGCGATAAGGAAGGCCGCATGCTGTTGTGCAATGACGCCTACCTCGACA
CCTTTGGCGTGACTGCCGATGCGGTACTGGGCAAGACCATCCCGGAGGCCAACGTGGTGGGCGACCCGGCGCTGGCTCGC
GAGATGCACGAGTTCCTGCTCACGCGCATGGCCGCCGAGCGCGAGCCGCGCTTCGAGGACCGCGATGTCACGCTGCACGG
CCGCACCCGCCATGTCTACCAGTGGACGGTTCCGTACGGCGACTCGCTGGGCGAACTCAAGGGCATCATCGGCGGCTGGA
TCGACATTACCGAACGCGCCGAGCTGCTGCGCGAGCTGCACGACGCCAAGGAAAGCGCCGACGCCGCCAACCGGGCCAAG
ACCACGTTCCTGGCAACGATGAGCCACGAGATCCGCACGCCGATGAACGCGATCATCGGCATGCTGGAGCTGGCGCTGCT
CCGTCCGGCCGACCAGGAGCCGGACCGCCAGTCCATCCAGGTCGCGTACGACTCGGCCCGCAGCCTGCTGGAGCTGATAG
GCGACATCCTGGACATTGCGAAGATCGAGGCGGGAAAATTCGACCTGGCGCCGGTGCGCACGGCGCTGCGCGCCCTGCCC
GAAGGGGCGATCCGCGTCTTCGACGGGTTGGCGCGCCAGAAAGGCATAGAGCTGGTATTGAAGACCGACATCGTGGGCGT
GGACGATGTATTGATAGACCCCTTGCGCATGAAGCAAGTGCTCTCGAACCTGGTGGGCAACGCCATCAAGTTCACCACCG
AAGGCCAGGTTGTCCTTACCGTGACCGCGCGCCCCGACGGCGAGGCCGCGCACGTGCAGTTCAGCGTGAGCGACACCGGC
TGCGGCATCAGCGAGGCCGACCAACGGCAGCTGTTCAAACCGTTCTCGCAGGTGGGCGGCAGCGCCGAGGCCGGGCCGGC
GCCGGGCACTGGCCTGGGCCTGTCCATCAGCCGCCGCCTCGTCGAATTGATGGGGGGAACGCTGGTCATGCGCAGCGCGC
CAGGGGTGGGCACAACGGTTTCGGTGGACCTGAGGCTGACCATGGTCGAAAAATCCGCGCAGGCCACGCCGCCCGCTGCG
GCCGCTCAGGCCACGCCATCCAAGCCGCAGGTATCGCTGCGCGTGCTGGTCGTCGATGACCACAAGCCCAACCTGATGCT
GCTGCGCCAGCAGCTGGACTACCTGGGCCAGCGTGTCGTCGCCGCCGACTCCGGCGAAGCCGCCCTGGCCCTGTGGCACG
AGCATGCGTTCGACGTCGTGATCACCGATTGCAACATGCCCGGTATCAACGGCTACGAATTGGCGCGCCGCATACGCGCC
GCCGAGGCCGCGCCCGGTTACGGACGTACGCGGTGCATTCTGTTCGGCTTCACGGCTTCGGCGCAGATGGACGAAGCGCA
GCGCTGCCGCGCCGCCGGCATGGACGACTGCCTGTTCAAGCCGATCGGCGTGGACGCCTTGCGGCAACGCTTGAACGAAG
CCGCGGCACGGGCCGCGCTCCCCACGCCCCCCTCGCCCCAGGCTGCCGCGCCGGCCACGCACGACGCCACCCCGGCGGCG
TTCTCGGCCGAGTCGATTCTTGCCCTGACGCAGAACGATGAGGCGCTGATCCGGCAATTGCTCGAAGAAGTGATTCGCAC
CAACCGGGCGGACGTCGATCAATTGCAGAAGCTGCACCAGCAGGCCGATTGGCCGAAGGTCTCGGACATGGCGCACAGGC
TGGCCGGCGGCGCGCGCGTGGTCGATGCCAAGGCCATGATAGACACTGCGCTGGCGCTGGAGAAAAAAGCGCAAGGCCAG
GCTGGCCCCTCGCCCGAAATCGACGGCATGGTACGTACGCTTGCGGCGCAGTCCGCCGCGCTGGAGACGCAACTACGCGC
CTGGCTGGAGCAACGGCCGCATCAAGGCCAGCCCTGA
>Bb_B0230.seq
ATGCCCGCCCCGCACCGCCTGTACCCCCGCAGTCTGATCTGCCTGGCTCAGGCGCTATTGGCATGGGCTTTGCTGGCATG
GGCGCCCGCGCAGGCAAGCCAGGAGCTGACCCTGGTCGGCAAGGCTGCCGTTCCCGACGTCGAGGTCGCGCTCGACGGCG
ACGACTGGCGCTGGCTGGCGCGCAAGCGGGTGCTGACGCTGGGCGTGTACGCGCCGGACATTCCCCCGTTCGACGTCACC
TATGACGAGCGCTACGAAGGCCTGACGGCCGACTACATGGCGATCATCGCGCACAACCTGGGCGTCCAGGCAAAAGTGCT
GCGCTACCCCACGCGCGAGCAAGCCGTCGGCGCACTGGAAAGCGGGCAGATCGACCTCATCGGCACCGTCAATGGCATCG
AGGGCCGGCAGCAGAGCCTGCGCCTGAGTGTTCCCTACGCGGCCGACCACCCGGTGCTGGTCATGCCCATCGGCGCGCGC
CGCGCTCCGCCCGCGGACCTGGCAGGCCAGCGGTTGGCGGTTGACGCCAACTACTTGCCCAGGGAAACGCTGCAGCAGGC
CTATCCCCAGGCAACGCTGCATTACTTCCCATCGTCCGAACAGGCGCTGGCCGCGGTGGCCTATGGACAAGCCGACGTGT
TCATCGGCGATGCGCTGACCACCTCGCACCTCGTTTCGCAAAGCTACTTCAACGACGTTCGCGTCGTCGCCCCGGCCCAG
ATCGTGACGGGCGGGGAATCCTTCGGCGTGCGCGCCGACAATACCCGCCTGCTGCGGGTGGTCAATGCCGTGCTCGAAGC
CATTCCGGCCTCCGAGCGCCGCAGCCTGATCTACCGCTGGGGCCTGGGCAGCAGCATTTCGCTCGATTTCGCGCGCCCCG
CCTATTCAGCGCGCGAGCAGCAGTGGATGGCAAACCATCCGGTCGTCAAGGTGGCGGTCCTGAACCTGTTCGCGCCCTTC
ACCCTGTTTCGCACCGATGAACAGTTCGGCGGCATCAGTGCCGCCGTGCTGCAACTGCTGCAGTTGCGCACCGGCCTGGA
TTTCCAGATCATCGGCGTCGACACGGTCGACGAGCTGATCGCCAAGCTGCGCTCGGGCGAAGCCGACATGGCCGGCGCCC
TGTTCGTCAATGCCGCGCGGGAATCCGTCCTCAGCTTCAGCCGGCCGTATGTGCGCAATGGTCTGGTGATCGTCACGCGC
CAGGACCCCGCCGCGCCCGCCGACGCCGATCACCTCGACGGCCGCACGATTGCGATGGTGCGCAACAGCGCCGCCATCCC
GCTCCTGCAGCAGCGCTATCCCCAGGCGAAGGTCGTGACCGCCGACAACCCGACCGAAGCCATGCTGCTGGTGGCCGATG
GCCAGGCCGACGCCGTCGTGCAGACGCAGATCAGCGCCAGCTACTACGTCAACCGCTACTTCGCCGGAAAACTGCGCATT
GCCTCGGCGCTGGACCTGCCTCCGGCCGAGATCGCGCTGGCGACGGCGCGCGGCCAGACCGAGCTGATGTCCATCCTGAA
CAAGGCGCTCTACAGCATTTCGAACGACGAACTCGCCTCCATCGTCAGCCGCTGGCGTGGCAGCGACGGCGATCCGCGCA
CCTGGTACGCCTACCGCAACGAGATCTACCTGCTGATCGGGCTGGGCCTGTTGTCGGCCCTGCTGTTCCTGAGCTGGATC
GTCTACCTGCGGCGCCAGATCCGCCAACGCAAGCGGGCCGAGCGGGCGCTGAACGACCAGCTGGAATTCATGCGCGTGCT
CATCGACGGCACGCCCAACCCCATCTATGTGCGCGATAAGGAAGGCCGCATGCTGTTGTGCAATGACGCCTACCTCGACA
CCTTTGGCGTGACTGCCGATGCGGTACTGGGCAAGACCATCCCGGAGGCCAACGTGGTGGGCGACCCGGCGCTGGCTCGC
GAGATGCACGAGTTCCTGCTCACGCGCATGGCCGCCGAGCGCGAGCCGCGCTTCGAGGACCGCGATGTCACGCTGCACGG
CCGCACCCGCCATGTCTACCAGTGGACGGTTCCATACGGCGACTCGCTGGGCGAACTCAAGGGCATCATCGGCGGCTGGA
TCGACATTACCGAACGCGCCGAGCTGCTGCGCGAGCTGCACGACGCCAAGGAAAGCGCCGACGCCGCCAACCGGGCCAAG
ACCACGTTCCTGGCAACGATGAGCCACGAGATCCGCACGCCGATGAACGCGATCATCGGCATGCTGGAGCTGGCGCTGCT
CCGTCCGGCCGACCAGGAGCCGGACCGCCAGTCCATCCAGGTCGCGTACGACTCGGCCCGCAGCCTGCTGGAGCTGATAG
GCGACATCCTGGACATTGCGAAGATCGAGGCGGGAAAATTCGACCTGGCGCCGGTGCGCACGGCGCTGCGCGCCCTGCCC
GAAGGGGCGATCCGCGTCTTCGACGGGTTGGCGCGCCAGAAAGGCATAGAGCTGGTATTGAAGACCGACATCGTGGGCGT
GGACGATGTATTGATAGACCCCTTGCGCATGAAGCAAGTGCTCTCGAACCTGGTGGGCAACGCCATCAAGTTCACCACCG
AAGGCCAGGTTGTCCTTACCGTGACCGCGCGCCCCGACGGCGAGGCCGCGCACGTGCAGTTCAGCGTGAGCGACACCGGC
TGCGGCATCAGCGAGGCCGACCAACGGCACCTGTTCAAACCGTTCTCGCAGGTGGGTGGCAGCGCCGAGGCCGGGCCGGC
GCCGGGCACTGGCCTGGGCCTGTCCATCAGCCGCCGCCTCGTCGAATTGATGGGGGGAACGCTGGTCATGCGCAGCGCGC
CAGGGGTGGGCACAACGGTTTCGGTGGACCTGAGGCTGACCATGGTCGAAAAATCCGCGCAGGCCACGCCGCCCGCTGCG
GCCGCTCAGGCCACGCCATCCAAGCCGCAGGTATCGCTGCGCGTGCTGGTCGTCGATGACCACAAGCCCAACCTGATGCT
GCTGCGCCAGCAGCTGGACTACCTGGGCCAGCGTGTCGTCGCCGCCGACTCCGGCGAAGCCGCCCTGGCCCTGTGGCACG
AGCATGCGTTCGACGTCGTGATCACCGATTGCAACATGCCCGGTATCAACGGCTACGAATTGGCGCGCCGCATACGCGCC
GCCGAGGCCGCGCCCGGTTACGGACGTACGCGGTGCATTCTGTTCGGCTTCACGGCTTCGGCGCAGATGGACGAAGCGCA
GCGCTGCCGCGCCGCCGGCATGGACGACTGCCTGTTCAAGCCGATCGGCGTGGACGCCTTGCGGCAACGCTTGAACGAAG
CCGCGGCACGGGCCGCGCTCCCCACGCCCCCCTCGCCCCAGGCTGCCGCGCCGGCCACGCACGACGCCACCCCGGCGGCG
TTCTCGGCCGAGTCGATTCTTGCCCTGACGCAGAACGACGAGGCGCTGATCCGGCAATTGCTCGAAGAAGTGATTCGCAC
CAACCGGGCGGACGTCGATCAATTGCAGAAGCTGCACCAGCAGGCCGATTGGCCGAAGGTCTCGGACATGGCGCACAGGC
TGGCCGGCGGCGCGCGCGTGGTCGATGCCAAGGCCATGATAGACACTGCGCTGGCGCTGGAGAAAAAAGCGCAAGGCCAG
GCTGGCCCCTCGCCCGAAATCGACGGCCTGGTACGTACGCTTGCGGCGCAGTCCGCCGCGCTGGAGACGCAACTACGCGC
CTGGCTGGAGCAACGGCCGCATCAAGGCCAGCCCTGA
>Bb_B0226.seq
ATGCCCGCCCCGCACCGCCTGTACCCCCGCAGTCTGATCTGCCTGGCTCAGGCGCTATTGGCATGGGCTTTGCTGGCATG
GGCGCCCGCGCAGGCAAGCCAGGAGCTGACCCTGGTCGGCAAGGCTGCCGTTCCCGACGTCGAGATCACGCTCGACGGCG
ACGACTGGCGCTGGCTGGCGCGCAAGCGGGTGCTGACGCTGGGCGTGTACGCGCCGGACATTCCCCCGTTCGACGTCACC
TATGACGAGCGCTACGAAGGCCTGACGGCCGACTACATGGCGATCATCGCGCACAACCTGGGCGTCCAGGCAAAAGTGCT
GCGCTACCCCACGCGCGAGCAAGCCGTCGGCGCACTGGAAAGCGGACAGATCGACCTCATCGGCACGGTCAATGGCACCG
ACAGCCGGCAACAGAGCCTGAGTCTGAGCATTCCCTACGCCGCCGACCACCCGGTGATCGTCATGCCCATCGGCGCACGC
CACGTTCCAGCCTCGAACCTGGCCGGCCAGCGGTTGGCGGTTGACGCCAACTACTTGCCCAGGGAAACGCTGCAGCAGGC
CTATCCCCAGGCAACGCTGCATTACTTCCCATCGTCCGAACAGGCGCTGGCCGCGGTGGCCTATGGACAAGCCGACGTGT
TCATCGGCGATGCGCTGACCACCTCGCACCTCGTTTCGCAAAGCTACTTCAACGACGTTCGCGTCGTCGCCCCGGCCCAG
ATCGTGACGGGCGGGGAATCCTTCGGCGTGCGCGCCGACAATACCCGCCTGCTGCGGGTGGTCAATGCCGTGCTCGAAGC
CATTCCGGCCTCCGAGCGCCGCAGCCTGATCTACCGCTGGGGCCTGGGCAGCAGCATTTCGCTCGATTTCGCGCGCCCCG
CCTATTCAGCGCGCGAGCAGCAGTGGATGGCAAACCATCCGGTCGTCAAGGTGGCGGTCCTGAACCTGTTCGCGCCCTTC
ACCCTGTTTCGCACCGATGAACAGTTCGGCGGCATCAGTGCCGCCGTGCTGCAACTGCTGCAGTTGCGCACCGGCCTGGA
TTTCCAGATCATCGGCGTCGACACGGTCGAGGAGCTGATCGCCAAGCTGCGCTCGGGCGAAGCCGACATGGCCGGCGCCC
TGTTCGTCAATGCCGCGCGGGAATCCGTCCTCAGCTTCAGCCGGCCGTATGTGCGCAATGGTCTGGTGATCGTCACGCGC
CAGGACCCCGCCGCGCCCGCCGACGCCGATCACCTCGACGGCCGCACGATTGCGATGGTGCGCAACAGCGCCGCCATCCC
GCTCCTGCAGCAGCGCTATCCCCAGGCGAAGGTCGTGACCGCCGACAACCCGACCGAAGCCATGCTGCTGGTGGCCGATG
GCCAGGCCGACGCCGTCGTGCAGACGCAGATCAGCGCCAGCTACTACGTCAACCGCTACTTCGCCGGAAAACTGCGCATT
GCCTCGGCGCTGGACCTGCCGCCGGCCGAGATCGCGCTGGCGACGGCGCGCGGCCAGACCGAGCTGATATCCATCCTGAA
CAAGGCGCTCTACAGCATTTCGAACGACGAACTCGCCTCCATCGTCAGCCGCTGGCGCGGCAGCGACGGCGATCCGCGCA
CCTGGTACGCCTACCGCAACGAAATCTACCTGCTGATCGGGCTGGGCCTGTTGTCGGCCCTGCTGTTCCTGAGCTGGATC
GTCTACCTGCGGCGCCAGATCCGCCAGCGCAAGCGGGCCGAGCGGGCGCTGAACGACCAGCTGGAATTCATGCGCGTGCT
CATCGACGGCACGCCCAACCCCATCTATGTGCGCGATAAGGAAGGCCGCATGCTGTTGTGCAATGACGCCTACCTCGACA
CCTTTGGCGTGACTGCCGATGCGGTACTGGGCAAGACCATCCCGGAGGCCAACGTGGTGGGCGACCCGGCGCTGGCTCGC
GAGATGCACGAGTTCCTGCTCACGCGCATGGCCGCCGAGCGCGAGCCGCGCTTCGAGGACCGCGATGTCACGCTGCACGG
CCGCACCCGCCATGTCTACCAGTGGACGGTTCCGTACGGCGACTCGCTGGGCGAACTCAAGGGCATCATCGGCGGCTGGA
TCGACATTACCGAACGCGCCGAGCTGCTGCGCGAGCTGCACGACGCCAAGGAAAGCGCCGACGCCGCCAACCGGGCCAAG
ACCACGTTCCTGGCAACGATGAGCCACGAGATCCGCACGCCGATGAACGCGATCATCGGCATGCTGGAGCTGGCGCTGCT
CCGTCCGGCCGACCAGGAGCCGGACCGCCAGTCCATCCAGGTCGCGTACGACTCGGCCCGCAGCCTGCTGGAGCTGATAG
GCGACATCCTGGACATTGCGAAGATCGAGGCGGGAAAATTCGACCTGGCGCCGGTGCGCACGGCGCTGCGCGCCCTGCCC
GAAGGGGCGATCCGCGTCTTCGACGGGTTGGCGCGCCAGAAAGGCATAGAGCTGGTATTGAAGACCGACATCGTGGGCGT
GGACGATGTATTGATAGACCCCTTGCGCATGAAGCAAGTGCTCTCGAACCTGGTGGGCAACGCCATCAAGTTCACCACCG
AAGGCCAGGTTGTCCTTACCGTGACCGCGCGCCCCGACGGCGAGGCCGCGCACGTGCAGTTCAGCGTGAGCGACACCGGC
TGCGGCATCAGCGAGGCCGACCAACGGCAGCTGTTCAAACCGTTCTCGCAGGTGGGCGGCAGCGCCGAGGCCGGGCCGGC
GCCGGGCACTGGCCTGGGCCTGTCCATCAGCCGCCGCCTCGTCGAATTGATGGGGGGAACGCTGGTCATGCGCAGCGCGC
CAGGGGTGGGCACAACGGTTTCGGTGGACCTGAGGCTGACCATGGTCGAAAAATCCGCGCAGGCCACGCCGCCCGCTGCG
GCCGCTCAGGCCACGCCATCCAAGCCGCAGGTATCGCTGCGCGTGCTGGTCGTCGATGACCACAAGCCCAACCTGATGCT
GCTGCGCCAGCAGCTGGACTACCTGGGCCAGCGTGTCGTCGCCGCCGACTCCGGCGAAGCCGCCCTGGCCCTGTGGCACG
AGCATGCGTTCGACGTCGTGATCACCGATTGCAACATGCCCGGTATCAACGGCTACGAATTGGCGCGCCGCATACGCGCC
GCCGAGGCCGCGCCCGGTTACGGACGTACGCGGTGCATTCTGTTCGGCTTCACGGCTTCGGCGCAGATGGACGAAGCGCA
GCGCTGCCGCGCCGCCGGCATGGACGACTGCCTGTTCAAGCCGATCGGCGTGGACGCCTTGCGGCAACGCTTGAACGAAG
CCGCGGCACGGGCCGCGCTCCCCACGCCCCCCTCGCCCCAGGCTGCCGCGCCGGCCACGCACGACGCCACCCCGGCGGCG
TTCTCGGCCGAGTCGATTCTTGCCCTGACGCAGAACGACGAGGCGCTGATCCGGCAATTGCTCGAAGAAGTGATTCGCAC
CAACCGGGCGGACGTCGATCAATTGCAGAAGCTGCACCAGCAGGCCGATTGGCCGAAGGTCTCGGACATGGCGCACAGGC
TGGCCGGCGGCGCGCGCGTGGTCGATGCCAAGGCCATGATAGACACTGCGCTGGCGCTGGAGAAAAAAGCGCAAGGCCAG
GCTGGCCCCTCGCCCGAAATCGACGGCCTGGTACGTACGCTTGCGGCGCAGTCCGCCGCGCTGGAGACGCAACTACGCGC
CTGGCTGGAGCAACGGCCGCATCAAGGCCAGCCCTGA
>Bb_B0243.seq
ATGCCCGCCCCGCACCGCCTGTACCCCCGCAGTCTGATCTGCCTGGCTCAGGCGCTATTGGCATGGGCTTTGCTGGCATG
GGCGCCCGCGCAGGCAAGCCAGGAGCTGACCCTGGTCGGCAAGGCTGCCGTTCCCGACGTCGAGGTCGCGCTCGACGGCG
ACGACTGGCGTTGGCTGGCCCGCAAGCGGGTACTGACGCTGGGTGTGTACGCACCGGACATTCCTCCGTTCGACGTCACC
TATGGCGAACGCTACGAAGGCCTGACGGCCGACTACATGGCGATCATCGCGCACAACCTGGGGATGCAGGCGAAAGTGCT
GCGCTACCCCACGCGCGAACAAGCCCTCAGCGCGCTGGAAAGCGGGCAGATCGACCTCATCGGCACCGTCAATGGCACCG
ACGGCCGGCAACAGAGCCTGCGTCTGAGCGTTCCCTACGCCGCCGACCACCCGGTGATCGTCATGCCCATCGGCGCACGC
CACGTTCCAGCCTCGAACCTGGCCGGCCAGCGGCTGGCGGTCGACATCAACTACCTGCCCAAGGAAACGCTCGCACGGGC
CTACCCGCAGGCGACGCTGCATTACTTTCCCTCATCCGAGCAGGCGCTGGCCGCGGTGGCCTATGGGCAGGCCGACGTAT
TCATCGGCGATGCCCTGACCACCTCGCACCTCGTATCGCAAAGCTATTTCAATGACGTTCGCGCGGTCGCCCCGGCCCAT
ATCGCGACGGGCGGAGAATCCTTCGGCGTGCGCGCCGACAACACCCGCCTGCTGCGGGTGGTCAACGCCGTACTCGAAGC
CATTCCGCCTTCCGAACACCGCAGCCTGATCTACCGCTGGGGACTGGGCAGCAGCATTTCGCTCGATTTCGCGCACCCCG
CGTATTCCGCGCGCGAGCAGCAATGGATGGCAGACCACCCCGTCGTCAAGGTGGCGGTCCTGAATCTGTTCGCGCCCTTC
ACCCTGTTCCGCACCGACGAACAGTTCGGCGGGATCAGCGCCGCCGTGCTGCAGCTGCTGCAATTGCGCACCGGCCTGGA
CTTCGAGATCATCGGCGTCGACACGGTCGAGGAACTGATAGCCAAGCTGCGTTCGGGCGAAGCCGACATGGCCGGCGCCC
TGTTCGTCAACAGCGCGCGGGAGTCCTTCCTCAGTTTCAGCCGGCCGTATGTGCGCAATGGCATGGTGATCGTCACGCGC
CAGGACCCCGACGCGCCCGTCGACGCCGATCATCTGGACGGCCGCACGGTCGCGTTGGTGCGCAACAGCGCCGCCATTCC
TCTGCTGCAGCGGCGCTACCCCCAGGCGAAGGTGGTGACCGCCGACAACCCGAGCGAGGCGATGCTGATGGTGGCCAATG
GACAGGCCGACGCCGTCGTGCAGACGCAGATCAGCGCCAGCTATTACGTCAACCGCTACTTCGCCGGCAAGCTGCGCATC
GCCTCGGCGCTGGACCTGCCCCCGGCCGAGATCGCGCTGGCGACGACGCGCGGCCAGACCGAACTGATGTCCATCCTGAA
CAAGGCGCTCTACAGCATTTCGAACGACGAGCTCGCCTCCATCATCAGCCGCTGGCGCGGCAGCGACGGCGATCCGCGCA
CCTGGTACGCCTACCGCAACGAGATCTACCTGCTGATCGGGCTGGGCCTGTTGTCGGCCCTGCTGTTCCTGAGCTGGATC
GTCTACCTGCGGCGCCaGATCCGCCAGCGCAAGCGGGCCGAGCGGGCGCTGAACGACCAGCTGGAATTCATGCGCGTGCT
CATCGACGGCACGCCCAACCCCATCTATGTGCGCGATAAGGAAGGCCGCATGCTGTTGTGCAATGACGCCTACCTCGACA
CCTTTGGCGTGACTGCCGATGCGGTACTGGGCAAGACCATTCCGGAAGCCAACGTGGTGGGCGACCCGGCGCTGGCCCGC
GAAATGCACGAGTTCCTGCTCACGCGCGTGGCCGCCGAGCGCGAGCCGCGCTTCGAGGACCGCGATGTCACGCTGCACGG
CCGCACCCGCCATGTCTACCAGTGGACGATTCCGTACGGCGACTCGCTGGGCGAACTCAAGGGCATCATCGGCGGCTGGA
TCGACATCACCGAACGCGCCGAGCTGCTGCGCGAGCTGCACGACGCCAAGGAAAGCGCCGACGCCGCCAACCGGGCCAAG
ACCACGTTCCTGGCAACGATGAGCCACGAGATCCGCACGCCGATGaACGCGATCATCGGCATGCTGGAGCTGGCGCTGCT
CCGTCCGGCCGACCAGGAGCCGGATCGCCAGTCCATCCAGGTCGCGTACGACTCGGCCCGCAGCCTGCTGGAGCTGATAG
GCGACATCCTGGACATTGCGAAGATCGAGGCAGGAAAATTCGACCTGGCGCCGGTGCGCACGGCGCTGCGCGCCCTGCCC
GAAGGGGCGATCCGCGTCTTCGACGGATTGGCGCGCCAAAAAGGCATAGAGCTGGTATTGAAGACCGACATCGTGGGCGT
CGACGATGTATTGATAGACCCCTTGCGCATGAAGCAAGTGCTCTCGAACCTGGTGGGTAACGCCATCAAGTTCACCACCG
AAGGCCAGGTTGTCCTTGCCGTGACCGCACGCCCCGACGGCGAGGCCGCGCACGTGCAGTTCAGCGTGAGCGACACCGGC
TGCGGCATCAGCGAGGCCGACCAACGGCAGCTGTTCAAACCGTTCTCGCAGGTGGGTGGCAGCGCCGAGGCCGGGCCGGC
GCCGGGCACCGGCCTGGGCCTGTCCATCAGCCGTCGCCTCGTCGAATTGATGGGGGGAACGCTGGTCATGCGCAGCGCGC
CAGGGGTGGGCACAACGGTTTCGGTGGACCTGAGGCTGACCATGGTCGAAAAATCCGTGCAGGCCACGCCGCCCGCTGCG
GCCGCTGCGGCCACGCCGTCCAAGCCGCAGGTATCGCTGCGCGTGCTGGTCGTCGATGACCACAAACCCAACCTGATGCT
GCTGCGCCAGCAGCTGGACTACCTGGGCCAGCGTGTCATCGCCGCCGACTCCGGCGAAGCCGCCCTGGCCCTGTGGCGCG
AGCATGCGTTCGACGTCGTGATCACCGATTGCAACATGCCCGGTATCAGCGGCTACGAATtGGCGCGCCGCATACGCGCC
GCCGAGGCCGCGCCCGGTTACGGACGTACGCGGTGCATTCTGTTCGGCTTCACGGCTTCGGCGCAGATGGACGAAGCGCA
GCGCTGCCGCGCCGCCGGCATGGACGACTGCCTGTTCAAGCCGATCGGCGTGGACGCCTTGCGGCAACGCCTGAACGAAG
CCGTGGCACGGGCCGCGCTCCCCACGCCCCCCTCGCCCCAGGCTGCCGCGCCGGCCACGGACGACGCCACCCCGACGGCG
TTCTCGGCCGAATCGATTCTTGCCTTGACGCAGAACGATGAGGCGCTGATCCGGCAATTGCTCGAAGAAGTGATTCGCAC
CAACCGGGCGGACGTCGACCAATTGCAAAAGCTGCACCAGCAGGCCGATTGGCCGAAGGTCTCGGACATGGCGCACAGGC
TGGCCGGCGGCGCGCGCGTGGTCGATGCCAAGGCCATGATAGACACTGCGCTGGCGCTGGAGAAAAAAGCGCAAGGCCAG
GCTGGCCCCTCACCCGAAATCGACGGCCTGGTACGTACGCTTGCGGCGCAGTCCGCCGCGCTGGAGACGCAACTGCGCGC
CTGGCTGGAGCAACGGCCGCATCAAGATCAGCCCTGA
>Bb_B0246.seq
ATGCCCGCCCCGCACCGCCTGTACCCCCGCAGTCTGATCTGCCTGGCTCAGGCGCTATTGGCATGGGCTTTGCTGGCATG
GGCGCCCGCGCAGGCAAGCCAGGAGCTGACCCTGGTCGGCAAGGCTGCCGTTCCCGACGTCGAGATCACGCTCGACGGCG
ACGACTGGCGCTGGCTGGCGCGCAAGCGGGTGCTGACGCTGGGCGTGTACGCGCCGGACATTCCCCCGTTCGACGTCACC
TATGACGAGCGCTACGAAGGCCTGACGGCCGACTACATGGCGATCATCGCGCACAACCTGGGCGTCCAGGCAAAAGTGCT
GCGCTACCCCACGCGCGAGCAAGCCGTCGGCGCACTGGAAAGCGGACAGATCGACCTCATCGGCACCGTCAATGGCATCG
AGGGCCGGCTGCAGAGCCTGCGCCTGAGTGTTCCCTACGCGGCCGACCACCCGGTGCTGGTCATGCCCATCGGCGCGCGC
CGCGCTCCGCCCGCGGACCTGGCAGGCCAGCGGTTGGCGGTTGACGCCAACTACTTGCCCAGGGAAACGCTGCAGCAGGC
CTATCCCCAGGCAACGCTGCATTACTTCCCATCATCCGAACAGGCGCTGGCCGCGGTGGCCTATGGACAAGCCGACGTGT
TCATCGGCGATGCGCTGACCACCTCGCACCTCGTTTCGCAAAGCTACTTCAACGACGTTCGCGTCGTCGCCCCGGCCCAG
ATCGTGACGGGCGGGGAATCCTTCGGCGTGCGCGCCGACAATACCCGCCTGCTGCGGGTGGTCAATGCCGTGCTCGAAGC
CATTCCGGCCTCCGAGCGCCGCAGCCTGATCTACCGCTGGGGCCTGGGCAGCAGCATTTCGCTCGATTTCGCGCGCCCCG
CCTATTCAGCGCGCGAGCAGCAGTGGATGGCAAACCATCCGGTCGTCAAGGTGGCGGTCCTGAACCTGTTCGCGCCCTTC
ACCCTGTTTCGCACCGATGAACAGTTCGGCGGCATCAGTGCCGCCGTGCTGCAACTGCTGCAGTTGCGCACCGGCCTGGA
TTTCCAGATCATCGGCGTCGACACGGTCGAGGAGCTGATCGCCAAGCTGCGCTCGGGCGAAGCCGACATGGCCGGCGCCC
TGTTCGTCAATGCCGCGCGGGAATCCGTCCTCAGCTTCAGCCGGCCGTATGTGCGCAATGGTCTGGTGATCGTCACGCGC
CAGGACCCCGCCGCGCCCGCCGACGCCGATCACCTCGACGGCCGCACGATTGCGATGGTGCGCAACAGCGCCGCCATCCC
GCTCCTGCAGCAGCGCTATCCCCAGGCGAAGGTCGTGACCGCCGACAACCCGACCGAAGCCATGCTGCTGGTGGCCGATG
GCCAGGCCGACGCCGTCGTGCAGACGCAGATCAGCGCCAGCTACTACGTCAACCGCTACTTCGCCGGAAAACTGCGCATT
GCCTCGGCGCTGGACCTGCCGCCGGCCGAGATCGCGCTGGCGACGGCGCGCGGCCAGACCGAGCTGATATCCATCCTGAA
CAAGGCGCTCTACAGCATTTCGAACGACGAACTCGCCTCCATCGTCAGCCGCTGGCGCGGCAGCGACGGCGATCCGCGCA
CCTGGTACGCCTACCGCAACGAAATCTACCTGCTGATCGGGCTGGGCCTGTTGTCGGCCCTGCTGTTCCTGAGCTGGATC
GTCTACCTGCGGCGCCAGATCCGCCAGCGCAAGCGGGCCGAGCGGGCGCTGAACGACCAGCTGGAATTCATGCGCGTGCT
CATCGACGGCACGCCCAACCCCATCTATGTGCGCGATAAGGAAGGCCGCATGCTGTTGTGCAATGACGCCTACCTCGACA
CCTTTGGCGTGACTGCCGATGCGGTACTGGGCAAGACCATCCCGGAGGCCAACGTGGTGGGCGACCCGGCGCTGGCTCGC
GAGATGCACGAGTTCCTGCTCACGCGCATGGCCGCCGAGCGCGAGCCGCGCTTCGAGGACCGCGATGTCACGCTGCACGG
CCGCACCCGCCATGTCTACCAGTGGACGGTTCCGTACGGCGACTCGCTGGGCGAACTCAAGGGCATCATCGGCGGCTGGA
TCGACATTACCGAACGCGCCGAGCTGCTGCGCGAGCTGCACGACGCCAAGGAAAGCGCCGACGCCGCCAACCGGGCCAAG
ACCACGTTCCTGGCAACGATGAGCCACGAGATCCGCACGCCGATGAACGCGATCATCGGCATGCTGGAGCTGGCGCTGCT
CCGTCCGGCCGACCAGGAGCCGGACCGCCAGTCCATCCAGGTCGCGTACGACTCGGCCCGCAGCCTGCTGGAGCTGATAG
GCGACATCCTGGACATTGCGAAGATCGAGGCGGGAAAATTCGACCTGGCGCCGGTGCGCACGGCGCTGCGCGCCCTGCCC
GAAGGGGCGATCCGCGTCTTCGACGGGTTGGCGCGCCAGAAAGGCATAGAGCTGGTATTGAAGACCGACATCGTGGGCGT
GGACGATGTATTGATAGACCCCTTGCGCATGAAGCAAGTGCTCTCGAACCTGGTGGGCAACGCCATCAAGTTCACCACCG
AAGGCCAGGTTGTCCTTACCGTGACCGCGCGCCCCGACGGCGAGGCCGCGCACGTGCAGTTCAGCGTGAGCGACACCGGC
TGCGGCATCAGCGAGGCCGACCAACGGCAGCTGTTCAAACCGTTCTCGCAGGTGGGCGGCAGCGCCGAGGCCGGGCCGGC
GCCGGGCACTGGCCTGGGCCTGTCCATCAGCCGCCGCCTCGTCGAATTGATGGGGGGAACGCTGGTCATGCGCAGCGCGC
CAGGGGTGGGCACAACGGTTTCGGTGGACCTGAGGCTGACCATGGTCGAAAAATCCGCGCAGGCCACGCCGCCCGCTGCG
GCCGCTCAGGCCACGCCATCCAAGCCGCAGGTATCGCTGCGCGTGCTGGTCGTCGATGACCACAAGCCCAACCTGATGCT
GCTGCGCCAGCAGCTGGACTACCTGGGCCAGCGTGTCGTCGCCGCCGACTCCGGCGAAGCCGCCCTGGCCCTGTGGCACG
AGCATGCGTTCGACGTCGTGATCACCGATTGCAACATGCCCGGTATCAACGGCTACGAATTGGCGCGCCGCATACGCGCC
GCCGAGGCCGCGCCCGGTTACGGACGTACGCGGTGCATTCTGTTCGGCTTCACGGCTTCGGCGCAGATGGACGAAGCGCA
GCGCTGCCGCGCCGCCGGCATGGACGACTGCCTGTTCAAGCCGATCGGCGTGGACGCCTTGCGGCAACGCTTGAACGAAG
CCGCGGCACGGGCCGCGCTCCCCACGCCCCCCTCGCCCCAGGCTGCCGCGCCGGCCACGCACGACGCCACCCCGGCGGCG
TTCTCGGCCGAGTCGATTCTTGCCCTGACGCAGAACGATGAGGCGCTGATCCGGCAATTGCTCGAAGAAGTGATTCGCAC
CAACCGGGCGGACGTCGATCAATTGCAGAAGCTGCACCAGCAGGCCGATTGGCCGAAGGTCTCGGACATGGCGCACAGGC
TGGCCGGCGGCGCGCGCGTGGTCGATGCCAAGGCCATGATAGACACTGCGCTGGCGCTGGAGAAAAAAGCGCAAGGCCAG
GCTGGCCCCTCGCCCGAAATCGACGGCATGGTACGTACGCTTGCGGCGCAGTCCGCCGCGCTGGAGACGCAACTACGCGC
CTGGCTGGAGCAACGGCCGCATCAAGGCCAGCCCTGA
>Bb_B0231.seq
ATGCCCGCCCCGCACCGCCTGTACCCCCGCAGTCTGATCTGCCTGGCTCAGGCGCTATTGGCATGGGCTTTGCTGGCATG
GGCGCCCGCGCAGGCAAGCCAGGAGCTGACCCTGGTCGGCAAGGCTGCCGTTCCCGACGTCGAGATCACGCTCGACGGCG
ACGACTGGCGCTGGCTGGCGCGCAAGCGGGTGCTGACGCTGGGCGTGTACGCGCCGGACATTCCCCCGTTCGACGTCACC
TATGACGAGCGCTACGAAGGCCTGACGGCCGACTACATGGCGATCATCGCGCACAACCTGGGCGTCCAGGCAAAAGTGCT
GCGCTACCCCACGCGCGAGCAAGCCGTCGGCGCACTGGAAAGCGGACAGATCGACCTCATCGGCACCGTCAATGGCATCG
AGGGCCGGCTGCAGAGCCTGCGCCTGAGTGTTCCCTACGCGGCCGACCACCCGGTGCTGGTCATGCCCATCGGCGCGCGC
CGCGCTCCGCCCGCGGACCTGGCAGGCCAGCGGTTGGCGGTTGACGCCAACTACTTGCCCAGGGAAACGCTGCAGCAGGC
CTATCCCCAGGCAACGCTGCATTACTTCCCATCATCCGAACAGGCGCTGGCCGCGGTGGCCTATGGACAAGCCGACGTGT
TCATCGGCGATGCGCTGACCACCTCGCACCTCGTTTCGCAAAGCTACTTCAACGACGTTCGCGTCGTCGCCCCGGCCCAG
ATCGTGACGGGCGGGGAATCCTTCGGCGTGCGCGCCGACAATACCCGCCTGCTGCGGGTGGTCAATGCCGTGCTCGAAGC
CATTCCGGCCTCCGAGCGCCGCAGCCTGATCTACCGCTGGGGCCTGGGCAGCAGCATTTCGCTCGATTTCGCGCGCCCCG
CCTATTCAGCGCGCGAGCAGCAGTGGATGGCAAACCATCCGGTCGTCAAGGTGGCGGTCCTGAACCTGTTCGCGCCCTTC
ACCCTGTTTCGCACCGATGAACAGTTCGGCGGCATCAGTGCCGCCGTGCTGCAACTGCTGCAGTTGCGCACCGGCCTGGA
TTTCCAGATCATCGGCGTCGACACGGTCGAGGAGCTGATCGCCAAGCTGCGCTCGGGCGAAGCCGACATGGCCGGCGCCC
TGTTCGTCAATGCCGCGCGGGAATCCGTCCTCAGCTTCAGCCGGCCGTATGTGCGCAATGGTCTGGTGATCGTCACGCGC
CAGGACCCCGCCGCGCCCGCCGACGCCGATCACCTCGACGGCCGCACGATTGCGATGGTGCGCAACAGCGCCGCCATCCC
GCTCCTGCAGCAGCGCTATCCCCAGGCGAAGGTCGTGACCGCCGACAACCCGACCGAAGCCATGCTGCTGGTGGCCGATG
GCCAGGCCGACGCCGTCGTGCAGACGCAGATCAGCGCCAGCTACTACGTCAACCGCTACTTCGCCGGAAAACTGCGCATT
GCCTCGGCGCTGGACCTGCCGCCGGCCGAGATCGCGCTGGCGACGGCGCGCGGCCAGACCGAGCTGATATCCATCCTGAA
CAAGGCGCTCTACAGCATTTCGAACGACGAACTCGCCTCCATCGTCAGCCGCTGGCGCGGCAGCGACGGCGATCCGCGCA
CCTGGTACGCCTACCGCAACGAAATCTACCTGCTGATCGGGCTGGGCCTGTTGTCGGCCCTGCTGTTCCTGAGCTGGATC
GTCTACCTGCGGCGCCAGATCCGCCAGCGCAAGCGGGCCGAGCGGGCGCTGAACGACCAGCTGGAATTCATGCGCGTGCT
CATCGACGGCACGCCCAACCCCATCTATGTGCGCGATAAGGAAGGCCGCATGCTGTTGTGCAATGACGCCTACCTCGACA
CCTTTGGCGTGACTGCCGATGCGGTACTGGGCAAGACCATCCCGGAGGCCAACGTGGTGGGCGACCCGGCGCTGGCTCGC
GAGATGCACGAGTTCCTGCTCACGCGCATGGCCGCCGAGCGCGAGCCGCGCTTCGAGGACCGCGATGTCACGCTGCACGG
CCGCACCCGCCATGTCTACCAGTGGACGGTTCCGTACGGCGACTCGCTGGGCGAACTCAAGGGCATCATCGGCGGCTGGA
TCGACATTACCGAACGCGCCGAGCTGCTGCGCGAGCTGCACGACGCCAAGGAAAGCGCCGACGCCGCCAACCGGGCCAAG
ACCACGTTCCTGGCAACGATGAGCCACGAGATCCGCACGCCGATGAACGCGATCATCGGCATGCTGGAGCTGGCGCTGCT
CCGTCCGGCCGACCAGGAGCCGGACCGCCAGTCCATCCAGGTCGCGTACGACTCGGCCCGCAGCCTGCTGGAGCTGATAG
GCGACATCCTGGACATTGCGAAGATCGAGGCGGGAAAATTCGACCTGGCGCCGGTGCGCACGGCGCTGCGCGCCCTGCCC
GAAGGGGCGATCCGCGTCTTCGACGGGTTGGCGCGCCAGAAAGGCATAGAGCTGGTATTGAAGACCGACATCGTGGGCGT
GGACGATGTATTGATAGACCCCTTGCGCATGAAGCAAGTGCTCTCGAACCTGGTGGGCAACGCCATCAAGTTCACCACCG
AAGGCCAGGTTGTCCTTACCGTGACCGCGCGCCCCGACGGCGAGGCCGCGCACGTGCAGTTCAGCGTGAGCGACACCGGC
TGCGGCATCAGCGAGGCCGACCAACGGCAGCTGTTCAAACCGTTCTCGCAGGTGGGCGGCAGCGCCGAGGCCGGGCCGGC
GCCGGGCACTGGCCTGGGCCTGTCCATCAGCCGCCGCCTCGTCGAATTGATGGGGGGAACGCTGGTCATGCGCAGCGCGC
CAGGGGTGGGCACAACGGTTTCGGTGGACCTGAGGCTGACCATGGTCGAAAAATCCGCGCAGGCCACGCCGCCCGCTGCG
GCCGCTCAGGCCACGCCATCCAAGCCGCAGGTATCGCTGCGCGTGCTGGTCGTCGATGACCACAAGCCCAACCTGATGCT
GCTGCGCCAGCAGCTGGACTACCTGGGCCAGCGTGTCGTCGCCGCCGACTCCGGCGAAGCCGCCCTGGCCCTGTGGCACG
AGCATGCGTTCGACGTCGTGATCACCGATTGCAACATGCCCGGTATCAACGGCTACGAATTGGCGCGCCGCATACGCGCC
GCCGAGGCCGCGCCCGGTTACGGACGTACGCGGTGCATTCTGTTCGGCTTCACGGCTTCGGCGCAGATGGACGAAGCGCA
GCGCTGCCGCGCCGCCGGCATGGACGACTGCCTGTTCAAGCCGATCGGCGTGGACGCCTTGCGGCAACGCTTGAACGAAG
CCGCGGCACGGGCCGCGCTCCCCACGCCCCCCTCGCCCCAGGCTGCCGCGCCGGCCACGCACGACGCCACCCCGGCGGCG
TTCTCGGCCGAGTCGATTCTTGCCCTGACGCAGAACGATGAGGCGCTGATCCGGCAATTGCTCGAAGAAGTGATTCGCAC
CAACCGGGCGGACGTCGATCAATTGCAGAAGCTGCACCAGCAGGCCGATTGGCCGAAGGTCTCGGACATGGCGCACAGGC
TGGCCGGCGGCGCGCGCGTGGTCGATGCCAAGGCCATGATAGACACTGCGCTGGCGCTGGAGAAAAAAGCGCAAGGCCAG
GCTGGCCCCTCGCCCGAAATCGACGGCATGGTACGTACGCTTGCGGCGCAGTCCGCCGCGCTGGAGACGCAACTACGCGC
CTGGCTGGAGCAACGGCCGCATCAAGGCCAGCCCTGA
>Bb_B0189.seq
ATGCCCGCCCCGCACCGCCTGTACCCCCGCAGTCTGATCTGCCTGGCTCAGGCGCTATTGGCATGGGCTTTGCTGGCATG
GGCGCCCGCGCAGGCAAGCCAGGAGCTGACCCTGGTCGGCAAGGCTGCCGTTCCCGACGTCGAGATCACGCTCGACGGCG
ACGACTGGCGCTGGCTGGCGCGCAAGCGGGTGCTGACGCTGGGCGTGTACGCGCCGGACATTCCCCCGTTCGACGTCACC
TATGACGAGCGCTACGAAGGCCTGACGGCCGACTACATGGCGATCATCGCGCACAACCTGGGCGTCCAGGCAAAAGTGCT
GCGCTACCCCACGCGCGAGCAAGCCGTCGGCGCACTGGAAAGCGGACAGATCGACCTCATCGGCACCGTCAATGGCATCG
AGGGCCGGCTGCAGAGCCTGCGCCTGAGTGTTCCCTACGCGGCCGACCACCCGGTGCTGGTCATGCCCATCGGCGCGCGC
CGCGCTCCGCCCGCGGACCTGGCAGGCCAGCGGTTGGCGGTTGACGCCAACTACTTGCCCAGGGAAACGCTGCAGCAGGC
CTATCCCCAGGCAACGCTGCATTACTTCCCATCATCCGAACAGGCGCTGGCCGCGGTGGCCTATGGACAAGCCGACGTGT
TCATCGGCGATGCGCTGACCACCTCGCACCTCGTTTCGCAAAGCTACTTCAACGACGTTCGCGTCGTCGCCCCGGCCCAG
ATCGTGACGGGCGGGGAATCCTTCGGCGTGCGCGCCGACAATACCCGCCTGCTGCGGGTGGTCAATGCCGTGCTCGAAGC
CATTCCGGCCTCCGAGCGCCGCAGCCTGATCTACCGCTGGGGCCTGGGCAGCAGCATTTCGCTCGATTTCGCGCGCCCCG
CCTATTCAGCGCGCGAGCAGCAGTGGATGGCAAACCATCCGGTCGTCAAGGTGGCGGTCCTGAACCTGTTCGCGCCCTTC
ACCCTGTTTCGCACCGATGAACAGTTCGGCGGCATCAGTGCCGCCGTGCTGCAACTGCTGCAGTTGCGCACCGGCCTGGA
TTTCCAGATCATCGGCGTCGACACGGTCGAGGAGCTGATCGCCAAGCTGCGCTCGGGCGAAGCCGACATGGCCGGCGCCC
TGTTCGTCAATGCCGCGCGGGAATCCGTCCTCAGCTTCAGCCGGCCGTATGTGCGCAATGGTCTGGTGATCGTCACGCGC
CAGGACCCCGCCGCGCCCGCCGACGCCGATCACCTCGACGGCCGCACGATTGCGATGGTGCGCAACAGCGCCGCCATCCC
GCTCCTGCAGCAGCGCTATCCCCAGGCGAAGGTCGTGACCGCCGACAACCCGACCGAAGCCATGCTGCTGGTGGCCGATG
GCCAGGCCGACGCCGTCGTGCAGACGCAGATCAGCGCCAGCTACTACGTCAACCGCTACTTCGCCGGAAAACTGCGCATT
GCCTCGGCGCTGGACCTGCCGCCGGCCGAGATCGCGCTGGCGACGGCGCGCGGCCAGACCGAGCTGATATCCATCCTGAA
CAAGGCGCTCTACAGCATTTCGAACGACGAACTCGCCTCCATCGTCAGCCGCTGGCGCGGCAGCGACGGCGATCCGCGCA
CCTGGTACGCCTACCGCAACGAAATCTACCTGCTGATCGGGCTGGGCCTGTTGTCGGCCCTGCTGTTCCTGAGCTGGATC
GTCTACCTGCGGCGCCAGATCCGCCAGCGCAAGCGGGCCGAGCGGGCGCTGAACGACCAGCTGGAATTCATGCGCGTGCT
CATCGACGGCACGCCCAACCCCATCTATGTGCGCGATAAGGAAGGCCGCATGCTGTTGTGCAATGACGCCTACCTCGACA
CCTTTGGCGTGACTGCCGATGCGGTACTGGGCAAGACCATCCCGGAGGCCAACGTGGTGGGCGACCCGGCGCTGGCTCGC
GAGATGCACGAGTTCCTGCTCACGCGCATGGCCGCCGAGCGCGAGCCGCGCTTCGAGGACCGCGATGTCACGCTGCACGG
CCGCACCCGCCATGTCTACCAGTGGACGGTTCCGTACGGCGACTCGCTGGGCGAACTCAAGGGCATCATCGGCGGCTGGA
TCGACATTACCGAACGCGCCGAGCTGCTGCGCGAGCTGCACGACGCCaAGGAAAGCGCCGACGCCGCCAACCGGGCCAAG
ACCACGTTCCTGGCAACGATGAGCCACGAGATCCGCACGCCGATGAACGCGATCATCGGCATGCTGGAGCTGGCGCTGCT
CCGTCCGGCCGACCAGGAGCCGGACCGCCAGTCCATCCAGGTCGCGTACGACTCGGCCCGCAGCCTGCTGGAGCTGATAG
GCGACATCCTGGACATTGCGAAGATCGAGGCGGGAAAATTCGACCTGGCGCCGGTGCGCACGGCGCTGCGCGCCCTGCCC
GAAGGGGCGATCCGCGTCTTCGACGGGTTGGCGCGCCAGAAAGGCATAGAGCTGGTATTGAAGACCGACATCGTGGGCGT
GGACGATGTATTGATAGACCCCTTGCGCATGAAGCAAGTGCTCTCGAACCTGGTGGGCAACGCCATCAAGTTCACCACCG
AAGGCCAGGTTGTCCTTACCGTGACCGCGCGCCCCGACGGCGAGGCCGCGCACGTGCAGTTCAGCGTGAGCGACACCGGC
TGCGGCATCAGCGAGGCCGACCAACGGCAGCTGTTCAAACCGTTCTCGCAGGTGGGCGGCAGCGCCGAGGCCGGGCCGGC
GCCGGGCACTGGCCTGGGCCTGTCCATCAGCCGCCGCCTCGTCGAATTGATGGGGGGAACGCTGGTCATGCGCAGCGCGC
CAGGGGTGGGCACAACGGTTTCGGTGGACCTGAGGCTGACCATGGTCGAAAAATCCGCGCAGGCCACGCCGCCCGCTGCG
GCCGCTCAGGCCACGCCATCCAAGCCGCAGGTATCGCTGCGCGTGCTGGTCGTCGATGACCACAAGCCCAACCTGATGCT
GCTGCGCCAGCAGCTGGACTACCTGGGCCAGCGTGTCGTCGCCGCCGACTCCGGCGAAGCCGCCCTGGCCCTGTGGCACG
AGCATGCGTTCGACGTCGTGATCACCGATTGCAACATGCCCGGTATCAACGGCTACGAATTGGCGCGCCGCATACGCGCC
GCCGAGGCCGCGCCCGGTTACGGACGTACGCGGTGCATTCTGTTCGGCTTCACGGCTTCGGCGCAGATGGACGAAGCGCA
GCGCTGCCGCGCCGCCGGCATGGACGACTGCCTGTTCAAGCCGATCGGCGTGGACGCCTTGCGGCAACGCTTGAACGAAG
CCGCGGCACGGGCCGCGCTCCCCACGCCCCCCTCGCCCCAGGCTGCCGCGCCGGCCACGCACGACGCCACCCCGGCGGCG
TTCTCGGCCGAGTCGATTCTTGCCCTGACGCAGAACGATGAGGCGCTGATCCGGCAATTGCTCGAAGAAGTGATTCGCAC
CAACCGGGCGGACGTCGATCAATTGCAGAAGCTGCACCAGCAGGCCGATTGGCCGAAGGTCTCGGACATGGCGCACAGGC
TGGCCGGCGGCGCGCGCGTGGTCGATGCCAAGGCCATGATAGACACTGCGCTGGCGCTGGAGAAAAAAGCGCAAGGCCAG
GCTGGCCCCTCGCCCGAAATCGACGGCATGGTACGTACGCTTGCGGCGCAGTCCGCCGCGCTGGAGACGCAACTACGCGC
CTGGCTGGAGCAACGGCCGCATCAAGGCCAGCCCTGA
>Bb_B0084.seq
ATGCCCGCCCCGCACCGCCTGTACCCCCGCAGTCTGATCTGCCTGGCTCAGGCGCTATTGGCATGGGCTTTGCTGGCATG
GGCGCCCGCGCAGGCAAGCCAGGAGCTGACCCTGGTCGGCAAGGCTGCCGTTCCCGACGTCGAGATCACGCTCGACGGCG
ACGACTGGCGCTGGCTGGCGCGCAAGCGGGTGCTGACGCTGGGCGTGTACGCGCCGGACATTCCCCCGTTCGACGTCACC
TATGACGAGCGCTACGAAGGCCTGACGGCCGACTACATGGCGATCATCGCGCACAACCTGGGCGTCCAGGCAAAAGTGCT
GCGCTACCCCACGCGCGAGCAAGCCGTCGGCGCACTGGAAAGCGGACAGATCGACCTCATCGGCACCGTCAATGGCATCG
AGGGCCGGCTGCAGAGCCTGCGCCTGAGTGTTCCCTACGCGGCCGACCACCCGGTGCTGGTCATGCCCATCGGCGCGCGC
CGCGCTCCGCCCGCGGACCTGGCAGGCCAGCGGTTGGCGGTTGACGCCAACTACTTGCCCAGGGAAACGCTGCAGCAGGC
CTATCCCCAGGCAACGCTGCATTACTTCCCATCATCCGAACAGGCGCTGGCCGCGGTGGCCTATGGACAAGCCGACGTGT
TCATCGGCGATGCGCTGACCACCTCGCACCTCGTTTCGCAAAGCTACTTCAACGACGTTCGCGTCGTCGCCCCGGCCCAG
ATCGTGACGGGCGGGGAATCCTTCGGCGTGCGCGCCGACAATACCCGCCTGCTGCGGGTGGTCAATGCCGTGCTCGAAGC
CATTCCGGCCTCCGAGCGCCGCAGCCTGATCTACCGCTGGGGCCTGGGCAGCAGCATTTCGCTCGATTTCGCGCGCCCCG
CCTATTCAGCGCGCGAGCAGCAGTGGATGGCAAACCATCCGGTCGTCAAGGTGGCGGTCCTGAACCTGTTCGCGCCCTTC
ACCCTGTTTCGCACCGATGAACAGTTCGGCGGCATCAGTGCCGCCGTGCTGCAACTGCTGCAGTTGCGCACCGGCCTGGA
TTTCCAGATCATCGGCGTCGACACGGTCGAGGAGCTGATCGCCAAGCTGCGCTCGGGCGAAGCCGACATGGCCGGCGCCC
TGTTCGTCAATGCCGCGCGGGAATCCGTCCTCAGCTTCAGCCGGCCGTATGTGCGCAATGGTCTGGTGATCGTCACGCGC
CAGGACCCCGCCGCGCCCGCCGACGCCGATCACCTCGACGGCCGCACGATTGCGATGGTGCGCAACAGCGCCGCCATCCC
GCTCCTGCAGCAGCGCTATCCCCAGGCGAAGGTCGTGACCGCCGACAACCCGACCGAAGCCATGCTGCTGGTGGCCGATG
GCCAGGCCGACGCCGTCGTGCAGACGCAGATCAGCGCCAGCTACTACGTCAACCGCTACTTCGCCGGAAAACTGCGCATT
GCCTCGGCGCTGGACCTGCCGCCGGCCGAGATCGCGCTGGCGACGGCGCGCGGCCAGACCGAGCTGATATCCATCCTGAA
CAAGGCGCTCTACAGCATTTCGAACGACGAACTCGCCTCCATCGTCAGCCGCTGGCGCGGCAGCGACGGCGATCCGCGCA
CCTGGTACGCCTACCGCAACGAAATCTACCTGCTGATCGGGCTGGGCCTGTTGTCGGCCCTGCTGTTCCTGAGCTGGATC
GTCTACCTGCGGCGCCAGATCCGCCAGCGCAAGCGGGCCGAGCGGGCGCTGAACGACCAGCTGGAATTCATGCGCGTGCT
CATCGACGGCACGCCCAACCCCATCTATGTGCGCGATAAGGAAGGCCGCATGCTGTTGTGCAATGACGCCTACCTCGACA
CCTTTGGCGTGACTGCCGATGCGGTACTGGGCAAGACCATCCCGGAGGCCAACGTGGTGGGCGACCCGGCGCTGGCTCGC
GAGATGCACGAGTTCCTGCTCACGCGCATGGCCGCCGAGCGCGAGCCGCGCTTCGAGGACCGCGATGTCACGCTGCACGG
CCGCACCCGCCATGTCTACCAGTGGACGGTTCCGTACGGCGACTCGCTGGGCGAACTCAAGGGCATCATCGGCGGCTGGA
TCGACATTACCGAACGCGCCGAGCTGCTGCGCGAGCTGCACGACGCCAAGGAAAGCGCCGACGCCGCCAACCGGGCCAAG
ACCACGTTCCTGGCAACGATGAGCCACGAGATCCGCACGCCGATGAACGCGATCATCGGCATGCTGGAGCTGGCGCTGCT
CCGTCCGGCCGACCAGGAGCCGGACCGCCAGTCCATCCAGGTCGCGTACGACTCGGCCCGCAGCCTGCTGGAGCTGATAG
GCGACATCCTGGACATTGCGAAGATCGAGGCGGGAAAATTCGACCTGGCGCCGGTGCGCACGGCGCTGCGCGCCCTGCCC
GAAGGGGCGATCCGCGTCTTCGACGGGTTGGCGCGCCAGAAAGGCATAGAGCTGGTATTGAAGACCGACATCGTGGGCGT
GGACGATGTATTGATAGACCCCTTGCGCATGAAGCAAGTGCTCTCGAACCTGGTGGGCAACGCCATCAAGTTCACCACCG
AAGGCCAGGTTGTCCTTACCGTGACCGCGCGCCCCGACGGCGAGGCCGCGCACGTGCAGTTCAGCGTGAGCGACACCGGC
TGCGGCATCAGCGAGGCCGACCAACGGCAGCTGTTCAAACCGTTCTCGCAGGTGGGCGGCAGCGCCGAGGCCGGGCCGGC
GCCGGGCACTGGCCTGGGCCTGTCCATCAGCCGCCGCCTCGTCGAATTGATGGGGGGAACGCTGGTCATGCGCAGCGCGC
CAGGGGTGGGCACAACGGTTTCGGTGGACCTGAGGCTGACCATGGTCGAAAAATCCGCGCAGGCCACGCCGCCCGCTGCG
GCCGCTCAGGCCACGCCATCCAAGCCGCAGGTATCGCTGCGCGTGCTGGTCGTCGATGACCACAAGCCCAACCTGATGCT
GCTGCGCCAGCAGCTGGACTACCTGGGCCAGCGTGTCGTCGCCGCCGACTCCGGCGAAGCCGCCCTGGCCCTGTGGCACG
AGCATGCGTTCGACGTCGTGATCACCGATTGCAACATGCCCGGTATCAACGGCTACGAATTGGCGCGCCGCATACGCGCC
GCCGAGGCCGCGCCCGGTTACGGACGTACGCGGTGCATTCTGTTCGGCTTCACGGCTTCGGCGCAGATGGACGAAGCGCA
GCGCTGCCGCGCCGCCGGCATGGACGACTGCCTGTTCAAGCCGATCGGCGTGGACGCCTTGCGGCAACGCTTGAACGAAG
CCGCGGCACGGGCCGCGCTCCCCACGCCCCCCTCGCCCCAGGCTGCCGCGCCGGCCACGCACGACGCCACCCCGGCGGCG
TTCTCGGCCGAGTCGATTCTTGCCCTGACGCAGAACGATGAGGCGCTGATCCGGCAATTGCTCGAAGAAGTGATTCGCAC
CAACCGGGCGGACGTCGATCAATTGCAGAAGCTGCACCAGCAGGCCGATTGGCCGAAGGTCTCGGACATGGCGCACAGGC
TGGCCGGCGGCGCGCGCGTGGTCGATGCCAAGGCCATGATAGACACTGCGCTGGCGCTGGAGAAAAAAGCGCAAGGCCAG
GCTGGCCCCTCGCCCGAAATCGACGGCATGGTACGTACGCTTGCGGCGCAGTCCGCCGCGCTGGAGACGCAACTACGCGC
CTGGCTGGAGCAACGGCCGCATCAAGGCCAGCCCTGA
>Bb_B0232.seq
ATGCCCGCCCCGCACCGCCTGTACCCCCGCAGTCTGATCTGCCTGGCTCAGGCGCTATTGGCATGGGCTTTGCTGGCATG
GGCGCCCGCGCAGGCAAGCCAGGAGCTGACCCTGGTCGGCAAGGCTGCCGTTCCCGACGTCGAGGTCGCGCTCGACGGCG
ACGACTGGCGTTGGCTGGCCCGCAAGCGGGTACTGACGCTGGGTGTGTACGCACCGGACATTCCTCCGTTCGACGTCACC
TATGGCGAACGCTACGAAGGCCTGACGGCCGACTACATGGCGATCATCGCGCACAACCTGGGGATGCAGGCGAAAGTGCT
GCGCTACCCCACGCGCGAACAAGCCCTCAGCGCGCTGGAAAGCGGGCAGATCGACCTCATCGGCACCGTCAATGGCACCG
ACGGCCGGCAACAGAGCCTGCGTCTGAGCGTTCCCTACGCCGCCGACCACCCGGTGATCGTCATGCCCATCGGCGCACGC
CACGTTCCAGCCTCGAACCTGGCCGGCCAGCGGCTGGCGGTCGACATCAACTACCTGCCCAAGGAAACGCTCGCACGGGC
CTACCCGCAGGCGACGCTGCATTACTTTCCCTCATCCGAGCAGGCGCTGGCCGCGGTGGCCTATGGGCAGGCCGACGTAT
TCATCGGCGATGCCCTGACCACCTCGCACCTCGTATCGCAAAGCTATTTCAATGACGTTCGCGCGGTCGCCCCGGCCCAT
ATCGCGACGGGCGGAGAATCCTTCGGCGTGCGCGCCGACAACACCCGCCTGCTGCGGGTGGTCAACGCCGTACTCGAAGC
CATTCCGCCTTCCGAACACCGCAGCCTGATCTACCGCTGGGGACTGGGCAGCAGCATTTCGCTCGATTTCGCGCACCCCG
CGTATTCCGCGCGCGAGCAGCAATGGATGGCAGACCACCCCGTCGTCAAGGTGGCGGTCCTGAATCTGTTCGCGCCCTTC
ACCCTGTTCCGCACCGACGAACAGTTCGGCGGGATCAGCGCCGCCGTGCTGCAGCTGCTGCAATTGCGCACCGGCCTGGA
CTTCGAGATCATCGGCGTCGACACGGTCGAGGAACTGATAGCCAAGCTGCGTTCGGGCGAAGCCGACATGGCCGGCGCCC
TGTTCGTCAACAGCGCGCGGGAGTCCTTCCTCAGTTTCAGCCGGCCGTATGTGCGCAATGGCATGGTGATCGTCACGCGC
CAGGACCCCGACGCGCCCGTCGACGCCGATCATCTGGACGGCCGCACGGTCGCGTTGGTGCGCAACAGCGCCGCCATTCC
TCTGCTGCAGCGGCGCTACCCCCAGGCGAAGGTGGTGACCGCCGACAACCCGAGCGAGGCGATGCTGATGGTGGCCAATG
GACAGGCCGACGCCGTCGTGCAGACGCAGATCAGCGCCAGCTATTACGTCAACCGCTACTTCGCCGGCAAGCTGCGCATC
GCCTCGGCGCTGGACCTGCCCCCGGCCGAGATCGCGCTGGCGACGACGCGCGGCCAGACCGAACTGATGTCCATCCTGAA
CAAGGCGCTCTACAGCATTTCGAACGACGAGCTCGCCTCCATCATCAGCCGCTGGCGCGGCAGCGACGGCGATCCGCGCA
CCTGGTACGCCTACCGCAACGAGATCTACCTGCTGATCGGGCTGGGCCTGTTGTCGGCCCTGCTGTTCCTGAGCTGGATC
GTCTACCTGCGGCGCCaGATCCGCCAGCGCAAGCGGGCCGAGCGGGCGCTGAACGACCAGCTGGAATTCATGCGCGTGCT
CATCGACGGCACGCCCAACCCCATCTATGTGCGCGATAAGGAAGGCCGCATGCTGTTGTGCAATGACGCCTACCTCGACA
CCTTTGGCGTGACTGCCGATGCGGTACTGGGCAAGACCATTCCGGAAGCCAACGTGGTGGGCGACCCGGCGCTGGCCCGC
GAAATGCACGAGTTCCTGCTCACGCGCGTGGCCGCCGAGCGCGAGCCGCGCTTCGAGGACCGCGATGTCACGCTGCACGG
CCGCACCCGCCATGTCTACCAGTGGACGATTCCGTACGGCGACTCGCTGGGCGAACTCAAGGGCATCATCGGCGGCTGGA
TCGACATCACCGAACGCGCCGAGCTGCTGCGCGAGCTGCACGACGCCAAGGAAAGCGCCGACGCCGCCAACCGGGCCAAG
ACCACGTTCCTGGCAACGATGAGCCACGAGATCCGCACGCCGATGaACGCGATCATCGGCATGCTGGAGCTGGCGCTGCT
CCGTCCGGCCGACCAGGAGCCGGATCGCCAGTCCATCCAGGTCGCGTACGACTCGGCCCGCAGCCTGCTGGAGCTGATAG
GCGACATCCTGGACATTGCGAAGATCGAGGCAGGAAAATTCGACCTGGCGCCGGTGCGCACGGCGCTGCGCGCCCTGCCC
GAAGGGGCGATCCGCGTCTTCGACGGATTGGCGCGCCAAAAAGGCATAGAGCTGGTATTGAAGACCGACATCGTGGGCGT
CGACGATGTATTGATAGACCCCTTGCGCATGAAGCAAGTGCTCTCGAACCTGGTGGGTAACGCCATCAAGTTCACCACCG
AAGGCCAGGTTGTCCTTGCCGTGACCGCACGCCCCGACGGCGAGGCCGCGCACGTGCAGTTCAGCGTGAGCGACACCGGC
TGCGGCATCAGCGAGGCCGACCAACGGCAGCTGTTCAAACCGTTCTCGCAGGTGGGTGGCAGCGCCGAGGCCGGGCCGGC
GCCGGGCACCGGCCTGGGCCTGTCCATCAGCCGCCGCCTCGTCGAATTGATGGGGGGAACGCTGGTCATGCGCAGCGCGC
CAGGGGTGGGCACAACGGTTTCGGTGGACCTGAGGCTGACCATGGTCGAAAAATCCGTGCAGGCCACGCCGCCCGCTGCG
GCCGCTGCGGCCACGCCGTCCAAGCCGCAGGTATCGCTGCGCGTGCTGGTCGTCGATGACCACAAACCCAACCTGATGCT
GCTGCGCCAGCAGCTGGACTACCTGGGCCAGCGTGTCATCGCCGCCGACTCCGGCGAAGCCGCCCTGGCCCTGTGGCGCG
AGCATGCGTTCGACGTCGTGATCACCGATTGCAACATGCCCGGTATCAGCGGCTACGAATTGGCGCGCCGCATACGCGCC
GCCGAGGCCGCGCCCGGTTACGGACGTACGCGGTGCATTCTGTTCGGCTTCACGGCTTCGGCGCAgATGGACGAAGCGCA
GCGCTGCCGCGCCGCCGGCATGGACGACTGCCTGTTCAAGCCGATCGGCGTGGACGCCTTGCGGCAACGCCTGAACGAAG
CCGTGGCACGGGCCGCGCTCCCCACGCCCCCCTCGCCCCAGGCTGCCGCGCCGGCCACGGACGACGCCACCCCGACGGCG
TTCTCGGCCGAATCGATTCTTGCCTTGACGCAGAACGATGAGGCGCTGATCCGGCAATTGCTCGAAGAAGTGATCCGCAC
CAACCGGGCGGACGTCGACCAATTGCAAAAGCTGCACCAGCAGGCCGATTGGCCGAAGGTCTCGGACATGGCGCACAGGC
TGGCCGGCGGCGCGCGCGTGGTCGATGCCAAGGCCATGATAGACACTGCGCTGGCGCTGGAGAAAAAAGCGCAAGGCCAG
GCTGGCCCCTCACCCGAAATCGACGGCCTGGTACGTACGCTTGCGGCGCAGTCCGCCGCGCTGGAGACGCAACTGCGCGC
CTGGCTGGAGCAACGGCCGCATCAAGATCAGCCCTGA
>Bb_B0188.seq
ATGCCCGCCCCGCACCGCCTGTACCCCCGCAGTCTGATCTGCCTGGCTCAGGCGCTATTGGCATGGGCTTTGCTGGCATG
GGCGCCCGCGCAGGCAAGCCAGGAGCTGACCCTGGTCGGCAAGGCTGCCGTTCCCGACGTCGAGATCACGCTCGACGGCG
ACGACTGGCGCTGGCTGGCGCGCAAGCGGGTGCTGACGCTGGGCGTGTACGCGCCGGACATTCCCCCGTTCGACGTCACC
TATGACGAGCGCTACGAAGGCCTGACGGCCGACTACATGGCGATCATCGCGCACAACCTGGGCGTCCAGGCAAAAGTGCT
GCGCTACCCCACGCGCGAGCAAGCCGTCGGCGCACTGGAAAGCGGACAGATCGACCTCATCGGCACCGTCAATGGCATCG
AGGGCCGGCTGCAGAGCCTGCGCCTGAGTGTTCCCTACGCGGCCGACCACCCGGTGCTGGTCATGCCCATCGGCGCGCGC
CGCGCTCCGCCCGCGGACCTGGCAGGCCAGCGGTTGGCGGTTGACGCCAACTACTTGCCCAGGGAAACGCTGCAGCAGGC
CTATCCCCAGGCAACGCTGCATTACTTCCCATCATCCGAACAGGCGCTGGCCGCGGTGGCCTATGGACAAGCCGACGTGT
TCATCGGCGATGCGCTGACCACCTCGCACCTCGTTTCGCAAAGCTACTTCAACGACGTTCGCGTCGTCGCCCCGGCCCAG
ATCGTGACGGGCGGGGAATCCTTCGGCGTGCGCGCCGACAATACCCGCCTGCTGCGGGTGGTCAATGCCGTGCTCGAAGC
CATTCCGGCCTCCGAGCGCCGCAGCCTGATCTACCGCTGGGGCCTGGGCAGCAGCATTTCGCTCGATTTCGCGCGCCCCG
CCTATTCAGCGCGCGAGCAGCAGTGGATGGCAAACCATCCGGTCGTCAAGGTGGCGGTCCTGAACCTGTTCGCGCCCTTC
ACCCTGTTTCGCACCGATGAACAGTTCGGCGGCATCAGTGCCGCCGTGCTGCAACTGCTGCAGTTGCGCACCGGCCTGGA
TTTCCAGATCATCGGCGTCGACACGGTCGAGGAGCTGATCGCCAAGCTGCGCTCGGGCGAAGCCGACATGGCCGGCGCCC
TGTTCGTCAATGCCGCGCGGGAATCCGTCCTCAGCTTCAGCCGGCCGTATGTGCGCAATGGTCTGGTGATCGTCACGCGC
CAGGACCCCGCCGCGCCCGCCGACGCCGATCACCTCGACGGCCGCACGATTGCGATGGTGCGCAACAGCGCCGCCATCCC
GCTCCTGCAGCAGCGCTATCCCCAGGCGAAGGTCGTGACCGCCGACAACCCGACCGAAGCCATGCTGCTGGTGGCCGATG
GCCAGGCCGACGCCGTCGTGCAGACGCAGATCAGCGCCAGCTACTACGTCAACCGCTACTTCGCCGGAAAACTGCGCATT
GCCTCGGCGCTGGACCTGCCGCCGGCCGAGATCGCGCTGGCGACGGCGCGCGGCCAGACCGAGCTGATATCCATCCTGAA
CAAGGCGCTCTACAGCATTTCGAACGACGAACTCGCCTCCATCGTCAGCCGCTGGCGCGGCAGCGACGGCGATCCGCGCA
CCTGGTACGCCTACCGCAACGAAATCTACCTGCTGATCGGGCTGGGCCTGTTGTCGGCCCTGCTGTTCCTGAGCTGGATC
GTCTACCTGCGGCGCCAGATCCGCCAGCGCAAGCGGGCCGAGCGGGCGCTGAACGACCAGCTGGAATTCATGCGCGTGCT
CATCGACGGCACGCCCAACCCCATCTATGTGCGCGATAAGGAAGGCCGCATGCTGTTGTGCAATGACGCCTACCTCGACA
CCTTTGGCGTGACTGCCGATGCGGTACTGGGCAAGACCATCCCGGAGGCCAACGTGGTGGGCGACCCGGCGCTGGCTCGC
GAGATGCACGAGTTCCTGCTCACGCGCATGGCCGCCGAGCGCGAGCCGCGCTTCGAGGACCGCGATGTCACGCTGCACGG
CCGCACCCGCCATGTCTACCAGTGGACGGTTCCGTACGGCGACTCGCTGGGCGAACTCAAGGGCATCATCGGCGGCTGGA
TCGACATTACCGAACGCGCCGAGCTGCTGCGCGAGCTGCACGACGCCAAGGAAAGCGCCGACGCCGCCAACCGGGCCAAG
ACCACGTTCCTGGCAACGATGAGCCACGAGATCCGCACGCCGATGAACGCGATCATCGGCATGCTGGAGCTGGCGCTGCT
CCGTCCGGCCGACCAGGAGCCGGACCGCCAGTCCATCCAGGTCGCGTACGACTCGGCCCGCAGCCTGCTGGAGCTGATAG
GCGACATCCTGGACATTGCGAAGATCGAGGCGGGAAAATTCGACCTGGCGCCGGTGCGCACGGCGCTGCGCGCCCTGCCC
GAAGGGGCGATCCGCGTCTTCGACGGGTTGGCGCGCCAGAAAGGCATAGAGCTGGTATTGAAGACCGACATCGTGGGCGT
GGACGATGTATTGATAGACCCCTTGCGCATGAAGCAAGTGCTCTCGAACCTGGTGGGCAACGCCATCAAGTTCACCACCG
AAGGCCAGGTTGTCCTTACCGTGACCGCGCGCCCCGACGGCGAGGCCGCGCACGTGCAGTTCAGCGTGAGCGACACCGGC
TGCGGCATCAGCGAGGCCGACCAACGGCAGCTGTTCAAACCGTTCTCGCAGGTGGGCGGCAGCGCCGAGGCCGGGCCGGC
GCCGGGCACTGGCCTGGGCCTGTCCATCAGCCGCCGCCTCGTCGAATTGATGGGGGGAACGCTGGTCATGCGCAGCGCGC
CAGGGGTGGGCACAACGGTTTCGGTGGACCTGAGGCTGACCATGGTCGAAAAATCCGCGCAGGCCACGCCGCCCGCTGCG
GCCGCTCAGGCCACGCCATCCAAGCCGCAGGTATCGCTGCGCGTGCTGGTCGTCGATGACCACAAGCCCAACCTGATGCT
GCTGCGCCAGCAGCTGGACTACCTGGGCCAGCGTGTCGTCGCCGCCGACTCCGGCGAAGCCGCCCTGGCCCTGTGGCACG
AGCATGCGTTCGACGTCGTGATCACCGATTGCAACATGCCCGGTATCAACGGCTACGAATTGGCGCGCCGCATACGCGCC
GCCGAGGCCGCGCCCGGTTACGGACGTACGCGGTGCATTCTGTTCGGCTTCACGGCTTCGGCGCAGATGGACGAAGCGCA
GCGCTGCCGCGCCGCCGGCATGGACGACTGCCTGTTCAAGCCGATCGGCGTGGACGCCTTGCGGCAACGCTTGAACGAAG
CCGCGGCACGGGCCGCGCTCCCCACGCCCCCCTCGCCCCAGGCTGCCGCGCCGGCCACGCACGACGCCACCCCGGCGGCG
TTCTCGGCCGAGTCGATTCTTGCCCTGACGCAGAACGATGAGGCGCTGATCCGGCAATTGCTCGAAGAAGTGATTCGCAC
CAACCGGGCGGACGTCGATCAATTGCAGAAGCTGCACCAGCAGGCCGATTGGCCGAAGGTCTCGGACATGGCGCACAGGC
TGGCCGGCGGCGCGCGCGTGGTCGATGCCAAGGCCATGATAGACACTGCGCTGGCGCTGGAGAAAAAAGCGCAAGGCCAG
GCTGGCCCCTCGCCCGAAATCGACGGCATGGTACGTACGCTTGCGGCGCAGTCCGCCGCGCTGGAGACGCAACTACGCGC
CTGGCTGGAGCAACGGCCGCATCAAGGCCAGCCCTGA
>Bb_B0228.seq
ATGCCCGCCCCGCACCGCCTGTACCCCCGCAGTCTGATCTGCCTGGCTCAGGCGCTATTGGCATGGGCTTTGCTGGCATG
GGCGCCCGCGCAGGCAAGCCAGGAGCTGACCCTGGTCGGCAAGGCTGCCGTTCCCGACGTCGAGATCACGCTCGACGGCG
ACGACTGGCGCTGGCTGGCGCGCAAGCGGGTGCTGACGCTGGGCGTGTACGCGCCGGACATTCCCCCGTTCGACGTCACC
TATGACGAGCGCTACGAAGGCCTGACGGCCGACTACATGGCGATCATCGCGCACAACCTGGGCGTCCAGGCAAAAGTGCT
GCGCTACCCCACGCGCGAGCAAGCCGTCGGCGCACTGGAAAGCGGACAGATCGACCTCATCGGCACCGTCAATGGCATCG
AGGGCCGGCTGCAGAGCCTGCGCCTGAGTGTTCCCTACGCGGCCGACCACCCGGTGCTGGTCATGCCCATCGGCGCGCGC
CGCGCTCCGCCCGCGGACCTGGCAGGCCAGCGGTTGGCGGTTGACGCCAACTACTTGCCCAGGGAAACGCTGCAGCAGGC
CTATCCCCAGGCAACGCTGCATTACTTCCCATCATCCGAACAGGCGCTGGCCGCGGTGGCCTATGGACAAGCCGACGTGT
TCATCGGCGATGCGCTGACCACCTCGCACCTCGTTTCGCAAAGCTACTTCAACGACGTTCGCGTCGTCGCCCCGGCCCAG
ATCGTGACGGGCGGGGAATCCTTCGGCGTGCGCGCCGACAATACCCGCCTGCTGCGGGTGGTCAATGCCGTGCTCGAAGC
CATTCCGGCCTCCGAGCGCCGCAGCCTGATCTACCGCTGGGGCCTGGGCAGCAGCATTTCGCTCGATTTCGCGCGCCCCG
CCTATTCAGCGCGCGAGCAGCAGTGGATGGCAAACCATCCGGTCGTCAAGGTGGCGGTCCTGAACCTGTTCGCGCCCTTC
ACCCTGTTTCGCACCGATGAACAGTTCGGCGGCATCAGTGCCGCCGTGCTGCAACTGCTGCAGTTGCGCACCGGCCTGGA
TTTCCAGATCATCGGCGTCGACACGGTCGAGGAGCTGATCGCCAAGCTGCGCTCGGGCGAAGCCGACATGGCCGGCGCCC
TGTTCGTCAATGCCGCGCGGGAATCCGTCCTCAGCTTCAGCCGGCCGTATGTGCGCAATGGTCTGGTGATCGTCACGCGC
CAGGACCCCGCCGCGCCCGCCGACGCCGATCACCTCGACGGCCGCACGATTGCGATGGTGCGCAACAGCGCCGCCATCCC
GCTCCTGCAGCAGCGCTATCCCCAGGCGAAGGTCGTGACCGCCGACAACCCGACCGAAGCCATGCTGCTGGTGGCCGATG
GCCAGGCCGACGCCGTCGTGCAGACGCAGATCAGCGCCAGCTACTACGTCAACCGCTACTTCGCCGGAAAACTGCGCATT
GCCTCGGCGCTGGACCTGCCGCCGGCCGAGATCGCGCTGGCGACGGCGCGCGGCCAGACCGAGCTGATATCCATCCTGAA
CAAGGCGCTCTACAGCATTTCGAACGACGAACTCGCCTCCATCGTCAGCCGCTGGCGCGGCAGCGACGGCGATCCGCGCA
CCTGGTACGCCTACCGCAACGAAATCTACCTGCTGATCGGGCTGGGCCTGTTGTCGGCCCTGCTGTTCCTGAGCTGGATC
GTCTACCTGCGGCGCCAGATCCGCCAGCGCAAGCGGGCCGAGCGGGCGCTGAACGACCAGCTGGAATTCATGCGCGTGCT
CATCGACGGCACGCCCAACCCCATCTATGTGCGCGATAAGGAAGGCCGCATGCTGTTGTGCAATGACGCCTACCTCGACA
CCTTTGGCGTGACTGCCGATGCGGTACTGGGCAAGACCATCCCGGAGGCCAACGTGGTGGGCGACCCGGCGCTGGCTCGC
GAGATGCACGAGTTCCTGCTCACGCGCATGGCCGCCGAGCGCGAGCCGCGCTTCGAGGACCGCGATGTCACGCTGCACGG
CCGCACCCGCCATGTCTACCAGTGGACGGTTCCGTACGGCGACTCGCTGGGCGAACTCAAGGGCATCATCGGCGGCTGGA
TCGACATTACCGAACGCGCCGAGCTGCTGCGCGAGCTGCACGACGCCAAGGAAAGCGCCGACGCCGCCAACCGGGCCAAG
ACCACGTTCCTGGCAACGATGAGCCACGAGATCCGCACGCCGATGAACGCGATCATCGGCATGCTGGAGCTGGCGCTGCT
CCGTCCGGCCGACCAGGAGCCGGACCGCCAGTCCATCCAGGTCGCGTACGACTCGGCCCGCAGCCTGCTGGAgCTGATAG
GCGACATCCTGGACATTGCGAAGATCGAGGCGGGAAAATTCGACCTGGCGCCGGTGCGCACGGCGCTGCGCGCCCTGCCC
GAAGGGGCGATCCGCGTCTTCGACGGGTTGGCGCGCCAGAAAGGCATAGAGCTGGTATTGAAGACCGACATCGTGGGCGT
GGACGATGTATTGATAGACCCCTTGCGCATGAAGCAAGTGCTCTCGAACCTGGTGGGCAACGCCATCAAGTTCACCACCG
AAGGCCAGGTTGTCCTTACCGTGACCGCGCGCCCCGACGGCGAGGCCGCGCACGTGCAGTTCAGCGTGAGCGACACCGGC
TGCGGCATCAGCGAGGCCGACCAACGGCAGCTGTTCAAACCGTTCTCGCAGGTGGGCGGCAGCGCCGAGGCCGGGCCGGC
GCCGGGCACTGGCCTGGGCCTGTCCATCAGCCGCCGCCTCGTCGAATTGATGGGGGGAACGCTGGTCATGCGCAGCGCGC
CAGGGGTGGGCACAACGGTTTCGGTGGACCTGAGGCTGACCATGGTCGAAAAATCCGCGCAGGCCACGCCGCCCGCTGCG
GCCGCTCAGGCCACGCCATCCAAGCCGCAGGTATCGCTGCGCGTGCTGGTCGTCGATGACCACAAGCCCAACCTGATGCT
GCTGCGCCAGCAGCTGGACTACCTGGGCCAGCGTGTCGTCGCCGCCGACTCCGGCGAAGCCGCCCTGGCCCTGTGGCACG
AGCATGCGTTCGACGTCGTGATCACCGATTGCAACATGCCCGGTATCAACGGCTACGAATTGGCGCGCCGCATACGCGCC
GCCGAGGCCGCGCCCGGTTACGGACGTACGCGGTGCATTCTGTTCGGCTTCACGGCTTCGGCGCAGATGGACGAAGCGCA
GCGCTGCCGCGCCGCCGGCATGGACGACTGCCTGTTCAAGCCGATCGGCGTGGACGCCTTGCGGCAACGCTTGAACGAAG
CCGCGGCACGGGCCGCGCTCCCCACGCCCCCCTCGCCCCAGGCTGCCGCGCCGGCCACGCACGACGCCACCCCGGCGGCG
TTCTCGGCCGAGTCGATTCTTGCCCTGACGCAGAACGATGAGGCGCTGATCCGGCAATTGCTCGAAGAAGTGATTCGCAC
CAACCGGGCGGACGTCGATCAATTGCAGAAGCTGCACCAGCAGGCCGATTGGCCGAAGGTCTCGGACATGGCGCACAGGC
TGGCCGGCGGCGCGCGCGTGGTCGATGCCAAGGCCATGATAGACACTGCGCTGGCGCTGGAGAAAAAAGCGCAAGGCCAG
GCTGGCCCCTCGCCCGAAATCGACGGCATGGTACGTACGCTTGCGGCGCAGTCCGCCGCGCTGGAGACGCAACTACGCGC
CTGGCTGGAGCAACGGCCGCATCAAGGCCAGCCCTGA
>Bb_B0260.seq
ATGCCCGCCCCGCACCGCCTGTACCCCCGCAGTCTGATCTGCCTGGCTCAGGCGCTATTGGCATGGGCTTTGCTGGCATG
GGCGCCCGCGCAGGCAAGCCAGGAGCTGACCCTGGTCGGCAAGGCTGCCGTTCCCAACGTCGAGATCACGCTCGACGGCG
ACGACTGGCGCTGGCTGGCGCGCAAGCGGGTGCTGACGCTGGGCGTGTACGCGCCGGACATTCCCCCGTTCGACGTCACC
TATGACGAGCGCTACGAAGGCCTGACGGCCGACTACATGGCGATCATCGCGCACAACCTGGGCGTCCAGGCAAAAGTGCT
GCGCTACCCCACGCGCGAGCAAGCCGTCGGCGCACTGGAAAGCGGACAGATCGACCTCATCGGCACCGTCAATGGCATCG
AGGGCCGGCTGCAGAGCCTGCGCCTGAGTGTTCCCTACGCGGCCGACCACCCGGTGCTGGTCATGCCCATCGGCGCGCGC
CGCGCTCCGCCCGCGGACCTGGCAGGCCAGCGGTTGGCGGTTGACGCCAACTACTTGCCCAGGGAAACGCTGCAGCAGGC
CTATCCCCAGGCAACGCTGCATTACTTCCCATCATCCGAACAGGCGCTGGCCGCGGTGGCCTATGGACAAGCCGACGTGT
TCATCGGCGATGCGCTGACCACCTCGCACCTCGTTTCGCAAAGCTACTTCAACGACGTTCGCGTCGTCGCCCCGGCCCAG
ATCGTGACGGGCGGGGAATCCTTCGGCGTGCGCGCCGACAATACCCGCCTGCTGCGGGTGGTCAATGCCGTGCTCGAAGC
CATTCCGGCCTCCGAGCGCCGCAGCCTGATCTACCGCTGGGGCCTGGGCAGCAGCATTTCGCTCGATTTCGCGCGCCCCG
CCTATTCAGCGCGCGAGCAGCAGTGGATGGCAAACCATCCGGTCGTCAAGGTGGCGGTCCTGAACCTGTTCGCGCCCTTC
ACCCTGTTTCGCACCGATGAACAGTTCGGCGGCATCAGTGCCGCCGTGCTGCAACTGCTGCAGTTGCGCACCGGCCTGGA
TTTCCAGATCATCGGCGTCGACACGGTCGAGGAGCTGATCGCCAAGCTGCGCTCGGGCGAAGCCGACATGGCCGGCGCCC
TGTTCGTCAATGCCGCGCGGGAATCCGTCCTCAGCTTCAGCCGGCCGTATGTGCGCAATGGTCTGGTGATCGTCACGCGC
CAGGACCCCGCCGCGCCCGCCGACGCCGATCACCTCGACGGCCGCACGATTGCGATGGTGCGCAACAGCGCCGCCATCCC
GCTCCTGCAGCAGCGCTATCCCCAGGCGAAGGTCGTGACCGCCGACAACCCGACCGAAGCCATGCTGCTGGTGGCCGATG
GCCAGGCCGACGCCGTCGTGCAGACGCAGATCAGCGCCAGCTACTACGTCAACCGCTACTTCGCCGGAAAACTGCGCATT
GCCTCGGCGCTGGACCTGCCGCCGGCCGAGATCGCGCTGGCGACGGCGCGCGGCCAGACCGAGCTGATATCCATCCTGAA
CAAGGCGCTCTACAGCATTTCGAACGACGAACTCGCCTCCATCGTCAGCCGCTGGCGCGGCAGCGACGGCGATCCGCGCA
CCTGGTACGCCTACCGCAACGAAATCTACCTGCTGATCGGGCTGGGCCTGTTGTCGGCCCTGCTGTTCCTGAGCTGGATC
GTCTACCTGCGGCGCCAGATCCGCCAGCGCAAGCGGGCCGAGCGGGCGCTGAACGACCAGCTGGAATTCATGCGCGTGCT
CATCGACGGCACGCCCAACCCCATCTATGTGCGCGATAAGGAAGGCCGCATGCTGTTGTGCAATGACGCCTACCTCGACA
CCTTTGGCGTGACTGCCGATGCGGTACTGGGCAAGACCATCCCGGAGGCCAACGTGGTGGGCGACCCGGCGCTGGCTCGC
GAGATGCACGAGTTCCTGCTCACGCGCATGGCCGCCGAGCGCGAGCCGCGCTTCGAGGACCGCGATGTCACGCTGCACGG
CCGCACCCGCCATGTCTACCAGTGGACGGTTCCGTACGGCGACTCGCTGGGCGAACTCAAGGGCATCATCGGCGGCTGGA
TCGACATTACCGAACGCGCCGAGCTGCTGCGCGAGCTGCACGACGCCAAGGAAAGCGCCGACGCCGCCAACCGGGCCAAG
ACCACGTTCCTGGCAACGATGAGCCACGAGATCCGCACGCCGATGAACGCGATCATCGGCATGCTGGAGCTGGCGCTGCT
CCGTCCGGCCGACCAGGAGCCGGACCGCCAGTCCATCCAGGTCGCGTACGACTCGGCCCGCAGCCTGCTGGAGcTGATAG
GCGACATCCTGGACATTGCGAAGATCGAGGCGGGAAAATTCGACCTGGCGCCGGTGCGCACGGCGCTGCGCGCCCTGCCC
GAAGGGGCGATCCGCGTCTTCGACGGGTTGGCGCGCCAGAAAGGCATAGAGCTGGTATTGAAGACCGACATCGTGGGCGT
GGACGATGTATTGATAGACCCCTTGCGCATGAAGCAAGTGCTCTCGAACCTGGTGGGCAACGCCATCAAGTTCACCACCG
AAGGCCAGGTTGTCCTTACCGTGACCGCGCGCCCCGACGGCGAGGCCGCGCACGTGCAGTTCAGCGTGAGCGACACCGGC
TGCGGCATCAGCGAGGCCGACCAACGGCAGCTGTTCAAACCGTTCTCGCAGGTGGGCGGCAGCGCCGAGGCCGGGCCGGC
GCCGGGCACTGGCCTGGGCCTGTCCATCAGCCGCCGCCTCGTCGAATTGATGGGGGGAACGCTGGTCATGCGCAGCGCGC
CAGGGGTGGGCACAACGGTTTCGGTGGACCTGAGGCTGACCATGGTCGAAAAATCCGCGCAGGCCACGCCGCCCGCTGCG
GCCGCTCAGGCCACGCCATCCAAGCCGCAGGTATCGCTGCGCGTGCTGGTCGTCGATGACCACAAGCCCAACCTGATGCT
GCTGCGCCAGCAGCTGGACTACCTGGGCCAGCGTGTCGTCGCCGCCGACTCCGGCGAAGCCGCCCTGGCCCTGTGGCACG
AGCATGCGTTCGACGTCGTGATCACCGATTGCAACATGCCCGGTATCAACGGCTACGAATTGGCGCGCCGCATACGCGCC
GCCGAGGCCGCGCCCGGTTACGGACGTACGCGGTGCATTCTGTTCGGCTTCACGGCTTCGGCGCAGATGGACGAAGCGCA
GCGCTGCCGCGCCGCCGGCATGGACGACTGCCTGTTCAAGCCGATCGGCGTGGACGCCTTGCGGCAACGCTTGAACGAAG
CCGCGGCACGGGCCGCGCTCCCCACGCCCCCCTCGCCCCAGGCTGCCGCGCCGGCCACGCACGACGCCACCCCGGCGGCG
TTCTCGGCCGAGTCGATTCTTGCCCTGACGCAGAACGATGAGGCGCTGATCCGGCAATTGCTCGAAGAAGTGATTCGCAC
CAACCGGGCGGACGTCGATCAATTGCAGAAGCTGCACCAGCAGGCCGATTGGCCGAAGGTCTCGGACATGGCGCACAGGC
TGGCCGGCGGCGCGCGCGTGGTCGATGCCAAGGCCATGATAGACACTGCGCTGGCGCTGGAGAAAAAAGCGCAAGGCCAG
GCTGGCCCCTCGCCCGAAATCGACGGCATGGTACGTACGCTTGCGGCGCAGTCCGCCGCGCTGGAGACGCAACTACGCGC
CTGGCTGGAGCAACGGCCGCATCAAGGCCAGCCCTGA
>Bb_B0261.seq
ATGCCCGCCCCGCACCGCCTGTACCCCCGCAGTCTGATCTGCCTGGCTCAGGCGCTATTGGCATGGGCTTTGCTGGCATG
GGCGCCCGCGCAGGCAAGCCAGGAGCTGACCCTGGTCGGCAAGGCTGCCGTTCCCGACGTCGAGATCGCGCTCGACGGCG
ACGACTGGCGCTGGCTGGCGCGCAAGCGGGTGCTGACGCTGGGCGTGTACGCGCCGGACATTCCCCCGTTCGACGTCACC
TATGACGAGCGCTACGAAGGCCTGACGGCCGACTACATGGCGATCATCGCACACAACCTGGGCGTCCAGGCAAAAGTGCT
GCGCTACCCCACGCGCGAGCAAGCCGTCGGCGCACTGGAAAGCGGACAGATCGACCTCATCGGCACCGTCAATGGCATCG
AGGGCCGGCTGCAGAGCCTGCGCCTGAGTGTTCCCTACGCGGCCGACCACCCGGTGCTGGTCATGCCCATCGGCGCGCGC
CGCGCTCCGCCCGCGGACCTGGCAGGCCAGCGGTTGGCGGTTGACGCCAACTACTTGCCCAGGGAAACGCTGCAGCAGGC
CTATCCCCAGGCAACGCTGCATTACTTCCCATCGTCCGAACAGGCGCTGGCCGCGGTGGCCTATGGACAAGCCGACGTGT
TCATCGGCGATGCGCTGACCACCTCGCACCTCGTTTCGCAAAGCTACTTCAACGACGTTCGCGTCGTCGCCCCGGCCCAG
ATCGTGACGGGCGGGGAATCCTTCGGCGTGCGCGCCGACAATACCCGCCTGCTGCGGGTGGTCAATGCCGTACTCGAAGC
CATTCCGGCCTCCGAGCGCCGCAGCCTGATCTACCGCTGGGGCCTGGGCAGCAGCATTTCGCTCGATTTCGCGCGCCCCG
CCTATTCAGCGCGCGAGCAGCAGTGGATGGCAAACCATCCGGTCGTCAAGGTGGCGGTCCTGAACCTGTTCGCGCCCTTC
ACCCTGTTTCGCACCGATGAACAGTTCGGCGGCATCAGTGCCGCCGTGCTGCAACTGCTGCAGTTGCGCACCGGCCTGGA
TTTCCAGATCATCGGCGTCGACACGGTCGAGGAGCTGATCGCCAAGCTGCGCTCGGGCGAAGCCGACATGGCCGGCGCCC
TGTTCGTCAATGCCGCGCGGGAATCCGTCCTCAGCTTCAGCCGGCCGTATGTGCGCAATGGCATGGTGATCGTCACGCGC
CAGGACCCCGCCGCGCCCGCCGACGCCGATCACCTCGACGGCCGCACGATTGCGATGGTGCGCAACAGCGCCGCCATCCC
GCTCCTGCAGCAGCGCTATCCCCAGGCGAAGGTCGTGACCGCCGACAACCCGACCGAAGCCATGCTGCTGGTGGCCGATG
GCCAGGCCGACGCCGTCGTGCAGACGCAGATCAGCGCCAGCTACTACGTCAACCGCTACTTCGCCGGAAAACTGCGCATT
GCCTCGGCGCTGGACCTGCCGCCGGCCGAGATCGCGCTGGCGACGGCGCGCGGCCAGACCGAGCTGATATCCATCCTGAA
CAAGGCGCTCTACAGCATTTCGAACGACGAACTCGCCTCCATCGTCAGCCGCTGGCGCGGCAGCGACGGCGATCCGCGCA
CCTGGTACGCCTACCGCAACGAGATCTACCTGCTGATCGGGCTGGGCCTGTTGTCGGCCCTGCTGTTCCTGAGCTGGATC
GTCTACCTGCGGCGCCAGATCCGCCAGCGCAAGCGGGCCGAGCGGGCGCTGAACGACCAGCTGGAATTCATGCGCGTGCT
CATCGACGGCACGCCCAACCCCATCTATGTGCGCGATAAGGAAGGCCGCATGCTGTTGTGCAATGACGCCTACCTCGACA
CCTTTGGCGTGACTGCCGATGCGGTACTGGGCAAGACCATCCCGGAGGCCAACGTGGTGGGCGACCCGGCGCTGGCTCGC
GAGATGCACGAGTTCCTGCTCACGCGCATGTCCGCCGAGCGCGAGCCGCGCTTCGAGGACCGCGATGTCACGCTGCACGG
CCGCACCCGCCATGTCTACCAGTGGACGGTTCCGTACGGCGACTCGCTGGGCGAACTCAAGGGCATCATCGGCGGCTGGA
TCGACATTACCGAACGCGCCGAGCTGCTGCGCGAGCTGCACGACGCCAAGGAAAGCGCCGACGCCGCCAACCGGGCCAAG
ACCACGTTCCTGGCAACGATGAGCCACGAGATCCGCACGCCGATGAACGCGATCATCGGCATGCTGGAGCTGGCGCTGCT
CCGTCCGGCCGACCAGGAGCCGGACCGCCAGTCCATCCAGGTCGCGTACGACTCGGCCCGCAGCCTGCTGGAGCTGATAG
GCGACATCCTGGACATTGCGAAGATCGAGGCGGGAAAATTCGACCTGGCGCCGGTGCGCACGGCGCTGCGCGCCCTACCC
GAAGGGGCGATCCGCGTCTTCGACGGGTTGGCGCGCCAGAAAGGCATAGAGCTGGTATTGAAGACCGACATCGTGGGCGT
GGACGATGTATTGATAGACCCCTTGCGCATGAAGCAAGTGCTCTCGAACCTGGTGGGCAACGCCATCAAGTTCACCACCG
AAGGCCAGGTTGTCCTTACCGTGACCGCGCGCCCCGACGGCGAGGCCGCGCACGTGCAGTTCAGCGTGAGCGACACCGGC
TGCGGCATCAGCGAGGCCGACCAACGGCAGCTGTTCAAACCGTTCTCGCAGGTGGGCGGCAGCGCCGAGGCCGGGCCGGC
GCCGGGCACTGGCCTGGGCCTGTCCATCAGCCGCCGCCTCGTCGAATTGATGGGGGGAACGCTGGTCATGCGCAGCGCGC
CAGGGGTGGGCACAACGGTTTCGGTGGACCTGAGGCTGACCATGGTCGAAAAATCCGCGCAGGCCACGCCGCCCGCTGCG
GCCGCTCAGGCCACGCCATCCAAGCCGCAGGTATCGCTGCGCGTGCTGGTCGTCGATGACCACAAGCCCAACCTGATGCT
GCTGCGCCAGCAGCTGGACTACCTGGGCCAGCGTGTCGTCGCCGCCGACTCCGGCGAAGCCGCCCTGGCCCTGTGGCACG
AGCATGCGTTCGACGTCGTGATCACCGATTGCAACATGCCCGGTATCAACGGCTACGAATtGGCGCGCCGCATACGCGCC
GCCGAGGCCGCGCCCGGTTACGGACGTACGCGGTGCATTCTGTTCGGCTTCACGGCTTCGGCGCAGATGGACGAAGCGCA
GCGCTGCCGCGCCGCCGGCATGGACGACTGCCTGTTCAAGCCGATCGGCGTGGACGCCTTGCGGCAACGCTTGAACGAAG
CCGCGGCACGGGCCGCGCTCCCCACGCCCCCCTCGCCCCAGGCTGCCGCGCCGGCCACGCACGACGCCACCCCGGCGGCG
TTCTCGGCCGAGTCGATTCTTGCCCTGACGCAGAACGATGAGGCGCTCATCCGGCAATTGCTCGAAGAACTGATTCGCAC
CAACCGGGCGGACGTCGATCAATTGCAGAAGCTGCACCAGCAGGCCGATTGGCCGAAGGTCTCGGACATGGCGCACAGGC
TGGCCGGCGGCGCGCGCGTGGTCGATGCCAAGGCCATGATAGACACTGCGCTGGCGCTGGAAAAAAAAGCGCAAGGCCAG
GCTGGCCCCTCGCCCGAAATCGACGGCATGGTACGTACGCTTGCGGCGCAGTCCGCCGCGCTGGAGACGCAACTACGCGC
CTGGCTGGAGCAACGGCCGCATCAAGGCCAGCCCTGA
>Bb_B0505.seq
ATGCCCGCCCCGCACCGCCTGTACCCCCGCAGTCTGATCTGCCTGGCTCAGGCGCTATTGGCATGGGCTTTGCTGGCATG
GGCGCCCGCGCAGGCAAGCCAGGAGCTGACCCTGGTCGGCAAGGCTGCCGTTCCCGACGTCGAGATCGCGCTCGACGGCG
ACGACTGGCGCTGGCTGGCGCGCAAGCGGGTGCTGACGCTGGGCGTGTACGCGCCGGACATTCCCCCGTTCGACGTCACC
TATGACGAGCGCTACGAAGGCCTGACGGCCGACTACATGGCGATCATCGCACACAACCTGGGCGTCCAGGCAAAAGTGCT
GCGCTACCCCACGCGCGAGCAAGCCGTCGGCGCACTGGAAAGCGGACAGATCGACCTCATCGGCACCGTCAATGGCATCG
AGGGCCGGCTGCAGAGCCTGCGCCTGAGTGTTCCCTACGCGGCCGACCACCCGGTGCTGGTCATGCCCATCGGCGCGCGC
CGCGCTCCGCCCGCGGACCTGGCAGGCCAGCGGTTGGCGGTTGACGCCAACTACTTGCCCAGGGAAACGCTGCAGCAGGC
CTATCCCCAGGCAACGCTGCATTACTTCCCATCGTCCGAACAGGCGCTGGCCGCGGTGGCCTATGGACAAGCCGACGTGT
TCATCGGCGATGCGCTGACCACCTCGCACCTCGTTTCGCAaAGCTACTTCAACGACGTTCGCGTCGTCGCCCCGGCCCAG
ATCGTGACGGGCGGGGAATCCTTCGGCGTGCGCGCCGACAATACCCGCCTGCTGCGGGTGGTCAATGCCGTACTCGAAGC
CATTCCGGCCTCCGAGCGCCGCAGCCTGATCTACCGCTGGGGCCTGGGCAGCAGCATTTCGCTCGATTTCGCGCGCCCCG
CCTATTCAGCGCGCGAGCAGCAGTGGATGGCAAACCATCCGGTCGTCAAGGTGGCGGTCCTGAACCTGTTCGCGCCCTTC
ACCCTGTTTCGCACCGATGAACAGTTCGGCGGCATCAGTGCCGCCGTGCTGCAACTGCTGCAGTTGCGCACCGGCCTGGA
TTTCCAGATCATCGGCGTCGACACGGTCGAGGAGCTGATCGCCAAGCTGCGCTCGGGCGAAGCCGACATGGCCGGCGCCC
TGTTCGTCAATGCCGCGCGGGAATCCGTCCTCAGCTTCAGCCGGCCGTATGTGCGCAATGGCATGGTGATCGTCACGCGC
CAGGACCCCGCCGCGCCCGCCGACGCCGATCACCTCGACGGCCGCACGATTGCGATGGTGCGCAACAGCGCCGCCATCCC
GCTCCTGCAGCAGCGCTATCCCCAGGCGAAGGTCGTGACCGCCGACAACCCGACCGAAGCCATGCTGCTGGTGGCCGATG
GCCAGGCCGACGCCGTCGTGCAGACGCAGATCAGCGCCAGCTACTACGTCAACCGCTACTTCGCCGGAAAACTGCGCATT
GCCTCGGCGCTGGACCTGCCGCCGGCCGAGATCGCGCTGGCGACGGCGCGCGGCCAGACCGAGCTGATATCCATCCTGAA
CAAGGCGCTCTACAGCATTTCGAACGACGAACTCGCCTCCATCGTCAGCCGCTGGCGCGGCAGCGACGGCGATCCGCGCA
CCTGGTACGCCTACCGCAACGAGATCTACCTGCTGATCGGGCTGGGCCTGTTGTCGGCCCTGCTGTTCCTGAGCTGGATC
GTCTACCTGCGGCGCCaGATCCGCCAGCGCAAGCGGGCCGAGCGGGCGCTGAACGACCAGCTGGAATTCATGCGCGTGCT
CATCGACGGCACGCCCAACCCCATCTATGTGCGCGATAAGGAAGGCCGCATGCTGTTGTGCAATGACGCCTACCTCGACA
CCTTTGGCGTGACTGCCGATGCGGTACTGGGCAAGACCATCCCGGAGGCCAACGTGGTGGGCGACCCGGCGCTGGCTCGC
GAGATGCACGAGTTCCTGCTCACGCGCATGTCCGCCGAGCGCGAGCCGCGCTTCGAGGACCGCGATGTCACGCTGCACGG
CCGCACCCGCCATGTCTACCAGTGGACGGTTCCGTACGGCGACTCGCTGGGCGAACTCAAGGGCATCATCGGCGGCTGGA
TCGACATTACCGAACGCGCCGAGCTGCTGCGCGAGCTGCACGACGCCAAGGAAAGCGCCGACGCCGCCAACCGGGCCAAG
ACCACGTTCCTGGCAACGATGAGCCACGAGATCCGCACGCCGATGAACGCGATCATCGGCATGCTGGAGCTGGCGCTGCT
CCGTCCGGCCGACCAGGAGCCGGACCGCCAGTCCATCCAGGTCGCGTACGACTCGGCCCGCAGCCTGCTGGAGCTGATAG
GCGACATCCTGGACATTGCGAAGATCGAGGCGGGAAAATTCGACCTGGCGCCGGTGCGCACGGCGCTGCGCGCCCTACCC
GAAGGGGCGATCCGCGTCTTCGACGGGTTGGCGCGCCAGAAAGGCATAGAGCTGGTATTGAAGACCGACATCGTGGGCGT
GGACGATGTATTGATAGACCCCTTGCGCATGAAGCAAGTGCTCTCGAACCTGGTGGGCAACGCCATCAAGTTCACCACCG
AAGGCCAGGTTGTCCTTACCGTGACCGCGCGCCCCGACGGCGAGGCCGCGCACGTGCAGTTCAGCGTGAGCGACACCGGC
TGCGGCATCAGCGAGGCCGACCAACGGCAGCTGTTCAAACCGTTCTCGCAGGTGGGCGGCAGCGCCGAGGCCGGGCCGGC
GCCGGGCACTGGCCTGGGCCTGTCCATCAGCCGCCGCCTCGTCGAATTGATGGGGGGAACGCTGGTCATGCGCAGCGCGC
CAGGGGTGGGCACAACGGTTTCGGTGGACCTGAGGCTGACCATGGTCGAAAAATCCGCGCAGGCCACGCCGCCCGCTGCG
GCCGCTCAGGCCACGCCATCCAAGCCGCAGGTATCGCTGCGCGTGCTGGTCGTCGATGACCACAAGCCCAACCTGATGCT
GCTGCGCCAGCAGCTGGACTACCTGGGCCAGCGTGTCGTCGCCGCCGACTCCGGCGAAGCCGCCCTGGCCCTGTGGCACG
AGCATGCGTTCGACGTCGTGATCACCGATTGCAACATGCCCGGTATCAACGGCTACGAATtGGCGCGCCGCATACGCGCC
GCCGAGGCCGCGCCCGGTTACGGACGTACGCGGTGCATTCTGTTCGGCTTCACGGCTTCGGCGCAGATGGACGAAGCGCA
GCGCTGCCGCGCCGCCGGCATGGACGACTGCCTGTTCAAGCCGATCGGCGTGGACGCCTTGCGGCAACGCTTGAACGAAG
CCGCGGCACGGGCCGCGCTCCCCACGCCCCCCTCGCCCCAGGCTGCCGCGCCGGCCACGCACGACGCCACCCCGGCGGCG
TTCTCGGCCGAGTCGATTCTTGCCCTGACGCAGAACGATGAGGCGCTCATCCGGCAATTGCTCGAAGAACTGATTCGCAC
CAACCGGGCGGACGTCGATCAATTGCAGAAGCTGCACCAGCAGGCCGATTGGCCGAAGGTCTCGGACATGGCGCACAGGC
TGGCCGGCGGCGCGCGCGTGGTCGATGCCAAGGCCATGATAGACACTGCGCTGGCGCTGGAAAAAAAAGCGCAAGGCCAG
GCTGGCCCCTCGCCCGAAATCGACGGCATGGTACGTACGCTTGCGGCGCAGTCCGCCGCGCTGGAGACGCAACTACGCGC
CTGGCTGGAGCAACGGCCGCATCAAGGCCAGCCCTGA
>Bb_B1965.seq
ATGCCCGCCCCGCACCGCCTGTACCCCCGCAGTCTGATCTGCCTGGCTCAGGCGCTATTGGCATGGGCTTTGCTGGCATG
GGCGCCCGCGCAGGCAAGCCAGGAGCTGACCCTGGTCGGCAAGGCTGCCGTTCCCGACGTCGAGATCACGCTCGACGGCG
ACGACTGGCGCTGGCTGGCGCGCAAGCGGGTGCTGACGCTGGGCGTGTACGCGCCGGACATTCCCCCGTTCGACGTCACC
TATGACGAGCGCTACGAAGGCCTGACGGCCGACTACATGGCGATCATCGCGCACAACCTGGGCGTCCAGGCAAAAGTGCT
GCGCTACCCCACGCGCGAGCAAGCCGTCGGCGCACTGGAAAGCGGACAGATCGACCTCATCGGCACCGTCAATGGCATCG
AGGGCCGGCTGCAGAGCCTGCGCCTGAGTGTTCCCTACGCGGCCGACCACCCGGTGCTGGTCATGCCCATCGGCGCGCGC
CGCGCTCCGCCCGCGGACCTGGCAGGCCAGCGGTTGGCGGTTGACGCCAACTACTTGCCCAGGGAAACGCTGCAGCAGGC
CTATCCCCAGGCAACGCTGCATTACTTCCCATCATCCGAACAGGCGCTGGCCGCGGTGGCCTATGGACAAGCCGACGTGT
TCATCGGCGATGCGCTGACCACCTCGCACCTCGTTTCGCAAAGCTACTTCAACGACGTTCGCGTCGTCGCCCCGGCCCAG
ATCGTGACGGGCGGGGAATCCTTCGGCGTGCGCGCCGACAATACCCGCCTGCTGCGGGTGGTCAATGCCGTGCTCGAAGC
CATTCCGGCCTCCGAGCGCCGCAGCCTGATCTACCGCTGGGGCCTGGGCAGCAGCATTTCGCTCGATTTCGCGCGCCCCG
CCTATTCAGCGCGCGAGCAGCAGTGGATGGCAAACCATCCGGTCGTCAAGGTGGCGGTCCTGAACCTGTTCGCGCCCTTC
ACCCTGTTTCGCACCGATGAACAGTTCGGCGGCATCAGTGCCGCCGTGCTGCAACTGCTGCAGTTGCGCACCGGCCTGGA
TTTCCAGATCATCGGCGTCGACACGGTCGAGGAGCTGATCGCCAAGCTGCGCTCGGGCGAAGCCGACATGGCCGGCGCCC
TGTTCGTCAATGCCGCGCGGGAATCCGTCCTCAGCTTCAGCCGGCCGTATGTGCGCAATGGTCTGGTGATCGTCACGCGC
CAGGACCCCGCCGCGCCCGCCGACGCCGATCACCTCGACGGCCGCACGATTGCGATGGTGCGCAACAGCGCCGCCATCCC
GCTCCTGCAGCAGCGCTATCCCCAGGCGAAGGTCGTGACCGCCGACAACCCGACCGAAGCCATGCTGCTGGTGGCCGATG
GCCAGGCCGACGCCGTCGTGCAGACGCAGATCAGCGCCAGCTACTACGTCAACCGCTACTTCGCCGGAAAACTGCGCATT
GCCTCGGCGCTGGACCTGCCGCCGGCCGAGATCGCGCTGGCGACGGCGCGCGGCCAGACCGAGCTGATATCCATCCTGAA
CAAGGCGCTCTACAGCATTTCGAACGACGAACTCGCCTCCATCGTCAGCCGCTGGCGCGGCAGCGACGGCGATCCGCGCA
CCTGGTACGCCTACCGCAACGAAATCTACCTGCTGATCGGGCTGGGCCTGTTGTCGGCCCTGCTGTTCCTGAGCTGGATC
GTCTACCTGCGGCGCCAGATCCGCCAGCGCAAGCGGGCCGAGCGGGCGCTGAACGACCAGCTGGAATTCATGCGCGTGCT
CATCGACGGCACGCCCAACCCCATCTATGTGCGCGATAAGGAAGGCCGCATGCTGTTGTGCAATGACGCCTACCTCGACA
CCTTTGGCGTGACTGCCGATGCGGTACTGGGCAAGACCATCCCGGAGGCCAACGTGGTGGGCGACCCGGCGCTGGCTCGC
GAGATGCACGAGTTCCTGCTCACGCGCATGGCCGCCGAGCGCGAGCCGCGCTTCGAGGACCGCGATGTCACGCTGCACGG
CCGCACCCGCCATGTCTACCAGTGGACGGTTCCGTACGGCGACTCGCTGGGCGAACTCAAGGGCATCATCGGCGGCTGGA
TCGACATTACCGAACGCGCCGAGCTGCTGCGCGAGCTGCACGACGCCAAGGAAAGCGCCGACGCCGCCAACCGGGCCAAG
ACCACGTTCCTGGCAACGATGAGCCACGAGATCCGCACGCCGATGaACGCGATCATCGGCATGCTGGAGCTGGCGCTGCT
CCGTCCGGCCGACCAGGAGCCGGACCGCCAGTCCATCCAGGTCGCGTACGACTCGGCCCGCAGCCTGCTGGAGCTGATAG
GCGACATCCTGGACATTGCGAAGATCGAGGCGGGAAAATTCGACCTGGCGCCGGTGCGCACGGCGCTGCGCGCCCTGCCC
GAAGGGGCGATCCGCGTCTTCGACGGGTTGGCGCGCCAGAAAGGCATAGAGCTGGTATTGAAGACCGACATCGTGGGCGT
GGACGATGTATTGATAGACCCCTTGCGCATGAAGCAAGTGCTCTCGAACCTGGTGGGCAACGCCATCAAGTTCACCACCG
AAGGCCAGGTTGTCCTTACCGTGACCGCGCGCCCCGACGGCGAGGCCGCGCACGTGCAGTTCAGCGTGAGCGACACCGGC
TGCGGCATCAGCGAGGCCGACCAACGGCAGCTGTTCAAACCGTTCTCGCAGGTGGGCGGCAGCGCCGAGGCCGGGCCGGC
GCCGGGCACTGGCCTGGGCCTGTCCATCAGCCGCCGCCTCGTCGAATTGATGGGGGGAACGCTGGTCATGCGCAGCGCGC
CAGGGGTGGGCACAACGGTTTCGGTGGACCTGAGGCTGACCATGGTCGAAAAATCCGCGCAGGCCACGCCGCCCGCTGCG
GCCGCTCAGGCCACGCCATCCAAGCCGCAGGTATCGCTGCGCGTGCTGGTCGTCGATGACCACAAGCCCAACCTGATGCT
GCTGCGCCAGCAGCTGGACTACCTGGGCCAGCGTGTCGTCGCCGCCGACTCCGGCGAAGCCGCCCTGGCCCTGTGGCACG
AGCATGCGTTCGACGTCGTGATCACCGATTGCAACATGCCCGGTATCAACGGCTACGAATTGGCGCGCCGCATACGCGCC
GCCGAGGCCGCGCCCGGTTACGGACGTACGCGGTGCATTCTGTTCGGCTTCACGGCTTCGGCGCAGATGGACGAAGCGCA
GCGCTGCCGCGCCGCCGGCATGGACGACTGCCTGTTCAAGCCGATCGGCGTGGACGCCTTGCGGCAACGCTTGAACGAAG
CCGCGGCACGGGCCGCGCTCCCCACGCCCCCCTCGCCCCAGGCTGCCGCGCCGGCCACGCACGACGCCACCCCGGCGGCG
TTCTCGGCCGAGTCGATTCTTGCCCTGACGCAGAACGATGAGGCGCTGATCCGGCAATTGCTCGAAGAAGTGATTCGCAC
CAACCGGGCGGACGTCGATCAATTGCAGAAGCTGCACCAGCAGGCCGATTGGCCGAAGGTCTCGGACATGGCGCACAGGC
TGGCCGGCGGCGCGCGCGTGGTCGATGCCAAGGCCATGATAGACACTGCGCTGGCGCTGGAGAAAAAAGCGCAAGGCCAG
GCTGGCCCCTCGCCCGAAATCGACGGCATGGTACGTACGCTTGCGGCGCAGTCCGCCGCGCTGGAGACGCAACTACGCGC
CTGGCTGGAGCAACGGCCGCATCAAGGCCAGCCCTGA
>Bb_B1969.seq
ATGCCCGCCCCGCACCGCCTGTACCCCCGCAGTCTGATCTGCCTGGCTCAGGCGCTATTGGCATGGGCTTTGCTGGCATG
GGCGCCCGCGCAGGCAAGCCAGGAGCTGACCCTGGTCGGCAAGGCTGCCGTTCCCGACGTCGAGGTCGCGCTCGACGGCG
ACGACTGGCGTTGGCTGGCCCGCAAGCGGGTACTGACGCTGGGTGTGTACGCACCGGACATTCCTCCGTTCGACGTCACC
TATGGCGAACGCTACGAAGGCCTGACGGCCGACTACATGGCGATCATCGCGCACAACCTGGGGATGCAGGCGAAAGTGCT
GCGCTACCCCACGCGCGAACAAGCCCTCAGCGCGCTGGAAAGCGGGCAGATCGACCTCATCGGCACCGTCAATGGCACCG
ACGGCCGGCAACAGAGCCTGCGTCTGAGCGTTCCCTACGCCGCCGACCACCCGGTGATCGTCATGCCCATCGGCGCACGC
CACGTTCCAGCCTCGAACCTGGCCGGCCAGCGGCTGGCGGTCGACATCAACTACCTGCCCAAGGAAACGCTCGCACGGGC
CTACCCGCAGGCGACGCTGCATTACTTTCCCTCATCCGAGCAGGCGCTGGCCGCGGTGGCCTATGGGCAGGCCGACGTAT
TCATCGGCGATGCCCTGACCACCTCGCACCTCGTATCGCAAAGCTATTTCAATGACGTTCGCGCGGTCGCCCCGGCCCAT
ATCGCGACGGGCGGAGAATCCTTCGGCGTGCGCGCCGACAACACCCGCCTGCTGCGGGTGGTCAACGCCGTACTCGAAGC
CATTCCGCCTTCCGAACACCGCAGCCTGATCTACCGCTGGGGACTGGGCAGCAGCATTTCGCTCGATTTCGCGCACCCCG
CGTATTCCGCGCGCGAGCAGCAATGGATGGCAGACCACCCCGTCGTCAAGGTGGCGGTCCTGAATCTGTTCGCGCCCTTC
ACCCTGTTCCGCACCGACGAACAGTTCGGCGGGATCAGCGCCGCCGTGCTGCAGCTGCTGCAATTGCGCACCGGCCTGGA
CTTCGAGATCATCGGCGTCGACACGGTCGAGGAACTGATAGCCAAGCTGCGTTCGGGCGAAGCCGACATGGCCGGCGCCC
TGTTCGTCAACAGCGCGCGGGAGTCCTTCCTCAGTTTCAGCCGGCCGTATGTGCGCAATGGCATGGTGATCGTCACGCGC
CAGGACCCCGACGCGCCCGTCGACGCCGATCATCTGGACGGCCGCACGGTCGCGTTGGTGCGCAACAGCGCCGCCATTCC
TCTGCTGCAGCGGCGCTACCCCCAGGCGAAGGTGGTGACCGCCGACAACCCGAGCGAGGCGATGCTGATGGTGGCCAATG
GACAGGCCGACGCCGTCGTGCAGACGCAGATCAGCGCCAGCTATTACGTCAACCGCTACTTCGCCGGCAAGCTGCGCATC
GCCTCGGCGCTGGACCTGCCCCCGGCCGAGATCGCGCTGGCGACGACGCGCGGCCAGACCGAACTGATGTCCATCCTGAA
CAAGGCGCTCTACAGCATTTCGAACGACGAGCTCGCCTCCATCATCAGCCGCTGGCGCGGCAGCGACGGCGATCCGCGCA
CCTGGTACGCCTACCGCAACGAGATCTACCTGCTGATCGGGCTGGGCCTGTTGTCGGCCCTGCTGTTCCTGAGCTGGATC
GTCTACCTGCGGCGCCaGATCCGCCAGCGCAAGCGGGCCGAGCGGGCGCTGAACGACCAGCTGGAATTCATGCGCGTGCT
CATCGACGGCACGCCCAACCCCATCTATGTGCGCGATAAGGAAGGCCGCATGCTGTTGTGCAATGACGCCTACCTCGACA
CCTTTGGCGTGACTGCCGATGCGGTACTGGGCAAGACCATTCCGGAAGCCAACGTGGTGGGCGACCCGGCGCTGGCCCGC
GAAATGCACGAGTTCCTGCTCACGCGCGTGGCCGCCGAGCGCGAGCCGCGCTTCGAGGACCGCGATGTCACGCTGCACGG
CCGCACCCGCCATGTCTACCAGTGGACGATTCCGTACGGCGACTCGCTGGGCGAACTCAAGGGCATCATCGGCGGCTGGA
TCGACATCACCGAACGCGCCGAGCTGCTGCGCGAGCTGCACGACGCCAAGGAAAGCGCCGACGCCGCCAACCGGGCCAAG
ACCACGTTCCTGGCAACGATGAGCCACGAGATCCGCACGCCGATGaACGCGATCATCGGCATGCTGGAGCTGGCGCTGCT
CCGTCCGGCCGACCAGGAGCCGGATCGCCAGTCCATCCAGGTCGCGTACGACTCGGCCCGCAGCCTGCTGGAGCTGATAG
GCGACATCCTGGACATTGCGAAGATCGAGGCAGGAAAATTCGACCTGGCGCCGGTGCGCACGGCGCTGCGCGCCCTGCCC
GAAGGGGCGATCCGCGTCTTCGACGGATTGGCGCGCCAAAAAGGCATAGAGCTGGTATTGAAGACCGACATCGTGGGCGT
CGACGATGTATTGATAGACCCCTTGCGCATGAAGCAAGTGCTCTCGAACCTGGTGGGTAACGCCATCAAGTTCACCACCG
AAGGCCAGGTTGTCCTTGCCGTGACCGCACGCCCCGACGGCGAGGCCGCGCACGTGCAGTTCAGCGTGAGCGACACCGGC
TGCGGCATCAGCGAGGCCGACCAACGGCAGCTGTTCAAACCGTTCTCGCAGGTGGGTGGCAGCGCCGAGGCCGGGCCGGC
GCCGGGCACCGGCCTGGGCCTGTCCATCAGCCGCCGCCTCGTCGAATTGATGGGGGGAACGCTGGTCATGCGCAGCGCGC
CAGGGGTGGGCACAACGGTTTCGGTGGACCTGAGGCTGACCATGGTCGAAAAATCCGTGCAGGCCACGCCGCCCGCTGCG
GCCGCTGCGGCCACGCCGTCCAAGCCGCAGGTATCGCTGCGCGTGCTGGTCGTCGATGACCACAAACCCAACCTGATGCT
GCTGCGCCAGCAGCTGGACTACCTGGGCCAGCGTGTCATCGCCGCCGACTCCGGCGAAGCCGCCCTGGCCCTGTGGCGCG
AGCATGCGTTCGACGTCGTGATCACCGATTGCAACATGCCCGGTATCAGCGGCTACGAATtGGCGCGCCGCATACGCGCC
GCCGAGGCCGCGCCCGGTTACGGACGTACGCGGTGCATTCTGTTCGGCTTCACGGCTTCGGCGCAGATGGACGAAGCGCA
GCGCTGCCGCGCCGCCGGCATGGACGACTGCCTGTTCAAGCCGATCGGCGTGGACGCCTTGCGGCAACGCCTGAACGAAG
CCGTGGCACGGGCCGCGCTCCCCACGCCCCCCTCGCCCCAGGCTGCCGCGCCGGCCACGGACGACGCCACCCCGACGGCG
TTCTCGGCCGAATCGATTCTTGCCTTGACGCAGAACGATGAGGCGCTGATCCGGCAATTGCTCGAAGAAGTGATCCGCAC
CAACCGGGCGGACGTCGACCAATTGCAAAAGCTGCACCAGCAGGCCGATTGGCCGAAGGTCTCGGACATGGCGCACAGGC
TGGCCGGCGGCGCGCGCGTGGTCGATGCCAAGGCCATGATAGACACTGCGCTGGCGCTGGAGAAAAAAGCGCAAGGCCAG
GCTGGCCCCTCACCCGAAATCGACGGCCTGGTACGTACGCTTGCGGCGCAGTCCGCCGCGCTGGAGACGCAACTGCGCGC
CTGGCTGGAGCAACGGCCGCATCAAGATCAGCCCTGA
>Bb_B1973.seq
ATGCCCGCCCCGCACCGCCTGTACCCCCGCAGTCTGATCTGCCTGGCTCAGGCGCTATTGGCATGGGCTTTGCTGGCATG
GGCGCCCGCGCAGGCAAGCCAGGAGCTGACCCTGGTCGGCAAGGCTGCCGTTCCCGACGTCGAGATCACGCTCGACGGCG
ACGACTGGCGCTGGCTGGCGCGCAAGCGGGTGCTGACGCTGGGCGTGTACGCGCCGGACATTCCCCCGTTCGACGTCACC
TATGACGAGCGCTACGAAGGCCTGACGGCCGACTACATGGCGATCATCGCGCACAACCTGGGCGTCCAGGCAAAAGTGCT
GCGCTACCCCACGCGCGAGCAAGCCGTCGGCGCACTGGAAAGCGGACAGATCGACCTCATCGGCACCGTCAATGGCATCG
AGGGCCGGCTGCAGAGCCTGCGCCTGAGTGTTCCCTACGCGGCCGACCACCCGGTGCTGGTCATGCCCATCGGCGCGCGC
CGCGCTCCGCCCGCGGACCTGGCAGGCCAGCGGTTGGCGGTTGACGCCAACTACTTGCCCAGGGAAACGCTGCAGCAGGC
CTATCCCCAGGCAACGCTGCATTACTTCCCATCATCCGAACAGGCGCTGGCCGCGGTGGCCTATGGACAAGCCGACGTGT
TCATCGGCGATGCGCTGACCACCTCGCACCTCGTTTCGCAAAGCTACTTCAACGACGTTCGCGTCGTCGCCCCGGCCCAG
ATCGTGACGGGCGGGGAATCCTTCGGCGTGCGCGCCGACAATACCCGCCTGCTGCGGGTGGTCAATGCCGTGCTCGAAGC
CATTCCGGCCTCCGAGCGCCGCAGCCTGATCTACCGCTGGGGCCTGGGCAGCAGCATTTCGCTCGATTTCGCGCGCCCCG
CCTATTCAGCGCGCGAGCAGCAGTGGATGGCAAACCATCCGGTCGTCAAGGTGGCGGTCCTGAACCTGTTCGCGCCCTTC
ACCCTGTTTCGCACCGATGAACAGTTCGGCGGCATCAGTGCCGCCGTGCTGCAACTGCTGCAGTTGCGCACCGGCCTGGA
TTTCCAGATCATCGGCGTCGACACGGTCGAGGAGCTGATCGCCAAGCTGCGCTCGGGCGAAGCCGACATGGCCGGCGCCC
TGTTCGTCAATGCCGCGCGGGAATCCGTCCTCAGCTTCAGCCGGCCGTATGTGCGCAATGGTCTGGTGATCGTCACGCGC
CAGGACCCCGCCGCGCCCGCCGACGCCGATCACCTCGACGGCCGCACGATTGCGATGGTGCGCAACAGCGCCGCCATCCC
GCTCCTGCAGCAGCGCTATCCCCAGGCGAAGGTCGTGACCGCCGACAACCCGACCGAAGCCATGCTGCTGGTGGCCGATG
GCCAGGCCGACGCCGTCGTGCAGACGCAGATCAGCGCCAGCTACTACGTCAACCGCTACTTCGCCGGAAAACTGCGCATT
GCCTCGGCGCTGGACCTGCCGCCGGCCGAGATCGCGCTGGCGACGGCGCGCGGCCAGACCGAGCTGATATCCATCCTGAA
CAAGGCGCTCTACAGCATTTCGAACGACGAACTCGCCTCCATCGTCAGCCGCTGGCGCGGCAGCGACGGCGATCCGCGCA
CCTGGTACGCCTACCGCAACGAAATCTACCTGCTGATCGGGCTGGGCCTGTTGTCGGCCCTGCTGTTCCTGAGCTGGATC
GTCTACCTGCGGCGCCAGATCCGCCAGCGCAAGCGGGCCGAGCGGGCGCTGAACGACCAGCTGGAATTCATGCGCGTGCT
CATCGACGGCACGCCCAACCCCATCTATGTGCGCGATAAGGAAGGCCGCATGCTGTTGTGCAATGACGCCTACCTCGACA
CCTTTGGCGTGACTGCCGATGCGGTACTGGGCAAGACCATCCCGGAGGCCAACGTGGTGGGCGACCCGGCGCTGGCTCGC
GAGATGCACGAGTTCCTGCTCACGCGCATGGCCGCCGAGCGCGAGCCGCGCTTCGAGGACCGCGATGTCACGCTGCACGG
CCGCACCCGCCATGTCTACCAGTGGACGGTTCCGTACGGCGACTCGCTGGGCGAACTCAAGGGCATCATCGGCGGCTGGA
TCGACATTACCGAACGCGCCGAGCTGCTGCGCGAGCTGCACGACGCCAAGGAAAGCGCCGACGCCGCCAACCGGGCCAAG
ACCACGTTCCTGGCAACGATGAGCCACGAGATCCGCACGCCGATGAACGCGATCATCGGCATGCTGGAGCTGGCGCTGCT
CCGTCCGGCCGACCAGGAGCCGGACCGCCAGTCCATCCAGGTCGCGTACGACTCGGCCCGCAGCCTGCTGGAGCTGATAG
GCGACATCCTGGACATTGCGAAGATCGAGGCGGGAAAATTCGACCTGGCGCCGGTGCGCACGGCGCTGCGCGCCCTGCCC
GAAGGGGCGATCCGCGTCTTCGACGGGTTGGCGCGCCAGAAAGGCATAGAGCTGGTATTGAAGACCGACATCGTGGGCGT
GGACGATGTATTGATAGACCCCTTGCGCATGAAGCAAGTGCTCTCGAACCTGGTGGGCAACGCCATCAAGTTCACCACCG
AAGGCCAGGTTGTCCTTACCGTGACCGCGCGCCCCGACGGCGAGGCCGCGCACGTGCAGTTCAGCGTGAGCGACACCGGC
TGCGGCATCAGCGAGGCCGACCAACGGCAGCTGTTCAAACCGTTCTCGCAGGTGGGCGGCAGCGCCGAGGCCGGGCCGGC
GCCGGGCACTGGCCTGGGCCTGTCCATCAGCCGCCGCCTCGTCGAATTGATGGGGGGAACGCTGGTCATGCGCAGCGCGC
CAGGGGTGGGCACAACGGTTTCGGTGGACCTGAGGCTGACCATGGTCGAAAAATCCGCGCAGGCCACGCCGCCCGCTGCG
GCCGCTCAGGCCACGCCATCCAAGCCGCAGGTATCGCTGCGCGTGCTGGTCGTCGATGACCACAAGCCCAACCTGATGCT
GCTGCGCCAGCAGCTGGACTACCTGGGCCAGCGTGTCGTCGCCGCCGACTCCGGCGAAGCCGCCCTGGCCCTGTGGCACG
AGCATGCGTTCGACGTCGTGATCACCGATTGCAACATGCCCGGTATCAACGGCTACGAATTGGCGCGCCGCATACGCGCC
GCCGAGGCCGCGCCCGGTTACGGACGTACGCGGTGCATTCTGTTCGGCTTCACGGCTTCGGCGCAGATGGACGAAGCGCA
GCGCTGCCGCGCCGCCGGCATGGACGACTGCCTGTTCAAGCCGATCGGCGTGGACGCCTTGCGGCAACGCTTGAACGAAG
CCGCGGCACGGGCCGCGCTCCCCACGCCCCCCTCGCCCCAGGCTGCCGCGCCGGCCACGCACGACGCCACCCCGGCGGCG
TTCTCGGCCGAGTCGATTCTTGCCCTGACGCAGAACGATGAGGCGCTGATCCGGCAATTGCTCGAAGAAGTGATTCGCAC
CAACCGGGCGGACGTCGATCAATTGCAGAAGCTGCACCAGCAGGCCGATTGGCCGAAGGTCTCGGACATGGCGCACAGGC
TGGCCGGCGGCGCGCGCGTGGTCGATGCCAAGGCCATGATAGACACTGCGCTGGCGCTGGAGAAAAAAGCGCAAGGCCAG
GCTGGCCCCTCGCCCGAAATCGACGGCATGGTACGTACGCTTGCGGCGCAGTCCGCCGCGCTGGAGACGCAACTACGCGC
CTGGCTGGAGCAACGGCCGCATCAAGGCCAGCCCTGA
>Bb_B1977.seq
ATGCCCGCCCCGCACCGCCTGTACCCCCGCAGTCTGATCTGCCTGGCTCAGGCGCTATTGGCATGGGCTTTGCTGGCATG
GGCGCCCGCGCAGGCAAGCCAGGAGCTGACCCTGGTCGGCAAGGCTGCCGTTCCCGACGTCGAGATCGCGCTCGACGGCG
ACGACTGGCGCTGGCTGGCGCGCAAGCGGGTGCTGACGCTGGGCGTGTACGCGCCGGACATTCCCCCGTTCGACGTCACC
TATGACGAGCGCTACGAAGGCCTGACGGCCGACTACATGGCGATCATCGCACACAACCTGGGCGTCCAGGCAAAAGTGCT
GCGCTACCCCACGCGCGAGCAAGCCGTCGGCGCACTGGAAAGCGGACAGATCGACCTCATCGGCACCGTCAATGGCATCG
AGGGCCGGCTGCAGAGCCTGCGCCTGAGTGTTCCCTACGCGGCCGACCACCCGGTGCTGGTCATGCCCATCGGCGCGCGC
CGCGCTCCGCCCGCGGACCTGGCAGGCCAGCGGTTGGCGGTTGACGCCAACTACTTGCCCAGGGAAACGCTGCAGCAGGC
CTATCCCCAGGCAACGCTGCATTACTTCCCATCGTCCGAACAGGCGCTGGCCGCGGTGGCCTATGGACAAGCCGACGTGT
TCATCGGCGATGCGCTGACCACCTCGCACCTCGTTTCGCAAaGCTACTTCAACGACGTTCGCGTCGTCGCCCCGGCCCAG
ATCGTGACGGGCGGGGAATCCTTCGGCGTGCGCGCCGACAATACCCGCCTGCTGCGGGTGGTCAATGCCGTACTCGAAGC
CATTCCGGCCTCCGAGCGCCGCAGCCTGATCTACCGCTGGGGCCTGGGCAGCAGCATTTCGCTCGATTTCGCGCGCCCCG
CCTATTcAGCGCGCGAGCAGCAGTGGATGGCAAACCATCCGGTCGTCAAGGTGGCGGTCCTGAACCTGTTCGCGCCCTTC
ACCCTGTTTCGCACCGATGAACAGTTCGGCGGCATCAGTGCCGCCGTGCTGCAACTGCTGCAGTTGCGCACCGGCCTGGA
TTTCCAGATCATCGGCGTCGACACGGTCGAGGAGCTGATCGCCAAGCTGCGCTCGGGCGAAGCCGACATGGCCGGCGCCC
TGTTCGTCAATGCCGCGCGGGAATCCGTCCTCAGCTTCAGCCGGCCGTATGTGCGCAATGGCATGGTGATCGTCACGCGC
CAGGACCCCGCCGCGCCCGCCGACGCCGATCACCTCGACGGCCGCACGATTGCGATGGTGCGCAACAGCGCCGCCATCCC
GCTCCTGCAGCAGCGCTATCCCCAGGCGAAGGTCGTGACCGCCGACAACCCGACCGAAGCCATGCTGCTGGTGGCCGATG
GCCAGGCCGACGCCGTCGTGCAGACGCAGATCAGCGCCAGCTACTACGTCAACCGCTACTTCGCCGGAAAACTGCGCATT
GCCTCGGCGCTGGACCTGCCGCCGGCCGAGATCGCGCTGGCGACGGCGCGCGGCCAGACCGAGCTGATATCCATCCTGAA
CAAGGCGCTCTACAGCATTTCGAACGACGAACTCGCCTCCATCGTCAGCCGCTGGCGCGGCAGCGACGGCGATCCGCGCA
CCTGGTACGCCTACCGCAACGAGATCTACCTGCTGATCGGGCTGGGCCTGTTGTCGGCCCTGCTGTTCCTGAGCTGGATC
GTCTACCTGCGGCGCCAGATCCGCCAGCGCAAGCGGGCCGAGCGGGCGCTGAACGACCAGCTGGAATTCATGCGCGTGCT
CATCGACGGCACGCCCAACCCCATCTATGTGCGCGATAAGGAAGGCCGCATGCTGTTGTGCAATGACGCCTACCTCGACA
CCTTTGGCGTGACTGCCGATGCGGTACTGGGCAAGACCATCCCGGAGGCCAACGTGGTGGGCGACCCGGCGCTGGCTCGC
GAGATGCACGAGTTCCTGCTCACGCGCATGTCCGCCGAGCGCGAGCCGCGCTTCGAGGACCGCGATGTCACGCTGCACGG
CCGCACCCGCCATGTCTACCAGTGGACGGTTCCGTACGGCGACTCGCTGGGCGAACTCAAGGGCATCATCGGCGGCTGGA
TCGACATTACCGAACGCGCCGAGCTGCTGCGCGAGCTGCACGACGCCAAGGAAAGCGCCGACGCCGCCAACCGGGCCAAG
ACCACGTTCCTGGCAACGATGAGCCACGAGATCCGCACGCCGATGAACGCGATCATCGGCATGCTGGAGCTGGCGCTGCT
CCGTCCGGCCGACCAGGAGCCGGACCGCCAGTCCATCCAGGTCGCGTACGACTCGGCCCGCAGCCTGCTGGAGCTGATAG
GCGACATCCTGGACATTGCGAAGATCGAGGCGGGAAAATTCGACCTGGCGCCGGTGCGCACGGCGCTGCGCGCCCTACCC
GAAGGGGCGATCCGCGTCTTCGACGGGTTGGCGCGCCAGAAAGGCATAGAGCTGGTATTGAAGACCGACATCGTGGGCGT
GGACGATGTATTGATAGACCCCTTGCGCATGAAGCAAGTGCTCTCGAACCTGGTGGGCAACGCCATCAAGTTCACCACCG
AAGGCCAGGTTGTCCTTACCGTGACCGCGCGCCCCGACGGCGAGGCCGCGCACGTGCAGTTCAGCGTGAGCGACACCGGC
TGCGGCATCAGCGAGGCCGACCAACGGCAGCTGTTCAAACCGTTCTCGCAGGTGGGCGGCAGCGCCGAGGCCGGGCCGGC
GCCGGGCACTGGCCTGGGCCTGTCCATCAGCCGCCGCCTCGTCGAATTGATGGGGGGAACGCTGGTCATGCGCAGCGCGC
CAGGGGTGGGCACAACGGTTTCGGTGGACCTGAGGCTGACCATGGTCGAAAAATCCGCGCAGGCCACGCCGCCCGCTGCG
GCCGCTCAGGCCACGCCATCCAAGCCGCAGGTATCGCTGCGCGTGCTGGTCGTCGATGACCACAAGCCCAACCTGATGCT
GCTGCGCCAGCAGCTGGACTACCTGGGCCAGCGTGTCGTCGCCGCCGACTCCGGCGAAGCCGCCCTGGCCCTGTGGCACG
AGCATGCGTTCGACGTCGTGATCACCGATTGCAACATGCCCGGTATCAACGGCTACGAATTGGCGCGCCGCATACGCGCC
GCCGAGGCCGCGCCCGGTTACGGACGTACGCGGTGCATTCTGTTCGGCTTCACGGCTTCGGCGCAGATGGACGAAGCGCA
GCGCTGCCGCGCCGCCGGCATGGACGACTGCCTGTTCAAGCCGATCGGCGTGGACGCCTTGCGGCAACGCTTGAACGAAG
CCGCGGCACGGGCCGCGCTCCCCACGCCCCCCTCGCCCCAGGCTGCCGCGCCGGCCACGCACGACGCCACCCCGGCGGCG
TTCTCGGCCGAGTCGATTCTTGCCCTGACGCAGAACGATGAGGCGCTCATCCGGCAATTGCTCGAAGAACTGATTCGCAC
CAACCGGGCGGACGTCGATCAATTGCAGAAGCTGCACCAGCAGGCCGATTGGCCGAAGGTCTCGGACATGGCGCACAGGC
TGGCCGGCGGCGCGCGCGTGGTCGATGCCAAGGCCATGATAGACACTGCGCTGGCGCTGGAAAAAAAAGCGCAAGGCCAG
GCTGGCCCCTCGCCCGAAATCGACGGCATGGTACGTACGCTTGCGGCGCAGTCCGCCGCGCTGGAGACGCAACTACGCGC
CTGGCTGGAGCAACGGCCGCATCAAGGCCAGCCCTGA
>Bb_B1986.seq
ATGCCCGCCCCGCACCGCCTGTACCCCCGCAGTCTGATCTGCCTGGCTCAGGCGCTATTGGCATGGGCTTTGCTGGCATG
GGCGCCCGCGCAGGCAAGCCAGGAGCTGACCCTGGTCGGCAAGGCTGCCGTTCCCGACGTCGAGATCGCGCTCGACGGCG
ACGACTGGCGCTGGCTGGCGCGCAAGCGGGTGCTGACGCTGGGCGTGTACGCGCCGGACATTCCCCCGTTCGACGTCACC
TATGACGAGCGCTACGAAGGCCTGACGGCCGACTACATGGCGATCATCGCGCACAACCTGGGCGTCCAGGCAAAAGTGCT
GCGCTACCCCACGCGCGAGCAAGCCGTCGGCGCACTGGAAAGCGGACAGATCGACCTCATCGGCACCGTCAATGGCATCG
AGGGCCGGCTGCAGAGCCTGCGCCTGAGTGTTCCCTACGCGGCCGACCACCCGGTGCTGGTCATGCCCATCGGCGCGCGC
CGCGCTCCGCCCGCGGACCTGGCAGGCCAGCGGTTGGCGGTTGACGCCAACTACTTGCCCAGGGAAACGCTGCAGCAGGC
CTATCCCCAGGCAACGCTGCATTACTTCCCATCGTCCGAACAGGCGCTGGCCGCGGTGGCCTATGGACAAGCCGACGTGT
TCATCGGCGATGCGCTGACCGCCTCGCACCTCGTTTCGCAAAGCTACTTCAACGACGTTCGCGTCGTCGCCCCGGCCCAG
ATCGTGACGGGCGGGGAATCCTTCGGCGTGCGCGCCGACAATACCCGCCTGCTGCGGGTGGTCAATGCCGTGCTCGAAGC
CATTCCGGCCTCCGAGCGCCGCAGCCTGATCTACCGCTGGGGCCTGGGCAGCAGCATTTCGCTCGATTTCGCGCGCCCCG
CCTATTCAGCGCGCGAGCAGCAGTGGATGGCAAACCATCCGGTCGTCAAGGTGGCGGTCCTGAACCTGTTCGCGCCCTTC
ACCCTGTTTCGCACCGATGAACAGTTCGGCGGCATCAGTGCCGCCGTGCTGCAACTGCTGCAGTTGCGCACCGGCCTGGA
TTTCCAGATCATCGGCGTCGACACGGTCGAGGAGCTGATCGCCAAGCTGCGCTCGGGCGAAGCCGACATGGCCGGCGCCC
TGTTCGTCAATGCCGCGCGGGAATCCGTCCTCAGCTTCAGCCGGCCGTATGTGCGCAATGGTCTGGTGATCGTCACGCGC
CAGGACCCCGCCGCGCCCGCCGACGCCGATCACCTCGACGGCCGCACGATTGCGATGGTGCGCAACAGCGCCGCCATCCC
GCTCCTGCAGCAGCGCTATCCCCAGGCGAAGGTCGTGACCGCCGACAACCCGACCGAAGCCATGCTGCTGGTGGCCGATG
GCCAGGCCGACGCCGTCGTGCAGACGCAGATCAGCGCCAGCTACTACGTCAACCGCTACTTCGCCGGAAAACTGCGCATT
GCCTCGGCGCTGGACCTGCCGCCGGCCGAGATCGCGCTGGCGACGGCGCGCGGCCAGACCGAGCTGATATCCATCCTGAA
CAAGGCGCTCTACAGCATTTCGAACGACGAACTCGCCTCCATCGTCAGCCGCTGGCGCGGCAGCGACGGCGATCCGCGCA
CCTGGTACGCCTACCGCAACGAAATCTACCTGCTGATCGGGCTGGGCCTGTTGTCGGCCCTGCTGTTCCTGAGCTGGATC
GTCTACCTGCGGCGCCAGATCCGCCAGCGCAAGCGGGCCGAGCGGGCGCTGAACGACCAGCTGGAATTCATGCGCGTGCT
CATCGACGGCACGCCCAACCCCATCTATGTGCGCGATAAGGAAGGCCGCATGCTGTTGTGCAATGACGCCTACCTCGACA
CCTTTGGCGTGACTGCCGATGCGGTACTGGGCAAGACCATCCCGGAGGCCAACGTGGTGGGCGACCCGGCGCTGGCTCGC
GAGATGCACGAGTTCCTGCTCACGCGCATGGCCGCCGAGCGCGAGCCGCGCTTCGAGGACCGCGATGTCACGCTGCACGG
CCGCACCCGCCATGTCTACCAGTGGACGGTTCCGTACGGCGACTCGCTGGGCGAACTCAAGGGCATCATCGGCGGCTGGA
TCGACATTACCGAACGCGCCGAGCTGCTGCGCGAGCTGCACGACGCCAAGGAAAGCGCCGACGCCGCCAACCGGGCCAAG
ACCACGTTCCTGGCAACGATGAGCCACGAGATCCGCACGCCGATGAACGCGATCATCGGCATGCTGGAGCTGGCGCTGCT
CCGTCCGGCCGACCAGGAGCCGGACCGCCAGTCCATCCAGGTCGCGTACGACTCGGCCCGCAGCCTGCTGGAGCTGATAG
GCGACATCCTGGACATTGCGAAGATCGAGGCGGGAAAATTCGACCTGGCGCCGGTGCGCACGGCGCTGCGCGCCCTGCCC
GAAGGGGCGATCCGCGTCTTCGACGGGTTGGCGCGCCAGAAAGGCATAGAGCTGGTATTGAAGACCGACATCGTGGGCGT
GGACGATGTATTGATAGACCCCTTGCGCATGAAGCAAGTGCTCTCGAACCTGGTGGGCAACGCCATCAAGTTCACCACCG
AAGGCCAGGTTGTCCTTACCGTGACCGCGCGCCCCGACGGCGAGGCCGCGCACGTGCAGTTCAGCGTGAGCGACACCGGC
TGCGGCATCAGCGAGGCCGACCAACGGCAGCTGTTCAAACCGTTCTCGCAGGTGGGCGGCAGCGCCGAGGCCGGGCCGGC
GCCGGGCACTGGCCTGGGCCTGTCCATCAGCCGCCGCCTCGTCGAATTGATGGGGGGAACGCTGGTCATGCGCAGCGCGC
CAGGGGTGGGCACAACGGTTTCGGTGGACCTGAGGTTGACCATGGTCGAAAAATCCGCGCAGGCCACGCCGCCCGCTGCG
GCCGCTCAGGCCACGCCATCCAAGCCGCAGGTATCGCTGCGCGTGCTGGTCGTCGATGACCACAAGCCCAACCTGATGCT
GCTGCGCCAGCAGCTGGACTACCTGGGCCAGCGTGTCGTCGCCGCCGACTCCGGCGAAGCCGCCCTGGCCCTGTGGCACG
AGCATGCGTTCGACGTCGTGATCACCGATTGCAACATGCCCGGTATCAACGGCTACGAATTGGCGCGCCGCATACGCGCC
GCCGAGGCCGCGCCCGGTTACGGACGTACGCGGTGCATTCTGTTCGGCTTCACGGCTTCGGCGCAGATGGACGAAGCGCA
GCGCTGCCGCGCCGCCGGCATGGACGACTGCCTGTTCAAGCCGATCGGCGTGGACGCCTTGCGGCAACGCTTGAACGAAG
CCGCGGCACGGGCCGCGCTCCCCACGCCCCCCTCGCCCCAGGCTGCCGCGCCGGCCACGCACGACGCCACCCCGGCGGCG
TTCTCGGCCGAGTCGATTCTTGCCCTGACGCAGAACGATGAGGCGCTCATCCGGCAATTGCTCGAAGAAGTGATTCGCAC
CAACCGGGCGGACGTCGATCAATTGCAGAAGCTGCACCAGCAGGCCGATTGGCCGAAGGTCTCGGACATGGCGCACAGGC
TGGCCGGCGGCGCGCGCGTGGTCGATGCCAAGGCCATGATAGACACTGCGCTGGCGCTGGAGAAAAAAGCGCAAGGCCAG
GCTGGCCCCTCGCCCGAAATCGACGGCATGGTACGTACGCTTGCGGCGCAGTCCGCCGCGCTGGAGACGCAACTACGCGC
CTGGCTGGAGCAACGGCCGCATCAAGGCCAGCCCTGA
>Bb_B1987.seq
ATGCCCGCCCCGCACCGCCTGTACCCCCGCAGTCTGATCTGCCTGGCTCAGGCGCTATTGGCATGGGCTTTGCTGGCATG
GGCGCCCGCGCAGGCAAGCCAGGAGCTGACCCTGGTCGGCAAGGCTGCCGTTCCCGACGTCGAGATCGCGCTCGACGGCG
ACGACTGGCGCTGGCTGGCGCGCAAGCGGGTGCTGACGCTGGGCGTGTACGCGCCGGACATTCCCCCGTTCGACGTCACC
TATGACGAGCGCTACGAAGGCCTGACGGCCGACTACATGGCGATCATCGCGCACAACCTGGGCGTCCAGGCAAAAGTGCT
GCGCTACCCCACGCGCGAGCAAGCCGTCGGCGCACTGGAAAGCGGACAGATCGACCTCATCGGCACCGTCAATGGCATCG
AGGGCCGGCTGCAGAGCCTGCGCCTGAGTGTTCCCTACGCGGCCGACCACCCGGTGCTGGTCATGCCCATCGGCGCGCGC
CGCGCTCCGCCCGCGGACCTGGCAGGCCAGCGGTTGGCGGTTGACGCCAACTACTTGCCCAGGGAAACGCTGCAGCAGGC
CTATCCCCAGGCAACGCTGCATTACTTCCCATCGTCCGAACAGGCGCTGGCCGCGGAGGCCTATGGACAAGCCGACGTGT
TCATCGGCGATGCGCTGACCACCTCGCACCTCGTTTCGCAAAGCTACTTCAACGACGTTCGCGTCGTCGCCCCGGCCCAG
ATCGTGACGGGCGGGGAATCCTTCGGCGTGCGCGCCGACAATACCCGCCTGCTGCGGGTGGTCAATGCCGTGCTCGAAGC
CATTCCGGCCTCCGAGCGCCGCAGCCTGATCTACCGCTGGGGCCTGGGCAGCAGCATTTCGCTCGATTTCGCGCGCCCCG
CCTATTCAGCGCGCGAGCAGCAGTGGATGGCAAACCATCCGGTCGTCAAGGTGGCGGTCCTGAACCTGTTCGCGCCCTTC
ACCCTGTTTCGCACCGATGAACAGTTCGGCGGCATCAGTGCCGCCGTGCTGCAACTGCTACAGTTGCGCACCGGCCTGGA
TTTCCAGATCATCGGCGTCGACACGGTCGAGGAGCTGATCGCCAAGCTGCGCTCGGGCGAAGCCGACATGGCCGGCGCCC
TGTTCGTCAATGCCGCGCGGGAATCCGTCCTCAGCTTCAGCCGGCCGTATGTGCGCAATGGTCTGGTGATCGTCACGCGC
CAGGACCCCGCCGCGCCCGCCGACGCCGATCACCTCGACGGCCGCACGATTGCGATGGTGCGCAACAGCGCCGCCATCCC
GCTCCTGCAGCAGCGCTATCCCCAGGCGAAGGTCGTGACCGCCGACAACCCGACCGAAGCCATGCTGCTGGTGGCCGATG
GCCAGGCCGACGCCGTCGTGCAGACGCAGATCAGCGCCAGCTACTACGTCAACCGCTACTTCGCCGGAAAACTGCGCATT
GCCTCGGCGCTGGACCTGCCGCCGGCCGAGATCGCGCTGGCGACGGCGCGCGGCCAGACCGAGCTGATATCCATCCTGAA
CAAGGCGCTCTACAGCATTTCGAACGACGAACTCGCCTCCATCGTCAGCCGCTGGCGCGGCAGCGACGGCGATCCGCGCA
CCTGGTACGCCTACCGCAACGAAATCTACCTGCTGATCGGGCTGGGCCTGTTGTCGGCCCTGCTGTTCCTGAGCTGGATC
GTCTACCTGCGGCGCCAGATCCGCCAGCGCAAGCGGGCCGAGCGGGCGCTGAACGACCAGCTGGAATTCATGCGCGTGCT
CATCGACGGCACGCCCAACCCCATCTATGTGCGCGATAAGGAAGGCCGCATGCTGTTGTGCAATGACGCCTACCTCGACA
CCTTTGGCGTGACTGCCGATGCGGTACTGGGCAAGACCATCCCGGAGGCCAACGTGGTGGGCGACCCGGCGCTGGTTCGC
GAGATGCACGAGTTCCTGCTCACGCGCATGGCCGCCGAGCGCGAGCCGCGCTTCGAGGACCGCGATGTCACGCTGCACGG
CCGCACCCGCCATGTCTACCAGTGGACGGTTCCGTACGGCGACTCGCTGGGCGAACTCAAGGGCATCATCGGCGGCTGGA
TCGACATTACCGAACGCGCCGAGCTGCTGCGCGAGCTGCACGACGCCAAGGAAAGCGCCGACGCCGCCAACCGGGCCAAG
ACCACGTTCCTGGCAACGATGAGCCACGAGATCCGCACGCCGATGAACGCGATCATCGGCATGCTGGAGCTGGCGCTGCT
CCGTCCGGCCGACCAGGAGCCGGACCGCCAGTCCATCCAGGTCGCGTACGACTCGGCCCGCAGCCTGCTGGAGCTGATAG
GCGACATCCTGGACATTGCGAAGATCGAGGCGGGAAAATTCGACCTGGCGCCGGTGCGCACGGCGCTGCGCGCCCTGCCC
GAAGGGGCGATCCGCGTCTTCGACGGGTTGGCGCGCCAGAAAGGCATAGAGCTGGTATTGAAGACCGACATCGTGGGCGT
GGACGATGTATTGATAGACCCCTTGCGCATGAAGCAAGTGCTCTCGAACCTGGTGGGCAACGCCATCAAGTTCACCACCG
AAGGCCAGGTTGTCCTTACCGTGACCGCGCGCCCCGACGGCGAGGCCGCGCACGTGCAGTTCAGCGTGAGCGACACCGGC
TGCGGCATCAGCGAGGCCGACCAACGGCAGCTGTTCAAACCGTTCTCGCAGGTGGGCGGCAGCGCCGAGGCCGGGCCGGC
GCCGGGCACTGGCCTGGGCCTGTCCATCAGCCGCCGCCTCGTCGAATTGATGGGGGGAACGCTGGTCATGCGCAGCGCGC
CAGGGGTGGGCACAACGGTTTCGGTGGACCTGAGGCTGACCATGGTCGAAAAATCCGCGCAGGCCACGCCGCCCGCTGCG
GCCGCTCAGGCCACGCCATCCAAGCCGCAGGTATCGCTGCGCGTGCTGGTCGTCGATGACCACAAGCCCAACCTGATGCT
GCTGCGCCAGCAGCTGGACTACCTGGGCCAGCGTGTCGTCGCCGCCGACTCCGGCGAAGCCGCCCTGGCCCTGTGGCACG
AGCATGCGTTCGACGTCGTGATCACCGATTGCAACATGCCCGGTATCAACGGCTACGAATTGGCGCGCCGCATACGCGCC
GCCGAGGCCGCGCCCGGTTACGGACGTACGCGGTGCATTCTGTTCGGCTTCACGGCTTCGGCGCAGATGGACGAAGCGCA
GCGCTGCCGCGCCGCCGGCATGGACGACTGCCTGTTCAAGCCGATCGGCGTGGACGCCTTGCGGCAACGCTTGAACGAAG
CCGCGGCACGGGCCGCGCTCCCCACGCCCCCCTCGCCCCAGGCTGCCGCGCCGGCCACGCACGACGCCACCCCGGCGGCG
TTCTCGGCCGAGTCGATTCTTGCCCTGACGCAGAACGATGAGGCGCTCATCCGGCAATTGCTCGAAGAAGTGATTCGCAC
CAACCGGGCGGACGTCGATCAATTGCAGAAGCTGCACCAGCAGGCCGATTGGCCGAAGGTCTCGGACATGGCGCACAGGC
TGGCCGGCGGCGCGCGCGTGGTCGATGCCAAGGCCATGATAGACACTGCGCTGGCGCTGGAGAAAAAAGCGCAAGGCCAG
GCTGGCCCCTCGCCCGAAATCGACGGCATGGTACGTACGCTTGCGGCGCAGTCCGCCGCGCTGGAGACGCAACTACGCGC
CTGGCTGGAGCAACGGCCGCATCAAGGCCAGCCCTGA
>Bb_B2104.seq
ATGCCCGCCCCGCACCGCCTGTACCCCCGCAGTCTGATCTGCCTGGCTCAGGCGCTATTGGCATGGGCTTTGCTGGCATG
GGCGCCCGCGCAGGCAAGCCAGGAGCTGACCCTGGTCGGCAAGGCTGCCGTTCCCAACGTCGAGATCACGCTCGACGGCG
ACGACTGGCGCTGGCTGGCGCGCAAGCGGGTGCTGACGCTGGGCGTGTACGCGCCGGACATTCCCCCGTTCGACGTCACC
TATGACGAGCGCTACGAAGGCCTGACGGCCGACTACATGGCGATCATCGCGCACAACCTGGGCGTCCAGGCAAAAGTGCT
GCGCTACCCCACGCGCGAGCAAGCCGTCGGCGCACTGGAAAGCGGACAGATCGACCTCATCGGCACCGTCAATGGCATCG
AGGGCCGGCTGCAGAGCCTGCGCCTGAGTGTTCCCTACGCGGCCGACCACCCGGTGCTGGTCATGCCCATCGGCGCGCGC
CGCGCTCCGCCCGCGGACCTGGCAGGCCAGCGGTTGGCGGTTGACGCCAACTACTTGCCCAGGGAAACGCTGCAGCAGGC
CTATCCCCAGGCAACGCTGCATTACTTCCCATCATCCGAACAGGCGCTGGCCGCGGTGGCCTATGGACAAGCCGACGTGT
TCATCGGCGATGCGCTGACCACCTCGCACCTCGTTTCGCAAAGCTACTTCAACGACGTTCGCGTCGTCGCCCCGGCCCAG
ATCGTGACGGGCGGGGAATCCTTCGGCGTGCGCGCCGACAATACCCGCCTGCTGCGGGTGGTCAATGCCGTGCTCGAAGC
CATTCCGGCCTCCGAGCGCCGCAGCCTGATCTACCGCTGGGGCCTGGGCAGCAGCATTTCGCTCGATTTCGCGCGCCCCG
CCTATTCAGCGCGCGAGCAGCAGTGGATGGCAAACCATCCGGTCGTCAAGGTGGCGGTCCTGAACCTGTTCGCGCCCTTC
ACCCTGTTTCGCACCGATGAACAGTTCGGCGGCATCAGTGCCGCCGTGCTGCAACTGCTGCAGTTGCGCACCGGCCTGGA
TTTCCAGATCATCGGCGTCGACACGGTCGAGGAGCTGATCGCCAAGCTGCGCTCGGGCGAAGCCGACATGGCCGGCGCCC
TGTTCGTCAATGCCGCGCGGGAATCCGTCCTCAGCTTCAGCCGGCCGTATGTGCGCAATGGTCTGGTGATCGTCACGCGC
CAGGACCCCGCCGCGCCCGCCGACGCCGATCACCTCGACGGCCGCACGATTGCGATGGTGCGCAACAGCGCCGCCATCCC
GCTCCTGCAGCAGCGCTATCCCCAGGCGAAGGTCGTGACCGCCGACAACCCGACCGAAGCCATGCTGCTGGTGGCCGATG
GCCAGGCCGACGCCGTCGTGCAGACGCAGATCAGCGCCAGCTACTACGTCAACCGCTACTTCGCCGGAAAACTGCGCATT
GCCTCGGCGCTGGACCTGCCGCCGGCCGAGATCGCGCTGGCGACGGCGCGCGGCCAGACCGAGCTGATATCCATCCTGAA
CAAGGCGCTCTACAGCATTTCGAACGACGAACTCGCCTCCATCGTCAGCCGCTGGCGCGGCAGCGACGGCGATCCGCGCA
CCTGGTACGCCTACCGCAACGAAATCTACCTGCTGATCGGGCTGGGCCTGTTGTCGGCCCTGCTGTTCCTGAGCTGGATC
GTCTACCTGCGGCGCCAGATCCGCCAGCGCAAGCGGGCCGAGCGGGCGCTGAACGACCAGCTGGAATTCATGCGCGTGCT
CATCGACGGCACGCCCAACCCCATCTATGTGCGCGATAAGGAAGGCCGCATGCTGTTGTGCAATGACGCCTACCTCGACA
CCTTTGGCGTGACTGCCGATGCGGTACTGGGCAAGACCATCCCGGAGGCCAACGTGGTGGGCGACCCGGCGCTGGCTCGC
GAGATGCACGAGTTCCTGCTCACGCGCATGGCCGCCGAGCGCGAGCCGCGCTTCGAGGACCGCGATGTCACGCTGCACGG
CCGCACCCGCCATGTCTACCAGTGGACGGTTCCGTACGGCGACTCGCTGGGCGAACTCAAGGGCATCATCGGCGGCTGGA
TCGACATTACCGAACGCGCCGAGCTGCTGCGCGAGCTGCACGACGCCAAGGAAAGCGCCGACgCCGCCAACCGGGCCAAG
ACCACGTTCCTGGCAACGATGAGCCACGAGATCCGCACGCCGATGaACGCGATCATCGGCATGCTGGAGCTGGCGCTGCT
CCGTCCGGCCGACCAGGAGCCGGACCGCCAGTCCATCCAGGTCGCGTACGACTCGGCCCGCAGCCTGCTGGAGCTGATAG
GCGACATCCTGGACATTGCGAAGATCGAGGCGGGAAAATTCGACCTGGCGCCGGTGCGCACGGCGCTGCGCGCCCTGCCC
GAAGGGGCGATCCGCGTCTTCGACGGGTTGGCGCGCCAGAAAGGCATAGAGCTGGTATTGAAGACCGACATCGTGGGCGT
GGACGATGTATTGATAGACCCCTTGCGCATGAAGCAAGTGCTCTCGAACCTGGTGGGCAACGCCATCAAGTTCACCACCG
AAGGCCAGGTTGTCCTTACCGTGACCGCGCGCCCCGACGGCGAGGCCGCGCACGTGCAGTTCAGCGTGAGCGACACCGGC
TGCGGCATCAGCGAGGCCGACCAACGGCAGCTGTTCAAACCGTTCTCGCAGGTGGGCGGCAGCGCCGAGGCCGGGCCGGC
GCCGGGCACTGGCCTGGGCCTGTCCATCAGCCGCCGCCTCGTCGAATTGATGGGGGGAACGCTGGTCATGCGCAGCGCGC
CAGGGGTGGGCACAACGGTTTCGGTGGACCTGAGGCTGACCATGGTCGAAAAATCCGCGCAGGCCACGCCGCCCGCTGCG
GCCGCTCAGGCCACGCCATCCAAGCCGCAGGTATCGCTGCGCGTGCTGGTCGTCGATGACCACAAGCCCAACCTGATGCT
GCTGCGCCAGCAGCTGGACTACCTGGGCCAGCGTGTCGTCGCCGCCGACTCCGGCGAAGCCGCCCTGGCCCTGTGGCACG
AGCATGCGTTCGACGTCGTGATCACCGATTGCAACATGCCCGGTATCAACGGCTACGAATtGGCGCGCCGCATACGCGCC
GCCGAGGCCGCGCCCGGTTACGGACGTACGCGGTGCATTCTGTTCGGCTTCACGGCTTCGGCGCATATGGACGAAGCGCA
GCGCTGCCGCGCCGCCGGCATGGACGACTGCCTGTTCAAGCCGATCGGCGTGGACGCCTTGCGGCAACGCTTGAACAAAG
CCGCGGCACGGGCCGCGCTCCCCACGCCCCCCTCGCCCCAGGCTGCCGCGCCGGCCACGCACGACGCCACCCCGGCGGCG
TTCTCGGCCGAGTCGATTCTTGCCCTGACGCAGAACGATGAGGCGCTGATCCGGCAATTGCTCGAAGAAGTGATTCGCAC
CAACCGGGCGGACGTCGATCAATTGCAGAAGCTGCACCAGCAGGCCGATTGGCCGAAGGTCTCGGACATGGCGCACAGGC
TGGCCGGCGGCGCGCGCGTGGTCGATGCCAAGGCCATGATAGACACTGCGCTGGCGCTGGAGAAAAAAGCGCAAGGCCAG
GCTGGCCCCTCGCCCGAAATCGACGGCATGGTACGTACGCTTGCGGCGCAGTCCGCCGCGCTGGAGACGCAACTACGCGC
CTGGCTGGAGCAACGGCCGCATCAAGGCCAGCCCTGA
>Bb_B2105.seq
ATGCCCGCCCCGCACCGCCTGTACCCCCGCAGTCTGATCTGCCTGGCTCAGGCGCTATTGGCATGGGCTTTGCTGGCATG
GGCGCCCGCGCAGGCAAGCCAGGAGCTGACCCTGGTCGGCAAGGCTGCCGTTCCCGACGTCGAGATCACGCTCGACGGCG
ACGACTGGCGCTGGCTGGCGCGCAAGCGGGTGCTGACGCTGGGCGTGTACGCGCCGGACATTCCCCCGTTCGACGTCACC
TATGACGAGCGCTACGAAGGCCTGACGGCCGACTACATGGCGATCATCGCGCACAACCTGGGCGTCCAGGCAAAAGTGCT
GCGCTACCCCACGCGCGAGCAAGCCGTCGGCGCACTGGAAAGCGGACAGATCGACCTCATCGGCACCGTCAATGGCATCG
AGGGCCGGCTGCAGAGCCTGCGCCTGAGTGTTCCCTACGCGGCCGACCACCCGGTGCTGGTCATGCCCATCGGCGCGCGC
CGCGCTCCGCCCGCGGACCTGGCAGGCCAGCGGTTGGCGGTTGACGCCAACTACTTGCCCAGGGAAACGCTGCAGCAGGC
CTATCCCCAGGCAACGCTGCATTACTTCCCATCATCCGAACAGGCGCTGGCCGCGGTGGCCTATGGACAAGCCGACGTGT
TCATCGGCGATGCGCTGACCACCTCGCACCTCGTTTCGCAAAGCTACTTCAACGACGTTCGCGTCGTCGCCCCGGCCCAG
ATCGTGACGGGCGGGGAATCCTTCGGCGTGCGCGCCGACAATACCCGCCTGCTGCGGGTGGTCAATGCCGTGCTCGAAGC
CATTCCGGCCTCCGAGCGCCGCAGCCTGATCTACCGCTGGGGCCTGGGCAGCAGCATTTCGCTCGATTTCGCGCGCCCCG
CCTATTCAGCGCGCGAGCAGCAGTGGATGGCAAACCATCCGGTCGTCAAGGTGGCGGTCCTGAACCTGTTCGCGCCCTTC
ACCCTGTTTCGCACCGATGAACAGTTCGGCGGCATCAGTGCCGCCGTGCTGCAACTGCTGCAGTTGCGCACCGGCCTGGA
TTTCCAGATCATCGGCGTCGACACGGTCGAGGAGCTGATCGCCAAGCTGCGCTCGGGCGAAGCCGACATGGCCGGCGCCC
TGTTCGTCAATGCCGCGCGGGAATCCGTCCTCAGCTTCAGCCGGCCGTATGTGCGCAATGGTCTGGTGATCGTCACGCGC
CAGGACCCCGCCGCGCCCGCCGACGCCGATCACCTCGACGGCCGCACGATTGCGATGGTGCGCAACAGCGCCGCCATCCC
GCTCCTGCAGCAGCGCTATCCCCAGGCGAAGGTCGTGACCGCCGACAACCCGACCGAAGCCATGCTGCTGGTGGCCGATG
GCCAGGCCGACGCCGTCGTGCAGACGCAGATCAGCGCCAGCTACTACGTCAACCGCTACTTCGCCGGAAAACTGCGCATT
GCCTCGGCGCTGGACCTGCCGCCGGCCGAGATCGCGCTGGCGACGGCGCGCGGCCAGACCGAGCTGATATCCATCCTGAA
CAAGGCGCTCTACAGCATTTCGAACGACGAACTCGCCTCCATCGTCAGCCGCTGGCGCGGCAGCGACGGCGATCCGCGCA
CCTGGTACGCCTACCGCAACGAAATCTACCTGCTGATCGGGCTGGGCCTGTTGTCGGCCCTGCTGTTCCTGAGCTGGATC
GTCTACCTGCGGCGCCAGATCCGCCAGCGCAAGCGGGCCGAGCGGGCGCTGAACGACCAGCTGGAATTCATGCGCGTGCT
CATCGACGGCACGCCCAACCCCATCTATGTGCGCGATAAGGAAGGCCGCATGCTGTTGTGCAATGACGCCTACCTCGACA
CCTTTGGCGTGACTGCCGATGCGGTACTGGGCAAGACCATCCCGGAGGCCAACGTGGTGGGCGACCCGGCGCTGGCTCGC
GAGATGCACGAGTTCCTGCTCACGCGCATGGCCGCCGAGCGCGAGCCGCGCTTCGAGGACCGCGATGTCACGCTGCACGG
CCGCACCCGCCATGTCTACCAGTGGACGGTTCCGTACGGCGACTCGCTGGGCGAACTCAAGGGCATCATCGGCGGCTGGA
TCGACATTACCGAACGCGCCGAGCTGCTGCGCGAGCTGCACGACGCCAAGGAAAGCGCCGACGCCGCCAACCGGGCCAAG
ACCACGTTCCTGGCAACGATGAGCCACGAGATCCGCACGCCGATGAACGCGATCATCGGCATGCTGGAGCTGGCGCTGCT
CCGTCCGGCCGACCAGGAGCCGGACCGCCAGTCCATCCAGGTCGCGTACGACTCGGCCCGCAGCCTGCTGGAGCTGATAG
GCGACATCCTGGACATTGCGAAGATCGAGGCGGGAAAATTCGACCTGGCGCCGGTGCGCACGGCGCTGCGCGCCCTGCCC
GAAGGGGCGATCCGCGTCTTCGACGGGTTGGCGCGCCAGAAAGGCATAGAGCTGGTATTGAAGACCGACATCGTGGGCGT
GGACGATGTATTGATAGACCCCTTGCGCATGAAGCAAGTGCTCTCGAACCTGGTGGGCAACGCCATCAAGTTCACCACCG
AAGGCCAGGTTGTCCTTACCGTGACCGCGCGCCCCGACGGCGAGGCCGCGCACGTGCAGTTCAGCGTGAGCGACACCGGC
TGCGGCATCAGCGAGGCCGACCAACGGCAGCTGTTCAAACCGTTCTCGCAGGTGGGCGGCAGCGCCGAGGCCGGGCCGGC
GCCGGGCACTGGCCTGGGCCTGTCCATCAGCCGCCGCCTCGTCGAATTGATGGGGGGAACGCTGGTCATGCGCAGCGCGC
CAGGGGTGGGCACAACGGTTTCGGTGGACCTGAGGCTGACCATGGTCGAAAAATCCGCGCAGGCCACGCCGCCCGCTGCG
GCCGCTCAGGCCACGCCATCCAAGCCGCAGGTATCGCTGCGCGTGCTGGTCGTCGATGACCACAAGCCCAACCTGATGCT
GCTGCGCCAGCAGCTGGACTACCTGGGCCAGCGTGTCGTCGCCGCCGACTCCGGCGAAGCCGCCCTGGCCCTGTGGCACG
AGCATGCGTTCGACGTCGTGATCACCGATTGCAACATGCCCGGTATCAACGGCTACGAATTGGCGCGCCGCATACGCGCC
GCCGAGGCCGCGCCCGGTTACGGACGTACGCGGTGCATTCTGTTCGGCTTCACGGCTTCGGCGCAGATGGACGAAGCGCA
GCGCTGCCGCGCCGCCGGCATGGACGACTGCCTGTTCAAGCCGATCGGCGTGGACGCCTTGCGGCAACGCTTGAACGAAG
CCGCGGCACGGGCCGCGCTCCCCACGCCCCCCTCGCCCCAGGCTGCCGCGCCGGCCACGCACGACGCCACCCCGGCGGCG
TTCTCGGCCGAGTCGATTCTTGCCCTGACGCAGAACGATGAGGCGCTGATCCGGCAATTGCTCGAAGAAGTGATTCGCAC
CAACCGGGCGGACGTCGATCAATTGCAGAAGCTGCACCAGCAGGCCGATTGGCCGAAGGTCTCGGACATGGCGCACAGGC
TGGCCGGCGGCGCGCGCGTGGTCGATGCCAAGGCCATGATAGACACTGCGCTGGCGCTGGAGAAAAAAGCGCAAGGCCAG
GCTGGCCCCTCGCCCGAAATCGACGGCATGGTACGTACGCTTGCGGCGCAGTCCGCCGCGCTGGAGACGCAACTACGCGC
CTGGCTGGAGCAACGGCCGCATCAAGGCCAGCCCTGA
>Bb_B2108.seq
ATGCCCGCCCCGCACCGCCTGTACCCCCGCAGTCTGATCTGCCTGGCTCAGGCGCTATTGGCATGGGCTTTGCTGGCATG
GGCGCCCGCGCAGGCAAGCCAGGAGCTGACCCTGGTCGGCAAGGCTGCCGTTCCCGACGTCGAGATCACGCTCGACGGCG
ACGACTGGCGCTGGCTGGCGCGCAAGCGGGTGCTGACGCTGGGCGTGTACGCGCCGGACATTCCCCCGTTCGACGTCACC
TATGACGAGCGCTACGAAGGCCTGACGGCCGACTACATGGCGATCATCGCGCACAACCTGGGCGTCCAGGCAAAAGTGCT
GCGCTACCCCACGCGCGAGCAAGCCGTCGGCGCACTGGAAAGCGGACAGATCGACCTCATCGGCACCGTCAATGGCATCG
AGGGCCGGCTGCAGAGCCTGCGCCTGAGTGTTCCCTACGCGGCCGACCACCCGGTGCTGGTCATGCCCATCGGCGCGCGC
CGCGCTCCGCCCGCGGACCTGGCAGGCCAGCGGTTGGCGGTTGACGCCAACTACTTGCCCAGGGAAACGCTGCAGCAGGC
CTATCCCCAGGCAACGCTGCATTACTTCCCATCATCCGAACAGGCGCTGGCCGCGGTGGCCTATGGACAAGCCGACGTGT
TCATCGGCGATGCGCTGACCACCTCGCACCTCGTTTCGCAAAGCTACTTCAACGACGTTCGCGTCGTCGCCCCGGCCCAG
ATCGTGACGGGCGGGGAATCCTTCGGCGTGCGCGCCGACAATACCCGCCTGCTGCGGGTGGTCAATGCCGTGCTCGAAGC
CATTCCGGCCTCCGAGCGCCGCAGCCTGATCTACCGCTGGGGCCTGGGCAGCAGCATTTCGCTCGATTTCGCGCGCCCCG
CCTATTCAGCGCGCGAGCAGCAGTGGATGGCAAACCATCCGGTCGTCAAGGTGGCGGTCCTGAACCTGTTCGCGCCCTTC
ACCCTGTTTCGCACCGATGAACAGTTCGGCGGCATCAGTGCCGCCGTGCTGCAACTGCTGCAGTTGCGCACCGGCCTGGA
TTTCCAGATCATCGGCGTCGACACGGTCGAGGAGCTGATCGCCAAGCTGCGCTCGGGCGAAGCCGACATGGCCGGCGCCC
TGTTCGTCAATGCCGCGCGGGAATCCGTCCTCAGCTTCAGCCGGCCGTATGTGCGCAATGGTCTGGTGATCGTCACGCGC
CAGGACCCCGCCGCGCCCGCCGACGCCGATCACCTCGACGGCCGCACGATTGCGATGGTGCGCAACAGCGCCGCCATCCC
GCTCCTGCAGCAGCGCTATCCCCAGGCGAAGGTCGTGACCGCCGACAACCCGACCGAAGCCATGCTGCTGGTGGCCGATG
GCCAGGCCGACGCCGTCGTGCAGACGCAGATCAGCGCCAGCTACTACGTCAACCGCTACTTCGCCGGAAAACTGCGCATT
GCCTCGGCGCTGGACCTGCCGCCGGCCGAGATCGCGCTGGCGACGGCGCGCGGCCAGACCGAGCTGATATCCATCCTGAA
CAAGGCGCTCTACAGCATTTCGAACGACGAACTCGCCTCCATCGTCAGCCGCTGGCGCGGCAGCGACGGCGATCCGCGCA
CCTGGTACGCCTACCGCAACGAAATCTACCTGCTGATCGGGCTGGGCCTGTTGTCGGCCCTGCTGTTCCTGAGCTGGATC
GTCTACCTGCGGCGCCAGATCCGCCAGCGCAAGCGGGCCGAGCGGGCGCTGAACGACCAGCTGGAATTCATGCGCGTGCT
CATCGACGGCACGCCCAACCCCATCTATGTGCGCGATAAGGAAGGCCGCATGCTGTTGTGCAATGACGCCTACCTCGACA
CCTTTGGCGTGACTGCCGATGCGGTACTGGGCAAGACCATCCCGGAGGCCAACGTGGTGGGCGACCCGGCGCTGGCTCGC
GAGATGCACGAGTTCCTGCTCACGCGCATGGCCGCCGAGCGCGAGCCGCGCTTCGAGGACCGCGATGTCACGCTGCACGG
CCGCACCCGCCATGTCTACCAGTGGACGGTTCCGTACGGCGACTCGCTGGGCGAACTCAAGGGCATCATCGGCGGCTGGA
TCGACATTACCGAACGCGCCGAGCTGCTGCGCGAGCTGCACGACGCCAAGGAAAGCGCCGACGCCGCCAACCGGGCCAAG
ACCACGTTCCTGGCAACGATGAGCCACGAGATCCGCACGCCGATGAACGCGATCATCGGCATGCTGGAGCTGGCGCTGCT
CCGTCCGGCCGACCAGGAGCCGGACCGCCAGTCCATCCAGGTCGCGTACGACTCGGCCCGCAGCCTGCTGGAGCTGATAG
GCGACATCCTGGACATTGCGAAGATCGAGGCGGGAAAATTCGACCTGGCGCCGGTGCGCACGGCGCTGCGCGCCCTGCCC
GAAGGGGCGATCCGCGTCTTCGACGGGTTGGCGCGCCAGAAAGGCATAGAGCTGGTATTGAAGACCGACATCGTGGGCGT
GGACGATGTATTGATAGACCCCTTGCGCATGAAGCAAGTGCTCTCGAACCTGGTGGGCAACGCCATCAAGTTCACCACCG
AAGGCCAGGTTGTCCTTACCGTGACCGCGCGCCCCGACGGCGAGGCCGCGCACGTGCAGTTCAGCGTGAGCGACACCGGC
TGCGGCATCAGCGAGGCCGACCAACGGCAGCTGTTCAAACCGTTCTCGCAGGTGGGCGGCAGCGCCGAGGCCGGGCCGGC
GCCGGGCACTGGCCTGGGCCTGTCCATCAGCCGCCGCCTCGTCGAATTGATGGGGGGAACGCTGGTCATGCGCAGCGCGC
CAGGGGTGGGCACAACGGTTTCGGTGGACCTGAGGCTGACCATGGTCGAAAAATCCGCGCAGGCCACGCCGCCCGCTGCG
GCCGCTCAGGCCACGCCATCCAAGCCGCAGGTATCGCTGCGCGTGCTGGTCGTCGATGACCACAAGCCCAACCTGATGCT
GCTGCGCCAGCAGCTGGACTACCTGGGCCAGCGTGTCGTCGCCGCCGACTCCGGCGAAGCCGCCCTGGCCCTGTGGCACG
AGCATGCGTTCGACGTCGTGATCACCGATTGCAACATGCCCGGTATCAACGGCTACGAATTGGCGCGCCGCATACGCGCC
GCCGAGGCCGCGCCCGGTTACGGACGTACGCGGTGCATTCTGTTCGGCTTCACGGCTTCGGCGCAGATGGACGAAGCGCA
GCGCTGCCGCGCCGCCGGCATGGACGACTGCCTGTTCAAGCCGATCGGCGTGGACGCCTTGCGGCAACGCTTGAACGAAG
CCGCGGCACGGGCCGCGCTCCCCACGCCCCCCTCGCCCCAGGCTGCCGCGCCGGCCACGCACGACGCCACCCCGGCGGCG
TTCTCGGCCGAGTCGATTCTTGCCCTGACGCAGAACGATGAGGCGCTGATCCGGCAATTGCTCGAAGAAGTGATTCGCAC
CAACCGGGCGGACGTCGATCAATTGCAGAAGCTGCACCAGCAGGCCGATTGGCCGAAGGTCTCGGACATGGCGCACAGGC
TGGCCGGCGGCGCGCGCGTGGTCGATGCCAAGGCCATGATAGACACTGCGCTGGCGCTGGAGAAAAAAGCGCAAGGCCAG
GCTGGCCCCTCGCCCGAAATCGACGGCATGGTACGTACGCTTGCGGCGCAGTCCGCCGCGCTGGAGACGCAACTACGCGC
CTGGCTGGAGCAACGGCCGCATCAAGGCCAGCCCTGA
>Bb_B2112.seq
ATGCCCGCCCCGCACCGCCTGTACCCCCGCAGTCTGATCTGCCTGGCTCAGGCGCTATTGGCATGGGCTTTGCTGGCATG
GGCGCCCGCGCAGGCAAGCCAGGAGCTGACCCTGGTCGGCAAGGCTGCCGTTCCCGACGTCGAGATCACGCTCGACGGCG
ACGACTGGCGCTGGCTGGCGCGCAAGCGGGTGCTGACGCTGGGCGTGTACGCGCCGGACATTCCCCCGTTCGACGTCACC
TATGACGAGCGCTACGAAGGCCTGACGGCCGACTACATGGCGATCATCGCGCACAACCTGGGCGTCCAGGCAAAAGTGCT
GCGCTACCCCACGCGCGAGCAAGCCGTCGGCGCACTGGAAAGCGGACAGATCGACCTCATCGGCACCGTCAATGGCATCG
AGGGCCGGCTGCAGAGCCTGCGCCTGAGTGTTCCCTACGCGGCCGACCACCCGGTGCTGGTCATGCCCATCGGCGCGCGC
CGCGCTCCGCCCGCGGACCTGGCAGGCCAGCGGTTGGCGGTTGACGCCAACTACTTGCCCAGGGAAACGCTGCAGCAGGC
CTATCCCCAGGCAACGCTGCATTACTTCCCATCATCCGAACAGGCGCTGGCCGCGGTGGCCTATGGACAAGCCGACGTGT
TCATCGGCGATGCGCTGACCACCTCGCACCTCGTTTCGCAAAGCTACTTCAACGACGTTCGCGTCGTCGCCCCGGCCCAG
ATCGTGACGGGCGGGGAATCCTTCGGCGTGCGCGCCGACAATACCCGCCTGCTGCGGGTGGTCAATGCCGTGCTCGAAGC
CATTCCGGCCTCCGAGCGCCGCAGCCTGATCTACCGCTGGGGCCTGGGCAGCAGCATTTCGCTCGATTTCGCGCGCCCCG
CCTATTCAGCGCGCGAGCAGCAGTGGATGGCAAACCATCCGGTCGTCAAGGTGGCGGTCCTGAACCTGTTCGCGCCCTTC
ACCCTGTTTCGCACCGATGAACAGTTCGGCGGCATCAGTGCCGCCGTGCTGCAACTGCTGCAGTTGCGCACCGGCCTGGA
TTTCCAGATCATCGGCGTCGACACGGTCGAGGAGCTGATCGCCAAGCTGCGCTCGGGCGAAGCCGACATGGCCGGCGCCC
TGTTCGTCAATGCCGCGCGGGAATCCGTCCTCAGCTTCAGCCGGCCGTATGTGCGCAATGGTCTGGTGATCGTCACGCGC
CAGGACCCCGCCGCGCCCGCCGACGCCGATCACCTCGACGGCCGCACGATTGCGATGGTGCGCAACAGCGCCGCCATCCC
GCTCCTGCAGCAGCGCTATCCCCAGGCGAAGGTCGTGACCGCCGACAACCCGACCGAAGCCATGCTGCTGGTGGCCGATG
GCCAGGCCGACGCCGTCGTGCAGACGCAGATCAGCGCCAGCTACTACGTCAACCGCTACTTCGCCGGAAAACTGCGCATT
GCCTCGGCGCTGGACCTGCCGCCGGCCGAGATCGCGCTGGCGACGGCGCGCGGCCAGACCGAGCTGATATCCATCCTGAA
CAAGGCGCTCTACAGCATTTCGAACGACGAACTCGCCTCCATCGTCAGCCGCTGGCGCGGCAGCGACGGCGATCCGCGCA
CCTGGTACGCCTACCGCAACGAAATCTACCTGCTGATCGGGCTGGGCCTGTTGTCGGCCCTGCTGTTCCTGAGCTGGATC
GTCTACCTGCGGCGCCaGATCCGCCAGCGCAAGCGGGCCGAGCGGGCGCTGAACGACCAGCTGGAATTCATGCGCGTGCT
CATCGACGGCACGCCCAACCCCATCTATGTGCGCGATAAGGAAGGCCGCATGCTGTTGTGCAATGACGCCTACCTCGACA
CCTTTGGCGTGACTGCCGATGCGGTACTGGGCAAGACCATCCCGGAGGCCAACGTGGTGGGCGACCCGGCGCTGGCTCGC
GAGATGCACGAGTTCCTGCTCACGCGCATGGCCGCCGAGCGCGAGCCGCGCTTCGAGGACCGCGATGTCACGCTGCACGG
CCGCACCCGCCATGTCTACCAGTGGACGGTTCCGTACGGCGACTCGCTGGGCGAACTCAAGGGCATCATCGGCGGCTGGA
TCGACATTACCGAACGCGCCGAGCTGCTGCGCGAGCTGCACGACGCCAAGGAAAGCGCCGACGCCGCCAACCGGGCCAAG
ACCACGTTCCTGGCAACGATGAGCCACGAGATCCGCACGCCGATGaACGCGATCATCGGCATGCTGGAGCTGGCGCTGCT
CCGTCCGGCCGACCAGGAGCCGGACCGCCAGTCCATCCAGGTCGCGTACGACTCGGCCCGCAGCCTGCTGGAGCTGATAG
GCGACATCCTGGACATTGCGAAGATCGAGGCGGGAAAATTCGACCTGGCGCCGGTGCGCACGGCGCTGCGCGCCCTGCCC
GAAGGGGCGATCCGCGTCTTCGACGGGTTGGCGCGCCAGAAAGGCATAGAGCTGGTATTGAAGACCGACATCGTGGGCGT
GGACGATGTATTGATAGACCCCTTGCGCATGAAGCAAGTGCTCTCGAACCTGGTGGGCAACGCCATCAAGTTCACCACCG
AAGGCCAGGTTGTCCTTACCGTGACCGCGCGCCCCGACGGCGAGGCCGCGCACGTGCAGTTCAGCGTGAGCGACACCGGC
TGCGGCATCAGCGAGGCCGACCAACGGCAGCTGTTCAAACCGTTCTCGCAGGTGGGCGGCAGCGCCGAGGCCGGGCCGGC
GCCGGGCACTGGCCTGGGCCTGTCCATCAGCCGCCGCCTCGTCGAATTGATGGGGGGAACGCTGGTCATGCGCAGCGCGC
CAGGGGTGGGCACAACGGTTTCGGTGGACCTGAGGCTGACCATGGTCGAAAAATCCGCGCAGGCCACGCCGCCCGCTGCG
GCCGCTCAGGCCACGCCATCCAAGCCGCAGGTATCGCTGCGCGTGCTGGTCGTCGATGACCACAAGCCCAACCTGATGCT
GCTGCGCCAGCAGCTGGACTACCTGGGCCAGCGTGTCGTCGCCGCCGACTCCGGCGAAGCCGCCCTGGCCCTGTGGCACG
AGCATGCGTTCGACGTCGTGATCACCGATTGCAACATGCCCGGTATCAACGGCTACGAATtGGCGCGCCGCATACGCGCC
GCCGAGGCCGCGCCCGGTTACGGACGTACGCGGTGCATTCTGTTCGGCTTCACGGCTTCGGCGCAGATGGACGAAGCGCA
GCGCTGCCGCGCCGCCGGCATGGACGACTGCCTGTTCAAGCCGATCGGCGTGGACGCCTTGCGGCAACGCTTGAACGAAG
CCGCGGCACGGGCCGCGCTCCCCACGCCCCCCTCGCCCCAGGCTGCCGCGCCGGCCACGCACGACGCCACCCCGGCGGCG
TTCTCGGCCGAGTCGATTCTTGCCCTGACGCAGAACGATGAGGCGCTGATCCGGCAATTGCTCGAAGAAGTGATTCGCAC
CAACCGGGCGGACGTCGATCAATTGCAGAAGCTGCACCAGCAGGCCGATTGGCCGAAGGTCTCGGACATGGCGCACAGGC
TGGCCGGCGGCGCGCGCGTGGTCGATGCCAAGGCCATGATAGACACTGCGCTGGCGCTGGAGAAAAAAGCGCAAGGCCAG
GCTGGCCCCTCGCCCGAAATCGACGGCATGGTACGTACGCTTGCGGCGCAGTCCGCCGCGCTGGAGACGCAACTACGCGC
CTGGCTGGAGCAACGGCCGCATCAAGGCCAGCCCTGA
>Bb_B2114.seq
ATGCCCGCCCCGCACCGCCTGTACCCCCGCAGTCTGATCTGCCTGGCTCAGGCGCTATTGGCATGGGCTTTGCTGGCATG
GGCGCCCGCGCAGGCAAGCCAGGAGCTGACCCTGGTCGGCAAGGCTGCCGTTCCCGACGTCGAGGTCGCGCTCGACGGCG
ACGACTGGCGTTGGCTGGCCCGCAAGCGGGTACTGACGCTGGGTGTGTACGCACCGGACATTCCTCCGTTCGACGTCACC
TATGGCGAACGCTACGAAGGCCTGACGGCCGACTACATGGCGATCATCGCGCACAACCTGGGGATGCAGGCGAAAGTGCT
GCGATACCCCACGCGCGAACAAGCCCTCGGCGCGCTGGAAAGCGGGCAGATCGACCTCATCGGCACCGTCAATGGCACCG
ACGGCCGGCAACAGAGCCTGCGTCTGAGCGTTCCCTACGCCGCCGACCACCCGGTGATCGTCATGCCCATCGGCGCACGC
CACGTTCCAGCCTCGAACCTGGCCGGCCAGCGGCTGGCGGTCGACATCAACTACCTGCCCAAGGAAACGCTCGCACGGGC
CTACCCGCAGGCAACGCTGCATTACTTTTCCTCATCCGAGCAGGCGCTGGCCGCGGTGGCCTATGGGCAGGCCGACGTAT
TCATCGGCGATGCCCTGACCACCTCGCACCTCGTATCGCAAAGCTATTTCAATGACGTTCGCGTGGTCGCCCCGGCCCAT
ATCGCGACGGGCGGAGAATCCTTCGGCGTGCGCGCCGACAACACCCGCCTGCTGCGGGTGGTCAACGCCGTACTCGAAGC
CATTCCGCCTTCCGAACACCGCAGCCTGATCTACCGCTGGGGACTGGGCAGCAGCATTTCGCTCGATTTCGCGCACCCCG
CGTATTCCGCGCGCGAGCAGCAATGGATGGCAGACCACCCCGTCGTCAAGGTGGCGGTCCTGAATCTGTTCGCGCCCTTC
ACCCTGTTCCGCACCGACGAACAGTTCGGCGGGATCAGCGCCGCCGTGCTGCAGCTGCTGCAATTGCGCACCGGCCTGGA
CTTCGAGATCATCGGCGTCGACACGGTCGAGGAACTGATAGCCAAGCTGCGTTCGGGCGAAGCCGACATGGCCGGCGCCC
TGTTCGTCAACAGCGCGCGGGAGTCCTTCCTCAGTTTCAGCCGGCCGTATGTGCGCAATGGCATGGTGATCGTCACGCGC
CAGGACCCCGACGCGCCCGTCGACGCCGATCATCTGGACGGCCGCACGGTCGCGTTGGTGCGCAACAGCGCCGCCATTCC
CCTGCTGCAGCGGCGCTATCCCCAGGCGAAGGTGGTGACCGCCGACAACCCGAGCGAGGCGATGCTGATGGTGGCCAATG
GACAGGCCGACGCCGTCGTGCAGACGCAGATCAGCGCCAGCTATTACGTCAACCGCTACTTCGCCGGCAAGCTGCGCATC
GCCTCGGCGCTGGACCTGCCTCCGGCCGAGATCGCGCTGGCGACGACGCGCGGCCAGACCGAACTGATGTCCATCCTGAA
CAAGGCGCTCTACAGCATTTCGAACGACGAGCTCGCCTCCATCATCAGCCGCTGGCGCGGCAGCGACGGCGATCCGCGCA
CCTGGTACGCCTACCGCAACGAGATCTACCTGCTGATCGGGCTGGGCCTGTTGTCGGCCCTGCTGTTCCTGAGCTGGATC
GTCTACCTGCGGCGCCaGATCCGCCAGCGCAAGCGGGCCGAGCGGGCGCTGAACGACCAGCTGGAATTCATGCGCGTGCT
CATCGACGGCACGCCCAACCCCATCTATGTGCGCGATAAGGAAGGCCGCATGCTGTTGTGCAATGACGCCTACCTCGACA
CCTTTGGCGTGACTGCCGATGCGGTACTGGGCAAGACCATTCCGGAAGCCAACGTGGTGGGCGACCCGGCGCTGGCCCGC
GAAATGCACGAGTTCCTGCTCACCCGCGTGGCCGCCGAGCGCGAGCCGCGCTTCGAGGACCGCGATGTCACGCTGCACGG
CCGCACCCGCCATGTCTACCAGTGGACGATTCCGTACGGCGACTCGCTGGGCGAACTCAAGGGCATCATCGGCGGCTGGA
TCGACATCACCGAACGCGCCGAGCTGCTGCGCGAGCTGCACGACGCCAAGGAAAGCGCCGACGCCGCCAACCGGGCCAAG
ACCACGTTCCTGGCAACGATGAGCCACGAGATCCGCACGCCGATGAACGCGATCATCGGCATGCTGGAGCTGGCGCTGCT
CCGTCCGGCCGACCAGGAGCCGGATCGCCAGTCCATCCAGGTCGCGTACGACTCGGCCCGCAGCCTGCTGGAGCTGATAG
GCGACATCCTGGACATTGCGAAGATCGAGGCGGGAAAATTCGACCTGGCGCCGGTGCGCACGGCGCTGCGCGCCCTGCCC
GAAGGGGCGATCCGCGTCTTCGACGGATTGGCGCGCCAGAAAGGCATAGAGCTGGTATTGAAGACCGACATCGTGGGCGT
CGACGATGTATTGATAGACCCCTTGCGCATGAAGCAAGTGCTCTCGAACCTGGTGGGCAACGCCATCAAGTTCACCACCG
AAGGCCAGGTTGTCCTTGCCGTGACCGCACGCCCCGACGGCGAGGCCGCGCACGTGCAGTTCAGCGTGAGCGACACCGGC
TGCGGCATCAGCGAGGCCGACCAACGGCAGCTGTTCAAACCGTTCTCGCAGGTGGGTGGCAACGCCGAGGCCGGGCCGGC
GCCGGGCACCGGCCTGGGCCTGTCCATCAGCCGCCGCCTCGTCGAATTGATGGGGGGAACGCTGGTCATGCGCAGCGCGC
CAGGGGTGGGCACAACGGTTTCGGTGGACCTGAGGCTGACCATGGTCGAAAAATCCGTGCAGGCCACGCCGCCCGCTGCG
GCCGCTGCGGCCACGCCGTCCAAGCCGCAGGTATCGCTGCGCGTGCTGGTCGTCGATGACCACAAACCCAACCTGATGCT
GCTGCGCCAGCAGCTGGACTACCTGGGCCAGCGTGTCATCGCCGCCGACTCCGGCGAAGCCGCCCTGGCCCTGTGGCGCG
AGCATGCGTTCGACGTCgTGATCACCGATTgCAACATGCCCGGTATCAGCGGCTACGAAtTGGCGCGCCGCATACGCGCC
GCCGAGGCCGCGCCCGGTTACGGACGTACGCGGTGCATTCTGTTCGGCTTCACGGCTTCGGCGCAGATGGACGAAGCGCA
GCGCTGCCGCGCCGCCGGCATGGACGACTGCCTGTTCAAGCCGATCGGCGTGGACGCCTTGCGGCAACGCCTGAACGAAG
CCGTGGCACGGGCCGCGCTCCCCACGCCCCCCTCGCCCCAGGCTGCCGCGCCGGCCACGGACGACGCCACCCCGGCGGCG
TTCTCGGCCGAGTCGATTCTTGCCTTGACGCAGAACGATGAGGCGCTGATCCGGCAATTGCTCGAAGAAGTGATTCGCAC
CAACCGGGCGGACGTCGACCAATTGCAAAAGCTGCACCAGCAGGCCGATTGGCCGAAGGTCTCGGACATGGCGCACAGGC
TGGCCGGCGGCGCGCGCGTGGTCGATGCCAAGGCCATGATAGACACTGCGCTGGCGCTGGAGAAAAAAGCGCAAGGCCAG
GCTGGCCCCTCGCCCGAAATCGACGGCCTGGTACGTACGCTTGCGGCGCAGTCCGCCGCGCTGGAGACGCAACTGCGCGC
CTGGCTGGAGCAACGGCCGCATCAAGATCAGCCCTGA
>Bb_B2115.seq
ATGCCCGCCCCGCACCGCCTGTACCCCCGCAGTCTGATCTGCCTGGCTCAGGCGCTATTGGCATGGGCTTTGCTGGCATG
GGCGCCCGCGCAGGCAAGCCAGGAGCTGACCCTGGTCGGCAAGGCTGCCGTTCCCGACGTCGAGATCACGCTCGACGGCG
ACGACTGGCGCTGGCTGGCGCGCAAGCGGGTGCTGACGCTGGGCGTGTACGCGCCGGACATTCCCCCGTTCGACGTCACC
TATGACGAGCGCTACGAAGGCCTGACGGCCGACTACATGGCGATCATCGCGCACAACCTGGGCGTCCAGGCAAAAGTGCT
GCGCTACCCCACGCGCGAGCAAGCCGTCGGCGCACTGGAAAGCGGACAGATCGACCTCATCGGCACCGTCAATGGCATCG
AGGGCCGGCTGCAGAGCCTGCGCCTGAGTGTTCCCTACGCGGCCGACCACCCGGTGCTGGTCATGCCCATCGGCGCGCGC
CGCGCTCCGCCCGCGGACCTGGCAGGCCAGCGGTTGGCGGTTGACGCCAACTACTTGCCCAGGGAAACGCTGCAGCAGGC
CTATCCCCAGGCAACGCTGCATTACTTCCCATCGTCCGAACAGGCGCTGGCCGCGGTGGCCTATGGACAAGCCGACGTGT
TCATCGGCGATGCGCTGACCACCTCGCACCTCGTTTCGCAAAGCTACTTCAACGACGTTCGCGTCGTCGCCCCGGCCCAG
ATCGTGACGGGCGGGGAATCCTTCGGCGTGCGCGCCGACAATACCCGCCTGCTGCGGGTGGTCAATGCCGTGCTCGAAGC
CATTCCGGCCTCCGAGCGCCGCAGCCTGATCTACCGCTGGGGCCTGGGCAGCAGCATTTCGCTCGATTTCGCGCGCCCCG
CCTATTCAGCGCGCGAGCAGCAGTGGATGGCAAACCATCCGGTCGTCAAGGTGGCGGTCCTGAACCTGTTCGCGCCCTTC
ACCCTGTTTCGCACCGATGAACAGTTCGGCGGCATCAGTGCCGCCGTGCTGCAACTGCTGCAGTTGCGCACCGGCCTGGA
TTTCCAGATCATCGGCGTCGACACGGTCGAGGAGCTGATCGCCAAGCTGCGCTCGGGCGAAGCCGACATGGCCGGCGCCC
TGTTCGTCAATGCCGCGCGGGAATCCGTCCTCAGCTTCAGCCGGCCGTATGTGCGCAATGGCATGGTGATCGTCACGCGC
CAGGACCCCGCCGCGCCCGCCGACGCCGATCACCTCGACGGCCGCACGATTGCGATGGTGCGCAACAGCGCCGCCATCCC
GCTCCTGCAGCAGCGCTATCCCCAGGCGAAGGTCGTGACCGCCGACAACCCGACCGAAGCCATGCTGCTGGTGGCCGATG
GCCAGGCCGACGCCGTCGTGCAGACGCAGATCAGCGCCAGCTACTACGTCAACCGCTACTTCGCCGGAAAACTGCGCATT
GCCTCGGCGCTGGACCTGCCGCCGGCCGAGATCGCGCTGGCGACGGCGCGCGGCCAGACCGAGCTGATATCCATCCTGAA
CAAGGCGCTCTACAGCATTTCGAACGACGAACTCGCCTCCATCGTCAGCCGCTGGCGCGGCAGCGACGGCGATCCGCGCA
CCTGGTACGCCTACCGCAACGAGATCTACCTGCTGATCGGGCTGGGCCTGTTGTCGGCCCTGCTGTTCCTGAGCTGGATC
GTCTACCTGCGGCGCCAGATCCGCCAGCGCAAGCGGGCCGAGCGGGCGCTGAACGACCAGCTGGAATTCATGCGCGTGCT
CATCGACGGCACGCCCAACCCCATCTATGTGCGCGATAAGGAAGGCCGCATGCTGTTGTGCAATGACGCCTACCTCGACA
CCTTTGGCGTGACTGCCGATGCGGTACTGGGCAAGACCATCCCGGAGGCCAACGTGGTGGGCGACCCGGCGCTGGCTCGC
GAGATGCACGAGTTCCTGCTCACGCGCATGTCCGCCGAGCGCGAGCCGCGCTTCGAGGACCGCGATGTCACGCTGCACGG
CCGCACCCGCCATGTCTACCAGTGGACGGTTCCGTACGGCGACTCGCTGGGCGAACTCAAGGGCATCATCGGCGGCTGGA
TCGACATTACCGAACGCGCCGAGCTGCTGCGCGAGCTGCACGACGCCAAGGAAAGCGCCGACGCCGCCAACCGGGCCAAG
ACCACGTTCCTGGCAACGATGAGCCACGAGATCCGCACGCCGATGAACGCGATCATCGGCATGCTGGAGCTGGCGCTGCT
CCGTCCGGCCGACCAGGAGCCGGACCGCCAGTCCATCCAGGTCGCGTACGACTCGGCCCGCAGCCTGCTGGAGCTGATAG
GCGACATCCTGGACATTGCGAAGATCGAGGCGGGAAAATTCGACCTGGCGCCGGTGCGCACGGCGCTGCGCGCCCTGCCC
GAAGGGGCGATCCGCGTCTTCGACGGGTTGGCGCGCCAGAAAGGCATAGAGCTGGTATTGAAGACCGACATCGTGGGCGT
GGACGATGTATTGATAGACCCCTTGCGCATGAAGCAAGTGCTCTCGAACCTGGTGGGCAACGCCATCAAGTTCACCACCG
AAGGCCAGGTTGTCCTTACCGTGACCGCGCGCCCCGACGGCGAGGCCGCGCACGTGCAGTTCAGCGTGAGCGACACCGGC
TGCGGCATCAGCGAGGCCGACCAACGGCAGCTGTTCAAACCGTTCTCGCAGGTGGGCGGCAGCGCCGAGGCCGGGCCGGC
GCCGGGCACTGGCCTGGGCCTGTCCATCAGCCGCCGCCTCGTCGAATTGATGGGGGGAACGCTGGTCATGCGCAGCGCGC
CAGGGGTGGGCACAACGGTTTCGGTGGACCTGAGGCTGACCATGGTCGAAAAATCCGCGCAGGCCACGCCGCCCGCTGCG
GCCGCTCAGGCCACGCCATCCAAGCCGCAGGTATCGCTGCGCGTGCTGGTCGTCGATGACCACAAGCCCAACCTGATGCT
GCTGCGCCAGCAGCTGGACTACCTGGGCCAGCGTGTCGTCGCCGCCGACTCCGGCGAAGCCGCCCTGGCCCTGTGGCACG
AGCATGCGTTCGACGTCGTGATCACCGATTGCAACATGCCCGGTATCAACGGCTACGAATtGGCGCGCCGCATACGCGCC
GCCGAGGCCGCGCCCGGTTACGGACGTACGCGGTGCATTCTGTTCGGCTTCACGGCTTCGGCGCAGATGGACGAAGCGCA
GCGCTGCCGCGCCGCCGGCATGGACGACTGCCTGTTCAAGCCGATCGGCGTGGACGCCTTGCGGCAACGCTTGAACGAAG
CCGCGGCACGGGCCGCGCTCCCCACGCCCCCCTCGCCCCAGGCTGCCGCGCCGGCCACGCACGACGCCACCCCGGCGGCG
TTCTCGGCCGAGTCGATTCTTGCCCTGACGCAGAACGATGAGGCGCTCATCCGGCAATTGCTCGAAGAACTGATTCGCAC
CAACCGGGCGGACGTCGATCAATTGCAGAAGCTGCACCAGCAGGCCGATTGGCCGAAGGTCTCGGACATGGCGCACAGGC
TGGCCGGCGGCGCGCGCGTGGTCGATGCCAAGGCCATGATAGACACTGCGCTGGCGCTGGAGAAAAAAGCGCAAGGCCAG
GCTGGCCCCTCGCCCGAAATCGACGGCATGGTACGTACGCTTGCGGCGCAGTCCGCCGCGCTGGAGACGCAACTACGCGC
CTGGCTGGAGCAACGGCCGCATCAAGGCCAGCCCTGA
>Bb_B2116.seq
ATGCCCGCCCCGCACCGCCTGTACCCCCGCAGTCTGATCTGCCTGGCTCAGGCGCTATTGGCATGGGCTTTGCTGGCATG
GGCGCCCGCGCAGGCAAGCCAGGAGCTGACCCTGGTCGGCAAGGCTGCCGTTCCCGACGTCGAGATCACGCTCGACGGCG
ACGACTGGCGCTGGCTGGCGCGCAAGCGGGTGCTGACGCTGGGCGTGTACGCGCCGGACATTCCCCCGTTCGACGTCACC
TATGACGAGCGCTACGAAGGCCTGACGGCCGACTACATGGCGATCATCGCGCACAACCTGGGCGTCCAGGCAAAAGTGCT
GCGCTACCCCACGCGCGAGCAAGCCGTCGGCGCACTGGAAAGCGGACAGATCGACCTCATCGGCACCGTCAATGGCATCG
AGGGCCGGCTGCAGAGCCTGCGCCTGAGTGTTCCCTACGCGGCCGACCACCCGGTGCTGGTCATGCCCATCGGCGCGCGC
CGCGCTCCGCCCGCGGACCTGGCAGGCCAGCGGTTGGCGGTTGACGCCAACTACTTGCCCAGGGAAACGCTGCAGCAGGC
CTATCCCCAGGCAACGCTGCATTACTTCCCATCATCCGAACAGGCGCTGGCCGCGGTGGCCTATGGACAAGCCGACGTGT
TCATCGGCGATGCGCTGACCACCTCGCACCTCGTTTCGCAAAGCTACTTCAACGACGTTCGCGTCGTCGCCCCGGCCCAG
ATCGTGACGGGCGGGGAATCCTTCGGCGTGCGCGCCGACAATACCCGCCTGCTGCGGGTGGTCAATGCCGTGCTCGAAGC
CATTCCGGCCTCCGAGCGCCGCAGCCTGATCTACCGCTGGGGCCTGGGCAGCAGCATTTCGCTCGATTTCGCGCGCCCCG
CCTATTCAGCGCGCGAGCAGCAGTGGATGGCAAACCATCCGGTCGTCAAGGTGGCGGTCCTGAACCTGTTCGCGCCCTTC
ACCCTGTTTCGCACCGATGAACAGTTCGGCGGCATCAGTGCCGCCGTGCTGCAACTGCTGCAGTTGCGCACCGGCCTGGA
TTTCCAGATCATCGGCGTCGACACGGTCGAGGAGCTGATCGCCAAGCTGCGCTCGGGCGAAGCCGACATGGCCGGCGCCC
TGTTCGTCAATGCCGCGCGGGAATCCGTCCTCAGCTTCAGCCGGCCGTATGTGCGCAATGGTCTGGTGATCGTCACGCGC
CAGGACCCCGCCGCGCCCGCCGACGCCGATCACCTCGACGGCCGCACGATTGCGATGGTGCGCAACAGCGCCGCCATCCC
GCTCCTGCAGCAGCGCTATCCCCAGGCGAAGGTCGTGACCGCCGACAACCCGACCGAAGCCATGCTGCTGGTGGCCGATG
GCCAGGCCGACGCCGTCGTGCAGACGCAGATCAGCGCCAGCTACTACGTCAACCGCTACTTCGCCGGAAAACTGCGCATT
GCCTCGGCGCTGGACCTGCCGCCGGCCGAGATCGCGCTGGCGACGGCGCGCGGCCAGACCGAGCTGATATCCATCCTGAA
CAAGGCGCTCTACAGCATTTCGAACGACGAACTCGCCTCCATCGTCAGCCGCTGGCGCGGCAGCGACGGCGATCCGCGCA
CCTGGTACGCCTACCGCAACGAAATCTACCTGCTGATCGGGCTGGGCCTGTTGTCGGCCCTGCTGTTCCTGAGCTGGATC
GTCTACCTGCGGCGCCAGATCCGCCAGCGCAAGCGGGCCGAGCGGGCGCTGAACGACCAGCTGGAATTCATGCGCGTGCT
CATCGACGGCACGCCCAACCCCATCTATGTGCGCGATAAGGAAGGCCGCATGCTGTTGTGCAATGACGCCTACCTCGACA
CCTTTGGCGTGACTGCCGATGCGGTACTGGGCAAGACCATCCCGGAGGCCAACGTGGTGGGCGACCCGGCGCTGGCTCGC
GAGATGCACGAGTTCCTGCTCACGCGCATGGCCGCCGAGCGCGAGCCGCGCTTCGAGGACCGCGATGTCACGCTGCACGG
CCGCACCCGCCATGTCTACCAGTGGACGGTTCCGTACGGCGACTCGCTGGGCGAACTCAAGGGCATCATCGGCGGCTGGA
TCGACATTACCGAACGCGCCGAGCTGCTGCGCGAGCTGCACGACGCCAAGGAAAGCGCCGACGCCGCCAACCGGGCCAAG
ACCACGTTCCTGGCAACGATGAGCCACGAGATCCGCACGCCGATGAACGCGATCATCGGCATGCTGGAGCTGGCGCTGCT
CCGTCCGGCCGACCAGGAGCCGGACCGCCAGTCCATCCAGGTCGCGTACGACTCGGCCCGCAGCCTGCTGGAGCTGATAG
GCGACATCCTGGACATTGCGAAGATCGAGGCGGGAAAATTCGACCTGGCGCCGGTGCGCACGGCGCTGCGCGCCCTGCCC
GAAGGGGCGATCCGCGTCTTCGACGGGTTGGCGCGCCAGAAAGGCATAGAGCTGGTATTGAAGACCGACATCGTGGGCGT
GGACGATGTATTGATAGACCCCTTGCGCATGAAGCAAGTGCTCTCGAACCTGGTGGGCAACGCCATCAAGTTCACCACCG
AAGGCCAGGTTGTCCTTACCGTGACCGCGCGCCCCGACGGCGAGGCCGCGCACGTGCAGTTCAGCGTGAGCGACACCGGC
TGCGGCATCAGCGAGGCCGACCAACGGCAGCTGTTCAAACCGTTCTCGCAGGTGGGCGGCAGCGCCGAGGCCGGGCCGGC
GCCGGGCACTGGCCTGGGCCTGTCCATCAGCCGCCGCCTCGTCGAATTGATGGGGGGAACGCTGGTCATGCGCAGCGCGC
CAGGGGTGGGCACAACGGTTTCGGTGGACCTGAGGCTGACCATGGTCGAAAAATCCGCGCAGGCCACGCCGCCCGCTGCG
GCCGCTCAGGCCACGCCATCCAAGCCGCAGGTATCGCTGCGCGTGCTGGTCGTCGATGACCACAAGCCCAACCTGATGCT
GCTGCGCCAGCAGCTGGACTACCTGGGCCAGCGTGTCGTCGCCGCCGACTCCGGCGAAGCCGCCCTGGCCCTGTGGCACG
AGCATGCGTTCGACGTCGTGATCACCGATtGCAACATGCCCGGTATCAACGGCTACGAATtGGCGCGCCGCATACGCGCC
GCCGAGGCCGCGCCCGGTTACGGACGTACGCGGTGCATTCTGTTCGGCTTCACGGCTTCGGCGCAGATGGACGAAGCGCA
GCGCTGCCGCGCCGCCGGCATGGACGACTGCCTGTTCAAGCCGATCGGCGTGGACGCCTTGCGGCAACGCTTGAACGAAG
CCGCGGCACGGGCCGCGCTCCCCACGCCCCCCTCGCCCCAGGCTGCCGCGCCGGCCACGCACGACGCCACCCCGGCGGCG
TTCTCGGCCGAGTCGATTCTTGCCCTGACGCAGAACGATGAGGCGCTGATCCGGCAATTGCTCGAAGAAGTGATTCGCAC
CAACCGGGCGGACGTCGATCAATTGCAGAAGCTGCACCAGCAGGCCGATTGGCCGAAGGTCTCGGACATGGCGCACAGGC
TGGCCGGCGGCGCGCGCGTGGTCGATGCCAAGGCCATGATAGACACTGCGCTGGCGCTGGAGAAAAAAGCGCAAGGCCAG
GCTGGCCCCTCGCCCGAAATCGACGGCATGGTACGTACGCTTGCGGCGCAGTCCGCCGCGCTGGAGACGCAACTACGCGC
CTGGCTGGAGCAACGGCCGCATCAAGGCCAGCCCTGA
>Bb_B2490.seq
ATGCCCGCCCCGCACCGCCTGTACCCCCGCAGTCTGATCTGCCTGGCTCAGGCGCTATTGGCATGGGCTTTGCTGGCATG
GGCGCCCGCGCAGGCAAGCCAGGAGCTGGCCCTGGTCGGCAAGGCTGCCGTTCCCGACGTCGAGGTCGCACTCGACGGCG
ACGACTGGCGTTGGCTGGCCCGCAAGCGGGTACTGACGCTGGGTGTGTACGCACCGGACATTCCTCCGTTCGACGTCACC
TATGGCGAACGCTACGAAGGCCTGACGGCCGACTACATGGCGATCATCGCGCACAATCTGGGGATGCAGGCGAAAGTGCT
GCGATACCCCACGCGCGAACAAGCCCTCGGCGCGCTGGAAAGCGGGCAGATCGACCTCATCGGCACCGTCAATGGCACCG
ACGGCCGGCAACAGAGCCTGCGTCTGAGCGTTCCCTACGCCGCCGACCACCCGGTGATCGTCATGCCCATCGGCGCACGC
CACGTTCCAGCCTCGAACCTGGCCGGCCAGCGGCTGGCGGTCGACATCAACTACCTGCCCAAGGAAACGCTCGCACGGGC
CTACCCGCAGGCTACGCTGCATTACTTTCCCTCATCCGAACAGGCGCTGGCCGCGGTGGCCTATGGGCAGGCCGACGTAT
TCATCGGCGATGCCCTGACCACCTCGCACCTCGTATCGCAAAGCTATTTCAATGACGTTCGCGTAGTCGCCCCGGCCCAT
ATCGCGACGGGCGGAGAATCCTTCGGCGTGCGCGCCGACAACACCCGCCTGCTGCGGGTGGTCAACGCCGTACTCGAAGC
CATTCCGCCTTCCGAACACCGCAGCCTGATCTACCGCTGGGGACTGGGCAGCAGCATTTCGCTCGATTTCGCGCACCCCG
CGTATTCCGCGCGCGAGCAGCAATGGATGGCAGACCACCCCGTCGTCAAGGTGGCGGTCCTGAATCTGTTCGCGCCCTTC
ACCCTGTTCCGCACCGACGAACAGTTCGGCGGGATCAGCGCCGCCGTGCTGCAGCTGCTGCAATTGCGCACCGGCCTGGA
CTTCGAGATCATCGGCGTCGACACGGTCGAGGAACTGATAGCCAAGCTGCGTTCGGGCGAAGCCGACATGGCCGGCGCCC
TGTTCGTCAACAGCGCGCGGGAGTCCTTCCTCAGTTTCAGCCGGCCGTATGTGCGCAATGGCATGGTGATCGTCACGCGC
CAGGACCCCGACGCGCCCGTCGACGCCGATCATCTGGACGGCCGCACGGTCGCGTTGGTGCGCAACAGCGCCGCCATTCC
CCTGCTGCAGCGGCGCTATCCCCAGGCGAAGGTGGTGACCGCCGACAACCCGAGCGAGGCGATGCTGATGGTGGCCAATG
GACAGGCCGACGCCGTCGTGCAGACGCAGATCAGCGCCAGCTATTACGTCAACCGCTACTTCGCCGGCAAGCTGCGCATC
GCCTCGGCGCTGGACCTGCCTCCGGCCGAGATCGCGCTGGCGACGACGCGCGGCCAGACCGAACTGATGTCCATCCTGAA
CAAGGCGCTCTACAGCATTTCGAACGACGAGCTCGCCTCCATCATCAGCCGCTGGCGCGGCAGCGACGGCGATCCGCGCA
CCTGGTACGCCTACCGCAACGAGATCTACCTGCTGATCGGGCTGGGCCTGTTGTCGGCCCTGCTGTTCCTGAGCTGGATC
GTCTACCTGCGGCGCCaGATCCGCCaGCGCAAGCGGGCCGAGCGGGCGCTGAACGACCAGCTGGAATTCATGCGCGTGCT
CATCGACGGCACGCCCAACCCCATCTATGTGCGCGATAAGGAAGGCCGCATGCTGTTGTGCAATGACGCCTACCTCGACA
CCTTTGGCGTGACTGCCGATGCGGTACTGGGCAAGACCATTCCGGAAGCCAACGTGGTGGGCGACCCGGCGCTGGCCCGC
GAAATGCACGAGTTCCTGCTCACCCGCGTGGCCGCCGAGCGCGAGCCGCGCTTCGAGGACCGCGATGTCACGCTGCACGG
CCGCACCCGCCATGTCTACCAGTGGACGATTCCGTACGGCGACTCGCTGGGCGAACTCAAGGGCATCATCGGCGGCTGGA
TCGACATCACCGAACGCGCCGAGCTGCTGCGCGAGCTGCACGACGCCAAGGAAAGCGCCGACGCCGCCAACCGGGCCAAG
ACCACGTTCCTGGCAACGATGAGCCACGAGATCCGCACGCCGATGAACGCGATCATCGGCATGCTGGAGCTGGCGCTGCT
CCGTCCGGCCGACCAGGAGCCGGATCGCCAGTCCATCCAGGTCGCGTACGACTCGGCCCGCAGCCTGCTGGAGCTGATAG
GCGACATCCTGGACATTGCGAAGATCGAGGCGGGAAAATTCGACCTGGCGCCGGTGCGCACGGCGCTGCGCGCCCTGCCC
GAAGGGGCGATCCGCGTCTTCGACGGATTGGCGCGCCAAAAAGGCATAGAGCTGGTATTGAAGACCGACATCGTGGGCGT
CGACGATGTATTGATAGACCCCTTGCGCATGAAGCAAGTGCTCTCGAACCTGGTGGGCAACGCCATCAAGTTCACCACCG
AAGGCCAGGTTGTCCTTGCCGTGACCGCACGCCCCGACGGCGACGCCGCGCACGTGCAGTTCAGCGTGAGCGACACCGGC
TGCGGCATCAGCGAGGCCGACCAACGGCAGCTGTTCAAACCGTTCTCGCAGGTGGGTGGCAGCGCCGAGGCCGGGCCGGC
GCCGGGCACCGGCCTGGGCCTGTCCATCAGCCGCCGCCTCGTCGAATTGATGGGGGGAACGCTGGTCATGCGCAGCGCGC
CAGGGGTGGGCACAACGGTTTCGGTGGACCTGAGGCTGACCATGGTCGAAAAATCCGTGCAGGCCACGCCGCCCGCTGCG
GCCGCTGCGGCCACGCCGTCCAAGCCGCAGGTATCGCTGCGCGTGCTGGTCGTCGATGACCACAAACCCAACCTGATGCT
GCTGCGCCAGCAGCTGGACTACCTGGGCCAGCGTGTCATCGCCGCCGACTCCGGCGAAGCCGCCCTGGCCCTGTGGCGCG
AGCATGCGTTCGACGTCGTGATCACCGATTGCAACATGCCCGGTATCAGCGGCTACGAATtGGCGCGCCGCATACGCGCC
GCCGAGGCCGCGCCCGGTTACGGACGTACGCGGTGCATTCTGTTCGGCTTCACGGCTTCGGCGCAGATGGACGAAGCGCA
GCGCTGCCGCGCCGCCGGCATGGACGACTGCCTGTTCAAGCCGATCGGCGTGGACGCCTTGCGGCAACGCTTGAACGAAG
CCGTGGCACGGGCCGCGCTCCCCACGCCCCCCTCGCCCCAGGCTGCCGCGCCGGCCACGGACGACGCCACCCCGACGGCG
TTCTCGGCCGAGTCGATTCTTGCCTTGACGCAGAACGATGAGGCGCTGATCCGGCAATTGCTCGAAGAAGTGATTCGCAC
CAACCGGGCGGACGTCGACCAATTGCAAAAGCTGCACCAGCAGGCCGATTGGCCGAAGGTCTCGGACATGGCGCACAGGC
TGGCCGGCGGCGCGCGCGTGGTCGATGCCAAGGCCATGATAGACACTGCGCTGGCGCTGGAGAAAAAAGCGCAAGGCCAG
GCTGGCCCCTCGCCCGAAATCGACGGCCTGGTACGTACGCTTGCGGCGCAGTCCGCCGCGCTGGAGACGCAACTGCGCGC
CTGGCTGGAGCAACGGCCGCATCAAGATCAGCCCTGA
>Bb_B2491.seq
ATGCCCGCCCCGCACCGCCTGTACCCCCGCAGTCTGATCTGCCTGGCTCAGGCGCTATTGGCATGGGCTTTGCTGGCATG
GGCGCCCGCGCAGGCAAGCCAGGAGCTGACCCTGGTCGGCAAGGCTGCCGTTCCCGACGTCGAGGTCGCGCTCGACGGCG
ACGACTGGCGTTGGCTGGCCCGCAAGCGGGTACTGACGCTGGGTGTGTACGCACCGGACATTCCTCCGTTCGACGTCACC
TATGGCGAACGCTACGAAGGCCTGACGGCCGACTACATGGCGATCATCGCGCACAACCTGGGGATGCAGGCGAAAGTGCT
GCGCTACCCCACGCGCGAACAAGCCCTCAGCGCGCTGGAAAGCGGGCAGATCGACCTCATCGGCACCGTCAATGGCACCG
ACGGCCGGCAACAGAGCCTGCGTCTGAGCGTTCCCTACGCCGCCGACCACCCGGTGATCGTCATGCCCATCGGCGCACGC
CACGTTCCAGCCTCGAACCTGGCCGGCCAGCGGCTGGCGGTCGACATCAACTACCTGCCCAAGGAAACGCTCGCACGGGC
CTACCCGCAGGCGACGCTGCATTACTTTCCCTCATCCGAGCAGGCGCTGGCCGCGGTGGCCTATGGGCAGGCCGACGTAT
TCATCGGCGATGCCCTGACCACCTCGCACCTCGTATCGCAAAGCTATTTCAATGACGTTCGCGCGGTCGCCCCGGCCCAT
ATCGCGACGGGCGGAGAATCCTTCGGCGTGCGCGCCGACAACACCCGCCTGCTGCGGGTGGTCAACGCCGTACTCGAAGC
CATTCCGCCTTCCGAACACCGCAGCCTGATCTACCGCTGGGGACTGGGCAGCAGCATTTCGCTCGATTTCGCGCACCCCG
CGTATTCCGCGCGCGAGCAGCAATGGATGGCAGACCACCCCGTCGTCAAGGTGGCGGTCCTGAATCTGTTCGCGCCCTTC
ACCCTGTTCCGCACCGACGAACAGTTCGGCGGGATCAGCGCCGCCGTGCTGCAGCTGCTGCAATTGCGCACCGGCCTGGA
CTTCGAGATCATCGGCGTCGACACGGTCGAGGAACTGATAGCCAAGCTGCGTTCGGGCGAAGCCGACATGGCCGGCGCCC
TGTTCGTCAACAGCGCGCGGGAGTCCTTCCTCAGTTTCAGCCGGCCGTATGTGCGCAATGGCATGGTGATCGTCACGCGC
CAGGACCCCGACGCGCCCGTCGACGCCGATCATCTGGACGGCCGCACGGTCGCGTTGGTGCGCAACAGCGCCGCCATTCC
TCTGCTGCAGCGGCGCTACCCCCAGGCGAAGGTGGTGACCGCCGACAACCCGAGCGAGGCGATGCTGATGGTGGCCAATG
GACAGGCCGACGCCGTCGTGCAGACGCAGATCAGCGCCAGCTATTACGTCAACCGCTACTTCGCCGGCAAGCTGCGCATC
GCCTCGGCGCTGGACCTGCCCCCGGCCGAGATCGCGCTGGCGACGACGCGCGGCCAGACCGAACTGATGTCCATCCTGAA
CAAGGCGCTCTACAGCATTTCGAACGACGAGCTCGCCTCCATCATCAGCCGCTGGCGCGGCAGCGACGGCGATCCGCGCA
CCTGGTACGCCTACCGCAACGAGATCTACCTGCTGATCGGGCTGGGCCTGTTGTCGGCCCTGCTGTTCCTGAGCTGGATC
GTCTACCTGCGGCGCCaGATCCGCCAGCGCAAGCGGGCCGAGCGGGCGCTGAACGACCAGCTGGAATTCATGCGCGTGCT
CATCGACGGCACGCCCAACCCCATCTATGTGCGCGATAAGGAAGGCCGCATGCTGTTGTGCAATGACGCCTACCTCGACA
CCTTTGGCGTGACTGCCGATGCGGTACTGGGCAAGACCATTCCGGAAGCCAACGTGGTGGGCGACCCGGCGCTGGCCCGC
GAAATGCACGAGTTCCTGCTCACGCGCGTGGCCGCCGAGCGCGAGCCGCGCTTCGAGGACCGCGATGTCACGCTGCACGG
CCGCACCCGCCATGTCTACCAGTGGACGATTCCGTACGGCGACTCGCTGGGCGAACTCAAGGGCATCATCGGCGGCTGGA
TCGACATCACCGAACGCGCCGAGCTGCTGCGCGAGCTGCACGACGCCAAGGAAAGCGCCGACGCCGCCAACCGGGCCAAG
ACCACGTTCCTGGCAACGATGAGCCACGAGATCCGCACGCCGATGAACGCGATCATCGGCATGCTGGAGCTGGCGCTGCT
CCGTCCGGCCGACCAGGAGCCGGATCGCCAGTCCATCCAGGTCGCGTACGACTCGGCCCGCAGCCTGCTGGAGCTGATAG
GCGACATCCTGGACATTGCGAAGATCGAGGCAGGAAAATTCGACCTGGCGCCGGTGCGCACGGCGCTGCGCGCCCTGCCC
GAAGGGGCGATCCGCGTCTTCGACGGATTGGCGCGCCAAAAAGGCATAGAGCTGGTATTGAAGACCGACATCGTGGGCGT
CGACGATGTATTGATAGACCCCTTGCGCATGAAGCAAGTGCTCTCGAACCTGGTGGGTAACGCCATCAAGTTCACCACCG
AAGGCCAGGTTGTCCTTGCCGTGACCGCACGCCCCGACGGCGAGGCCGCGCACGTGCAGTTCAGCGTGAGCGACACCTGC
TGCGGCATCAGCGAGGCCGACCAACGGCAGCTGTTCAAACCGTTCTCGCAGGTGGGTGGCAGCGCCGAGGCCGGGCCGGC
GCCGGGCACCGGCCTGGGCCTGTCCATCAGCCGTCGCCTCGTCGAATTGATGGGGGGAACGCTGGTCATGCGCAGCGCGC
CAGGGGTGGGCACAACGGTTTCGGTGGACCTGAGGCTGACCATGGTCGAAAAATCCGTGCAGGCCACGCCGCCCGCTGCG
GCCGCTGCGGCCACGCCGTCCAAGCCGCAGGTATCGCTGCGCGTGCTGGTCGTCGATGACCACAAACCCAACCTGATGCT
GCTGCGCCAGCAGCTGGACTACCTGGGCCAGCGTGTCATCGCCGCCGACTCCGGCGAAGCCGCCCTGGCCCTGTGGCGCG
AGCATGCGTTCGACGTCGTGATCACCGATTGCAACATGCCCGGTATCAGCGGCTACGAATtGGCGCGCCGCATACGCGCC
GCCGAGGCCGCGCCCGGTTACGGACGTACGCGGTGCATTCTGTTCGGCTTCACGGCTTCGGCGCAGATGGACGAAGCGCA
GCGCTGCCGCGCCGCCGGCATGGACGACTGCCTGTTCAAGCCGATCGGCGTGGACGCCTTGCGGCAACGCCTGAACGAAG
CCGTGGCACGGGCCGCGCTCCCCACGCCCCCCTCGCCCCAGGCTGCCGCGCCGGCCACGGACGACGCCACCCCGACGGCG
TTCTCGGCCGAATCGATTCTTGCCTTGACGCAGAACGATGAGGCGCTGATCCGGCAATTGCTCGAAGAAGTGATTCGCAC
CAACCGGGCGGACGTCGACCAATTGCAAAAGCTGCACCAGCAGGCCGATTGGCCGAAGGTCTCGGACATGGCGCACAGGC
TGGCCGGCGGCGCGCGCGTGGTCGATGCCAAGGCCATGATAGACACTGCGCTGGCGCTGGAGAAAAAAGCGCAAGGCCAG
GCTGGCCCCTCACCCGAAATCGACGGCCTGGTACGTACGCTTGCGGCGCAGTCCGCCGCGCTGGAGACGCAACTGCGCGC
CTGGCTGGAGCAACGGCCGCATCAAGATCAGCCCTGA
>Bb_B2492.seq
ATGCCCGCCCCGCACCGCCTGTACCCCCGCAGTCTGATCTGCCTGGCTCAGGCGCTATTGGCATGGGCTTTGCTGGCATG
GGCGCCCGCGCAGGCAAGCCAGGAGCTGGCCCTGGTCGGCAAGGCTGCCGTTCCCGACGTCGAGGTCGCACTCGACGGCG
ACGACTGGCGTTGGCTGGCCCGCAAGCGGGTACTGACGCTGGGTGTGTACGCACCGGACATTCCTCCGTTCGACGTCACC
TATGGCGAACGCTACGAAGGCCTGACGGCCGACTACATGGCGATCATCGCGCACAATCTGGGGATGCAGGCGAAAGTGCT
GCGATACCCCACGCGCGAACAAGCCCTCGGCGCGCTGGAAAGCGGGCAGATCGACCTCATCGGCACCGTCAATGGCACCG
ACGGCCGGCAACAGAGCCTGCGTCTGAGCGTTCCCTACGCCGCCGACCACCCGGTGATCGTCATGCCCATCGGCGCACGC
CACGTTCCAGCCTCGAACCTGGCCGGCCAGCGGCTGGCGGTCGACATCAACTACCTGCCCAAGGAAACGCTCGCACGGGC
CTACCCGCAGGCGACGCTGCATTACTTTCCCTCATCCGAACAGGCGCTGGCCGCGGTGGCCTATGGGCAGGCCGACGTAT
TCATCGGCGATGCCCTGACCACCTCGCACCTCGTATCGCAAAGCTATTTCAATGACGTTCGCGTAGTCGCCCCGGCCCAT
ATCGCGACGGGCGGAGAATCCTTCGGCGTGCGCGCCGACAACACCCGCCTGCTGCGGGTGGTCAACGCCGTACTCGAAGC
CATTCCGCCTTCCGAACACCGCAGCCTGATCTACCGCTGGGGACTGGGCAGCAGCATTTCGCTCGATTTCGCGCACCCCG
CGTATTCCGCGCGCGAGCAGCAATGGATGGCAGACCACCCCGTCGTCAAGGTGGCGGTCCTGAATCTGTTCGCGCCCTTC
ACCCTGTTCCGCACCGACGAACAGTTCGGCGGGATCAGCGCCGCCGTGCTGCAGCTGCTGCAATTGCGCACCGGCCTGGA
CTTCGAGATCATCGGCGTCGACACGGTCGAGGAACTGATAGCCAAGCTGCGTTCGGGCGAAGCCGACATGGCCGGCGCCC
TGTTCGTCAACAGCGCGCGGGAGTCCTTCCTCAGTTTCAGCCGGCCGTATGTGCGCAATGGCATGGTGATCGTCACGCGC
CAGGACCCCGACGCGCCCGTCGACGCCGATCATCTGGACGGCCGCACGGTCGCGTTGGTGCGCAACAGCGCCGCCATTCC
CCTGCTGCAGCGGCGCTATCCCCAGGCGAAGGTGGTGACCGCCGACAACCCGAGCGAGGCGATGCTGATGGTGGCCAATG
GACAGGCCGACGCCGTCGTGCAGACGCAGATCAGCGCCAGCTATTACGTCAACCGCTACTTCGCCGGCAAGCTGCGCATC
GCCTCGGCGCTGGACCTGCCCCCGGCCGAGATCGCGCTGGCGACGACGCGCGGCCAGACCGAACTGATGTCCATCCTGAA
CAAGGCGCTCTACAGCATTTCGAACGACGAGCTCGCCTCCATCATCAGCCGCTGGCGCGGCAGCGACGGCGATCCGCGCA
CCTGGTACGCCTACCGCAACGAGATCTACCTGCTGATCGGGCTGGGCCTGTTGTCGGcCCTGCTGTTCCTGAGCTGGATC
GTCTACCTGCGGCGCCAGATCCGCCAGCGCAAGCGGGCCGAGCGGGCGCTGAACGACCAGCTGGAATTCATGCGCGTGCT
CATCGACGGCACGCCCAACCCCATCTATGTGCGCGATAAGGAAGGCCGCATGCTGTTGTGCAATGACGCCTACCTCGACA
CCTTTGGCGTGACTGCCGATGCGGTACTGGGCAAGACCATTCCGGAAGCCAACGTGGTGGGCGACCCGGCGCTGGCCCGC
GAAATGCACGAGTTCCTGCTCACGCGCGTGGCCGCCGAGCGCGAGCCGCGCTTCGAGGACCGCGATGTCACGCTGCACGG
CCGCACCCGCCATGTCTACCAGTGGACGATTCCGTACGGCGACTCGCTGGGCGAACTCAAGGGCATCATCGGCGGCTGGA
TCGACATCACCGAACGCGCCGAGCTGCTGCGCGAGCTGCACGACGCCAAGGAAAGCGCCGACGCCGCCAACCGGGCCAAG
ACCACGTTCCTGGCAACGATGAGCCACGAGATCCGCACGCCGATGaACGCGATCATCGGCATGCTGGAGCTGGCGCTGCT
CCGTCCGGCCGACCAGGAGCCGGATCGCCAGTCCATCCAGGTCGCGTACGACTCGGCCCGCAGCCTGCTGGAGCTGATAG
GCGACATCCTGGACATTGCGAAGATCGAGGCGGGAAAATTCGACCTGGCGCCGGTGCGCACGGCGCTGCGCGCCCTGCCC
GAAGGGGCGATCCGCGTCTTCGACGGATTGGCGCGCCAAAAAGGCATAGAGCTGGTATTGAAGACCGACATCGTGGGCGT
CGACGATGTCTTGATAGACCCCTTGCGCATGAAGCAAGTGCTCTCGAACCTGGTGGGCAACGCCATCAAGTTCACCACCG
AAGGCCAGGTTGTCCTTGCCGTGACCGCACGCCCCGACGGCGAGGCCGCGCACGTGCAGTTCAGCGTGAGCGACACCGGC
TGCGGCATCAGCGAGGCCGACCAACGGCAGCTGTTCAAACCGTTCTCGCAGGTGGGTGGCAGCGCCGAGGCCGGGCCGGC
GCCGGGCACCGGCCTGGGCCTGTCCATCAGCCGCCGCCTCGTCGAATTGATGGGGGGAACGCTGGTCATGCGCAGCGCGC
CAGGGGTGGGCACAACGGTTTCGGTGGACCTGAGGCTGACCATGGTCGAAAAATCCGTGCAGGCCACGCCGCCCGCTGCG
GCCGCTGCGGCCACGCCGTCCAAGCCGCAGGTATCGCTGCGCGTGCTGGTCGTCGATGACCACAAACCCAACCTGATGCT
GCTGCGCCAGCAGCTGGACTACCTGGGCCAGCGTGTCATCGCCGCCGACTCCGGCGAAGCCGCCCTGGcCCTGTGGCGCG
AGCATGCGTTCGACGTCGTGATCACCGATtGCAACATGCCCGGTATCAGCGGCTACGAATtGGCGCGCCGCATACGCGCC
GCCGAGGCCGCGCCCGGTTACGGACGTACGCGGTGTATTCTGTTCGGCTTCACGGCTTCGGCGCAGATGGACGAAGCGCA
GCGCTGCCGCGCCGCCGGCATGGACGACTGCCTGTTCAAGCCGATCGGCGTGGACGCCTTGCGGCAACGCTTGAACGAAG
CCGTGGCACGGGCCGCGCTCCCCACGCCCCCCTCGCCCCAGGCTGCCGCGCCGGCCACGGACGACGCCACCCCGACGGCG
TTCTCGGCCGAGTCGATTCTTGCCTTGACGCAGAACGATGAGGCGCTGATCCGGCAATTGCTCGAAGAAGTGATTCGCAC
CAACCGGGCGGACGTCGACCAATTGCAAAAGCTGCACCAGCAGGCCGATTGGCCGAAGGTCTCGGACATGGCGCACAGGC
TGGCCGGCGGCGCGCGCGTGGTCGATGCCAAGGCCATGATAGACACTGCGCTGGCGCTGGAGAAAAAAGCGCAAGGCCAG
GCTGGCCCCTCACCCGAAATCGACGGCCTGGTACGTACGCTTGCGGCGCAGTCCGCCGCGCTGGAGACGCAACTGCGCGC
CTGGCTGGAGCAACGGCCGCATCAAGATCAGCCCTGA
>Bb_B2493.seq
ATGCCCGCCCCGCACCGCCTGTACCCCCGCAGTCTGATCTGCCTGGCTCAGGCGCTATTGGCATGGGCTTTGCTGGCATG
GGCGCCCGCGCAGGCAAGCCAGGAGCTGACCCTGGTCGGCAAGGCTGCCGTTCCCGACGTCGAGATCACGCTCGACGGCG
ACGACTGGCGCTGGCTGGCGCGCAAGCGGGTGCTGACGCTGGGCGTGTACGCGCCGGACATTCCCCCGTTCGACGTCACC
TATGACGAGCGCTACGAAGGCCTGACGGCCGACTACATGGCGATCATCGCGCACAACCTGGGCGTCCAGGCAAAAGTGCT
GCGCTACCCCACGCGCGAGCAAGCCGTCGGCGCACTGGAAAGCGGACAGATCGACCTCATCGGCACCGTCAATGGCATCG
AGGGCCGGCTGCAGAGCCTGCGCCTGAGTGTTCCCTACGCGGCCGACCACCCGGTGCTGGTCATGCCCATCGGCGCGCGC
CGCGCTCCGCCCGCGGACCTGGCAGGCCAGCGGTTGGCGGTTGACGCCAACTACTTGCCCAGGGAAACGCTGCAGCAGGC
CTATCCCCAGGCAACGCTGCATTACTTCCCATCATCCGAACAGGCGCTGGCCGCGGTGGCCTATGGACAAGCCGACGTGT
TCATCGGCGATGCGCTGACCACCTCGCACCTCGTTTCGCAAAGCTACTTCAACGACGTTCGCGTCGTCGCCCCGGCCCAG
ATCGTGACGGGCGGGGAATCCTTCGGCGTGCGCGCCGACAATACCCGCCTGCTGCGGGTGGTCAATGCCGTGCTCGAAGC
CATTCCGGCCTCCGAGCGCCGCAGCCTGATCTACCGCTGGGGCCTGGGCAGCAGCATTTCGCTCGATTTCGCGCGCCCCG
CCTATTCAGCGCGCGAGCAGCAGTGGATGGCAAACCATCCGGTCGTCAAGGTGGCGGTCCTGAACCTGTTCGCGCCCTTC
ACCCTGTTTCGCACCGATGAACAGTTCGGCGGCATCAGTGCCGCCGTGCTGCAACTGCTGCAGTTGCGCACCGGCCTGGA
TTTCCAGATCATCGGCGTCGACACGGTCGAGGAGCTGATCGCCAAGCTGCGCTCGGGCGAAGCCGACATGGCCGGCGCCC
TGTTCGTCAATGCCGCGCGGGAATCCGTCCTCAGCTTCAGCCGGCCGTATGTGCGCAATGGTCTGGTGATCGTCACGCGC
CAGGACCCCGCCGCGCCCGCCGACGCCGATCACCTCGACGGCCGCACGATTGCGATGGTGCGCAACAGCGCCGCCATCCC
GCTCCTGCAGCAGCGCTATCCCCAGGCGAAGGTCGTGACCGCCGACAACCCGACCGAAGCCATGCTGCTGGTGGCCGATG
GCCAGGCCGACGCCGTCGTGCAGACGCAGATCAGCGCCAGCTACTACGTCAACCGCTACTTCGCCGGAAAACTGCGCATT
GCCTCGGCGCTGGACCTGCCGCCGGCCGAGATCGCGCTGGCGACGGCGCGCGGCCAGACCGAGCTGATATCCATCCTGAA
CAAGGCGCTCTACAGCATTTCGAACGACGAACTCGCCTCCATCGTCAGCCGCTGGCGCGGCAGCGACGGCGATCCGCGCA
CCTGGTACGCCTACCGCAACGAAATCTACCTGCTGATCGGGCTGGGCCTGTTGTCGGCCCTGCTGTTCCTGAGCTGGATC
GTCTACCTGCGGCGCCAGATCCGCCAGCGCAAGCGGGCCGAGCGGGCGCTGAACGACCAGCTGGAATTCATGCGCGTGCT
CATCGACGGCACGCCCAACCCCATCTATGTGCGCGATAAGGAAGGCCGCATGCTGTTGTGCAATGACGCCTACCTCGACA
CCTTTGGCGTGACTGCCGATGCGGTACTGGGCAAGACCATCCCGGAGGCCAACGTGGTGGGCGACCCGGCGCTGGCTCGC
GAGATGCACGAGTTCCTGCTCACGCGCATGGCCGCCGAGCGCGAGCCGCGCTTCGAGGACCGCGATGTCACGCTGCACGG
CCGCACCCGCCATGTCTACCAGTGGACGGTTCCGTACGGCGACTCGCTGGGCGAACTCAAGGGCATCATCGGCGGCTGGA
TCGACATTACCGAACGCGCCGAGCTGCTGCGCGAGCTGCACGACGCCAAGGAAAGCGCCGACGCCGCCAACCGGGCCAAG
ACCACGTTCCTGGCAACGATGAGCCACGAGATCCGCACGCCGATGAACGCGATCATCGGCATGCTGGAGCTGGCGCTGCT
CCGTCCGGCCGACCAGGAGCCGGACCGCCAGTCCATCCAGGTCGCGTACGACTCGGCCCGCAGCCTGCTGGAGCTGATAG
GCGACATCCTGGACATTGCGAAGATCGAGGCGGGAAAATTCGACCTGGCGCCGGTGCGCACGGCGCTGCGCGCCCTGCCC
GAAGGGGCGATCCGCGTCTTCGACGGGTTGGCGCGCCAGAAAGGCATAGAGCTGGTATTGAAGACCGACATCGTGGGCGT
GGACGATGTATTGATAGACCCCTTGCGCATGAAGCAAGTGCTCTCGAACCTGGTGGGCAACGCCATCAAGTTCACCACCG
AAGGCCAGGTTGTCCTTACCGTGACCGCGCGCCCCGACGGCGAGGCCGCGCACGTGCAGTTCAGCGTGAGCGACACCGGC
TGCGGCATCAGCGAGGCCGACCAACGGCAGCTGTTCAAACCGTTCTCGCAGGTGGGCGGCAGCGCCGAGGCCGGGCCGGC
GCCGGGCACTGGCCTGGGCCTGTCCATCAGCCGCCGCCTCGTCGAATTGATGGGGGGAACGCTGGTCATGCGCAGCGCGC
CAGGGGTGGGCACAACGGTTTCGGTGGACCTGAGGCTGACCATGGTCGAAAAATCCGCGCAGGCCACGCCGCCCGCTGCG
GCCGCTCAGGCCACGCCATCCAAGCCGCAGGTATCGCTGCGCGTGCTGGTCGTCGATGACCACAAGCCCAACCTGATGCT
GCTGCGCCAGCAGCTGGACTACCTGGGCCAGCGTGTCGTCGCCGCCGACTCCGGCGAAGCCGCCCTGGCCCTGTGGCACG
AGCATGCGTTCGACGTCGTGATCACCGATTGCAACATGCCCGGTATCAACGGCTACGAATTGGCGCGCCGCATACGCGCC
GCCGAGGCCGCGCCCGGTTACGGACGTACGCGGTGCATTCTGTTCGGCTTCACGGCTTCGGCGCAGATGGACGAAGCGCA
GCGCTGCCGCGCCGCCGGCATGGACGACTGCCTGTTCAAGCCGATCGGCGTGGACGCCTTGCGGCAACGCTTGAACGAAG
CCGCGGCACGGGCCGCGCTCCCCACGCCCCCCTCGCCCCAGGCTGCCGCGCCGGCCACGCACGACGCCACCCCGGCGGCG
TTCTCGGCCGAGTCGATTCTTGCCCTGACGCAGAACGATGAGGCGCTGATCCGGCAATTGCTCGAAGAAGTGATTCGCAC
CAACCGGGCGGACGTCGATCAATTGCAGAAGCTGCACCAGCAGGCCGATTGGCCGAAGGTCTCGGACATGGCGCACAGGC
TGGCCGGCGGCGCGCGCGTGGTCGATGCCAAGGCCATGATAGACACTGCGCTGGCGCTGGAGAAAAAAGCGCAAGGCCAG
GCTGGCCCCTCGCCCGAAATCGACGGCATGGTACGTACGCTTGCGGCGCAGTCCGCCGCGCTGGAGACGCAACTACGCGC
CTGGCTGGAGCAACGGCCGCATCAAGGCCAGCCCTGA
>Bb_B2494.seq
ATGCCCGCCCCGCACCGCCTGTACCCCCGCAGTCTGATCTGCCTGGCTCAGGCGCTATTGGCATGGGCTTTGCTGGCATG
GGCGCCCGCGCAGGCAAGCCAGGAGCTGACCCTGGTCGGCAAGGCTGCCGTTCCCGACGTCGAGGTCGCGCTCGACGGCG
ACGACTGGCGTTGGCTGGCCCGCAAGCGGGTACTGACGCTGGGTGTGTACGCACCGGACATTCCTCCGTTCGACGTCACC
TATGGCGAACGCTACGAAGGCCTGACGGCCGACTACATGGCGATCATCGCGCACAACCTGGGGATGCAGGCGAAAGTGCT
GCGCTACCCCACGCGCGAACAAGCCCTCAGCGCGCTGGAAAGCGGGCAGATCGACCTCATCGGCACCGTCAATGGCACCG
ACGGCCGGCAACAGAGCCTGCGTCTGAGCGTTCCCTACGCCGCCGACCACCCGGTGATCGTCATGCCCATCGGCGCACGC
CACGTTCCAGCCTCGAACCTGGCCGGCCAGCGGCTGGCGGTCGACATCAACTACCTGCCCAAGGAAACGCTCGCACGGGC
CTACCCGCAGGCGACGCTGCATTACTTTCCCTCATCCGAGCAGGCGCTGGCCGCGGTGGCCTATGGGCAGGCCGACGTAT
TCATCGGCGATGCCCTGACCACCTCGCACCTCGTATCGCAAAGCTATTTCAATGACGTTCGCGCGGTCGCCCCGGCCCAT
ATCGCGACGGGCGGAGAATCCTTCGGCGTGCGCGCCGACAACACCCGCCTGCTGCGGGTGGTCAACGCCGTACTCGAAGC
CATTCCGCCTTCCGAACACCGCAGCCTGATCTACCGCTGGGGACTGGGCAGCAGCATTTCGCTCGATTTCGCGCACCCCG
CGTATTCCGCGCGCGAGCAGCAATGGATGGCAGACCACCCCGTCGTCAAGGTGGCGGTCCTGAATCTGTTCGCGCCCTTC
ACCCTGTTCCGCACCGACGAACAGTTCGGCGGGATCAGCGCCGCCGTGCTGCAGCTGCTGCAATTGCGCACCGGCCTGGA
CTTCGAGATCATCGGCGTCGACACGGTCGAGGAACTGATAGCCAAGCTGCGTTCGGGCGAAGCCGACATGGCCGGCGCCC
TGTTCGTCAACAGCGCGCGGGAGTCCTTCCTCAGTTTCAGCCGGCCGTATGTGCGCAATGGCATGGTGATCGTCACGCGC
CAGGACCCCGACGCGCCCGTCGACGCCGATCATCTGGACGGCCGCACGGTCGCGTTGGTGCGCAACAGCGCCGCCATTCC
CCTGCTGCAGCGGCGCTACCCCCAGGCGAAGGTGGTGACCGCCGACAACCCGAGCGAGGCGATGCTGATGGTGGCCAATG
GACAGGCCGACGCCGTCGTGCAGACGCAGATCAGCGCCAGCTATTACGTCAACCGCTACTTCGCCGGCAAGCTGCGCATC
GCCTCGGCGCTGGACCTGCCCCCGGCCGAGATCGCGCTGGCGACGACGCGCGGCCAGACCGAACTGATGTCCATCCTGAA
CAAGGCGCTCTACAGCATTTCGAACGACGAGCTCGCCTCCATCATCAGCCGCTGGCGCGGCAGCGACGGCGATCCGCGCA
CCTGGTACGCCTACCGCAACGAGATCTACCTGCTGATCGGGCTGGGCCTGTTGTCGGCCCTGCTGTTCCTGAGCTGGATC
GTCTACCTGCGGCGCCaGATCCGCCAGCGCAAGCGGGCCGAGCGGGCGCTGAACGACCAGCTGGAATTCATGCGCGTGCT
CATCGACGGCACGCCCAACCCCATCTATGTGCGCGATAAGGAAGGCCGCATGCTGTTGTGCAATGACGCCTACCTCGACA
CCTTTGGCGTGACTGCCGATGCGGTACTGGGCAAGACCATTCCGGAAGCCAACGTGGTGGGCGACCCGGCGCTGGCCCGC
GAAATGCACGAGTTCCTGCTCACGCGCGTGGCCGCCGAGCGCGAGCCGCGCTTCGAGGACCGCGATGTCACGCTGCACGG
CCGCACCCGCCATGTCTACCAGTGGACGATTCCGTACGGCGACTCGCTGGGCGAACTCAAGGGCATCATCGGCGGCTGGA
TCGACATCACCGAACGCGCCGAGCTGCTGCGCGAGCTGCACGACGCCAAGGAAAGCGCCGACGCCGCCAACCGGGCCAAG
ACCACGTTCCTGGCAACGATGAGCCACGAGATCCGCACGCCGATGaACGCGATCATCGGCATGCTGGAGCTGGCGCTGCT
CCGTCCGGCCGACCAGGAGCCGGATCGCCAGTCCATCCAGGTCGCGTACGACTCGGCCCGCAGCCTGCTGGAGCTGATAG
GCGACATCCTGGACATTGCGAAGATCGAGGCAGGAAAATTCGACCTGGCGCCGGTGCGCACGGCGCTGCGCGCCCTGCCC
GAAGGGGCGATCCGCGTCTTCGACGGATTGGCGCGCCAAAAAGGCATAGAGCTGGTATTGAAGACCGACATCGTGGGCGT
CGACGATGTATTGATAGACCCCTTGCGCATGAAGCAAGTGCTCTCGAACCTGGTGGGTAACGCCATCAAGTTCACCACCG
AAGGCCAGGTTGTCCTTGCCGTGACCGCACGCCCCGACGGCGAGGCCGCGCACGTGCAGTTCAGCGTGAGCGACACCGGC
TGCGGCATCAGCGAGGCCGACCAACGGCAGCTGTTCAAACCGTTCTCGCAGGTGGGTGGCAGCGCCGAGGCCGGGCCGGC
GCCGGGCACCGGCCTGGGCCTGTCCATCAGCCGCCGCCTCGTCGAATTGATGGGGGGAACGCTGGTCATGCGCAGCGCGC
CAGGGGTGGGCACAACGGTTTCGGTGGACCTGAGGCTGACCATGGTCGAAAAATCCGTGCAGGCCACGCCGCCCGCTGCG
GCCGCTGCGGCCACGCCGTCCAAGCCGCAGGTATCGCTGTGCGTGCTGGTCGTCGATGACCACAAACCCAACCTGATGCT
GCTGCGCCAGCAGCTGGACTACCTGGGCCAGCGTGTCATCGCCGCCGACTCCGGCGAAGCCGCCCTGGCCCTGTGGCGCG
AGCATGCGTTCGACGTCGTGATCACCGATTGCAACATGCCCGGTATCAGCGGCTACGAATTGGCGCGCCGCATACGCGCC
GCCGAGGCCGCGCCCGGTTACGGACGTACGCGGTGCATTCTGTTCGGCTTCACGGCTTCGGCGCAGATGGACGAAGCGCA
GCGCTGCCGCGCCGCCGGCATGGACGACTGCCTGTTCAAGCCGATCGGCGTGGACGCCTTGCGGCAACGCCTGAACGAAG
CCGTGGCACGGGCCGCGCTCCCCACGCCCCCCTCGCCCCAGGCTGCCGCGCCGGCCACGGACGACGCCACCCCGGCGGCG
TTCTCGGCCGAGTCGATTCTTGCCTTGACGCAGAACGATGAGGCGCTGATCCGGCAATTGCTCGAAGAAGTGATTCGCAC
CAACCGGGCGGACGTCGACCAATTGCAAAAGCTGCACCAGCAGGCCGATTGGCCGAAGGTCTCGGACATGGCGCACAGGC
TGGCCGGCGGCGCGCGCGTGGTCGATGCCAAGGCCATGATAGACACTGCGCTGGCGCTGGAGAAAAAAGCGCAAGGCCAG
GCTGGCCCCTCGCCCGAAATCGACGGCCTGGTACGTACGCTTGCGGCGCAGTCCGCCGCGCTGGAGACGCAACTGCGCGC
CTGGCTGGAGCAACGGCCGCATCAAGATCAGCCCTGA
>Bb_B2495.seq
ATGCCCGCCCCGCACCGCCTGTACCCCCGCAGTCTGATCTGCCTGGCTCAGGCGCTATTGGCATGGGCTTTGCTGGCATG
GGCGCCCGCGCAGGCAAGCCAGGAGCTGACCCTGGTCGGCAAGGCTGCCGTTCCCGACGTCGAGGTCGCGCTCGACGGCG
ACGACTGGCGTTGGCTGGCCCGCAAGCGGGTACTGACGCTGGGTGTGTACGCACCGGACATTCCTCCGTTCGACGTCACC
TATGGCGAACGCTACGAAGGCCTGACGGCCGACTACATGGCGATCATCGCGCACAACCTGGGGATGCAGGCGAAAGTGCT
GCGCTACCCCACGCGCGAACAAGCCCTCAGCGCGCTGGAAAGCGGGCAGATCGACCTCATCGGCACCGTCAATGGCACCG
ACGGCCGGCAACAGAGCCTGCGTCTGAGCGTTCCCTACGCCGCCGACCACCCGGTGATCGTCATGCCCATCGGCGCACGC
CACGTTCCAGCCTCGAACCTGGCCGGCCAGCGGCTGGCGGTCGACATCAACTACCTGCCCAAGGAAACGCTCGCACGGGC
CTACCCGCAGGCGACGCTGCATTACTTTCCCTCATCCGAGCAGGCGCTGGCCGCGGTGGCCTATGGGCAGGCCGACGTAT
TCATCGGCGATGCCCTGACCACCTCGCACCTCGTATCGCAAAGCTATTTCAATGACGTTCGCGTGGTCGCCCCGGCCCAT
ATCGCGACGGGCGGAGAATCCTTCGGCGTGCGCGCCGACAACACCCGCCTGCTGCGGGTGGTCAACGCCGTACTCGAAGC
CATTCCGCCTTCCGAACACCGCAGCCTGATCTACCGCTGGGGACTGGGCAGCAGCATTTCGCTCGATTTCGCGCACCCCG
CGTATTCCGCGCGCGAGCAGCAATGGATGGCAGACCACCCCGTCGTCAAGGTGGCGGTCCTGAATCTGTTCGCGCCCTTC
ACCCTGTTCCGCACCGACGAACAGTTCGGCGGGATCAGCGCCGCCGTGCTGCAGCTGCTGCAATTGCGCACCGGCCTGGA
CTTCGAGATCATCGGCGTCGACACGGTCGAGGAACTGATAGCCAAGCTGCGTTCGGGCGAAGCCGACATGGCCGGCGCCC
TGTTCGTCAACAGCGCGCGGGAGTCCTTCCTCAGTTTCAGCCGGCCGTATGTGCGCAATGGCATGGTGATCGTCACGCGC
CAGGACCCCGACGCGCCCGTCGACGCCGATCATCTGGACGGCCGCACGGTCGCGTTGGTGCGCAACAGCGCCGCCATTCC
TCTGCTGCAGCGGCGCTATCCCCAGGCGAAGGTGGTGACCGCCGACAACCCGAGCGAGGCGATGCTGATGGTGGCCAATG
GACAGGCCGACGCCGTCGTGCAGACGCAGATCAGCGCCAGCTATTACGTCAACCGCTACTTCGCCGGCAAGCTGCGCATC
GCCTCGGCGCTGGACCTGCCTCCGGCCGAGATCGCGCTGGCGACGACGCGCGGCCAGACCGAACTGATGTCCATCCTGAA
CAAGGCGCTCTACAGCATTTCGAACGACGAGCTCGCCTCCATCATCAGCCGCTGGCGCGGCAGCGACGGCGATCCGCGCA
CCTGGTACGCCTACCGCAACGAGATCTACCTGCTGATCGGGCTGGGCCTGTTGTCGGCCCTGCTGTTCCTGAGCTGGATC
GTCTACCTGCGGCGCCaGATCCGCCAGCGCAAGCGGGCCGAGCGGGCGCTGAACGACCAGCTGGAATTCATGCGCGTGCT
CATCGACGGCACGCCCAACCCCATCTATGTGCGCGATAAGGAAGGCCGCATGCTGTTGTGCAATGACGCCTACCTCGACA
CCTTTGGCGTGACTGCCGATGCGGTACTGGGCAAGACCATTCCGGAAGCCAACGTGGTGGGCGACCCGGCGCTGGCCCGC
GAAATGCACGAGTTCCTGCTCACGCGCGTGGCCGCCGAGCGCGAGCCGCGCTTCGAGGACCGCGATGTCACGCTGCACGG
CCGCACCCGCCATGTCTACCAGTGGACGATTCCGTACGGCGACTCGCTGGGCGAACTCAAGGGCATCATCGGCGGCTGGA
TCGACATCACCGAACGCGCCGAGCTGCTGCGCGAGCTGCACGACGCCAAGGAAAGCGCCGACGCCGCCAACCGGGCCAAG
ACCACGTTCCTGGCAACGATGAGCCACGAGATCCGCACGCCGATGaACGCGATCATCGGCATGCTGGAGCTGGCGCTGCT
CCGTCCGGCCGACCAGGCGCCGGATCGCCAGTCCATCCAGGTCGCGTACGACTCGGCCCGCAGCCTGCTGGAGCTGATAG
GCGACATCCTGGACATTGCGAAGATCGAGGCGGGAAAATTCGACCTGGCGCCGGTGCGCACGGCGCTGCGCGCCCTGCCC
GAAGGGGCGATCCGCGTCTTCGACGGATTGGCGCGCCAAAAAGGCATAGAGCTGGTATTGAAGACCGACATCGTGGGCGT
CGACGATGTATTGATAGACCCCTTGCGCATGAAGCAAGTGCTCTCGAACCTGGTGGGTAACGCCATCAAGTTCACCACCG
AAGGCCAGGTTGTCCTTGCCGTGACCGCACGCCCCGACGGCGAGGCCGCGCACGTGCAGTTCAGCGTGAGCGACACCGGC
TGCGGCATCAGCGAGGCCGACCAACGGCAGCTGTTCAAACCGTTCTCGCAGGTGGGTGGCAGCGCCGAGGCCGGGCCGGC
GCCGGGCACCGGCCTGGGCCTGTCCATCAGCCGCCGCCTCGTCGAATTGATGGGGGGAACGCTGGTCATGCGCAGCGCGC
CAGGGGTGGGCACAACGGTTTCGGTGGACCTGAGGCTGACCATGGTCGAAAAATCCGTGCAGGCCACGCCGCCCGCTGCG
GCCGCTGCGGCCACGCCGTCCAAGCCGCAGGTATCGCTGCGCGTGCTGGTCGTCGATGACCACAAACCCAACCTGATGCT
GCTGCGCCAGCAGCTGGACTACCTGGGCCAGCGTGTCATCGCCGCCGACTCCGGCGAAGCCGCCCTGGCCCTGTGGCGCG
AGCATGCGTTCGACGTCGTGATCACCGATTGCAACATGCCCGGTATCAGCGGCTACGAATTGGCGCGCCGCATACGCGCC
GCCGAGGCCGCGCCCGGTTACGGACGTACGCGGTGCATTCTGTTCGGCTTCACGGCTTCGGCGCAGATGGACGAAGCGCA
GCACTGCCGCGCCGCCGGCATGGACGACTGCCTGTTCAAGCCGATCGGCGTGGACGCCTTGCGGCAACGCTTGAACGAAG
CCGTGGCACGGGCCGCGCTCCCCACGCCCCCCTCGCCCCAGGCTGCCGCGCCGGCCACGGACGACGCCACCCCGACGGCG
TTCTCGGCCGAATCGATTCTTGCCTTGACGCAGAACGATGAGGCGCTGATCCGGCAATTGCTCGAAGAAGTGATTCGCAC
CAACCGGGCGGACGTCGACCAATTGCAAAAGCTGCACCAGCAGGCCGATTGGCCGAAGGTCTCGGACATGGCGCACAGGC
TGGCCGGCGGCGCGCGCGTGGTCGATGCCAAGGCCATGATAGACACTGCGCTGGCGCTGGAGAAAAAAGCGCAAGGCCAG
GCTGGCCACTCGCCCGAAATCGACGGCCTGGTACGTACGCTTGCGGCGCAGTCCGCCGCGCTGGAGACGCAACTGCGCGC
CTGGCTGGAGCAACGGCCGCATCAAGATCAGCCCTGA
>Bb_B2496.seq
ATGCCCGCCCCGCACCGCCTGTACCCCCGCAGTCTGATCTGCCTGGCTCAGGCGCTATTGGCATGGGCTTTGCTGGCATG
GGCGCCCGCGCAGGCAAGCCAGGAGCTGACCCTGGTCGGCAAGGCTGCCGTTCCCGACGTCGAGGTCGCGCTCGACGGCG
ACGACTGGCGTTGGCTGGCCCGCAAGCGGGTACTGACGCTGGGTGTGTACGCACCGGACATTCCTCCGTTCGACGTCACC
TATGGCGAACGCTACGAAGGCCTGACGGCCGACTACATGGCGATCATCGCGCACAACCTGGGGATGCAGGCGAAAGTGCT
GCGATACCCCACGCGCGAACAAGCCCTCAGCGCGCTGGAAAGCGGGCAGATCGACCTCATCGGCACCGTCAATGGCACCG
ACGGCCGGCAACAGAGTCTGCGTCTGAGCGTTCCCTACGCCGCCGACCACCCGGTGATCGTCATGCCTATCGGCGCACGC
CACGTTCCAGCCTCGAACCTGGCCGGCCAGCGGCTGGCGGTCGACATCAACTACCTGCCCAAGGAAACGCTCGCACGGGC
CTACCCGCAGGCGACGCTGCATTACTTTCCCTCATCCGAGCAGGCGCTGGCCGCGGTGGCCTATGGGCAGGCCGACGTAT
TCATCGGCGATGCCCTGACCACCTCGCACCTCGTATCGCAAAGCTATTTCAATGACGTTCGCGCGGTCGCCCCGGCCCAT
ATCGCGACGGGCGGAGAATCCTTCGGCGTGCGCGCCGACAACACCCGCCTGCTGCGGGTGGTCAACGCCGTACTCGAAGC
CATTCCGCCTTCCGAACACCGCAGCCTGATCTACCGCTGGGGACTGGGCAGCAGCATTTCGCTCGATTTCGCGCACCCCG
CGTATTCCGCGCGCGAGCAGCAATGGATGGCAGACCACCCCGTCGTCAAGGTGGCGGTCCTGAATCTGTTCGCGCCCTTC
ACCCTGTTCCGCACCGACGAACAGTTCGGCGGGATCAGCGCCGCCGTGCTGCAGCTGCTGCAATTGCGCACCGGCCTGGA
CTTCGAGATCATCGGCGTCGACACGGTCGAGGAACTGATAGCCAAGCTGCGTTCGGGCGAAGCCGACATGGCCGGCGCCC
TGTTCGTCAACAGCGCGCGGGAGTCCTTCCTCAGTTTCAGCCGGCCGTATGTGCGCAATGGCATGGTGATCGTCACGCGC
CAGGACCCCGACGCGCCCGTCGACGCCGATCATCTGGACGGCCGCACGGTCGCGTTGGTGCGCAACAGCGCCGCCATTCC
TCTGCTGCAGCGGCGCTACCCCCAGGCGAAGGTGGTGACCGCCGACAACCCGAGCGAGGCGATGCTGATGGTGGCCAATG
GACAGGCCGACGCCGTCGTGCAGACGCAGATCAGCGCCAGCTATTACGTCAACCGCTACTTCGCCGGCAAGCTGCGCATC
GCCTCGGCGCTGGACCTGCCCCCGGCCGAGATCGCGCTGGCGACGACGCGCGGCCAGACCGAACTGATGTCCATCCTGAA
CAAGGCGCTCTACAGCATTTCGAACGACGAGCTCGCCTCCATCATCAGCCGCTGGCGCGGCAGCGACGGCGATCCGCGCA
CCTGGTACGCCTACCGCAACGAGATCTACCTGCTGATCGGGCTGGGCCTGTTGTCGGCCCTGCTGTTCCTGAGCTGGATC
GTCTACCTGCGGCGCCaGATCCGCCAGCGCAAGCGGGCCGAGCGGGCGCTGAACGACCAGCTGGAATTCATGCGCGTGCT
CATCGACGGCACGCCCAACCCCATCTATGTGCGCGATAAGGAAGGCCGCATGCTGTTGTGCAATGACGCCTACCTCGACA
CCTTTGGCGTGACTGCCGATGCGGTACTGGGCAAGACCATTCCGGAAGCCAACGTGGTGGGCGACCCGGCGCTGGCCCGC
GAAATGCACGAGTTCCTGCTCACCCGCGTGGCCGCCGAGCGCGAGCCGCGCTTCGAGGACCGCGATGTCACGCTGCACGG
CCGCACCCGCCATGTCTACCAGTGGACGATTCCGTACGGCGACTCGCTGGGCGAACTCAAGGGCATCATCGGCGGCTGGA
TCGACATCACCGAACGCGCCGAGCTGCTGCGCGAGCTGCACGACGCCAAGGAAAGCGCCGACGCCGCCAACCGGGCCAAG
ACCACGTTCCTGGCAACGATGAGCCACGAGATCCGCACGCCGATGAACGCGATCATCGGCATGCTGGAGCTGGCGCTGCT
CCGTCCGGCCGACCAGGAGCCGGATCGCCAGTCCATCCAGGTCGCGTACGACTCGGCCCGCAGCCTGCTGGAGCTGATAG
GCGACATCCTGGACATTGCGAAGATCGAGGCAGGAAAATTCGACCTGGCGCCGGTGCGCACGGCGCTGCGCGCCCTGCCC
GAAGGGGCGATCCGCGTCTTCGACGGATTGGCGCGCCAAAAAGGCATAGAGCTGGTATTGAAGACCGACATCGTGGGCGT
CGACGATGTATTGATAGACCCCTTGCGCATGAAGCAAGTGCTCTCGAACCTGGTGGGTAACGCCATCAAGTTCACCACCG
AAGGCCAGGTTGTCCTTGCCGTGACCGCACGCCCCGACGGCGAGGCCGCGCACGTGCAGTTCAGCGTGAGCGACACCGGC
TGCGGCATCAGCGAGGCCGACCAACGGCAGCTGTTCAAACCGTTCTCGCAGGTGGGTGGCAGCGCCGAGGCCGGGCCGGC
GCCGGGCACCGGCCTGGGCCTGTCCATCAGCCGCCGCCTCGTCGAATTGATGGGGGGAACGCTGGTCATGCGCAGCGCGC
CAGGGGTGGGCACAACGGTTTCGGTGGACCTGAGGCTGACCATGGTCGAAAAATCCGTGCAGGCCACGCCGCCCGCTGCG
GCCGCTGCGGCCACGCCGTCCAAGCCGCAGGTATCGCTGCGCGTGCTGGTCGTCGATGACCACAAACCCAACCTGATGCT
GCTGCGCCAGCAGCTGGACTACCTGGGCCAGCGTGTCATCGCCGCCGACTCCGGCGAAGCCGCCCTGGCCCTGTGGCGCG
AGCATGCGTTCGACGTCGTGATCACCGATTGCAACATGCCCGGTATCAGCGGCTACGAATTGGCGCGCCGCATACGCGCC
GCCGAGGCCGCGCCCGGTTACGGACGTACGCGGTGCATTCTGTTCGGCTTCACGGCTTCGGCGCAGATGGACGAAGCGCA
GCGCTGCCGCGCCGCCGGCATGGACGACTGCCTGTTCAAGCCGATCGGCGTGGACGCCTTGCGGCAACGCCTGAACGAAG
CCGTGGCACGGGCCGCGCTCCCCACGCCCCCCTCGCCCCAGGCTGCCGCGCCGGCCACGGACGACGCCACCCCGACGGCG
TTCTCGGCCGAATCGATTCTTGCCTTGACGCAGAACGATGAGGCGCTGATCCGGCAATTGCTCGAAGAAGTGATCCGCAC
CAACCGGGCGGACGTCGACCAATTGCAAAAGCTGCACCAGCAGGCCGATTGGCCGAAGGTCTCGGACATGGCGCACAGGC
TGGCCGGCGGCGCGCGCGTGGTCGATGCCAAGGCCATGATAGACACTGCGCTGGCGCTGGAGAAAAAAGCGCAAGGCCAG
GCTGGCCCCTCACCCGAAATCGACGGCCTGGTACGTACGCTTGCGGCGCAGTCCGCCGCGCTGGAGACGCAACTGCGCGC
CTGGCTGGAGCAACGGCCGCATCAAGATCAGCCCTGA
>Bb_B2498.seq
ATGCCCGCCCCGCACCGCCTGTACCCCCGCAGTCTGATCTGCCTGGCTCAGGCGCTATTGGCATGGGCTTTGCTGGCATG
GGCGCCCGCGCAGGCAAGCCAGGAGCTGACCCTGGTCGGCAAGGCTGCCGTTCCCAACGTCGAGATCACGCTCGACGGCG
ACGACTGGCGCTGGCTGGCGCGCAAGCGGGTGCTGACGCTGGGCGTGTACGCGCCGGACATTCCCCCGTTCGACGTCACC
TATGACGAGCGCTACGAAGGCCTGACGGCCGACTACATGGCGATCATCGCGCACAACCTGGGCGTCCAGGCAAAAGTGCT
GCGCTACCCCACGCGCGAGCAAGCCGTCGGCGCACTGGAAAGCGGACAGATCGACCTCATCGGCACCGTCAATGGCATCG
AGGGCCGGCTGCAGAGCCTGCGCCTGAGTGTTCCCTACGCGGCCGACCACCCGGTGCTGGTCATGCCCATCGGCGCGCGC
CGCGCTCCGCCCGCGGACCTGGCAGGCCAGCGGTTGGCGGTTGACGCCAACTACTTGCCCAGGGAAACGCTGCAGCAGGC
CTATCCCCAGGCAACGCTGCATTACTTCCCATCATCCGAACAGGCGCTGGCCGCGGTGGCCTATGGACAAGCCGACGTGT
TCATCGGCGATGCGCTGACCACCTCGCACCTCGTTTCGCAAAGCTACTTCAACGACGTTCGCGTCGTCGCCCCGGCCCAG
ATCGTGACGGGCGGGGAATCCTTCGGCGTGCGCGCCGACAATACCCGCCTGCTGCGGGTGGTCAATGCCGTGCTCGAAGC
CATTCCGGCCTCCGAGCGCCGCAGCCTGATCTACCGCTGGGGCCTGGGCAGCAGCATTTCGCTCGATTTCGCGCGCCCCG
CCTATTCAGCGCGCGAGCAGCAGTGGATGGCAAACCATCCGGTCGTCAAGGTGGCGGTCCTGAACCTGTTCGCGCCCTTC
ACCCTGTTTCGCACCGATGAACAGTTCGGCGGCATCAGTGCCGCCGTGCTGCAACTGCTGCAGTTGCGCACCGGCCTGGA
TTTCCAGATCATCGGCGTCGACACGGTCGAGGAGCTGATCGCCAAGCTGCGCTCGGGCGAAGCCGACATGGCCGGCGCCC
TGTTCGTCAATGCCGCGCGGGAATCCGTCCTCAGCTTCAGCCGGCCGTATGTGCGCAATGGTCTGGTGATCGTCACGCGC
CAGGACCCCGCCGCGCCCGCCGACGCCGATCACCTCGACGGCCGCACGATTGCGATGGTGCGCAACAGCGCCGCCATCCC
GCTCCTGCAGCAGCGCTATCCCCAGGCGAAGGTCGTGACCGCCGACAACCCGACCGAAGCCATGCTGCTGGTGGCCGATG
GCCAGGCCGACGCCGTCGTGCAGACGCAGATCAGCGCCAGCTACTACGTCAACCGCTACTTCGCCGGAAAACTGCGCATT
GCCTCGGCGCTGGACCTGCCGCCGGCCGAGATCGCGCTGGCGACGGCGCGCGGCCAGACCGAGCTGATATCCATCCTGAA
CAAGGCGCTCTACAGCATTTCGAACGACGAACTCGCCTCCATCGTCAGCCGCTGGCGCGGCAGCGACGGCGATCCGCGCA
CCTGGTACGCCTACCGCAACGAAATCTACCTGCTGATCGGGCTGGGCCTGTTGTCGGCCCTGCTGTTCCTGAGCTGGATC
GTCTACCTGCGGCGCCAGATCCGCCAGCGCAAGCGGGCCGAGCGGGCGCTGAACGACCAGCTGGAATTCATGCGCGTGCT
CATCGACGGCACGCCCAACCCCATCTATGTGCGCGATAAGGAAGGCCGCATGCTGTTGTGCAATGACGCCTACCTCGACA
CCTTTGGCGTGACTGCCGATGCGGTACTGGGCAAGACCATCCCGGAGGCCAACGTGGTGGGCGACCCGGCGCTGGCTCGC
GAGATGCACGAGTTCCTGCTCACGCGCATGGCCGCCGAGCGCGAGCCGCGCTTCGAGGACCGCGATGTCACGCTGCACGG
CCGCACCCGCCATGTCTACCAGTGGACGGTTCCGTACGGCGACTCGCTGGGCGAACTCAAGGGCATCATCGGCGGCTGGA
TCGACATTACCGAACGCGCCGAGCTGCTGCGCGAGCTGCACGACGCCAAGGAAAGCGCCGACGCCGCCAACCGGGCCAAG
ACCACGTTCCTGGCAACGATGAGCCACGAGATCCGCACGCCGATGAACGCGATCATCGGCATGCTGGAGCTGGCGCTGCT
CCGTCCGGCCGACCAGGAGCCGGACCGCCAGTCCATCCAGGTCGCGTACGACTCGGCCCGCAGCCTGCTGGAGCTGATAG
GCGACATCCTGGACATTGCGAAGATCGAGGCGGGAAAATTCGACCTGGCGCCGGTGCGCACGGCGCTGCGCGCCCTGCCC
GAAGGGGCGATCCGCGTCTTCGACGGGTTGGCGCGCCAGAAAGGCATAGAGCTGGTATTGAAGACCGACATCGTGGGCGT
GGACGATGTATTGATAGACCCCTTGCGCATGAAGCAAGTGCTCTCGAACCTGGTGGGCAACGCCATCAAGTTCACCACCG
AAGGCCAGGTTGTCCTTACCGTGACCGCGCGCCCCGACGGCGAGGCCGCGCACGTGCAGTTCAGCGTGAGCGACACCGGC
TGCGGCATCAGCGAGGCCGACCAACGGCAGCTGTTCAAACCGTTCTCGCAGGTGGGCGGCAGCGCCGAGGCCGGGCCGGC
GCCGGGCACTGGCCTGGGCCTGTCCATCAGCCGCCGCCTCGTCGAATTGATGGGGGGAACGCTGGTCATGCGCAGCGCGC
CAGGGGTGGGCACAACGGTTTCGGTGGACCTGAGGCTGACCATGGTCGAAAAATCCGCGCAGGCCACGCCGCCCGCTGCG
GCCGCTCAGGCCACGCCATCCAAGCCGCAGGTATCGCTGCGCGTGCTGGTCGTCGATGACCACAAGCCCAACCTGATGCT
GCTGCGCCAGCAGCTGGACTACCTGGGCCAGCGTGTCGTCGCCGCCGACTCCGGCGAAGCCGCCCTGGCCCTGTGGCACG
AGCATGCGTTCGACGTCGTGATCACCGATTGCAACATGCCCGGTATCAACGGCTACGAATtGGCGCGCCGCATACGCGCC
GCCGAGGCCGCGCCCGGTTACGGACGTACGCGGTGCATTCTGTTCGGCTTCACGGCTTCGGCGCAGATGGACGAAGCGCA
GCGCTGCCGCGCCGCCGGCATGGACGACTGCCTGTTCAAGCCGATCGGCGTGGACGCCTTGCGGCAACGCTTGAACGAAG
CCGCGGCACGGGCCGCGCTCCCCACGCCCCCCTCGCCCCAGGCTGCCGCGCCGGCCACGCACGACGCCACCCCGGCGGCG
TTCTCGGCCGAGTCGATTCTTGCCCTGACGCAGAACGATGAGGCGCTGATCCGGCAATTGCTCGAAGAAGTGATTCGCAC
CAACCGGGCGGACGTCGATCAATTGCAGAAGCTGCACCAGCAGGCCGATTGGCCGAAGGTCTCGGACATGGCGCACAGGC
TGGCCGGCGGCGCGCGCGTGGTCGATGCCAAGGCCATGATAGACACTGCGCTGGCGCTGGAGAAAAAAGCGCAAGGCCAG
GCTGGCCCCTCGCCCGAAATCGACGGCATGGTACGTACGCTTGCGGCGCAGTCCGCCGCGCTGGAGACGCAACTACGCGC
CTGGCTGGAGCAACGGCCGCATCAAGGCCAGCCCTGA
>Bb_B2501.seq
ATGCCCGCCCCGCACCGCCTGTACCCCCGCAgTCTGATCTGCCTGGCTCAGGCGCTATTGGCATGGGCTTTGCTGGCATG
GGCGCCCGCGCAGGCAAGCCAGGAGCTGACCCTGGTCGGCAAGGCTGCCGTTCCCGACGTCGAGATCACGCTCGACGGCG
ACGACTGGCGCTGGCTGGCGCGCAAGCGGGTGCTGACGCTGGGCGTGTACGCGCCGGACATTCCCCCGTTCGACGTCACC
TATGACGAGCGCTACGAAGGCCTGACGGCCGACTACATGGCGATCATCGCGCACAACCTGGGCGTCCAGGCAAAAGTGCT
GCGCTACCCCACGCGCGAGCAAGCCGTCGGCGCACTGGAAAGCGGACAGATCGACCTCATCGGCACCGTCAATGGCATCG
AGGGCCGGCTGCAGAGCCTGCGCCTGAGTGTTCCCTACGCGGCCGACCACCCGGTGCTGGTCATGCCCATCGGCGCGCGC
CGCGCTCCGCCCGCGGACCTGGCAGGCCAGCGGTTGGCGGTTGACGCCAACTACTTGCCCAGGGAAACGCTGCAGCAGGC
CTATCCCCAGGCAACGCTGCATTACTTCCCATCATCCGAACAGGCGCTGGCCGCGGTGGCCTATGGACAAGCCGACGTGT
TCATCGGCGATGCGCTGACCACCTCGCACCTCGTTTCGCAAAGCTACTTCAACGACGTTCGCGTCGTCGCCCCGGCCCAG
ATCGTGACGGGCGGGGAATCCTTCGGCGTGCGCGCCGACAATACCCGCCTGCTGCGGGTGGTCAATGCCGTGCTCGAAGC
CATTCCGGCCTCCGAGCGCCGCAGCCTGATCTACCGCTGGGGCCTGGGCAGCAGCATTTCGCTCGATTTCGCGCGCCCCG
CCTATTCAGCGCGCGAGCAGCAGTGGATGGCAAACCATCCGGTCGTCAAGGTGGCGGTCCTGAACCTGTTCGCGCCCTTC
ACCCTGTTTCGCACCGATGAACAGTTCGGCGGCATCAGTGCCGCCGTGCTGCAACTGCTGCAGTTGCGCACCGGCCTGGA
TTTCCAGATCATCGGCGTCGACACGGTCGAGGAGCTGATCGCCAAGCTGCGCTCGGGCGAAGCCGACATGGCCGGCGCCC
TGTTCGTCAATGCCGCGCGGGAATCCGTCCTCAGCTTCAGCCGGCCGTATGTGCGCAATGGTCTGGTGATCGTCACGCGC
CAGGACCCCGCCGCGCCCGCCGACGCCGATCACCTCGACGGCCGCACGATTGCGATGGTGCGCAACAGCGCCGCCATCCC
GCTCCTGCAGCAGCGCTATCCCCAGGCGAAGGTCGTGACCGCCGACAACCCGACCGAAGCCATGCTGCTGGTGGCCGATG
GCCAGGCCGACGCCGTCGTGCAGACGCAGATCAGCGCCAGCTACTACGTCAACCGCTACTTCGCCGGAAAACTGCGCATT
GCCTCGGCGCTGGACCTGCCGCCGGCCGAGATCGCGCTGGCGACGGCGCGCGGCCAGACCGAGCTGATATCCATCCTGAA
CAAGGCGCTCTACAGCATTTCGAACGACGAACTCGCCTCCATCGTCAGCCGCTGGCGCGGCAGCGACGGCGATCCGCGCA
CCTGGTACGCCTACCGCAACGAAATCTACCTGCTGATCGGGCTGGGCCTGTTGTCGGCCCTGCTGTTCCTGAGCTGGATC
GTCTACCTGCGGCGCCAGATCCGCCAGCGCAAGCGGGCCGAGCGGGCGCTGAACGACCAGCTGGAATTCATGCGCGTGCT
CATCGACGGCACGCCCAACCCCATCTATGTGCGCGATAAGGAAGGCCGCATGCTGTTGTGCAATGACGCCTACCTCGACA
CCTTTGGCGTGACTGCCGATGCGGTACTGGGCAAGACCATCCCGGAGGCCAACGTGGTGGGCGACCCGGCGCTGGCTCGC
GAGATGCACGAGTTCCTGCTCACGCGCATGGCCGCCGAGCGCGAGCCGCGCTTCGAGGACCGCGATGTCACGCTGCACGG
CCGCACCCGCCATGTCTACCAGTGGACGGTTCCGTACGGCGACTCGCTGGGCGAACTCAAGGGCATCATCGGCGGCTGGA
TCGACATTACCGAACGCGCCGAGCTGCTGCGCGAGCTGCACGACGCCAAGGAAAGCGCCGACGCCGCCAACCGGGCCAAG
ACCACGTTCCTGGCAACGATGAGCCACGAGATCCGCACGCCGATGAACGCGATCATCGGCATGCTGGAGCTGGCGCTGCT
CCGTCCGGCCGACCAGGAGCCGGACCGCCAGTCCATCCAGGTCGCGTACGACTCGGCCCGCAGCCTGCTGGAGCTGATAG
GCGACATCCTGGACATTGCGAAGATCGAGGCGGGAAAATTCGACCTGGCGCCGGTGCGCACGGCGCTGCGCGCCCTGCCC
GAAGGGGCGATCCGCGTCTTCGACGGGTTGGCGCGCCAGAAAGGCATAGAGCTGGTATTGAAGACCGACATCGTGGGCGT
GGACGATGTATTGATAGACCCCTTGCGCATGAAGCAAGTGCTCTCGAACCTGGTGGGCAACGCCATCAAGTTCACCACCG
AAGGCCAGGTTGTCCTTACCGTGACCGCGCGCCCCGACGGCGAGGCCGCGCACGTGCAGTTCAGCGTGAGCGACACCGGC
TGCGGCATCAGCGAGGCCGACCAACGGCAGCTGTTCAAACCGTTCTCGCAGGTGGGCGGCAGCGCCGAGGCCGGGCCGGC
GCCGGGCACTGGCCTGGGCCTGTCCATCAGCCGCCGCCTCGTCGAATTGATGGGGGGAACGCTGGTCATGCGCAGCGCGC
CAGGGGTGGGCACAACGGTTTCGGTGGACCTGAGGCTGACCATGGTCGAAAAATCCGCGCAGGCCACGCCGCCCGCTGCG
GCCGCTCAGGCCACGCCATCCAAGCCGCAGGTATCGCTGCGCGTGCTGGTCGTCGATGACCACAAGCCCAACCTGATGCT
GCTGCGCCAGCAGCTGGACTACCTGGGCCAGCGTGTCGTCGCCGCCGACTCCGGCGAAGCCGCCCTGGCCCTGTGGCACG
AGCATGCGTTCGACGTCGTGATCACCGATTGCAACATGCCCGGTATCAACGGCTACGAATTGGCGCGCCGCATACGCGCC
GCCGAGGCCGCGCCCGGTTACGGACGTACGCGGTGCATTCTGTTCGGCTTCACGGCTTCGGCGCAGATGGACGAAGCGCA
GCGCTGCCGCGCCGCCGGCATGGACGACTGCCTGTTCAAGCCGATCGGCGTGGACGCCTTGCGGCAACGCTTGAACGAAG
CCGCGGCACGGGCCGCGCTCCCCACGCCCCCCTCGCCCCAGGCTGCCGCGCCGGCCACGCACGACGCCACCCCGGCGGCG
TTCTCGGCCGAGTCGATTCTTGCCCTGACGCAGAACGATGAGGCGCTGATCCGGCAATTGCTCGAAGAAGTGATTCGCAC
CAACCGGGCGGACGTCGATCAATTGCAGAAGCTGCACCAGCAGGCCGATTGGCCGAAGGTCTCGGACATGGCGCACAGGC
TGGCCGGCGGCGCGCGCGTGGTCGATGCCAAGGCCATGATAGACACTGCGCTGGCGCTGGAGAAAAAAGCGCAAGGCCAG
GCTGGCCCCTCGCCCGAAATCGACGGCATGGTACGTACGCTTGCGGCGCAGTCCGCCGCGCTGGAGACGCAACTACGCGC
CTGGCTGGAGCAACGGCCGCATCAAGGCCAGCCCTGA
>Bb_B2508.seq
ATGCCCGCCCCGCACCGCCTGTACCCCCGCAGTCTGATCTGCCTGGCTCAGGCGCTATTGGCATGGGCTTTGCTGGCATG
GGCGCCCGCGCAGGCAAGCCAGGAGCTGACCCTGGTCGGCAAGGCTGCCGTTCCCGACGTCGAGATCACGCTCGACGGCG
ACGACTGGCGCTGGCTGGCGCGCAAGCGGGTGCTGACGCTGGGCGTGTACGCGCCGGACATTCCCCCGTTCGACGTCACC
TATGACGAGCGCTACGAAGGCCTGACGGCCGACTACATGGCGATCATCGCGCACAACCTGGGCGTCCAGGCAAAAGTGCT
GCGCTACCCCACGCGCGAGCAAGCCGTCGGCGCACTGGAAAGCGGACAGATCGACCTCATCGGCACCGTCAATGGCATCG
AGGGCCGGCTGCAGAGCCTGCGCCTGAGTGTTCCCTACGCGGCCGACCACCCGGTGCTGGTCATGCCCATCGGCGCGCGC
CGCGCTCCGCCCGCGGACCTGGCAGGCCAGCGGTTGGCGGTTGACGCCAACTACTTGCCCAGGGAAACGCTGCAGCAGGC
CTATCCCCAGGCAACGCTGCATTACTTCCCATCATCCGAACAGGCGCTGGCCGCGGTGGCCTATGGACAAGCCGACGTGT
TCATCGGCGATGCGCTGACCACCTCGCACCTCGTTTCGCAAAGCTACTTCAACGACGTTCGCGTCGTCGCCCCGGCCCAG
ATCGTGACGGGCGGGGAATCCTTCGGCGTGCGCGCCGACAATACCCGCCTGCTGCGGGTGGTCAATGCCGTGCTCGAAGC
CATTCCGGCCTCCGAGCGCCGCAGCCTGATCTACCGCTGGGGCCTGGGCAGCAGCATTTCGCTCGATTTCGCGCGCCCCG
CCTATtCAGCGCGCGAGCAGCAGTGGATGGCAAACCATCCGGTCGTCAAGGTGGCGGTCCTGAACCTGTTCGCGCCCTTC
ACCCTGTTTCGCACCGATGAACAGTTCGGCGGCATCAGTGCCGCCGTGCTGCAACTGCTGCAGTTGCGCACCGGCCTGGA
TTTCCAGATCATCGGCGTCGACACGGTCGAGGAGCTGATCGCCAAGCTGCGCTCGGGCGAAGCCGACATGGCCGGCGCCC
TGTTCGTCAATGCCGCGCGGGAATCCGTCCTCAGCTTCAGCCGGCCGTATGTGCGCAATGGTCTGGTGATCGTCACGCGC
CAGGACCCCGCCGCGCCCGCCGACGCCGATCACCTCGACGGCCGCACGATTGCGATGGTGCGCAACAGCGCCGCCATCCC
GCTCCTGCAGCAGCGCTATCCCCAGGCGAAGGTCGTGACCGCCGACAACCCGACCGAAGCCATGCTGCTGGTGGCCGATG
GCCAGGCCGACGCCGTCGTGCAGACGCAGATCAGCGCCAGCTACTACGTCAACCGCTACTTCGCCGGAAAACTGCGCATT
GCCTCGGCGCTGGACCTGCCGCCGGCCGAGATCGCGCTGGCGACGGCGCGCGGCCAGACCGAGCTGATATCCATCCTGAA
CAAGGCGCTCTACAGCATTTCGAACGACGAACTCGCCTCCATCGTCAGCCGCTGGCGCGGCAGCGACGGCGATCCGCGCA
CCTGGTACGCCTACCGCAACGAAATCTACCTGCTGATCGGGCTGGGCCTGTTGTCGGCCCTGCTGTTCCTGAGCTGGATC
GTCTACCTGCGGCGCCAGATCCGCCAGCGCAAGCGGGCCGAGCGGGCGCTGAACGACCAGCTGGAATTCATGCGCGTGCT
CATCGACGGCACGCCCAACCCCATCTATGTGCGCGATAAGGAAGGCCGCATGCTGTTGTGCAATGACGCCTACCTCGACA
CCTTTGGCGTGACTGCCGATGCGGTACTGGGCAAGACCATCCCGGAGGCCAACGTGGTGGGCGACCCGGCGCTGGCTCGC
GAGATGCACGAGTTCCTGCTCACGCGCATGGCCGCCGAGCGCGAGCCGCGCTTCGAGGACCGCGATGTCACGCTGCACGG
CCGCACCCGCCATGTCTACCAGTGGACGGTTCCGTACGGCGACTCGCTGGGCGAACTCAAGGGCATCATCGGCGGCTGGA
TCGACATTACCGAACGCGCCGAGCTGCTGCGCGAGCTGCACGACGCCAAGGAAAGCGCCGACGCCGCCAACCGGGCCAAG
ACCACGTTCCTGGCAACGATGAGCCACGAGATCCGCACGCCGATGAACGCGATCATCGGCATGCTGGAGCTGGCGCTGCT
CCGTCCGGCCGACCAGGAGCCGGACCGCCAGTCCATCCAGGTCGCGTACGACTCGGCCCGCAGCCTGCTGGAGCTGATAG
GCGACATCCTGGACATTGCGAAGATCGAGGCGGGAAAATTCGACCTGGCGCCGGTGCGCACGGCGCTGCGCGCCCTGCCC
GAAGGGGCGATCCGCGTCTTCGACGGGTTGGCGCGCCAGAAAGGCATAGAGCTGGTATTGAAGACCGACATCGTGGGCGT
GGACGATGTATTGATAGACCCCTTGCGCATGAAGCAAGTGCTCTCGAACCTGGTGGGCAACGCCATCAAGTTCACCACCG
AAGGCCAGGTTGTCCTTACCGTGACCGCGCGCCCCGACGGCGAGGCCGCGCACGTGCAGTTCAGCGTGAGCGACACCGGC
TGCGGCATCAGCGAGGCCGACCAACGGCAGCTGTTCAAACCGTTCTCGCAGGTGGGCGGCAGCGCCGAGGCCGGGCCGGC
GCCGGGCACTGGCCTGGGCCTGTCCATCAGCCGCCGCCTCGTCGAATTGATGGGGGGAACGCTGGTCATGCGCAGCGCGC
CAGGGGTGGGCACAACGGTTTCGGTGGACCTGAGGCTGACCATGGTCGAAAAATCCGCGCAGGCCACGCCGCCCGCTGCG
GCCGCTCAGGCCACGCCATCCAAGCCGCAGGTATCGCTGCGCGTGCTGGTCGTCGATGACCACAAGCCCAACCTGATGCT
GCTGCGCCAGCAGCTGGACTACCTGGGCCAGCGTGTCGTCGCCGCCGACTCCGGCGAAGCCGCCCTGGcCCTGTGGCACG
AGCATGCGTTCGACGTCGTGATCACCGATTGCAACATGCCCGGTATCAACGGCTACGAATTGGCGCGCCGCATACGCGCC
GCCGAGGCCGCGCCCGGTTACGGACGTACGCGGTGCATTCTGTTCGGCTTCACGGCTTCGGCGCAGATGGACGAAGCGCA
GCGCTGCCGCGCCGCCGGCATGGACGACTGCCTGTTCAAGCCGATCGGCGTGGACGCCTTGCGGCAACGCTTGAACGAAG
CCGCGGCACGGGCCGCGCTCCCCACGCCCCCCTCGCCCCAGGCTGCCGCGCCGGCCACGCACGACGCCACCCCGGCGGCG
TTCTCGGCCGAGTCGATTCTTGCCCTGACGCAGAACGATGAGGCGCTGATCCGGCAATTGCTCGAAGAAGTGATTCGCAC
CAACCGGGCGGACGTCGATCAATTGCAGAAGCTGCACCAGCAGGCCGATTGGCCGAAGGTCTCGGACATGGCGCACAGGC
TGGCCGGCGGCGCGCGCGTGGTCGATGCCAAGGCCATGATAGACACTGCGCTGGCGCTGGAGAAAAAAGCGCAAGGCCAG
GCTGGCCCCTCGCCCGAAATCGACGGCATGGTACGTACGCTTGCGGCGCAGTCCGCCGCGCTGGAGACGCAACTACGCGC
CTGGCTGGAGCAACGGCCGCATCAAGGCCAGCCCTGA
>Bb_B2511.seq
ATGCCCGCCCCGCACCGCCTGTACCCCCGCAGTCTGATCTGCCTGGCTCAGGCGCTATTGGCATGGGCTTTGCTGGCATG
GGCGCCCGCGCAGGCAAGCCAGGAGCTGACCCTGGTCGGCAAGGCTGCCGTTCCCGACGTCGAGATCACGCTCGACGGCG
ACGACTGGCGCTGGCTGGCGCGCAAGCGGGTGCTGACGCTGGGCGTGTACGCGCCGGACATTCCCCCGTTCGACGTCACC
TATGACGAGCGCTACGAAGGCCTGACGGCCGACTACATGGCGATCATCGCGCACAACCTGGGCGTCCAGGCAAAAGTGCT
GCGCTACCCCACGCGCGAGCAAGCCGTCGGCGCACTGGAAAGCGGACAGATCGACCTCATCGGCACCGTCAATGGCATCG
AGGGCCGGCTGCAGAGCCTGCGCCTGAGTGTTCCCTACGCGGCCGACCACCCGGTGCTGGTCATGCCCATCGGCGCGCGC
CGCGCTCCGCCCGCGGACCTGGCAGGCCAGCGGTTGGCGGTTGACGCCAACTACTTGCCCAGGGAAACGCTGCAGCAGGC
CTATCCCCAGGCAACGCTGCATTACTTCCCATCATCCGAACAGGCGCTGGCCGCGGTGGCCTATGGACAAGCCGACGTGT
TCATCGGCGATGCGCTGACCACCTCGCACCTCGTTTCGCAAAGCTACTTCAACGACGTTCGCGTCGTCGCCCCGGCCCAG
ATCGTGACGGGCGGGGAATCCTTCGGCGTGCGCGCCGACAATACCCGCCTGCTGCGGGTGGTCAATGCCGTGCTCGAAGC
CATTCCGGCCTCCGAGCGCCGCAGCCTGATCTACCGCTGGGGCCTGGGCAGCAGCATTTCGCTCGATTTCGCGCGCCCCG
CCTATTCAGCGCGCGAGCAGCAGTGGATGGCAAACCATCCGGTCGTCAAGGTGGCGGTCCTGAACCTGTTCGCGCCCTTC
ACCCTGTTTCGCACCGATGAACAGTTCGGCGGCATCAGTGCCGCCGTGCTGCAACTGCTGCAGTTGCGCACCGGCCTGGA
TTTCCAGATCATCGGCGTCGACACGGTCGAGGAGCTGATCGCCAAGCTGCGCTCGGGCGAAGCCGACATGGCCGGCGCCC
TGTTCGTCAATGCCGCGCGGGAATCCGTCCTCAGCTTCAGCCGGCCGTATGTGCGCAATGGTCTGGTGATCGTCACGCGC
CAGGACCCCGCCGCGCCCGCCGACGCCGATCACCTCGACGGCCGCACGATTGCGATGGTGCGCAACAGCGCCGCCATCCC
GCTCCTGCAGCAGCGCTATCCCCAGGCGAAGGTCGTGACCGCCGACAACCCGACCGAAGCCATGCTGCTGGTGGCCGATG
GCCAGGCCGACGCCGTCGTGCAGACGCAGATCAGCGCCAGCTACTACGTCAACCGCTACTTCGCCGGAAAACTGCGCATT
GCCTCGGCGCTGGACCTGCCGCCGGCCGAGATCGCGCTGGCGACGGCGCGCGGCCAGACCGAGCTGATATCCATCCTGAA
CAAGGCGCTCTACAGCATTTCGAACGACGAACTCGCCTCCATCGTCAGCCGCTGGCGCGGCAGCGACGGCGATCCGCGCA
CCTGGTACGCCTACCGCAACGAAATCTACCTGCTGATCGGGCTGGGCCTGTTGTCGGCCCTGCTGTTCCTGAGCTGGATC
GTCTACCTGCGGCGCCAGATCCGCCAGCGCAAGCGGGCCGAGCGGGCGCTGAACGACCAGCTGGAATTCATGCGCGTGCT
CATCGACGGCACGCCCAACCCCATCTATGTGCGCGATAAGGAAGGCCGCATGCTGTTGTGCAATGACGCCTACCTCGACA
CCTTTGGCGTGACTGCCGATGCGGTACTGGGCAAGACCATCCCGGAGGCCAACGTGGTGGGCGACCCGGCGCTGGCTCGC
GAGATGCACGAGTTCCTGCTCACGCGCATGGCCGCCGAGCGCGAGCCGCGCTTCGAGGACCGCGATGTCACGCTGCACGG
CCGCACCCGCCATGTCTACCAGTGGACGGTTCCGTACGGCGACTCGCTGGGCGAACTCAAGGGCATCATCGGCGGCTGGA
TCGACATTACCGAACGCGCCGAGCTGCTGCGCGAGCTGCACGACGCCAAGGAAAGCGCCGACGCCGCCAACCGGGCCAAG
ACCACGTTCCTGGCAACGATGAGCCACGAGATCCGCACGCCGATGAACGCGATCATCGGCATGCTGGAGCTGGCGCTGCT
CCGTCCGGCCGACCAGGAGCCGGACCGCCAGTCCATCCAGGTCGCGTACGACTCGGCCCGCAGCCTGCTGGAGCTGATAG
GCGACATCCTGGACATTGCGAAGATCGAGGCGGGAAAATTCGACCTGGCGCCGGTGCGCACGGCGCTGCGCGCCCTGCCC
GAAGGGGCGATCCGCGTCTTCGACGGGTTGGCGCGCCAGAAAGGCATAGAGCTGGTATTGAAGACCGACATCGTGGGCGT
GGACGATGTATTGATAGACCCCTTGCGCATGAAGCAAGTGCTCTCGAACCTGGTGGGCAACGCCATCAAGTTCACCACCG
AAGGCCAGGTTGTCCTTACCGTGACCGCGCGCCCCGACGGCGAGGCCGCGCACGTGCAGTTCAGCGTGAGCGACACCGGC
TGCGGCATCAGCGAGGCCGACCAACGGCAGCTGTTCAAACCGTTCTCGCAGGTGGGCGGCAGCGCCGAGGCCGGGCCGGC
GCCGGGCACTGGCCTGGGCCTGTCCATCAGCCGCCGCCTCGTCGAATTGATGGGGGGAACGCTGGTCATGCGCAGCGCGC
CAGGGGTGGGCACAACGGTTTCGGTGGACCTGAGGCTGACCATGGTCGAAAAATCCGCGCAGGCCACGCCGCCCGCTGCG
GCCGCTCAGGCCACGCCATCCAAGCCGCAGGTATCGCTGCGCGTGCTGGTCGTCGATGACCACAAGCCCAACCTGATGCT
GCTGCGCCAGCAGCTGGACTACCTGGGCCAGCGTGTCGTCGCCGCCGACTCCGGCGAAGCCGCCCTGGCCCTGTGGCACG
AGCATGCGTTCGACGTCGTGATCACCGATTGCAACATGCCCGGTATCAACGGCTACGAATTGGCGCGCCGCATACGCGCC
GCCGAGGCCGCGCCCGGTTACGGACGTACGCGGTGCATTCTGTTCGGCTTCACGGCTTCGGCGCAGATGGACGAAGCGCA
GCGCTGCCGCGCCGCCGGCATGGACGACTGCCTGTTCAAGCCGATCGGCGTGGACGCCTTGCGGCAACGCTTGAACGAAG
CCGCGGCACGGGCCGCGCTCCCCACGCCCCCCTCGCCCCAGGCTGCCGCGCCGGCCACGCACGACGCCACCCCGGCGGCG
TTCTCGGCCGAGTCGATTCTTGCCCTGACGCAGAACGATGAGGCGCTGATCCGGCAATTGCTCGAAGAAGTGATTCGCAC
CAACCGGGCGGACGTCGATCAATTGCAGAAGCTGCACCAGCAGGCCGATTGGCCGAAGGTCTCGGACATGGCGCACAGGC
TGGCCGGCGGCGCGCGCGTGGTCGATGCCAAGGCCATGATAGACACTGCGCTGGCGCTGGAGAAAAAAGCGCAAGGCCAG
GCTGGCCCCTCGCCCGAAATCGACGGCATGGTACGTACGCTTGCGGCGCAGTCCGCCGCGCTGGAGACGCAACTACGCGC
CTGGCTGGAGCAACGGCCGCATCAAGGCCAGCCCTGA
>Bb_B2588.seq
ATGCCCGCCCCGCACCGCCTGTACCCCCGCAGTCTGATCTGCCTGGCTCAGGCGCTATTGGCATGGGCTTTGCTGGCATG
GGCGCCCGCGCAGGCAAGCCAGGAGCTGACCCTGGTCGGCAAGGCTGCCGTTCCCGACGTCGAGGTCGCGCTCGACGGCG
ACGACTGGCGTTGGCTGGCCCGCAAGCGGGTACTGACGCTGGGTGTGTACGCACCGGACATTCCTCCGTTCGACGTCACC
TATGGCGAACGCTACGAAGGCCTGACGGCCGACTACATGGCGATCATCGCGCACAACCTGGGGATGCAGGCGAAAGTGCT
GCGATACCCCACGCGCGAACAAGCCCTCAGCGCGCTGGAAAGCGGGCAGATCGACCTCATCGGCACCGTCAATGGCACCG
ACGGCCGGCAACAGAGTCTGCGTCTGAGCGTTCCCTACGCCGCCGACCACCCGGTGATCGTCATGCCCATCGGCGCACGC
CACGTTCCAGCCTCGAACCTGGCCGGCCAGCGGCTGGCGGTCGACATCAACTACCTGCCCAAGGAAACGCTCGCACGGGC
CTACCCGCAGGCAACGCTGCATTACTTTCCCTCATCCGAGCAGGCGCTGGCCGCGGTGGCCTATGGGCAGGCCGACGTAT
TCATCGGCGATGCCCTGACCACCTCGCACCTCGTATCGCAAAGCTATTTCAATGACGTTCGCGTAGTCGCCCCGGCCCAT
ATCGCGACGGGCGGAGAATCCTTCGGCGTGCGCGCCGACAACACCCGCCTGCTGCGGGTGGTCAACGCCGTACTCGAAGC
CATTCCGCCTTCCGAACACCGCAGCCTGATCTACCGCTGGGGACTGGGCAGCAGCATTTCGCTCGATTTCGCGCACCCCG
CGTATTCCGCGCGCGAGCAGCAATGGATGGCAGACCACCCCGTCGTCAAGGTGGCGGTCCTGAATCTGTTCGCGCCCTTC
ACCCTGTTCCGCACCGACGAACAGTTCGGCGGGATCAGCGCCGCCGTGCTGCAGCTGCTGCAATTGCGCACCGGCCTGGA
CTTCGAGATCATCGGCGTCGACACGGTCGAGGAACTGATAGCCAAGCTGCGTTCGGGCGAAGCCGACATGGCCGGCGCCC
TGTTCGTCAACAGCGCGCGGGAGTCCTTCCTCAGTTTCAGCCGGCCGTATGTGCGCAATGGCATGGTGATCGTCACGCGC
CAGGACCCCGACGCGCCCGTCGACGCCGATCATCTGGACGGCCGCACGGTCGCGTTGGTGCGCAACAGCGCCGCCATTCC
TCTGCTGCAGCGGCGCTATCCCCAGGCGAAGGTGGTGACCGCCGACAACCCGAGCGAGGCGATGCTGATGGTGGCCAATG
GACAGGCCGACGCCGTCGTGCAGACGCAGATCAGCGCCAGCTATTACGTCAACCGCTACTTCGCCGGCAAGCTGCGCATC
GCCTCGGCGCTGGACCTGCCTCCGGCCGAGATCGCGCTGGCGACGACGCGCGGCCAGACCGAACTGATGTCCATCCTGAA
CAAGGCGCTCTACAGCATTTCGAACGACGAGCTCGCCTCCATCATCAGCCGCTGGCGCGGCAGCGACGGCGATCCGCGCA
CCTGGTACGCCTACCGCAACGAGATCTACCTGCTGATCGGGCTGGGCCTGTTGTCGGCCCTGCTGTTCCTGAGCTGGATC
GTCTACCTGCGGCGCCaGATCCGCCAGCGCAAGCGGGCCGAGCGGGCGCTGAACGACCAGCTGGAATTCATGCGCGTGCT
CATCGACGGCACGCCCAACCCCATCTATGTGCGCGATAAGGAAGGCCGCATGCTGTTGTGCAATGACGCCTACCTCGACA
CCTTTGGCGTGACTGCCGATGCGGTACTGGGCAAGACCATTCCGGAAGCCAACGTGGTGGGCGACCCGGCGCTGGCCCGC
GAAATGCACGAGTTCCTGCTCACGCGCGTGGCCGCCGAGCGCGAGCCGCGCTTCGAGGACCGCGATGTCACGCTGCACGG
CCGCACCCGCCATGTCTACCAGTGGACGATTCCGTACGGCGACTCGCTGGGCGAACTCAAGGGCATCATCGGCGGCTGGA
TCGACATCACCGAACGCGCCGAGCTGCTGCGCGAGCTGCACGACGCCaAGGAAAGCGCCGACGCCGCCAACCGGGCCAAG
ACCACGTTCCTGGCAACGATGAGCCACGAGATCCGCACGCCGATGAACGCGATCATCGGCATGCTGGAGCTGGCGCTGCT
CCGTCCGGCCGACCAGGAGCCGGATCGCCAGTCCATCCAGGTCGCGTACGACTCGGCCCGCAGCCTGCTGGAGCTGATAG
GCGACATCCTGGACATTGCGAAGATCGAGGCGGGAAAATTCGACCTGGCGCCGGTGCGCACGGCGCTGCGCGCCCTGCCC
GAAGGGGCGATCCGCGTCTTCGACGGATTGGCGCGCCAAAAAGGCATAGAGCTGGTATTGAAGACCGACATCGTGGGCGT
CGACGATGTCTTGATAGACCCCTTGCGCATGAAGCAAGTGCTCTCGAACCTGGTGGGCAACGCCATCAAGTTCACCACCG
AAGGCCAGGTTGTCCTTGCCGTGACCGCACGCCCCGACGGCGAGGCCGCGCACGTGCAGTTCAGCGTGAGCGACACCGGC
TGCGGCATCAGCGAGGCCGACCAACGGCAGCTGTTCAAACCGTTCTCGCAGGTGGGTGGCAGCGCCGAGGCCGGGCCGGC
GCCGGGCACCGGCCTGGGCCTGTCCATCAGCCGCCGCCTCGTCGAATTGATGGGGGGAACGCTGGTCATGCGCAGCGCGC
CAGGGGTGGGCACAACGGTTTCGGTGGACCTGAGGCTGACCATGGTCGAAAAATCCGTGCAGGCCACGCCGCCCGCTGCG
GCCGCTGCGGCCACGCCGTCCAAGCCGCAGGTATCGCTGCGCGTGCTGGTCGTCGATGACCACAAACCCAACCTGATGCT
GCTGCGCCAGCAGCTGGACTACCTGGGCCAGCGTGTCATCGCCGCCGACTCCGGCGAAGCCGCCCTGGCCCTGTGGCGCG
AGCATGCGTTCGACGTCGTGATCACCGATTGCAACATGCCCGGTATCAGCGGCTACGAATTGGCGCGCCGCATACGCGCC
GCCGAGGCCGCGCCCGGTTACGGACGTACGCGGTGCATTCTGTTCGGCTTCACGGCTTCGGCGCAGATGGACGAAGCGCA
GCGCTGCCGCGCCGCCGGCATGGACGACTGCCTGTTCAAGCCGATCGGCGTGGACGCCTTGCGGCAACGCTTGAACGAAG
CCGTGGCACGGGCCGCGCTCCCCACGCCCCCCTCGCCCCAGGCTGCCGCGCCGGCCACGCACGACGCCACCCCGACGGCG
TTCTCGGCCGAGTCGATTCTTGCCTTGACGCAGAACGATGAGGCGCTGATCCGGCAATTGCTCGAAGAAGTGATTCGCAC
CAACCGGGCGGACGTCGACCAATTGCAAAAGCTGCACCAGCAGGCCGATTGGCCGAAGGTCTCGGACATGGCGCACAGGC
TGGCCGGCGGCGCGCGCGTGGTCGATGCCAAGGCCATGATAGACACTGCGCTGGCGCTGGAGAAAAAAGCGCAAGGCCAG
GCTGGCCACTCGCCCGAAATCGACGGCCTGGTACGTACGCTTGCGGCGCAGTCCGCCGCGCTGGAGACGCAACTGCGCGC
CTGGCTGGAGCAACGGCCGCATCAAGATCAGCCCTGA
>Bb_RB50
atgcccgccccgcaccgcctgtacccccgcagtctgatctgcctggctcaggcgctattggcatgggctttgctggcatg
ggcgcccgcgcaggcaagccaggagctgaccctggtcggcaaggctgccgttcccgacgtcgagatcgcgctcgacggcg
acgactggcgctggctggcgcgcaagcgggtgctgacgctgggcgtgtacgcgccggacattcccccgttcgacgtcacc
tatgacgagcgctacgaaggcctgacggccgactacatggcgatcatcgcacacaacctgggcgtccaggcaaaagtgct
gcgctaccccacgcgcgagcaagccgtcggcgcactggaaagcggacagatcgacctcatcggcaccgtcaatggcatcg
agggccggctgcagagcctgcgcctgagtgttccctacgcggccgaccacccggtgctggtcatgcccatcggcgcgcgc
cgcgctccgcccgcggacctggcaggccagcggttggcggttgacgccaactacttgcccagggaaacgctgcagcaggc
ctatccccaggcaacgctgcattacttcccatcgtccgaacaggcgctggccgcggtggcctatggacaagccgacgtgt
tcatcggcgatgcgctgaccacctcgcacctcgtttcgcaaagctacttcaacgacgttcgcgtcgtcgccccggcccag
atcgtgacgggcggggaatccttcggcgtgcgcgccgacaatacccgcctgctgcgggtggtcaatgccgtactcgaagc
cattccggcctccgagcgccgcagcctgatctaccgctggggcctgggcagcagcatttcgctcgatttcgcgcgccccg
cctattcagcgcgcgagcagcagtggatggcaaaccatccggtcgtcaaggtggcggtcctgaacctgttcgcgcccttc
accctgtttcgcaccgatgaacagttcggcggcatcagtgccgccgtgctgcaactgctgcagttgcgcaccggcctgga
tttccagatcatcggcgtcgacacggtcgaggagctgatcgccaagctgcgctcgggcgaagccgacatggccggcgccc
tgttcgtcaatgccgcgcgggaatccgtcctcagcttcagccggccgtatgtgcgcaatggcatggtgatcgtcacgcgc
caggaccccgccgcgcccgccgacgccgatcacctcgacggccgcacgattgcgatggtgcgcaacagcgccgccatccc
gctcctgcagcagcgctatccccaggcgaaggtcgtgaccgccgacaacccgaccgaagccatgctgctggtggccgatg
gccaggccgacgccgtcgtgcagacgcagatcagcgccagctactacgtcaaccgctacttcgccggaaaactgcgcatt
gcctcggcgctggacctgccgccggccgagatcgcgctggcgacggcgcgcggccagaccgagctgatatccatcctgaa
caaggcgctctacagcatttcgaacgacgaactcgcctccatcgtcagccgctggcgcggcagcgacggcgatccgcgca
cctggtacgcctaccgcaacgagatctacctgctgatcgggctgggcctgttgtcggccctgctgttcctgagctggatc
gtctacctgcggcgccagatccgccagcgcaagcgggccgagcgggcgctgaacgaccagctggaattcatgcgcgtgct
catcgacggcacgcccaaccccatctatgtgcgcgataaggaaggccgcatgctgttgtgcaatgacgcctacctcgaca
cctttggcgtgactgccgatgcggtactgggcaagaccatcccggaggccaacgtggtgggcgacccggcgctggctcgc
gagatgcacgagttcctgctcacgcgcatgtccgccgagcgcgagccgcgcttcgaggaccgcgatgtcacgctgcacgg
ccgcacccgccatgtctaccagtggacggttccgtacggcgactcgctgggcgaactcaagggcatcatcggcggctgga
tcgacattaccgaacgcgccgagctgctgcgcgagctgcacgacgccaaggaaagcgccgacgccgccaaccgggccaag
accacgttcctggcaacgatgagccacgagatccgcacgccgatgaacgcgatcatcggcatgctggagctggcgctgct
ccgtccggccgaccaggagccggaccgccagtccatccaggtcgcgtacgactcggcccgcagcctgctggagctgatag
gcgacatcctggacattgcgaagatcgaggcgggaaaattcgacctggcgccggtgcgcacggcgctgcgcgccctaccc
gaaggggcgatccgcgtcttcgacgggttggcgcgccagaaaggcatagagctggtattgaagaccgacatcgtgggcgt
ggacgatgtattgatagaccccttgcgcatgaagcaagtgctctcgaacctggtgggcaacgccatcaagttcaccaccg
aaggccaggttgtccttaccgtgaccgcgcgccccgacggcgaggccgcgcacgtgcagttcagcgtgagcgacaccggc
tgcggcatcagcgaggccgaccaacggcagctgttcaaaccgttctcgcaggtgggcggcagcgccgaggccgggccggc
gccgggcactggcctgggcctgtccatcagccgccgcctcgtcgaattgatggggggaacgctggtcatgcgcagcgcgc
caggggtgggcacaacggtttcggtggacctgaggctgaccatggtcgaaaaatccgcgcaggccacgccgcccgctgcg
gccgctcaggccacgccatccaagccgcaggtatcgctgcgcgtgctggtcgtcgatgaccacaagcccaacctgatgct
gctgcgccagcagctggactacctgggccagcgtgtcgtcgccgccgactccggcgaagccgccctggccctgtggcacg
agcatgcgttcgacgtcgtgatcaccgattgcaacatgcccggtatcaacggctacgaattggcgcgccgcatacgcgcc
gccgaggccgcgcccggttacggacgtacgcggtgcattctgttcggcttcacggcttcggcgcagatggacgaagcgca
gcgctgccgcgccgccggcatggacgactgcctgttcaagccgatcggcgtggacgccttgcggcaacgcttgaacgaag
ccgcggcacgggccgcgctccccacgcccccctcgccccaggctgccgcgccggccacgcacgacgccaccccggcggcg
ttctcggccgagtcgattcttgccctgacgcagaacgatgaggcgctcatccggcaattgctcgaagaactgattcgcac
caaccgggcggacgtcgatcaattgcagaagctgcaccagcaggccgattggccgaaggtctcggacatggcgcacaggc
tggccggcggcgcgcgcgtggtcgatgccaaggccatgatagacactgcgctggcgctggaaaaaaaagcgcaaggccag
gctggcccctcgcccgaaatcgacggcatggtacgtacgcttgcggcgcagtccgccgcgctggagacgcaactacgcgc
ctggctggagcaacggccgcatcaaggccagccctga
>Bp_B0005.seq
ATGCCCGCCCCGCACCGCCTGTACCCCCGCAGTCTGATCTGCCTGGCTCAGGCGCTATTGGCATGGGCTTTGCTGGCATG
GGCGCCCGCGCAGGCAAGCCAGGAGCTGACCCTGGTCGGCAAGGCTGCCGTTCCCGACGTCGAGGTCGCGCTCGACGGCG
ACGACTGGCGTTGGCTGGCCCGCAAGCGGGTACTGACGCTGGGTGTGTACGCACCGGACATTCCTCCGTTCGACGTCACC
TATGGCGAACGCTACGAAGGCCTGACGGCCGACTACATGGCGATCATCGCGCACAACCTGGGGATGCAGGCGAAAGTGCT
GCGATACCCCACGCGCGAACAAGCCCTCAGCGCGCTGGAAAGCGGGCAGATCGACCTCATCGGCACCGTCAATGGCACGG
ACGGCCGGCAACAGAGCCTGCGTCTGAGCGTTCCCTACGCCGCCGACCACCCGGTGATCGTCATGCCCATCGGCGCACGC
CACGTTCCAGCCTCGAACCTGGCCGGCCAGCGGCTGGCGGTCGACATCAACTACCTGCCCAAGGAAACGCTCGCACGGGC
CTACCCGCAGGCTACGCTGCATTACTTCCCCTCATCCGAGCAGGCGCTGGCCGCGGTGGCCTATGGGCAGGCCGACGTAT
TCATCGGCGATGCCCTGACCACCTCGCACCTCGTATCGCAAAGCTATTTCAATGACGTTCGCGTAGTCGCCCCGGCCCAT
ATCGCGACGGGCGGAGAATCCTTCGGCGTGCGCGCCGACAACACCCGCCTGCTGCGGGTGGTCAACGCCGTACTCGAAGC
CATTCCGCCTTCCGAACACCGCAGCCTGATCTACCGCTGGGGACTGGGCAGCAGCATTTCGCTCGATTTCGCGCACCCCG
CGTATTCCGCGCGCGAGCAGCAATGGATGGCAGACCACCCCGTCGTCAAGGTGGCGGTCCTGAATCTGTTCGCGCCCTTC
ACCCTGTTCCGCACCGACGAACAGTTCGGCGGGATCAGCGCCGCCGTGCTGCAGCTGCTGCAATTGCGCACCGGCCTGGA
CTTCGAGATCATCGGCGTCGACACGGTCGAGGAACTGATAGCCAAGCTGCGTTCGGGCGAAGCCGACATGGCCGGCGCCC
TGTTCGTCAACAGCGCGCGGGAGTCCTTCCTCAGTTTCAGCCGGCCGTATGTGCGCAATGGCATGGTGATCGTCACGCGC
CAGGACCCCGACGCGCCCGTCGACGCCGATCATCTGGACGGCCGCACGGTCGCGTTGGTGCGCAACAGCGCCGCCATTCC
CCTGCTGCAGCGGCGCTATCCCCAGGCGAAGGTGGTGACCGCCGACAACCCGAGCGAGGCGATGCTGATGGTGGCCAATG
GACAGGCCGACGCCGTCGTGCAGACGCAGATCAGCGCCAGCTATTACGTCAACCGCTACTTCGCCGGCAAGCTGCGCATC
GCCTCGGCGCTGGACCTGCCCCCGGCCGAGATCGCGCTGGCGACGACGCGCGGCCAGACCGAACTGATGTCCATCCTGAA
CAAGGCGCTCTACAGCATTTCGAACGACGAGCTCGCCTCCATCATCAGCCGCTGGCGCGGCAGCGACGGCGATCCGCGCA
CCTGGTACGCCTACCGCAACGAGATCTACCTGCTGATCGGGCTGGGCCTGTTGTCGGCCCTGCTGTTCCTGAGCTGGATC
GTCTACCTGCGGCGCCAGATCCGCCAGCGCAAGCGGGCCGAGCGGGCGCTGAACGACCAGCTGGAATTCATGCGCGTGCT
CATCGACGGCACGCCTAACCCCATCTATGTGCGCGATAAGGAAGGCCGCATGCTGTTGTGCAATGACGCCTACCTCGACA
CCTTTGGCGTGACTGCCGATGCGGTACTGGGCAAGACCATTCCGGAAGCCAACGTGGTGGGCGACCCGGCGCTGGCCCGC
GAAATGCACGAGTTCCTGCTCACGCGCGTGGCCGCCGAGCGCGAGCCGCGCTTCGAGGACCGCGATGTCACGCTGCACGG
CCGCACCCGCCATGTCTACCAGTGGACGATTCCGTACGGCGACTCGCTGGGCGAACTCAAGGGCATCATCGGCGGCTGGA
TCGACATCACCGAACGCGCCGAGCTGCTGCGCGAGCTGCACGACGCCAAGGAAAGCGCCGACGCCGCCAACCGGGCCAAG
ACCACGTTCCTGGCAACGATGAGCCACGAGATCCGCACGCCGATGaACGCGATCATCGGCATGCTGGAGCTGGCGCTGCT
CCGTCCGACCGACCAGGAGCCGGATCGCCAGTCCATCCAGGTCGCGTACGACTCGGCCCGCAGCCTGCTGGAGCTGATAG
GCGACATCCTGGACATTGCGAAGATCGAGGCGGGAAAATTCGACCTGGCGCCGGTGCGCACGGCGCTGCGCGTCCTGCCC
GAAGGGGCGATCCGCGTCTTCGACGGATTGGCGCGCCAAAAAGGCATAGAGCTGGTATTGAAGACCGACATCGTGGGCGT
CGACGATGTATTGATAGACCCCTTGCGCATGAAGCAAGTGCTCTCGAACCTGGTGGGCAACGCCATCAAGTTCACCACCG
AAGGCCAGGTTGTCCTTGCCGTGACCGCACGCCCCGACGGCGACGCCGCGCACGTGCAGTTCAGCGTGAGCGACACCGGC
TGCGGCATCAGCGAGGCCGACCAACGGCAGCTGTTCAAACCGTTCTCGCAAGTGGGTGGCAGCGCCGAGGCCGGGCCGGC
GCCGGGCACCGGCCTGGGCCTGTCCATCAGCCGGCGCCTCGTCGAATTGATGGGGGGAACGCTGGTCATGCGCAGCGCGC
CAGGGGTGGGCACAACGGTTTCGGTGGACCTGAGGCTGACCATGGTCGAAAAATCCGTGCAGGCCGCGCCGCCCGCTGCG
GCCACTGCGGCCACGCCGTCCAAGCCGCAGGTATCGCTGCGCGTGCTGGTCGTCGATGACCACAAACCCAACCTGATGCT
GCTGCGCCAGCAGCTGGACTACCTGGGCCAGCGTGTCATCGCCGCCGACTCCGGCGAAGCCGCCCTGGcCCTGTGGCGCG
AGCATGCGTTCGACGTCGTGATCACCGATTGCAACATGCCCGGTATCAGCGGCTACGAATtGGCGCGCCGCATACGCGCC
GCCGAGGCCGCGCCCGGTTACGGACGTACGCGGTGCATTCTGTTCGGCTTCACGGCTTCGGCGCAGATGGACGAAGCGCA
GCGCTGCCGCGCCGCCGGCATGGACGACTGCCTGTTCAAGCCGATCGGCGTGGACGCCTTGCGGCAACGCTTGAACGAAG
CCGTGGCACGGGCCGCGCTCCCCACGCCCCCCTCGCCCCAGGCTGCCGCGCCGGCCACGGACGACGCCACCCCGACGGCG
TTCTCGGCCGAGTCGATTCTTGCCTTGACGCAGAACGATGAGGCGCTGATCCGGCAATTGCTCGAAGAAGTGATTCGCAC
CAACCGGGCGGACGTCGACCAATTGCAAAAGCTGCACCAGCAGGCCGATTGGCCGAAGGTCTCGGACATGGCGCACAGGC
TGGCCGGCGGCGCGCGCGTGGTCGATGCCAAGGCCATGATAGACACTGTGCTGGCGCTGGAGAAAAAAGCGCAAGGCCAG
GCTGGCCCCTCACCCGAAATCGACGGCCTGGTACGTACGCTTGCGGCGCAGTCCGCCGCGCTGGAGACGCAACTGCGCGC
CTGGCTGGAGCAACGGCCGCATCAAGATCAGCCCTGA
>Bp_B0336.seq
ATGCCCGCCCCGCACCGCCtGTACCCCCGCAGTCTGATCTGCCTGGCTCAGGCGCTATTGGCATGGGCTTTGCTGGCATG
GGCGCCCGCGCAGGCAAGCCAGGAGCTGACCCTGGTCGGCAAGGCTGCCGTTCCCGACGTCGAGGTCGCGCTCGACGGCG
ACGACTGGCGTTGGCTGGCCCGCAAGCGGGTACTGACGCTGGGTGTGTACGCACCGGACATTCCTCCGTTCGACGTCACC
TATGGCGAACGCTACGAAGGCCTGACGGCCGACTACATGGCGATCATCGCGCACAACCTGGGGATGCAGGCGAAAGTGCT
GCGATACCCCACGCGCGAACAAGCCCTCAGCGCGCTGGAAAGCGGGCAGATCGACCTCATCGGCACCGTCAATGGCACGG
ACGGCCGGCAACAGAGCCTGCGTCTGAGCGTTCCCTACGCCGCCGACCACCCGGTGATCGTCATGCCCATCGGCGCACGC
CACGTTCCAGCCTCGAACCTGGCCGGCCAGCGGCTGGCGGTCGACATCAACTACCTGCCCAAGGAAACGCTCGCACGGGC
CTACCCGCAGGCTACGCTGCATTACTTCCCCTCATCCGAGCAGGCGCTGGCCGCGGTGGCCTATGGGCAGGCCGACGTAT
TCATCGGCGATGCCCTGACCACCTCGCACCTCGTATCGCAAAGCTATTTCAATGACGTTCGCGTAGTCGCCCCGGCCCAT
ATCGCGACGGGCGGAGAATCCTTCGGCGTGCGCGCCGACAACACCCGCCTGCTGCGGGTGGTCAACGCCGTACTCGAAGC
CATTCCGCCTTCCGAACACCGCAGCCTGATCTACCGCTGGGGACTGGGCAGCAGCATTTCGCTCGATTTCGCGCACCCCG
CGTATTCCGCGCGCGAGCAGCAATGGATGGCAGACCACCCCGTCGTCAAGGTGGCGGTCCTGAATCTGTTCGCGCCCTTC
ACCCTGTTCCGCACCGACGAACAGTTCGGCGGGATCAGCGCCGCCGTGCTGCAGCTGCTGCAATTGCGCACCGGCCTGGA
CTTCGAGATCATCGGCGTCGACACGGTCGAGGAACTGATAGCCAAGCTGCGTTCGGGCGAAGCCGACATGGCCGGCGCCC
TGTTCGTCAACAGCGCGCGGGAGTCCTTCCTCAGTTTCAGCCGGCCGTATGTGCGCAATGGCATGGTGATCGTCACGCGC
CAGGACCCCGACGCGCCCGTCGACGCCGATCATCTGGACGGCCGCACGGTCGCGTTGGTGCGCAACAGCGCCGCCATTCC
CCTGCTGCAGCGGCGCTATCCCCAGGCGAAGGTGGTGACCGCCGACAACCCGAGCGAGGCGATGCTGATGGTGGCCAATG
GACAGGCCGACGCCGTCGTGCAGACGCAGATCAGCGCCAGCTATTACGTCAACCGCTACTTCGCCGGCAAGCTGCGCATC
GCCTCGGCGCTGGACCTGCCCCCGGCCGAGATCGCGCTGGCGACGACGCGCGGCCAGACCGAACTGATGTCCATCCTGAA
CAAGGCGCTCTACAGCATTTCGAACGACGAGCTCGCCTCCATCATCAGCCGCTGGCGCGGCAGCGACGGCGATCCGCGCA
CCTGGTACGCCTACCGCAACGAGATCTACCTGCTGATCGGGCTGGGCCTGTTGTCGGCCCTGCTGTTCCTGAGCTGGATC
GTCTACCTGCGGCGCCAGATCCGCCAGCGCAAGCGGGCCGAGCGGGCGCTGAACGACCAGCTGGAATTCATGCGCGTGCT
CATCGACGGCACGCCTAACCCCATCTATGTGCGCGATAAGGAAGGCCGCATGCTGTTGTGCAATGACGCCTACCTCGACA
CCTTTGGCGTGACTGCCGATGCGGTACTGGGCAAGACCATTCCGGAAGCCAACGTGGTGGGCGACCCGGCGCTGGCCCGC
GAAATGCACGAGTTCCTGCTCACGCGCGTGGCCGCCGAGCGCGAGCCGCGCTTCGAGGACCGCGATGTCACGCTGCACGG
CCGCACCCGCCATGTCTACCAGTGGACGATTCCGTACGGCGACTCGCTGGGCGAACTCAAGGGCATCATCGGCGGCTGGA
TCGACATCACCGAACGCGCCGAGCTGCTGCGCGAGCTGCACGACGCCAAGGAAAGCGCCGACGCCGCCAACCGGGCCAAG
ACCACGTTCCTGGCAACGATGAGCCACGAGATCCGCACGCCGATGaACGCGATCATCGGCATGCTGGAGCTGGCGCTGCT
CCGTCCGACCGACCAGGAGCCGGATCGCCAGTCCATCCAGGTCGCGTACGACTCGGCCCGCAGCCTGCTGGAGCTGATAG
GCGACATCCTGGACATTGCGAAGATCGAGGCGGGAAAATTCGACCTGGCGCCGGTGCGCACGGCGCTGCGCGTCCTGCCC
GAAGGGGCGATCCGCGTCTTCGACGGATTGGCGCGCCAAAAAGGCATAGAGCTGGTATTGAAGACCGACATCGTGGGCGT
CGACGATGTATTGATAGACCCCTTGCGCATGAAGCAAGTGCTCTCGAACCTGGTGGGCAACGCCATCAAGTTCACCACCG
AAGGCCAGGTTGTCCTTGCCGTGACCGCACGCCCCGACGGCGACGCCGCGCACGTGCAGTTCAGCGTGAGCGACACCGGC
TGCGGCATCAGCGAGGCCGACCAACGGCAGCTGTTCAAACCGTTCTCGCAAGTGGGTGGCAGCGCCGAGGCCGGGCCGGC
GCCGGGCACCGGCCTGGGCCTGTCCATCAGCCGGCGCCTCGTCGAATTGATGGGGGGAACGCTGGTCATGCGCAGCGCGC
CAGGGGTGGGCACAACGGTTTCGGTGGACCTGAGGCTGACCATGGTCGAAAAATCCGTGCAGGCCGCGCCGCCCGCTGCG
GCCACTGCGGCCACGCCGTCCAAGCCGCAGGTATCGCTGCGCGTGCTGGTCGTCGATGACCACAAACCCAACCTGATGCT
GCTGCGCCAGCAGCTGGACTACCTGGGCCAGCGTGTCATCGCCGCCGACTCCGGCGAAGCCGCCCTGGCCCTGTGGCGCG
AGCATGCGTTCGACGTCGTGATCACCGATTGCAACATGCCCGGTATCAGCGGCTACGAATTGGCGCGCCGCATACGCGCC
GCCGAGGCCGCGCCCGGTTACGGACGTACGCGGTGCATTCTGTTCGGCTTCACGGCTTCGGCGCAGATGGACGAAGCGCA
GCGCTGCCGCGCCGCCGGCATGGACGACTGCCTGTTCAAGCCGATCGGCGTGGACGCCTTGCGGCAACGCTTGAACGAAG
CCGTGGCACGGGCCGCGCTCCCCACGCCCCCCTCGCCCCAGGCTGCCGCGCCGGCCACGGACGACGCCACCCCGACGGCG
TTCTCGGCCGAGTCGATTCTTGCCTTGACGCAGAACGATGAGGCGCTGATCCGGCAATTGCTCGAAGAAGTGATTCGCAC
CAACCGGGCGGACGTCGACCAATTGCAAAAGCTGCACCAGCAGGCCGATTGGCCGAAGGTCTCGGACATGGCGCACAGGC
TGGCCGGCGGCGCGCGCGTGGTCGATGCCAAGGCCATGATAGACACTGTGCTGGCGCTGGAGAAAAAAGCGCAAGGCCAG
GCTGGCCCCTCACCCGAAATCGACGGCCTGGTACGTACGCTTGCGGCGCAGTCCGCCGCGCTGGAGACGCAACTGCGCGC
CTGGCTGGAGCAACGGCCGCATCAAGATCAGCCCTGA
>Bp_B0402.seq
ATGCCCGCCCCGCACCGCCTGTACCCCCGCAGTCTGATCTGCCTGGCTCAGGCGCTATTGGCATGGGCTTTGCTGGCATG
GGCGCCCGCGCAGGCAAGCCAGGAGCTGACCCTGGTCGGCAAGGCTGCCGTTCCCGACGTCGAGGTCGCGCTCGACGGCG
ACGACTGGCGTTGGCTGGCCCGCAAGCGGGTACTGACGCTGGGTGTGTACGCACCGGACATTCCTCCGTTCGACGTCACC
TATGGCGAACGCTACGAAGGCCTGACGGCCGACTACATGGCGATCATCGCGCACAACCTGGGGATGCAGGCGAAAGTGCT
GCGATACCCCACGCGCGAACAAGCCCTCAGCGCGCTGGAAAGCGGGCAGATCGACCTCATCGGCACCGTCAATGGCACGG
ACGGCCGGCAACAGAGCCTGCGTCTGAGCGTTCCCTACGCCGCCGACCACCCGGTGATCGTCATGCCCATCGGCGCACGC
CACGTTCCAGCCTCGAACCTGGCCGGCCAGCGGCTGGCGGTCGACATCAACTACCTGCCCAAGGAAACGCTCGCACGGGC
CTACCCGCAGGCTACGCTGCATTACTTCCCCTCATCCGAGCAGGCGCTGGCCGCGGTGGCCTATGGGCAGGCCGACGTAT
TCATCGGCGATGCCCTGACCACCTCGCACCTCGTATCGCAAAGCTATTTCAATGACGTTCGCGTAGTCGCCCCGGCCCAT
ATCGCGACGGGCGGAGAATCCTTCGGCGTGCGCGCCGACAACACCCGCCTGCTGCGGGTGGTCAACGCCGTACTCGAAGC
CATTCCGCCTTCCGAACACCGCAGCCTGATCTACCGCTGGGGACTGGGCAGCAGCATTTCGCTCGATTTCGCGCACCCCG
CGTATTCCGCGCGCGAGCAGCAATGGATGGCAGACCACCCCGTCGTCAAGGTGGCGGTCCTGAATCTGTTCGCGCCCTTC
ACCCTGTTCCGCACCGACGAACAGTTCGGCGGGATCAGCGCCGCCGTGCTGCAGCTGCTGCAATTGCGCACCGGCCTGGA
CTTCGAGATCATCGGCGTCGACACGGTCGAGGAACTGATAGCCAAGCTGCGTTCGGGCGAAGCCGACATGGCCGGCGCCC
TGTTCGTCAACAGCGCGCGGGAGTCCTTCCTCAGTTTCAGCCGGCCGTATGTGCGCAATGGCATGGTGATCGTCACGCGC
CAGGACCCCGACGCGCCCGTCGACGCCGATCATCTGGACGGCCGCACGGTCGCGTTGGTGCGCAACAGCGCCGCCATTCC
CCTGCTGCAGCGGCGCTATCCCCAGGCGAAGGTGGTGACCGCCGACAACCCGAGCGAGGCGATGCTGATGGTGGCCAATG
GACAGGCCGACGCCGTCGTGCAGACGCAGATCAGCGCCAGCTATTACGTCAACCGCTACTTCGCCGGCAAGCTGCGCATC
GCCTCGGCGCTGGACCTGCCCCCGGCCGAGATCGCGCTGGCGACGACGCGCGGCCAGACCGAACTGATGTCCATCCTGAA
CAAGGCGCTCTACAGCATTTCGAACGACGAGCTCGCCTCCATCATCAGCCGCTGGCGCGGCAGCGACGGCGATCCGCGCA
CCTGGTACGCCTACCGCAACGAGATCTACCTGCTGATCGGGCTGGGCCTGTTGTCGGCCCTGCTGTTCCTGAGCTGGATC
GTCTACCTGCGGCGCCAGATCCGCCAGCGCAAGCGGGCCGAGCGGGCGCTGAACGACCAGCTGGAATTCATGCGCGTGCT
CATCGACGGCACGCCTAACCCCATCTATGTGCGCGATAAGGAAGGCCGCATGCTGTTGTGCAATGACGCCTACCTCGACA
CCTTTGGCGTGACTGCCGATGCGGTACTGGGCAAGACCATTCCGGAAGCCAACGTGGTGGGCGACCCGGCGCTGGCCCGC
GAAATGCACGAGTTCCTGCTCACGCGCGTGGCCGCCGAGCGCGAGCCGCGCTTCGAGGACCGCGATGTCACGCTGCACGG
CCGCACCCGCCATGTCTACCAGTGGACGATTCCGTACGGCGACTCGCTGGGCGAACTCAAGGGCATCATCGGCGGCTGGA
TCGACATCACCGAACGCGCCGAGCTGCTGCGCGAGCTGCACGACGCCAAGGAAAGCGCCGACGCCGCCAACCGGGCCAAG
ACCACGTTCCTGGCAACGATGAGCCACGAGATCCGCACGCCGATGaACGCGATCATCGGCATGCTGGAGCTGGCGCTGCT
CCGTCCGACCGACCAGGAGCCGGATCGCCAGTCCATCCAGGTCGCGTACGACTCGGCCCGCAGCCTGCTGGAGCTGATAG
GCGACATCCTGGACATTGCGAAGATCGAGGCGGGAAAATTCGACCTGGCGCCGGTGCGCACGGCGCTGCGCGTCCTGCCC
GAAGGGGCGATCCGCGTCTTCGACGGATTGGCGCGCCAAAAAGGCATAGAGCTGGTATTGAAGACCGACATCGTGGGCGT
CGACGATGTATTGATAGACCCCTTGCGCATGAAGCAAGTGCTCTCGAACCTGGTGGGCAACGCCATCAAGTTCACCACCG
AAGGCCAGGTTGTCCTTGCCGTGACCGCACGCCCCGACGGCGACGCCGCGCACGTGCAGTTCAGCGTGAGCGACACCGGC
TGCGGCATCAGCGAGGCCGACCAACGGCAGCTGTTCAAACCGTTCTCGCAAGTGGGTGGCAGCGCCGAGGCCGGGCCGGC
GCCGGGCACCGGCCTGGGCCTGTCCATCAGCCGGCGCCTCGTCGAATTGATGGGGGGAACGCTGGTCATGCGCAGCGCGC
CAGGGGTGGGCACAACGGTTTCGGTGGACCTGAGGCTGACCATGGTCGAAAAATCCGTGCAGGCCGCGCCGCCCGCTGCG
GCCACTGCGGCCACGCCGTCCAAGCCGCAGGTATCGCTGCGCGTGCTGGTCGTCGATGACCACAAACCCAACCTGATGCT
GCTGCGCCAGCAGCTGGACTACCTGGGCCAGCGTGTCATCGCCGCCGACTCCGGCGAAGCCGCCCTGGCCCTGTGGCGCG
AGCATGCGTTCGACGTCGTGATCACCGATtGCAACATGCCCGGTATCAGCGGCTACGAATtGGCGCGCCGCATACGCGCC
GCCGAGGCCGCGCCCGGTTACGGACGTACGCGGTGCATTCTGTTCGGCTTCACGGCTTCGGCGCAGATGGACGAAGCGCA
GCGCTGCCGCGCCGCCGGCATGGACGACTGCCTGTTCAAGCCGATCGGCGTGGACGCCTTGCGGCAACGCTTGAACGAAG
CCGTGGCACGGGCCGCGCTCCCCACGCCCCCCTCGCCCCAGGCTGCCGCGCCGGCCACGGACGACGCCACCCCGACGGCG
TTCTCGGCCGAGTCGATTCTTGCCTTGACGCAGAACGATGAGGCGCTGATCCGGCAATTGCTCGAAGAAGTGATTCGCAC
CAACCGGGCGGACGTCGACCAATTGCAAAAGCTGCACCAGCAGGCCGATTGGCCGAAGGTCTCGGACATGGCGCACAGGC
TGGCCGGCGGCGCGCGCGTGGTCGATGCCAAGGCCATGATAGACACTGTGCTGGCGCTGGAGAAAAAAGCGCAAGGCCAG
GCTGGCCCCTCACCCGAAATCGACGGCCTGGTACGTACGCTTGCGGCGCAGTCCGCCGCGCTGGAGACGCAACTGCGCGC
CTGGCTGGAGCAACGGCCGCATCAAGATCAGCCCTGA
>Bp_B0403.seq
ATGCCCGCCCCGCACCGCCTGTACCCCCGCAGTCTGATCTGCCTGGCTCAGGCGCTATTGGCATGGGCTTTGCTGGCATG
GGCGCCCGCGCAGGCAAGCCAGGAGCTGACCCTGGTCGGCAAGGCTGCCGTTCCCGACGTCGAGGTCGCGCTCGACGGCG
ACGACTGGCGTTGGCTGGCCCGCAAGCGGGTACTGACGCTGGGTGTGTACGCACCGGACATTCCTCCGTTCGACGTCACC
TATGGCGAACGCTACGAAGGCCTGACGGCCGACTACATGGCGATCATCGCGCACAACCTGGGGATGCAGGCGAAAGTGCT
GCGATACCCCACGCGCGAACAAGCCCTCAGCGCGCTGGAAAGCGGGCAGATCGACCTCATCGGCACCGTCAATGGCACGG
ACGGCCGGCAACAGAGCCTGCGTCTGAGCGTTCCCTACGCCGCCGACCACCCGGTGATCGTCATGCCCATCGGCGCACGC
CACGTTCCAGCCTCGAACCTGGCCGGCCAGCGGCTGGCGGTCGACATCAACTACCTGCCCAAGGAAACGCTCGCACGGGC
CTACCCGCAGGCTACGCTGCATTACTTCCCCTCATCCGAGCAGGCGCTGGCCGCGGTGGCCTATGGGCAGGCCGACGTAT
TCATCGGCGATGCCCTGACCACCTCGCACCTCGTATCGCAAAGCTATTTCAATGACGTTCGCGTAGTCGCCCCGGCCCAT
ATCGCGACGGGCGGAGAATCCTTCGGCGTGCGCGCCGACAACACCCGCCTGCTGCGGGTGGTCAACGCCGTACTCGAAGC
CATTCCGCCTTCCGAACACCGCAGCCTGATCTACCGCTGGGGACTGGGCAGCAGCATTTCGCTCGATTTCGCGCACCCCG
CGTATTCCGCGCGCGAGCAGCAATGGATGGCAGACCACCCCGTCGTCAAGGTGGCGGTCCTGAATCTGTTCGCGCCCTTC
ACCCTGTTCCGCACCGACGAACAGTTCGGCGGGATCAGCGCCGCCGTGCTGCAGCTGCTGCAATTGCGCACCGGCCTGGA
CTTCGAGATCATCGGCGTCGACACGGTCGAGGAACTGATAGCCAAGCTGCGTTCGGGCGAAGCCGACATGGCCGGCGCCC
TGTTCGTCAACAGCGCGCGGGAGTCCTTCCTCAGTTTCAGCCGGCCGTATGTGCGCAATGGCATGGTGATCGTCACGCGC
CAGGACCCCGACGCGCCCGTCGACGCCGATCATCTGGACGGCCGCACGGTCGCGTTGGTGCGCAACAGCGCCGCCATTCC
CCTGCTGCAGCGGCGCTATCCCCAGGCGAAGGTGGTGACCGCCGACAACCCGAGCGAGGCGATGCTGATGGTGGCCAATG
GACAGGCCGACGCCGTCGTGCAGACGCAGATCAGCGCCAGCTATTACGTCAACCGCTACTTCGCCGGCAAGCTGCGCATC
GCCTCGGCGCTGGACCTGCCCCCGGCCGAGATCGCGCTGGCGACGACGCGCGGCCAGACCGAACTGATGTCCATCCTGAA
CAAGGCGCTCTACAGCATTTCGAACGACGAGCTCGCCTCCATCATCAGCCGCTGGCGCGGCAGCGACGGCGATCCGCGCA
CCTGGTACGCCTACCGCAACGAGATCTACCTGCTGATCGGGCTGGGCCTGTTGTCGGCCCTGCTGTTCCTGAGCTGGATC
GTCTACCTGCGGCGCCAGATCCGCCAGCGCAAGCGGGCCGAGCGGGCGCTGAACGACCAGCTGGAATTCATGCGCGTGCT
CATCGACGGCACGCCTAACCCCATCTATGTGCGCGATAAGGAAGGCCGCATGCTGTTGTGCAATGACGCCTACCTCGACA
CCTTTGGCGTGACTGCCGATGCGGTACTGGGCAAGACCATTCCGGAAGCCAACGTGGTGGGCGACCCGGCGCTGGCCCGC
GAAATGCACGAGTTCCTGCTCACGCGCGTGGCCGCCGAGCGCGAGCCGCGCTTCGAGGACCGCGATGTCACGCTGCACGG
CCGCACCCGCCATGTCTACCAGTGGACGATTCCGTACGGCGACTCGCTGGGCGAACTCAAGGGCATCATCGGCGGCTGGA
TCGACATCACCGAACGCGCCGAGCTGCTGCGCGAGCTGCACGACGCCAAGGAAAGCGCCGACGCCGCCAACCGGGCCAAG
ACCACGTTCCTGGCAACGATGAGCCACGAGATCCGCACGCCGATGaACGCGATCATCGGCATGCTGGAGCTGGCGCTGCT
CCGTCCGACCGACCAGGAGCCGGATCGCCAGTCCATCCAGGTCGCGTACGACTCGGCCCGCAGCCTGCTGGAGCTGATAG
GCGACATCCTGGACATTGCGAAGATCGAGGCGGGAAAATTCGACCTGGCGCCGGTGCGCACGGCGCTGCGCGTCCTGCCC
GAAGGGGCGATCCGCGTCTTCGACGGATTGGCGCGCCAAAAAGGCATAGAGCTGGTATTGAAGACCGACATCGTGGGCGT
CGACGATGTATTGATAGACCCCTTGCGCATGAAGCAAGTGCTCTCGAACCTGGTGGGCAACGCCATCAAGTTCACCACCG
AAGGCCAGGTTGTCCTTGCCGTGACCGCACGCCCCGACGGCGACGCCGCGCACGTGCAGTTCAGCGTGAGCGACACCGGC
TGCGGCATCAGCGAGGCCGACCAACGGCAGCTGTTCAAACCGTTCTCGCAAGTGGGTGGCAGCGCCGAGGCCGGGCCGGC
GCCGGGCACCGGCCTGGGCCTGTCCATCAGCCGGCGCCTCGTCGAATTGATGGGGGGAACGCTGGTCATGCGCAGCGCGC
CAGGGGTGGGCACAACGGTTTCGGTGGACCTGAGGCTGACCATGGTCGAAAAATCCGTGCAGGCCGCGCCGCCCGCTGCG
GCCACTGCGGCCACGCCGTCCAAGCCGCAGGTATCGCTGCGCGTGCTGGTCGTCGATGACCACAAACCCAACCTGATGCT
GCTGCGCCAGCAGCTGGACTACCTGGGCCAGCGTGTCATCGCCGCCGACTCCGGCGAAGCCGCCCTGGcCCTGTGGCGCG
AGCATGCGTTCGACGTCGTGATCACCGAtTGCAACATGCCCGGTATCAGCGGCTACGAATTGGCGCGCCGCATACGCGCC
GCCGAGGCCGCGCCCGGTTACGGACGTACGCGGTGCATTCTGTTCGGCTTCACGGCTTCGGCGCAGATGGACGAAGCGCA
GCGCTGCCGCGCCGCCGGCATGGACGACTGCCTGTTCAAGCCGATCGGCGTGGACGCCTTGCGGCAACGCTTGAACGAAG
CCGTGGCACGGGCCGCGCTCCCCACGCCCCCCTCGCCCCAGGCTGCCGCGCCGGCCACGGACGACGCCACCCCGACGGCG
TTCTCGGCCGAGTCGATTCTTGCCTTGACGCAGAACGATGAGGCGCTGATCCGGCAATTGCTCGAAGAAGTGATTCGCAC
CAACCGGGCGGACGTCGACCAATTGCAAAAGCTGCACCAGCAGGCCGATTGGCCGAAGGTCTCGGACATGGCGCACAGGC
TGGCCGGCGGCGCGCGCGTGGTCGATGCCAAGGCCATGATAGACACTGTGCTGGCGCTGGAGAAAAAAGCGCAAGGCCAG
GCTGGCCCCTCACCCGAAATCGACGGCCTGGTACGTACGCTTGCGGCGCAGTCCGCCGCGCTGGAGACGCAACTGCGCGC
CTGGCTGGAGCAACGGCCGCATCAAGATCAGCCCTGA
>Bp_B0540.seq
ATGCCCGCCCCGCACCGCCTGTACCCCCGCAGTCTGATCTGCCTGGCTCAGGCGCTATTGGCATGGGCTTTGCTGGCATG
GGCGCCCGCGCAGGCAAGCCAGGAGCTGACCCTGGTCGGCAAGGCTGCCGTTCCCGACGTCGAGGTCGCGCTCGACGGCG
ACGACTGGCGTTGGCTGGCCCGCAAGCGGGTACTGACGCTGGGTGTGTACGCACCGGACATTCCTCCGTTCGACGTCACC
TATGGCGAACGCTACGAAGGCCTGACGGCCGACTACATGGCGATCATCGCGCACAACCTGGGGATGCAGGCGAAAGTGCT
GCGATACCCCACGCGCGAACAAGCCCTCAGCGCGCTGGAAAGCGGGCAGATCGACCTCATCGGCACCGTCAATGGCACGG
ACGGCCGGCAACAGAGCCTGCGTCTGAGCGTTCCCTACGCCGCCGACCACCCGGTGATCGTCATGCCCATCGGCGCACGC
CACGTTCCAGCCTCGAACCTGGCCGGCCAGCGGCTGGCGGTCGACATCAACTACCTGCCCAAGGAAACGCTCGCACGGGC
CTACCCGCAGGCTACGCTGCATTACTTCCCCTCATCCGAGCAGGCGCTGGCCGCGGTGGCCTATGGGCAGGCCGACGTAT
TCATCGGCGATGCCCTGACCACCTCGCACCTCGTATCGCAAAGCTATTTCAATGACGTTCGCGTAGTCGCCCCGGCCCAT
ATCGCGACGGGCGGAGAATCCTTCGGCGTGCGCGCCGACAACACCCGCCTGCTGCGGGTGGTCAACGCCGTACTCGAAGC
CATTCCGCCTTCCGAACACCGCAGCCTGATCTACCGCTGGGGACTGGGCAGCAGCATTTCGCTCGATTTCGCGCACCCCG
CGTATTCCGCGCGCGAGCAGCAATGGATGGCAGACCACCCCGTCGTCAAGGTGGCGGTCCTGAATCTGTTCGCGCCCTTC
ACCCTGTTCCGCACCGACGAACAGTTCGGCGGGATCAGCGCCGCCGTGCTGCAGCTGCTGCAATTGCGCACCGGCCTGGA
CTTCGAGATCATCGGCGTCGACACGGTCGAGGAACTGATAGCCAAGCTGCGTTCGGGCGAAGCCGACATGGCCGGCGCCC
TGTTCGTCAACAGCGCGCGGGAGTCCTTCCTCAGTTTCAGCCGGCCGTATGTGCGCAATGGCATGGTGATCGTCACGCGC
CAGGACCCCGACGCGCCCGTCGACGCCGATCATCTGGACGGCCGCACGGTCGCGTTGGTGCGCAACAGCGCCGCCATTCC
CCTGCTGCAGCGGCGCTATCCCCAGGCGAAGGTGGTGACCGCCGACAACCCGAGCGAGGCGATGCTGATGGTGGCCAATG
GACAGGCCGACGCCGTCGTGCAGACGCAGATCAGCGCCAGCTATTACGTCAACCGCTACTTCGCCGGCAAGCTGCGCATC
GCCTCGGCGCTGGACCTGCCCCCGGCCGAGATCGCGCTGGCGACGACGCGCGGCCAGACCGAACTGATGTCCATCCTGAA
CAAGGCGCTCTACAGCATTTCGAACGACGAGCTCGCCTCCATCATCAGCCGCTGGCGCGGCAGCGACGGCGATCCGCGCA
CCTGGTACGCCTACCGCAACGAGATCTACCTGCTGATCGGGCTGGGCCTGTTGTCGGCCCTGCTGTTCCTGAGCTGGATC
GTCTACCTGCGGCGCCAGATCCGCCAGCGCAAGCGGGCCGAGCGGGCGCTGAACGACCAGCTGGAATTCATGCGCGTGCT
CATCGACGGCACGCCTAACCCCATCTATGTGCGCGATAAGGAAGGCCGCATGCTGTTGTGCAATGACGCCTACCTCGACA
CCTTTGGCGTGACTGCCGATGCGGTACTGGGCAAGACCATTCCGGAAGCCAACGTGGTGGGCGACCCGGCGCTGGCCCGC
GAAATGCACGAGTTCCTGCTCACGCGCGTGGCCGCCGAGCGCGAGCCGCGCTTCGAGGACCGCGATGTCACGCTGCACGG
CCGCACCCGCCATGTCTACCAGTGGACGATTCCGTACGGCGACTCGCTGGGCGAACTCAAGGGCATCATCGGCGGCTGGA
TCGACATCACCGAACGCGCCGAGCTGCTGCGCGAGCTGCACGACGCCAAGGAAAGCGCCGACGCCGCCAACCGGGCCAAG
ACCACGTTCCTGGCAACGATGAGCCACGAGATCCGCACGCCGATGAACGCGATCATCGGCATGCTGGAGCTGGCGCTGCT
CCGTCCGACCGACCAGGAGCCGGATCGCCAGTCCATCCAGGTCGCGTACGACTCGGCCCGCAGCCTGCTGGAGCTGATAG
GCGACATCCTGGACATTGCGAAGATCGAGGCGGGAAAATTCGACCTGGCGCCGGTGCGCACGGCGCTGCGCGTCCTGCCC
GAAGGGGCGATCCGCGTCTTCGACGGATTGGCGCGCCAAAAAGGCATAGAGCTGGTATTGAAGACCGACATCGTGGGCGT
CGACGATGTATTGATAGACCCCTTGCGCATGAAGCAAGTGCTCTCGAACCTGGTGGGCAACGCCATCAAGTTCACCACCG
AAGGCCAGGTTGTCCTTGCCGTGACCGCACGCCCCGACGGCGACGCCGCGCACGTGCAGTTCAGCGTGAGCGACACCGGC
TGCGGCATCAGCGAGGCCGACCAACGGCAGCTGTTCAAACCGTTCTCGCAAGTGGGTGGCAGCGCCGAGGCCGGGCCGGC
GCCGGGCACCGGCCTGGGCCTGTCCATCAGCCGGCGCCTCGTCGAATTGATGGGGGGAACGCTGGTCATGCGCAGCGCGC
CAGGGGTGGGCACAACGGTTTCGGTGGACCTGAGGCTGACCATGGTCGAAAAATCCGTGCAGGCCGCGCCGCCCGCTGCG
GCCACTGCGGCCACGCCGTCCAAGCCGCAGGTATCGCTGCGCGTGCTGGTCGTCGATGACCACAAACCCAACCTGATGCT
GCTGCGCCAGCAGCTGGACTACCTGGGCCAGCGTGTCATCGCCGCCGACTCCGGCGAAGCCGCCCTGGCCCTGTGGCGCG
AGCATGCGTTCGACGTCGTGATCACCGATTGCAACATGCCCGGTATCAGCGGCTACGAATtGGCGCGCCGCATACGCGCC
GCCGAGGCCGCGCCCGGTTACGGACGTACGCGGTGCATTCTGTTCGGCTTCACGGCTTCGGCGCAGATGGACGAAGCGCA
GCGCTGCCGCGCCGCCGGCATGGACGACTGCCTGTTCAAGCCGATCGGCGTGGACGCCTTGCGGCAACGCTTGAACGAAG
CCGTGGCACGGGCCGCGCTCCCCACGCCCCCCTCGCCCCAGGCTGCCGCGCCGGCCACGGACGACGCCACCCCGACGGCG
TTCTCGGCCGAGTCGATTCTTGCCTTGACGCAGAACGATGAGGCGCTGATCCGGCAATTGCTCGAAGAAGTGATTCGCAC
CAACCGGGCGGACGTCGACCAATTGCAAAAGCTGCACCAGCAGGCCGATTGGCCGAAGGTCTCGGACATGGCGCACAGGC
TGGCCGGCGGCGCGCGCGTGGTCGATGCCAAGGCCATGATAGACACTGTGCTGGCGCTGGAGAAAAAAGCGCAAGGCCAG
GCTGGCCCCTCACCCGAAATCGACGGCCTGGTACGTACGCTTGCGGCGCAGTCCGCCGCGCTGGAGACGCAACTGCGCGC
CTGGCTGGAGCAACGGCCGCATCAAGATCAGCCCTGA
>Bp_B0782.seq
ATGCCCGCCCCGCACCGCCTGTACCCCCGCAGTCTGATCTGCCTGGCTCAGGCGCTATTGGCATGGGCTTTGCTGGCATG
GGCGCCCGCGCAGGCAAGCCAGGAGCTGACCCTGGTCGGCAAGGCTGCCGTTCCCGACGTCGAGGTCGCGCTCGACGGCG
ACGACTGGCGTTGGCTGGCCCGCAAGCGGGTACTGACGCTGGGTGTGTACGCACCGGACATTCCTCCGTTCGACGTCACC
TATGGCGAACGCTACGAAGGCCTGACGGCCGACTACATGGCGATCATCGCGCACAACCTGGGGATGCAGGCGAAAGTGCT
GCGATACCCCACGCGCGAACAAGCCCTCAGCGCGCTGGAAAGCGGGCAGATCGACCTCATCGGCACCGTCAATGGCACGG
ACGGCCGGCAACAGAGCCTGCGTCTGAGCGTTCCCTACGCCGCCGACCACCCGGTGATCGTCATGCCCATCGGCGCACGC
CACGTTCCAGCCTCGAACCTGGCCGGCCAGCGGCTGGCGGTCGACATCAACTACCTGCCCAAGGAAACGCTCGCACGGGC
CTACCCGCAGGCTACGCTGCATTACTTCCCCTCATCCGAGCAGGCGCTGGCCGCGGTGGCCTATGGGCAGGCCGACGTAT
TCATCGGCGATGCCCTGACCACCTCGCACCTCGTATCGCAAAGCTATTTCAATGACGTTCGCGTAGTCGCCCCGGCCCAT
ATCGCGACGGGCGGAGAATCCTTCGGCGTGCGCGCCGACAACACCCGCCTGCTGCGGGTGGTCAACGCCGTACTCGAAGC
CATTCCGCCTTCCGAACACCGCAGCCTGATCTACCGCTGGGGACTGGGCAGCAGCATTTCGCTCGATTTCGCGCACCCCG
CGTATTCCGCGCGCGAGCAGCAATGGATGGCAGACCACCCCGTCGTCAAGGTGGCGGTCCTGAATCTGTTCGCGCCCTTC
ACCCTGTTCCGCACCGACGAACAGTTCGGCGGGATCAGCGCCGCCGTGCTGCAGCTGCTGCAATTGCGCACCGGCCTGGA
CTTCGAGATCATCGGCGTCGACACGGTCGAGGAACTGATAGCCAAGCTGCGTTCGGGCGAAGCCGACATGGCCGGCGCCC
TGTTCGTCAACAGCGCGCGGGAGTCCTTCCTCAGTTTCAGCCGGCCGTATGTGCGCAATGGCATGGTGATCGTCACGCGC
CAGGACCCCGACGCGCCCGTCGACGCCGATCATCTGGACGGCCGCACGGTCGCGTTGGTGCGCAACAGCGCCGCCATTCC
CCTGCTGCAGCGGCGCTATCCCCAGGCGAAGGTGGTGACCGCCGACAACCCGAGCGAGGCGATGCTGATGGTGGCCAATG
GACAGGCCGACGCCGTCGTGCAGACGCAGATCAGCGCCAGCTATTACGTCAACCGCTACTTCGCCGGCAAGCTGCGCATC
GCCTCGGCGCTGGACCTGCCCCCGGCCGAGATCGCGCTGGCGACGACGCGCGGCCAGACCGAACTGATGTCCATCCTGAA
CAAGGCGCTCTACAGCATTTCGAACGACGAGCTCGCCTCCATCATCAGCCGCTGGCGCGGCAGCGACGGCGATCCGCGCA
CCTGGTACGCCTACCGCAACGAGATCTACCTGCTGATCGGGCTGGGCCTGTTGTCGGCCCTGCTGTTCCTGAGCTGGATC
GTCTACCTGCGGCGCCAGATCCGCCAGCGCAAGCGGGCCGAGCGGGCGCTGAACGACCAGCTGGAATTCATGCGCGTGCT
CATCGACGGCACGCCTAACCCCATCTATGTGCGCGATAAGGAAGGCCGCATGCTGTTGTGCAATGACGCCTACCTCGACA
CCTTTGGCGTGACTGCCGATGCGGTACTGGGCAAGACCATTCCGGAAGCCAACGTGGTGGGCGACCCGGCGCTGGCCCGC
GAAATGCACGAGTTCCTGCTCACGCGCGTGGCCGCCGAGCGCGAGCCGCGCTTCGAGGACCGCGATGTCACGCTGCACGG
CCGCACCCGCCATGTCTACCAGTGGACGATTCCGTACGGCGACTCGCTGGGCGAACTCAAGGGCATCATCGGCGGCTGGA
TCGACATCACCGAACGCGCCGAGCTGCTGCGCGAGCTGCACGACGCCAAGGAAAGCGCCGACGCCGCCAACCGGGCCAAG
ACCACGTTCCTGGCAACGATGAGCCACGAGATCCGCACGCCGATGAACGCGATCATCGGCATGCTGGAGCTGGCGCTGCT
CCGTCCGACCGACCAGGAGCCGGATCGCCAGTCCATCCAGGTCGCGTACGACTCGGCCCGCAGCCTGCTGGAGCTGATAG
GCGACATCCTGGACATTGCGAAGATCGAGGCGGGAAAATTCGACCTGGCGCCGGTGCGCACGGCGCTGCGCGTCCTGCCC
GAAGGGGCGATCCGCGTCTTCGACGGATTGGCGCGCCAAAAAGGCATAGAGCTGGTATTGAAGACCGACATCGTGGGCGT
CGACGATGTATTGATAGACCCCTTGCGCATGAAGCAAGTGCTCTCGAACCTGGTGGGCAACGCCATCAAGTTCACCACCG
AAGGCCAGGTTGTCCTTGCCGTGACCGCACGCCCCGACGGCGACGCCGCGCACGTGCAGTTCAGCGTGAGCGACACCGGC
TGCGGCATCAGCGAGGCCGACCAACGGCAGCTGTTCAAACCGTTCTCGCAAGTGGGTGGCAGCGCCGAGGCCGGGCCGGC
GCCGGGCACCGGCCTGGGCCTGTCCATCAGCCGGCGCCTCGTCGAATTGATGGGGGGAACGCTGGTCATGCGCAGCGCGC
CAGGGGTGGGCACAACGGTTTCGGTGGACCTGAGGCTGACCATGGTCGAAAAATCCGTGCAGGCCGCGCCGCCCGCTGCG
GCCACTGCGGCCACGCCGTCCAAGCCGCAGGTATCGCTGCGCGTGCTGGTCGTCGATGACCACAAACCCAACCTGATGCT
GCTGCGCCAGCAGCTGGACTACCTGGGCCAGCGTGTCATCGCCGCCGACTCCGGCGAAGCCGCCCTGGCCCTGTGGCGCG
AGCATGCGTTCGACGTCGTGATCACCGATTGCAACATGCCCGGTATCAGCGGCTACGAATTGGCGCGCCGCATACGCGCC
GCCGAGGCCGCGCCCGGTTACGGACGTACGCGGTGCATTCTGTTCGGCTTCACGGCTTCGGCGCAGATGGACGAAGCGCA
GCGCTGCCGCGCCGCCGGCATGGACGACTGCCTGTTCAAGCCGATCGGCGTGGACGCCTTGCGGCAACGCTTGAACGAAG
CCGTGGCACGGGCCGCGCTCCCCACGCCCCCCTCGCCCCAGGCTGCCGCGCCGGCCACGGACGACGCCACCCCGACGGCG
TTCTCGGCCGAGTCGATTCTTGCCTTGACGCAGAACGATGAGGCGCTGATCCGGCAATTGCTCGAAGAAGTGATTCGCAC
CAACCGGGCGGACGTCGACCAATTGCAAAAGCTGCACCAGCAGGCCGATTGGCCGAAGGTCTCGGACATGGCGCACAGGC
TGGCCGGCGGCGCGCGCGTGGTCGATGCCAAGGCCATGATAGACACTGTGCTGGCGCTGGAGAAAAAAGCGCAAGGCCAG
GCTGGCCCCTCACCCGAAATCGACGGCCTGGTACGTACGCTTGCGGCGCAGTCCGCCGCGCTGGAGACGCAACTGCGCGC
CTGGCTGGAGCAACGGCCGCATCAAGATCAGCCCTGA
>Bp_B0939.seq
ATGCCCGCCCCGCACCGCCTGTACCCCCGCAGTCTGATCTGCCTGGCTCAGGCGCTATTGGCATGGGCTTTGCTGGCATG
GGCGCCCGCGCAGGCAAGCCAGGAGCTGACCCTGGTCGGCAAGGCTGCCGTTCCCGACGTCGAGGTCGCGCTCGACGGCG
ACGACTGGCGTTGGCTGGCCCGCAAGCGGGTACTGACGCTGGGTGTGTACGCACCGGACATTCCTCCGTTCGACGTCACC
TATGGCGAACGCTACGAAGGCCTGACGGCCGACTACATGGCGATCATCGCGCACAACCTGGGGATGCAGGCGAAAGTGCT
GCGATACCCCACGCGCGAACAAGCCCTCAGCGCGCTGGAAAGCGGGCAGATCGACCTCATCGGCACCGTCAATGGCACGG
ACGGCCGGCAACAGAGCCTGCGTCTGAGCGTTCCCTACGCCGCCGACCACCCGGTGATCGTCATGCCCATCGGCGCACGC
CACGTTCCAGCCTCGAACCTGGCCGGCCAGCGGCTGGCGGTCGACATCAACTACCTGCCCAAGGAAACGCTCGCACGGGC
CTACCCGCAGGCTACGCTGCATTACTTCCCCTCATCCGAGCAGGCGCTGGCCGCGGTGGCCTATGGGCAGGCCGACGTAT
TCATCGGCGATGCCCTGACCACCTCGCACCTCGTATCGCAAAGCTATTTCAATGACGTTCGCGTAGTCGCCCCGGCCCAT
ATCGCGACGGGCGGAGAATCCTTCGGCGTGCGCGCCGACAACACCCGCCTGCTGCGGGTGGTCAACGCCGTACTCGAAGC
CATTCCGCCTTCCGAACACCGCAGCCTGATCTACCGCTGGGGACTGGGCAGCAGCATTTCGCTCGATTTCGCGCACCCCG
CGTATTCCGCGCGCGAGCAGCAATGGATGGCAGACCACCCCGTCGTCAAGGTGGCGGTCCTGAATCTGTTCGCGCCCTTC
ACCCTGTTCCGCACCGACGAACAGTTCGGCGGGATCAGCGCCGCCGTGCTGCAGCTGCTGCAATTGCGCACCGGCCTGGA
CTTCGAGATCATCGGCGTCGACACGGTCGAGGAACTGATAGCCAAGCTGCGTTCGGGCGAAGCCGACATGGCCGGCGCCC
TGTTCGTCAACAGCGCGCGGGAGTCCTTCCTCAGTTTCAGCCGGCCGTATGTGCGCAATGGCATGGTGATCGTCACGCGC
CAGGACCCCGACGCGCCCGTCGACGCCGATCATCTGGACGGCCGCACGGTCGCGTTGGTGCGCAACAGCGCCGCCATTCC
CCTGCTGCAGCGGCGCTATCCCCAGGCGAAGGTGGTGACCGCCGACAACCCGAGCGAGGCGATGCTGATGGTGGCCAATG
GACAGGCCGACGCCGTCGTGCAGACGCAGATCAGCGCCAGCTATTACGTCAACCGCTACTTCGCCGGCAAGCTGCGCATC
GCCTCGGCGCTGGACCTGCCCCCGGCCGAGATCGCGCTGGCGACGACGCGCGGCCAGACCGAACTGATGTCCATCCTGAA
CAAGGCGCTCTACAGCATTTCGAACGACGAGCTCGCCTCCATCATCAGCCGCTGGCGCGGCAGCGACGGCGATCCGCGCA
CCTGGTACGCCTACCGCAACGAGATCTACCTGCTGATCGGGCTGGGCCTGTTGTCGGCCCTGCTGTTCCTGAGCTGGATC
GTCTACCTGCGGCGCCAGATCCGCCAGCGCAAGCGGGCCGAGCGGGCGCTGAACGACCAGCTGGAATTCATGCGCGTGCT
CATCGACGGCACGCCTAACCCCATCTATGTGCGCGATAAGGAAGGCCGCATGCTGTTGTGCAATGACGCCTACCTCGACA
CCTTTGGCGTGACTGCCGATGCGGTACTGGGCAAGACCATTCCGGAAGCCAACGTGGTGGGCGACCCGGCGCTGGCCCGC
GAAATGCACGAGTTCCTGCTCACGCGCGTGGCCGCCGAGCGCGAGCCGCGCTTCGAGGACCGCGATGTCACGCTGCACGG
CCGCACCCGCCATGTCTACCAGTGGACGATTCCGTACGGCGACTCGCTGGGCGAACTCAAGGGCATCATCGGCGGCTGGA
TCGACATCACCGAACGCGCCGAGCTGCTGCGCGAGCTGCACGACGCCAAGGAAAGCGCCGACGCCGCCAACCGGGCCAAG
ACCACGTTCCTGGCAACGATGAGCCACGAGATCCGCACGCCGATGaACGCGATCATCGGCATGCTGGAGCTGGCGCTGCT
CCGTCCGACCGACCAGGAGCCGGATCGCCAGTCCATCCAGGTCGCGTACGACTCGGCCCGCAGCCTGCTGGAGCTGATAG
GCGACATCCTGGACATTGCGAAGATCGAGGCGGGAAAATTCGACCTGGCGCCGGTGCGCACGGCGCTGCGCGTCCTGCCC
GAAGGGGCGATCCGCGTCTTCGACGGATTGGCGCGCCAAAAAGGCATAGAGCTGGTATTGAAGACCGACATCGTGGGCGT
CGACGATGTATTGATAGACCCCTTGCGCATGAAGCAAGTGCTCTCGAACCTGGTGGGCAACGCCATCAAGTTCACCACCG
AAGGCCAGGTTGTCCTTGCCGTGACCGCACGCCCCGACGGCGACGCCGCGCACGTGCAGTTCAGCGTGAGCGACACCGGC
TGCGGCATCAGCGAGGCCGACCAACGGCAGCTGTTCAAACCGTTCTCGCAAGTGGGTGGCAGCGCCGAGGCCGGGCCGGC
GCCGGGCACCGGCCTGGGCCTGTCCATCAGCCGGCGCCTCGTCGAATTGATGGGGGGAACGCTGGTCATGCGCAGCGCGC
CAGGGGTGGGCACAACGGTTTCGGTGGACCTGAGGCTGACCATGGTCGAAAAATCCGTGCAGGCCGCGCCGCCCGCTGCG
GCCACTGCGGCCACGCCGTCCAAGCCGCAGGTATCGCTGCGCGTGCTGGTCGTCGATGACCACAAACCCAACCTGATGCT
GCTGCGCCAGCAGCTGGACTACCTGGGCCAGCGTGTCATCGCCGCCGACTCCGGCGAAGCCGCCCTGGCCCTGTGGCGCG
AGCATGCGTTCGACGTCGTGATCACCGATTGCAACATGCCCGGTATCAGCGGCTACGAATtGGCGCGCCGCATACGCGCC
GCCGAGGCCGCGCCCGGTTACGGACGTACGCGGTGCATTCTGTTCGGCTTCACGGCTTCGGCGCAGATGGACGAAGCGCA
GCGCTGCCGCGCCGCCGGCATGGACGACTGCCTGTTCAAGCCGATCGGCGTGGACGCCTTGCGGCAACGCTTGAACGAAG
CCGTGGCACGGGCCGCGCTCCCCACGCCCCCCTCGCCCCAGGCTGCCGCGCCGGCCACGGACGACGCCACCCCGACGGCG
TTCTCGGCCGAGTCGATTCTTGCCTTGACGCAGAACGATGAGGCGCTGATCCGGCAATTGCTCGAAGAAGTGATTCGCAC
CAACCGGGCGGACGTCGACCAATTGCAAAAGCTGCACCAGCAGGCCGATTGGCCGAAGGTCTCGGACATGGCGCACAGGC
TGGCCGGCGGCGCGCGCGTGGTCGATGCCAAGGCCATGATAGACACTGTGCTGGCGCTGGAGAAAAAAGCGCAAGGCCAG
GCTGGCCCCTCACCCGAAATCGACGGCCTGGTACGTACGCTTGCGGCGCAGTCCGCCGCGCTGGAGACGCAACTGCGCGC
CTGGCTGGAGCAACGGCCGCATCAAGATCAGCCCTGA
>Bp_B1054.seq
ATGCCCGCCCCGCACCGCCTGTACCCCCGCAGTCTGATCTGCCTGGCTCAGGCGCTATTGGCATGGGCTTTGCTGGCATG
GGCGCCCGCGCAGGCAAGCCAGGAGCTGACCCTGGTCGGCAAGGCTGCCGTTCCCGACGTCGAGGTCGCGCTCGACGGCG
ACGACTGGCGTTGGCTGGCCCGCAAGCGGGTACTGACGCTGGGTGTGTACGCACCGGACATTCCTCCGTTCGACGTCACC
TATGGCGAACGCTACGAAGGCCTGACGGCCGACTACATGGCGATCATCGCGCACAACCTGGGGATGCAGGCGAAAGTGCT
GCGATACCCCACGCGCGAACAAGCCCTCAGCGCGCTGGAAAGCGGGCAGATCGACCTCATCGGCACCGTCAATGGCACGG
ACGGCCGGCAACAGAGCCTGCGTCTGAGCGTTCCCTACGCCGCCGACCACCCGGTGATCGTCATGCCCATCGGCGCACGC
CACGTTCCAGCCTCGAACCTGGCCGGCCAGCGGCTGGCGGTCGACATCAACTACCTGCCCAAGGAAACGCTCGCACGGGC
CTACCCGCAGGCTACGCTGCATTACTTCCCCTCATCCGAGCAGGCGCTGGCCGCGGTGGCCTATGGGCAGGCCGACGTAT
TCATCGGCGATGCCCTGACCACCTCGCACCTCGTATCGCAAAGCTATTTCAATGACGTTCGCGTAGTCGCCCCGGCCCAT
ATCGCGACGGGCGGAGAATCCTTCGGCGTGCGCGCCGACAACACCCGCCTGCTGCGGGTGGTCAACGCCGTACTCGAAGC
CATTCCGCCTTCCGAACACCGCAGCCTGATCTACCGCTGGGGACTGGGCAGCAGCATTTCGCTCGATTTCGCGCACCCCG
CGTATTCCGCGCGCGAGCAGCAATGGATGGCAGACCACCCCGTCGTCAAGGTGGCGGTCCTGAATCTGTTCGCGCCCTTC
ACCCTGTTCCGCACCGACGAACAGTTCGGCGGGATCAGCGCCGCCGTGCTGCAGCTGCTGCAATTGCGCACCGGCCTGGA
CTTCGAGATCATCGGCGTCGACACGGTCGAGGAACTGATAGCCAAGCTGCGTTCGGGCGAAGCCGACATGGCCGGCGCCC
TGTTCGTCAACAGCGCGCGGGAGTCCTTCCTCAGTTTCAGCCGGCCGTATGTGCGCAATGGCATGGTGATCGTCACGCGC
CAGGACCCCGACGCGCCCGTCGACGCCGATCATCTGGACGGCCGCACGGTCGCGTTGGTGCGCAACAGCGCCGCCATTCC
CCTGCTGCAGCGGCGCTATCCCCAGGCGAAGGTGGTGACCGCCGACAACCCGAGCGAGGCGATGCTGATGGTGGCCAATG
GACAGGCCGACGCCGTCGTGCAGACGCAGATCAGCGCCAGCTATTACGTCAACCGCTACTTCGCCGGCAAGCTGCGCATC
GCCTCGGCGCTGGACCTGCCCCCGGCCGAGATCGCGCTGGCGACGACGCGCGGCCAGACCGAACTGATGTCCATCCTGAA
CAAGGCGCTCTACAGCATTTCGAACGACGAGCTCGCCTCCATCATCAGCCGCTGGCGCGGCAGCGACGGCGATCCGCGCA
CCTGGTACGCCTACCGCAACGAGATCTACCTGCTGATCGGGCTGGGCCTGTTGTCGGCCCTGCTGTTCCTGAGCTGGATC
GTCTACCTGCGGCGCCAGATCCGCCAGCGCAAGCGGGCCGAGCGGGCGCTGAACGACCAGCTGGAATTCATGCGCGTGCT
CATCGACGGCACGCCTAACCCCATCTATGTGCGCGATAAGGAAGGCCGCATGCTGTTGTGCAATGACGCCTACCTCGACA
CCTTTGGCGTGACTGCCGATGCGGTACTGGGCAAGACCATTCCGGAAGCCAACGTGGTGGGCGACCCGGCGCTGGCCCGC
GAAATGCACGAGTTCCTGCTCACGCGCGTGGCCGCCGAGCGCGAGCCGCGCTTCGAGGACCGCGATGTCACGCTGCACGG
CCGCACCCGCCATGTCTACCAGTGGACGATTCCGTACGGCGACTCGCTGGGCGAACTCAAGGGCATCATCGGCGGCTGGA
TCGACATCACCGAACGCGCCGAGCTGCTGCGCGAGCTGCACGACGCCAAGGAAAGCGCCGACGCCGCCAACCGGGCCAAG
ACCACGTTCCTGGCAACGATGAGCCACGAGATCCGCACGCCGATGAACGCGATCATCGGCATGCTGGAGCTGGCGCTGCT
CCGTCCGACCGACCAGGAGCCGGATCGCCAGTCCATCCAGGTCGCGTACGACTCGGCCCGCAGCCTGCTGGAGCTGATAG
GCGACATCCTGGACATTGCGAAGATCGAGGCGGGAAAATTCGACCTGGCGCCGGTGCGCACGGCGCTGCGCGTCCTGCCC
GAAGGGGCGATCCGCGTCTTCGACGGATTGGCGCGCCAAAAAGGCATAGAGCTGGTATTGAAGACCGACATCGTGGGCGT
CGACGATGTATTGATAGACCCCTTGCGCATGAAGCAAGTGCTCTCGAACCTGGTGGGCAACGCCATCAAGTTCACCACCG
AAGGCCAGGTTGTCCTTGCCGTGACCGCACGCCCCGACGGCGACGCCGCGCACGTGCAGTTCAGCGTGAGCGACACCGGC
TGCGGCATCAGCGAGGCCGACCAACGGCAGCTGTTCAAACCGTTCTCGCAAGTGGGTGGCAGCGCCGAGGCCGGGCCGGC
GCCGGGCACCGGCCTGGGCCTGTCCATCAGCCGGCGCCTCGTCGAATTGATGGGGGGAACGCTGGTCATGCGCAGCGCGC
CAGGGGTGGGCACAACGGTTTCGGTGGACCTGAGGCTGACCATGGTCGAAAAATCCGTGCAGGCCGCGCCGCCCGCTGCG
GCCACTGCGGCCACGCCGTCCAAGCCGCAGGTATCGCTGCGCGTGCTGGTCGTCGATGACCACAAACCCAACCTGATGCT
GCTGCGCCAGCAGCTGGACTACCTGGGCCAGCGTGTCATCGCCGCCGACTCCGGCGAAGCCGCCCTGGCCCTGTGGCGCG
AGCATGCGTTCGACGTCGTGATCACCGATTgCAACATGCCCGGTATCAGCGGCTACGAATtGGCGCGCCGCATACGCGCC
GCCGAGGCCGCGCCCGGTTACGGACGTACGCGGTGCATTCTGTTCGGCTTCACGGCTTCGGCGCAGATGGACGAAGCGCA
GCGCTGCCGCGCCGCCGGCATGGACGACTGCCTGTTCAAGCCGATCGGCGTGGACGCCTTGCGGCAACGCTTGAACGAAG
CCGTGGCACGGGCCGCGCTCCCCACGCCCCCCTCGCCCCAGGCTGCCGCGCCGGCCACGGACGACGCCACCCCGACGGCG
TTCTCGGCCGAGTCGATTCTTGCCTTGACGCAGAACGATGAGGCGCTGATCCGGCAATTGCTCGAAGAAGTGATTCGCAC
CAACCGGGCGGACGTCGACCAATTGCAAAAGCTGCACCAGCAGGCCGATTGGCCGAAGGTCTCGGACATGGCGCACAGGC
TGGCCGGCGGCGCGCGCGTGGTCGATGCCAAGGCCATGATAGACACTGTGCTGGCGCTGGAGAAAAAAGCGCAAGGCCAG
GCTGGCCCCTCACCCGAAATCGACGGCCTGGTACGTACGCTTGCGGCGCAGTCCGCCGCGCTGGAGACGCAACTGCGCGC
CTGGCTGGAGCAACGGCCGCATCAAGATCAGCCCTGA
>Bp_B1055.seq
ATGCCCGCCCCGCACCGCCTGTACCCCCGCAGTCTGATCTGCCTGGCTCAGGCGCTATTGGCATGGGCTTTGCTGGCATG
GGCGCCCGCGCAGGCAAGCCAGGAGCTGACCCTGGTCGGCAAGGCTGCCGTTCCCGACGTCGAGGTCGCGCTCGACGGCG
ACGACTGGCGTTGGCTGGCCCGCAAGCGGGTACTGACGCTGGGTGTGTACGCACCGGACATTCCTCCGTTCGACGTCACC
TATGGCGAACGCTACGAAGGCCTGACGGCCGACTACATGGCGATCATCGCGCACAACCTGGGGATGCAGGCGAAAGTGCT
GCGATACCCCACGCGCGAACAAGCCCTCAGCGCGCTGGAAAGCGGGCAGATCGACCTCATCGGCACCGTCAATGGCACGG
ACGGCCGGCAACAGAGCCTGCGTCTGAGCGTTCCCTACGCCGCCGACCACCCGGTGATCGTCATGCCCATCGGCGCACGC
CACGTTCCAGCCTCGAACCTGGCCGGCCAGCGGCTGGCGGTCGACATCAACTACCTGCCCAAGGAAACGCTCGCACGGGC
CTACCCGCAGGCTACGCTGCATTACTTCCCCTCATCCGAGCAGGCGCTGGCCGCGGTGGCCTATGGGCAGGCCGACGTAT
TCATCGGCGATGCCCTGACCACCTCGCACCTCGTATCGCAAAGCTATTTCAATGACGTTCGCGTAGTCGCCCCGGCCCAT
ATCGCGACGGGCGGAGAATCCTTCGGCGTGCGCGCCGACAACACCCGCCTGCTGCGGGTGGTCAACGCCGTACTCGAAGC
CATTCCGCCTTCCGAACACCGCAGCCTGATCTACCGCTGGGGACTGGGCAGCAGCATTTCGCTCGATTTCGCGCACCCCG
CGTATTCCGCGCGCGAGCAGCAATGGATGGCAGACCACCCCGTCGTCAAGGTGGCGGTCCTGAATCTGTTCGCGCCCTTC
ACCCTGTTCCGCACCGACGAACAGTTCGGCGGGATCAGCGCCGCCGTGCTGCAGCTGCTGCAATTGCGCACCGGCCTGGA
CTTCGAGATCATCGGCGTCGACACGGTCGAGGAACTGATAGCCAAGCTGCGTTCGGGCGAAGCCGACATGGCCGGCGCCC
TGTTCGTCAACAGCGCGCGGGAGTCCTTCCTCAGTTTCAGCCGGCCGTATGTGCGCAATGGCATGGTGATCGTCACGCGC
CAGGACCCCGACGCGCCCGTCGACGCCGATCATCTGGACGGCCGCACGGTCGCGTTGGTGCGCAACAGCGCCGCCATTCC
CCTGCTGCAGCGGCGCTATCCCCAGGCGAAGGTGGTGACCGCCGACAACCCGAGCGAGGCGATGCTGATGGTGGCCAATG
GACAGGCCGACGCCGTCGTGCAGACGCAGATCAGCGCCAGCTATTACGTCAACCGCTACTTCGCCGGCAAGCTGCGCATC
GCCTCGGCGCTGGACCTGCCCCCGGCCGAGATCGCGCTGGCGACGACGCGCGGCCAGACCGAACTGATGTCCATCCTGAA
CAAGGCGCTCTACAGCATTTCGAACGACGAGCTCGCCTCCATCATCAGCCGCTGGCGCGGCAGCGACGGCGATCCGCGCA
CCTGGTACGCCTACCGCAACGAGATCTACCTGCTGATCGGGCTGGGCCTGTTGTCGGCCCTGCTGTTCCTGAGCTGGATC
GTCTACCTGCGGCGCCAGATCCGCCAGCGCAAGCGGGCCGAGCGGGCGCTGAACGACCAGCTGGAATTCATGCGCGTGCT
CATCGACGGCACGCCTAACCCCATCTATGTGCGCGATAAGGAAGGCCGCATGCTGTTGTGCAATGACGCCTACCTCGACA
CCTTTGGCGTGACTGCCGATGCGGTACTGGGCAAGACCATTCCGGAAGCCAACGTGGTGGGCGACCCGGCGCTGGCCCGC
GAAATGCACGAGTTCCTGCTCACGCGCGTGGCCGCCGAGCGCGAGCCGCGCTTCGAGGACCGCGATGTCACGCTGCACGG
CCGCACCCGCCATGTCTACCAGTGGACGATTCCGTACGGCGACTCGCTGGGCGAACTCAAGGGCATCATCGGCGGCTGGA
TCGACATCACCGAACGCGCCGAGCTGCTGCGCGAGCTGCACGACGCCAAGGAAAGCGCCGACGCCGCCAACCGGGCCAAG
ACCACGTTCCTGGCAACGATGAGCCACGAGATCCGCACGCCGATGaACGCGATCATCGGCATGCTGGAGCTGGCGCTGCT
CCGTCCGACCGACCAGGAGCCGGATCGCCAGTCCATCCAGGTCGCGTACGACTCGGCCCGCAGCCTGCTGGAGCTGATAG
GCGACATCCTGGACATTGCGAAGATCGAGGCGGGAAAATTCGACCTGGCGCCGGTGCGCACGGCGCTGCGCGTCCTGCCC
GAAGGGGCGATCCGCGTCTTCGACGGATTGGCGCGCCAAAAAGGCATAGAGCTGGTATTGAAGACCGACATCGTGGGCGT
CGACGATGTATTGATAGACCCCTTGCGCATGAAGCAAGTGCTCTCGAACCTGGTGGGCAACGCCATCAAGTTCACCACCG
AAGGCCAGGTTGTCCTTGCCGTGACCGCACGCCCCGACGGCGACGCCGCGCACGTGCAGTTCAGCGTGAGCGACACCGGC
TGCGGCATCAGCGAGGCCGACCAACGGCAGCTGTTCAAACCGTTCTCGCAAGTGGGTGGCAGCGCCGAGGCCGGGCCGGC
GCCGGGCACCGGCCTGGGCCTGTCCATCAGCCGGCGCCTCGTCGAATTGATGGGGGGAACGCTGGTCATGCGCAGCGCGC
CAGGGGTGGGCACAACGGTTTCGGTGGACCTGAGGCTGACCATGGTCGAAAAATCCGTGCAGGCCGCGCCGCCCGCTGCG
GCCACTGCGGCCACGCCGTCCAAGCCGCAGGTATCGCTGCGCGTGCTGGTCGTCGATGACCACAAACCCAACCTGATGCT
GCTGCGCCAGCAGCTGGACTACCTGGGCCAGCGTGTCATCGCCGCCGACTCCGGCGAAGCCGCCCTGGCCCTGTGGCGCG
AGCATGCGTTCGACGTCgTGATCACCGATTGCAACATGCCCGGTATCAGCGGCTACGAATTGGCGCGCCGCATACGCGCC
GCCGAGGCCGCGCCCGGTTACGGACGTACGCGGTGCATTCTGTTCGGCTTCACGGCTTCGGCGCAGATGGACGAAGCGCA
GCGCTGCCGCGCCGCCGGCATGGACGACTGCCTGTTCAAGCCGATCGGCGTGGACGCCTTGCGGCAACGCTTGAACGAAG
CCGTGGCACGGGCCGCGCTCCCCACGCCCCCCTCGCCCCAGGCTGCCGCGCCGGCCACGGACGACGCCACCCCGACGGCG
TTCTCGGCCGAGTCGATTCTTGCCTTGACGCAGAACGATGAGGCGCTGATCCGGCAATTGCTCGAAGAAGTGATTCGCAC
CAACCGGGCGGACGTCGACCAATTGCAAAAGCTGCACCAGCAGGCCGATTGGCCGAAGGTCTCGGACATGGCGCACAGGC
TGGCCGGCGGCGCGCGCGTGGTCGATGCCAAGGCCATGATAGACACTGTGCTGGCGCTGGAGAAAAAAGCGCAAGGCCAG
GCTGGCCCCTCACCCGAAATCGACGGCCTGGTACGTACGCTTGCGGCGCAGTCCGCCGCGCTGGAGACGCAACTGCGCGC
CTGGCTGGAGCAACGGCCGCATCAAGATCAGCCCTGA
>Bp_B1063.seq
ATGCCCGCCCCGCACCGCCTGTACCCCCGCAGTCTGATCTGCCTGGCTCAGGCGCTATTGGCATGGGCTTTGCTGGCATG
GGCGCCCGCGCAGGCAAGCCAGGAGCTGACCCTGGTCGGCAAGGCTGCCGTTCCCGACGTCGAGGTCGCGCTCGACGGCG
ACGACTGGCGTTGGCTGGCCCGCAAGCGGGTACTGACGCTGGGTGTGTACGCACCGGACATTCCTCCGTTCGACGTCACC
TATGGCGAACGCTACGAAGGCCTGACGGCCGACTACATGGCGATCATCGCGCACAACCTGGGGATGCAGGCGAAAGTGCT
GCGATACCCCACGCGCGAACAAGCCCTCAGCGCGCTGGAAAGCGGGCAGATCGACCTCATCGGCACCGTCAATGGCACGG
ACGGCCGGCAACAGAGCCTGCGTCTGAGCGTTCCCTACGCCGCCGACCACCCGGTGATCGTCATGCCCATCGGCGCACGC
CACGTTCCAGCCTCGAACCTGGCCGGCCAGCGGCTGGCGGTCGACATCAACTACCTGCCCAAGGAAACGCTCGCACGGGC
CTACCCGCAGGCTACGCTGCATTACTTCCCCTCATCCGAGCAGGCGCTGGCCGCGGTGGCCTATGGGCAGGCCGACGTAT
TCATCGGCGATGCCCTGACCACCTCGCACCTCGTATCGCAAaGCTATTTCAATGACGTTCGCGTAGTCGCCCCGGCCCAT
ATCGCGACGGGCGGAGAATCCTTCGGCGTGCGCGCCGACAACACCCGCCTGCTGCGGGTGGTCAACGCCGTACTCGAAGC
CATTCCGCCTTCCGAACACCGCAGCCTGATCTACCGCTGGGGACTGGGCAGCAGCATTTCGCTCGATTTCGCGCACCCCG
CGTATTCCGCGCGCGAGCAGCAATGGATGGCAGACCACCCCGTCGTCAAGGTGGCGGTCCTGAATCTGTTCGCGCCCTTC
ACCCTGTTCCGCACCGACGAACAGTTCGGCGGGATCAGCGCCGCCGTGCTGCAGCTGCTGCAATTGCGCACCGGCCTGGA
CTTCGAGATCATCGGCGTCGACACGGTCGAGGAACTGATAGCCAAGCTGCGTTCGGGCGAAGCCGACATGGCCGGCGCCC
TGTTCGTCAACAGCGCGCGGGAGTCCTTCCTCAGTTTCAGCCGGCCGTATGTGCGCAATGGCATGGTGATCGTCACGCGC
CAGGACCCCGACGCGCCCGTCGACGCCGATCATCTGGACGGCCGCACGGTCGCGTTGGTGCGCAACAGCGCCGCCATTCC
CCTGCTGCAGCGGCGCTATCCCCAGGCGAAGGTGGTGACCGCCGACAACCCGAGCGAGGCGATGCTGATGGTGGCCAATG
GACAGGCCGACGCCGTCGTGCAGACGCAGATCAGCGCCAGCTATTACGTCAACCGCTACTTCGCCGGCAAGCTGCGCATC
GCCTCGGCGCTGGACCTGCCCCCGGCCGAGATCGCGCTGGCGACGACGCGCGGCCAGACCGAACTGATGTCCATCCTGAA
CAAGGCGCTCTACAGCATTTCGAACGACGAGCTCGCCTCCATCATCAGCCGCTGGCGCGGCAGCGACGGCGATCCGCGCA
CCTGGTACGCCTACCGCAACGAGATCTACCTGCTGATCGGGCTGGGCCTGTTGTCGGCCCTGCTGTTCCTGAGCTGGATC
GTCTACCTGCGGCGCCAGATCCGCCAGCGCAAGCGGGCCGAGCGGGCGCTGAACGACCAGCTGGAATTCATGCGCGTGCT
CATCGACGGCACGCCTAACCCCATCTATGTGCGCGATAAGGAAGGCCGCATGCTGTTGTGCAATGACGCCTACCTCGACA
CCTTTGGCGTGACTGCCGATGCGGTACTGGGCAAGACCATTCCGGAAGCCAACGTGGTGGGCGACCCGGCGCTGGCCCGC
GAAATGCACGAGTTCCTGCTCACGCGCGTGGCCGCCGAGCGCGAGCCGCGCTTCGAGGACCGCGATGTCACGCTGCACGG
CCGCACCCGCCATGTCTACCAGTGGACGATTCCGTACGGCGACTCGCTGGGCGAACTCAAGGGCATCATCGGCGGCTGGA
TCGACATCACCGAACGCGCCGAGCTGCTGCGCGAGCTGCACGACGCCAAGGAAAGCGCCGACGCCGCCAACCGGGCCAAG
ACCACGTTCCTGGCAACGATGAGCCACGAGATCCGCACGCCGATGAACGCGATCATCGGCATGCTGGAGCTGGCGCTGCT
CCGTCCGACCGACCAGGAGCCGGATCGCCAGTCCATCCAGGTCGCGTACGACTCGGCCCGCAGCCTGCTGGAGCTGATAG
GCGACATCCTGGACATTGCGAAGATCGAGGCGGGAAAATTCGACCTGGCGCCGGTGCGCACGGCGCTGCGCGTCCTGCCC
GAAGGGGCGATCCGCGTCTTCGACGGATTGGCGCGCCAAAAAGGCATAGAGCTGGTATTGAAGACCGACATCGTGGGCGT
CGACGATGTATTGATAGACCCCTTGCGCATGAAGCAAGTGCTCTCGAACCTGGTGGGCAACGCCATCAAGTTCACCACCG
AAGGCCAGGTTGTCCTTGCCGTGACCGCACGCCCCGACGGCGACGCCGCGCACGTGCAGTTCAGCGTGAGCGACACCGGC
TGCGGCATCAGCGAGGCCGACCAACGGCAGCTGTTCAAACCGTTCTCGCAAGTGGGTGGCAGCGCCGAGGCCGGGCCGGC
GCCGGGCACCGGCCTGGGCCTGTCCATCAGCCGGCGCCTCGTCGAATTGATGGGGGGAACGCTGGTCATGCGCAGCGCGC
CAGGGGTGGGCACAACGGTTTCGGTGGACCTGAGGCTGACCATGGTCGAAAAATCCGTGCAGGCCGCGCCGCCCGCTGCG
GCCACTGCGGCCACGCCGTCCAAGCCGCAGGTATCGCTGCGCGTGCTGGTCGTCGATGACCACAAACCCAACCTGATGCT
GCTGCGCCAGCAGCTGGACTACCTGGGCCAGCGTGTCATCGCCGCCGACTCCGGCGAAGCCGCCCTGGCCCTGTGGCGCG
AGCATGCGTTCGACGTCGTGATCACCGATTgCAACATGCCCGGTATCAGCGGCTACGAATtGGCGCGCCGCATACGCGCC
GCCGAGGCCGCGCCCGGTTACGGACGTACGCGGTGCATTCTGTTCGGCTTCACGGCTTCGGCGCAGATGGACGAAGCGCA
GCGCTGCCGCGCCGCCGGCATGGACGACTGCCTGTTCAAGCCGATCGGCGTGGACGCCTTGCGGCAACGCTTGAACGAAG
CCGTGGCACGGGCCGCGCTCCCCACGCCCCCCTCGCCCCAGGCTGCCGCGCCGGCCACGGACGACGCCACCCCGACGGCG
TTCTCGGCCGAGTCGATTCTTGCCTTGACGCAGAACGATGAGGCGCTGATCCGGCAATTGCTCGAAGAAGTGATTCGCAC
CAACCGGGCGGACGTCGACCAATTGCAAAAGCTGCACCAGCAGGCCGATTGGCCGAAGGTCTCGGACATGGCGCACAGGC
TGGCCGGCGGCGCGCGCGTGGTCGATGCCAAGGCCATGATAGACACTGTGCTGGCGCTGGAGAAAAAAGCGCAAGGCCAG
GCTGGCCCCTCACCCGAAATCGACGGCCTGGTACGTACGCTTGCGGCGCAGTCCGCCGCGCTGGAGACGCAACTGCGCGC
CTGGCTGGAGCAACGGCCGCATCAAGATCAGCCCTGA
>Bp_B1121.seq
ATGCCCGCCCCGCACCGCCTGTACCCCCGCAGTCTGATCTGCCTGGCTCAGGCGCTATTGGCATGGGCTTTGCTGGCATG
GGCGCCCGCGCAGGCAAGCCAGGAGCTGACCCTGGTCGGCAAGGCTGCCGTTCCCGACGTCGAGGTCGCGCTCGACGGCG
ACGACTGGCGTTGGCTGGCCCGCAAGCGGGTACTGACGCTGGGTGTGTACGCACCGGACATTCCTCCGTTCGACGTCACC
TATGGCGAACGCTACGAAGGCCTGACGGCCGACTACATGGCGATCATCGCGCACAACCTGGGGATGCAGGCGAAAGTGCT
GCGATACCCCACGCGCGAACAAGCCCTCAGCGCGCTGGAAAGCGGGCAGACCGACCTCATCGGCACCGTCAATGGCACGG
ACGGCCGGCAACAGAGCCTGCGTCTGAGCGTTCCCTACGCCGCCGACCACCCGGTGATCGTCATGCCCATCGGCGCACGC
CACGTTCCAGCCTCGAACCTGGCCGGCCAGCGGCTGGCGGTCGACATCAACTACCTGCCCAAGGAAACGCTCGCACGGGC
CTACCCGCAGGCTACGCTGCATTACTTCCCCTCATCCGAGCAGGCGCTGGCCGCGGTGGCCTATGGGCAGGCCGACGTAT
TCATCGGCGATGCCCTGACCACCTCGCACCTCGTATCGCAAAGCTATTTCAATGACGTTCGCGTAGTCGCCCCGGCCCAT
ATCGCGACGGGCGGAGAATCCTTCGGCGTGCGCGCCGACAACACCCGCCTGCTGCGGGTGGTCAACGCCGTACTCGAAGC
CATTCCGCCTTCCGAACACCGCAGCCTGATCTACCGCTGGGGACTGGGCAGCAGCATTTCGCTCGATTTCGCGCACCCCG
CGTATTCCGCGCGCGAGCAGCAATGGATGGCAGACCACCCCGTCGTCAAGGTGGCGGTCCTGAATCTGTTCGCGCCCTTC
ACCCTGTTCCGCACCGACGAACAGTTCGGCGGGATCAGCGCCGCCGTGCTGCAGCTGCTGCAATTGCGCACCGGCCTGGA
CTTCGAGATCATCGGCGTCGACACGGTCGAGGAACTGATAGCCAAGCTGCGTTCGGGCGAAGCCGACATGGCCGGCGCCC
TGTTCGTCAACAGCGCGCGGGAGTCCTTCCTCAGTTTCAGCCGGCCGTATGTGCGCAATGGCATGGTGATCGTCACGCGC
CAGGACCCCGACGCGCCCGTCGACGCCGATCATCTGGACGGCCGCACGGTCGCGTTGGTGCGCAACAGCGCCGCCATTCC
CCTGCTGCAGCGGCGCTATCCCCAGGCGAAGGTGGTGACCGCCGACAACCCGAGCGAGGCGATGCTGATGGTGGCCAATG
GACAGGCCGACGCCGTCGTGCAGACGCAGATCAGCGCCAGCTATTACGTCAACCGCTACTTCGCCGGCAAGCTGCGCATC
GCCTCGGCGCTGGACCTGCCCCCGGCCGAGATCGCGCTGGCGACGACGCGCGGCCAGACCGAACTGATGTCCATCCTGAA
CAAGGCGCTCTACAGCATTTCGAACGACGAGCTCGCCTCCATCATCAGCCGCTGGCGCGGCAGCGACGGCGATCCGCGCA
CCTGGTACGCCTACCGCAACGAGATCTACCTGCTGATCGGGCTGGGCCTGTTGTCGGCCCTGCTGTTCCTGAGCTGGATC
GTCTACCTGCGGCGCCAGATCCGCCAGCGCAAGCGGGCCGAGCGGGCGCTGAACGACCAGCTGGAATTCATGCGCGTGCT
CATCGACGGCACGCCTAACCCCATCTATGTGCGCGATAAGGAAGGCCGCATGCTGTTGTGCAATGACGCCTACCTCGACA
CCTTTGGCGTGACTGCCGATGCGGTACTGGGCAAGACCATTCCGGAAGCCAACGTGGTGGGCGACCCGGCGCTGGCCCGC
GAAATGCACGAGTTCCTGCTCACGCGCGTGGCCGCCGAGCGCGAGCCGCGCTTCGAGGACCGCGATGTCACGCTGCACGG
CCGCACCCGCCATGTCTACCAGTGGACGATTCCGTACGGCGACTCGCTGGGCGAACTCAAGGGCATCATCGGCGGCTGGA
TCGACATCACCGAACGCGCCGAGCTGCTGCGCGAGCTGCACGACGCCAAGGAAAGCGCCGACGCCGCCAACCGGGCCAAG
ACCACGTTCCTGGCAACGATGAGCCACGAGATCCGCACGCCGATGaACGCGATCATCGGCATGCTGGAGCTGGCGCTGCT
CCGTCCGACCGACCAGGAGCCGGATCGCCAGTCCATCCAGGTCGCGTACGACTCGGCCCGCAGCCTGCTGGAGCTGATAG
GCGACATCCTGGACATTGCGAAGATCGAGGCGGGAAAATTCGACCTGGCGCCGGTGCGCACGGCGCTGCGCGTCCTGCCC
GAAGGGGCGATCCGCGTCTTCGACGGATTGGCGCGCCAAAAAGGCATAGAGCTGGTATTGAAGACCGACATCGTGGGCGT
CGACGATGTATTGATAGACCCCTTGCGCATGAAGCAAGTGCTCTCGAACCTGGTGGGCAACGCCATCAAGTTCACCACCG
AAGGCCAGGTTGTCCTTGCCGTGACCGCACGCCCCGACGGCGACGCCGCGCACGTGCAGTTCAGCGTGAGCGACACCGGC
TGCGGCATCAGCGAGGCCGACCAACGGCAGCTGTTCAAACCGTTCTCGCAAGTGGGTGGCAGCGCCGAGGCCGGGCCGGC
GCCGGGCACCGGCCTGGGCCTGTCCATCAGCCGGCGCCTCGTCGAATTGATGGGGGGAACGCTGGTCATGCGCAGCGCGC
CAGGGGTGGGCACAACGGTTTCGGTGGACCTGAGGCTGACCATGGTCGAAAAATCCGTGCAGGCCGCGCCGCCCGCTGCG
GCCACTGCGGCCACGCCGTCCAAGCCGCAGGTATCGCTGCGCGTGCTGGTCGTCGATGACCACAAACCCAACCTGATGCT
GCTGCGCCAGCAGCTGGACTACCTGGGCCAGCGTGTCATCGCCGCCGACTCCGGCGAAGCCGCCCTGGcCCTGTGGCGCG
AGCATGCGTTCGACGTCGTGATCACCGATtGCAACATGCCCGGTATCAGCGGCTACGAATTGGCGCGCCGCATACGCGCC
GCCGAGGCCGCGCCCGGTTACGGACGTACGCGGTGCATTCTGTTCGGCTTCACGGCTTCGGCGCAGATGGACGAAGCGCA
GCGCTGCCGCGCCGCCGGCATGGACGACTGCCTGTTCAAGCCGATCGGCGTGGACGCCTTGCGGCAACGCTTGAACGAAG
CCGTGGCACGGGCCGCGCTCCCCACGCCCCCCTCGCCCCAGGCTGCCGCGCCGGCCACGGACGACGCCACCCCGACGGCG
TTCTCGGCCGAGTCGATTCTTGCCTTGACGCAGAACGATGAGGCGCTGATCCGGCAATTGCTCGAAGAAGTGATTCGCAC
CAACCGGGCGGACGTCGACCAATTGCAAAAGCTGCACCAGCAGGCCGATTGGCCGAAGGTCTCGGACATGGCGCACAGGC
TGGCCGGCGGCGCGCGCGTGGTCGATGCCAAGGCCATGATAGACACTGTGCTGGCGCTGGAGAAAAAAGCGCAAGGCCAG
GCTGGCCCCTCACCCGAAATCGACGGCCTGGTACGTACGCTTGCGGCGCAGTCCGCCGCGCTGGAGACGCAACTGCGCGC
CTGGCTGGAGCAACGGCCGCATCAAGATCAGCCCTGA
>Bp_B1193.seq
ATGCCCGCCCCGCACCGCCTGTACCCCCGCAGTCTGATCTGCCTGGCTCAGGCGCTATTGGCATGGGCTTTGCTGGCATG
GGCGCCCGCGCAGGCAAGCCAGGAGCTGACCCTGGTCGGCAAGGCTGCCGTTCCCGACGTCGAGGTCGCGCTCGACGGCG
ACGACTGGCGTTGGCTGGCCCGCAAGCGGGTACTGACGCTGGGTGTGTACGCACCGGACATTCCTCCGTTCGACGTCACC
TATGGCGAACGCTACGAAGGCCTGACGGCCGACTACATGGCGATCATCGCGCACAACCTGGGGATGCAGGCGAAAGTGCT
GCGATACCCCACGCGCGAACAAGCCCTCAGCGCGCTGGAAAGCGGGCAGATCGACCTCATCGGCACCGTCAATGGCACGG
ACGGCCGGCAACAGAGCCTGCGTCTGAGCGTTCCCTACGCCGCCGACCACCCGGTGATCGTCATGCCCATCGGCGCACGC
CACGTTCCAGCCTCGAACCTGGCCGGCCAGCGGCTGGCGGTCGACATCAACTACCTGCCCAAGGAAACGCTCGCACGGGC
CTACCCGCAGGCTACGCTGCATTACTTCCCCTCATCCGAGCAGGCGCTGGCCGCGGTGGCCTATGGGCAGGCCGACGTAT
TCATCGGCGATGCCCTGACCACCTCGCACCTCGTATCGCAAaGCTATTTCAATGACGTTCGCGTAGTCGCCCCGGCCCAT
ATCGCGACGGGCGGAGAATCCTTCGGCGTGCGCGCCGACAACACCCGCCTGCTGCGGGTGGTCAACGCCGTACTCGAAGC
CATTCCGCCTTCCGAACACCGCAGCCTGATCTACCGCTGGGGACTGGGCAGCAGCATTTCGCTCGATTTCGCGCACCCCG
CGTATTCCGCGCGCGAGCAGCAATGGATGGCAGACCACCCCGTCGTCAAGGTGGCGGTCCTGAATCTGTTCGCGCCCTTC
ACCCTGTTCCGCACCGACGAACAGTTCGGCGGGATCAGCGCCGCCGTGCTGCAGCTGCTGCAATTGCGCACCGGCCTGGA
CTTCGAGATCATCGGCGTCGACACGGTCGAGGAACTGATAGCCAAGCTGCGTTCGGGCGAAGCCGACATGGCCGGCGCCC
TGTTCGTCAACAGCGCGCGGGAGTCCTTCCTCAGTTTCAGCCGGCCGTATGTGCGCAATGGCATGGTGATCGTCACGCGC
CAGGACCCCGACGCGCCCGTCGACGCCGATCATCTGGACGGCCGCACGGTCGCGTTGGTGCGCAACAGCGCCGCCATTCC
CCTGCTGCAGCGGCGCTATCCCCAGGCGAAGGTGGTGACCGCCGACAACCCGAGCGAGGCGATGCTGATGGTGGCCAATG
GACAGGCCGACGCCGTCGTGCAGACGCAGATCAGCGCCAGCTATTACGTCAACCGCTACTTCGCCGGCAAGCTGCGCATC
GCCTCGGCGCTGGACCTGCCCCCGGCCGAGATCGCGCTGGCGACGACGCGCGGCCAGACCGAACTGATGTCCATCCTGAA
CAAGGCGCTCTACAGCATTTCGAACGACGAGCTCGCCTCCATCATCAGCCGCTGGCGCGGCAGCGACGGCGATCCGCGCA
CCTGGTACGCCTACCGCAACGAGATCTACCTGCTGATCGGGCTGGGCCTGTTGTCGGCCCTGCTGTTCCTGAGCTGGATC
GTCTACCTGCGGCGCCAGATCCGCCAGCGCAAGCGGGCCGAGCGGGCGCTGAACGACCAGCTGGAATTCATGCGCGTGCT
CATCGACGGCACGCCTAACCCCATCTATGTGCGCGATAAGGAAGGCCGCATGCTGTTGTGCAATGACGCCTACCTCGACA
CCTTTGGCGTGACTGCCGATGCGGTACTGGGCAAGACCATTCCGGAAGCCAACGTGGTGGGCGACCCGGCGCTGGCCCGC
GAAATGCACGAGTTCCTGCTCACGCGCGTGGCCGCCGAGCGCGAGCCGCGCTTCGAGGACCGCGATGTCACGCTGCACGG
CCGCACCCGCCATGTCTACCAGTGGACGATTCCGTACGGCGACTCGCTGGGCGAACTCAAGGGCATCATCGGCGGCTGGA
TCGACATCACCGAACGCGCCGAGCTGCTGCGCGAGCTGCACGACGCCAAGGAAAGCGCCGACGCCGCCAACCGGGCCAAG
ACCACGTTCCTGGCAACGATGAGCCACGAGATCCGCACGCCGATGAACGCGATCATCGGCATGCTGGAGCTGGCGCTGCT
CCGTCCGACCGACCAGGAGCCGGATCGCCAGTCCATCCAGGTCGCGTACGACTCGGCCCGCAGCCTGCTGGAGCTGATAG
GCGACATCCTGGACATTGCGAAGATCGAGGCGGGAAAATTCGACCTGGCGCCGGTGCGCACGGCGCTGCGCGTCCTGCCC
GAAGGGGCGATCCGCGTCTTCGACGGATTGGCGCGCCAAAAAGGCATAGAGCTGGTATTGAAGACCGACATCGTGGGCGT
CGACGATGTATTGATAGACCCCTTGCGCATGAAGCAAGTGCTCTCGAACCTGGTGGGCAACGCCATCAAGTTCACCACCG
AAGGCCAGGTTGTCCTTGCCGTGACCGCACGCCCCGACGGCGACGCCGCGCACGTGCAGTTCAGCGTGAGCGACACCGGC
TGCGGCATCAGCGAGGCCGACCAACGGCAGCTGTTCAAACCGTTCTCGCAAGTGGGTGGCAGCGCCGAGGCCGGGCCGGC
GCCGGGCACCGGCCTGGGCCTGTCCATCAGCCGGCGCCTCGTCGAATTGATGGGGGGAACGCTGGTCATGCGCAGCGCGC
CAGGGGTGGGCACAACGGTTTCGGTGGACCTGAGGCTGACCATGGTCGAAAAATCCGTGCAGGCCGCGCCGCCCGCTGCG
GCCACTGCGGCCACGCCGTCCAAGCCGCAGGTATCGCTGCGCGTGCTGGTCGTCGATGACCACAAACCCAACCTGATGCT
GCTGCGCCAGCAGCTGGACTACCTGGGCCAGCGTGTCATCGCCGCCGACTCCGGCGAAGCCGCCCTGGCCCTGTGGCGCG
AGCATGCGTTCGACGTCGTGATCACCGATTGCAACATGCCCGGTATCAGCGGCTACGAATTGGCGCGCCGCATACGCGCC
GCCGAGGCCGCGCCCGGTTACGGACGTACGCGGTGCATTCTGTTCGGCTTCACGGCTTCGGCGCAGATGGACGAAGCGCA
GCGCTGCCGCGCCGCCGGCATGGACGACTGCCTGTTCAAGCCGATCGGCGTGGACGCCTTGCGGCAACGCTTGAACGAAG
CCGTGGCACGGGCCGCGCTCCCCACGCCCCCCTCGCCCCAGGCTGCCGCGCCGGCCACGGACGACGCCACCCCGACGGCG
TTCTCGGCCGAGTCGATTCTTGCCTTGACGCAGAACGATGAGGCGCTGATCCGGCAATTGCTCGAAGAAGTGATTCGCAC
CAACCGGGCGGACGTCGACCAATTGCAAAAGCTGCACCAGCAGGCCGATTGGCCGAAGGTCTCGGACATGGCGCACAGGC
TGGCCGGCGGCGCGCGCGTGGTCGATGCCAAGGCCATGATAGACACTGTGCTGGCGCTGGAGAAAAAAGCGCAAGGCCAG
GCTGGCCCCTCACCCGAAATCGACGGCCTGGTACGTACGCTTGCGGCGCAGTCCGCCGCGCTGGAGACGCAACTGCGCGC
CTGGCTGGAGCAACGGCCGCATCAAGATCAGCCCTGA
>Bp_B1198.seq
ATGCCCGCCCCGCACCGCCTGTACCCCCGCAGTCTGATCTGCCTGGCTCAGGCGCTATTGGCATGGGCTTTGCTGGCATG
GGCGCCCGCGCAGGCAAGCCAGGAGCTGACCCTGGTCGGCAAGGCTGCCGTTCCCGACGTCGAGGTCGCGCTCGACGGCG
ACGACTGGCGTTGGCTGGCCCGCAAGCGGGTACTGACGCTGGGTGTGTACGCACCGGACATTCCTCCGTTCGACGTCACC
TATGGCGAACGCTACGAAGGCCTGACGGCCGACTACATGGCGATCATCGCGCACAACCTGGGGATGCAGGCGAAAGTGCT
GCGATACCCCACGCGCGAACAAGCCCTCAGCGCGCTGGAAAGCGGGCAGATCGACCTCATCGGCACCGTCAATGGCACGG
ACGGCCGGCAACAGAGCCTGCGTCTGAGCGTTCCCTACGCCGCCGACCACCCGGTGATCGTCATGCCCATCGGCGCACGC
CACGTTCCAGCCTCGAACCTGGCCGGCCAGCGGCTGGCGGTCGACATCAACTACCTGCCCAAGGAAACGCTCGCACGGGC
CTACCCGCAGGCTACGCTGCATTACTTCCCCTCATCCGAGCAGGCGCTGGCCGCGGTGGCCTATGGGCAGGCCGACGTAT
TCATCGGCGATGCCCTGACCACCTCGCACCTCGTATCGCAAAGCTATTTCAATGACGTTCGCGTAGTCGCCCCGGCCCAT
ATCGCGACGGGCGGAGAATCCTTCGGCGTGCGCGCCGACAACACCCGCCTGCTGCGGGTGGTCAACGCCGTACTCGAAGC
CATTCCGCCTTCCGAACACCGCAGCCTGATCTACCGCTGGGGACTGGGCAGCAGCATTTCGCTCGATTTCGCGCACCCCG
CGTATTCCGCGCGCGAGCAGCAATGGATGGCAGACCACCCCGTCGTCAAGGTGGCGGTCCTGAATCTGTTCGCGCCCTTC
ACCCTGTTCCGCACCGACGAACAGTTCGGCGGGATCAGCGCCGCCGTGCTGCAGCTGCTGCAATTGCGCACCGGCCTGGA
CTTCGAGATCATCGGCGTCGACACGGTCGAGGAACTGATAGCCAAGCTGCGTTCGGGCGAAGCCGACATGGCCGGCGCCC
TGTTCGTCAACAGCGCGCGGGAGTCCTTCCTCAGTTTCAGCCGGCCGTATGTGCGCAATGGCATGGTGATCGTCACGCGC
CAGGACCCCGACGCGCCCGTCGACGCCGATCATCTGGACGGCCGCACGGTCGCGTTGGTGCGCAACAGCGCCGCCATTCC
CCTGCTGCAGCGGCGCTATCCCCAGGCGAAGGTGGTGACCGCCGACAACCCGAGCGAGGCGATGCTGATGGTGGCCAATG
GACAGGCCGACGCCGTCGTGCAGACGCAGATCAGCGCCAGCTATTACGTCAACCGCTACTTCGCCGGCAAGCTGCGCATC
GCCTCGGCGCTGGACCTGCCCCCGGCCGAGATCGCGCTGGCGACGACGCGCGGCCAGACCGAACTGATGTCCATCCTGAA
CAAGGCGCTCTACAGCATTTCGAACGACGAGCTCGCCTCCATCATCAGCCGCTGGCGCGGCAGCGACGGCGATCCGCGCA
CCTGGTACGCCTACCGCAACGAGATCTACCTGCTGATCGGGCTGGGCCTGTTGTCGGCCCTGCTGTTCCTGAGCTGGATC
GTCTACCTGCGGCGCCAGATCCGCCAGCGCAAGCGGGCCGAGCGGGCGCTGAACGACCAGCTGGAATTCATGCGCGTGCT
CATCGACGGCACGCCTAACCCCATCTATGTGCGCGATAAGGAAGGCCGCATGCTGTTGTGCAATGACGCCTACCTCGACA
CCTTTGGCGTGACTGCCGATGCGGTACTGGGCAAGACCATTCCGGAAGCCAACGTGGTGGGCGACCCGGCGCTGGCCCGC
GAAATGCACGAGTTCCTGCTCACGCGCGTGGCCGCCGAGCGCGAGCCGCGCTTCGAGGACCGCGATGTCACGCTGCACGG
CCGCACCCGCCATGTCTACCAGTGGACGATTCCGTACGGCGACTCGCTGGGCGAACTCAAGGGCATCATCGGCGGCTGGA
TCGACATCACCGAACGCGCCGAGCTGCTGCGCGAGCTGCACGACGCCAAGGAAAGCGCCGACGCCGCCAACCGGGCCAAG
ACCACGTTCCTGGCAACGATGAGCCACGAGATCCGCACGCCGATGAACGCGATCATCGGCATGCTGGAGCTGGCGCTGCT
CCGTCCGACCGACCAGGAGCCGGATCGCCAGTCCATCCAGGTCGCGTACGACTCGGCCCGCAGCCTGCTGGAGCTGATAG
GCGACATCCTGGACATTGCGAAGATCGAGGCGGGAAAATTCGACCTGGCGCCGGTGCGCACGGCGCTGCGCGTCCTGCCC
GAAGGGGCGATCCGCGTCTTCGACGGATTGGCGCGCCAAAAAGGCATAGAGCTGGTATTGAAGACCGACATCGTGGGCGT
CGACGATGTATTGATAGACCCCTTGCGCATGAAGCAAGTGCTCTCGAACCTGGTGGGCAACGCCATCAAGTTCACCACCG
AAGGCCAGGTTGTCCTTGCCGTGACCGCACGCCCCGACGGCGACGCCGCGCACGTGCAGTTCAGCGTGAGCGACACCGGC
TGCGGCATCAGCGAGGCCGACCAACGGCAGCTGTTCAAACCGTTCTCGCAAGTGGGTGGCAGCGCCGAGGCCGGGCCGGC
GCCGGGCACCGGCCTGGGCCTGTCCATCAGCCGGCGCCTCGTCGAATTGATGGGGGGAACGCTGGTCATGCGCAGCGCGC
CAGGGGTGGGCACAACGGTTTCGGTGGACCTGAGGCTGACCATGGTCGAAAAATCCGTGCAGGCCGCGCCGCCCGCTGCG
GCCACTGCGGCCACGCCGTCCAAGCCGCAGGTATCGCTGCGCGTGCTGGTCGTCGATGACCACAAACCCAACCTGATGCT
GCTGCGCCAGCAGCTGGACTACCTGGGCCAGCGTGTCATCGCCGCCGACTCCGGCGAAGCCGCCCTGGcCCTGTGGCGCG
AGCATGCGTTCGACGTCGTGATCACCGATTGCAACATGCCCGGTATCAGCGGCTACGAATtGGCGCGCCGCATACGCGCC
GCCGAGGCCGCGCCCGGTTACGGACGTACGCGGTGCATTCTGTTCGGCTTCACGGCTTCGGCGCAGATGGACGAAGCGCA
GCGCTGCCGCGCCGCCGGCATGGACGACTGCCTGTTCAAGCCGATCGGCGTGGACGCCTTGCGGCAACGCTTGAACGAAG
CCGTGGCACGGGCCGCGCTCCCCACGCCCCCCTCGCCCCAGGCTGCCGCGCCGGCCACGGACGACGCCACCCCGACGGCG
TTCTCGGCCGAGTCGATTCTTGCCTTGACGCAGAACGATGAGGCGCTGATCCGGCAATTGCTCGAAGAAGTGATTCGCAC
CAACCGGGCGGACGTCGACCAATTGCAAAAGCTGCACCAGCAGGCCGATTGGCCGAAGGTCTCGGACATGGCGCACAGGC
TGGCCGGCGGCGCGCGCGTGGTCGATGCCAAGGCCATGATAGACACTGTGCTGGCGCTGGAGAAAAAAGCGCAAGGCCAG
GCTGGCCCCTCACCCGAAATCGACGGCCTGGTACGTACGCTTGCGGCGCAGTCCGCCGCGCTGGAGACGCAACTGCGCGC
CTGGCTGGAGCAACGGCCGCATCAAGATCAGCCCTGA
>Bp_B1325.seq
ATGCCCGCCCCGCACCGCCTGTACCCCCGCAGTCTGATCTGCCTGGCTCAGGCGCTATTGGCATGGGCTTTGCTGGCATG
GGCGCCCGCGCAGGCAAGCCAGGAGCTGACCCTGGTCGGCAAGGCTGCCGTTCCCGACGTCGAGGTCGCGCTCGACGGCG
ACGACTGGCGTTGGCTGGCCCGCAAGCGGGTACTGACGCTGGGTGTGTACGCACCGGACATTCCTCCGTTCGACGTCACC
TATGGCGAACGCTACGAAGGCCTGACGGCCGACTACATGGCGATCATCGCGCACAACCTGGGGATGCAGGCGAAAGTGCT
GCGATACCCCACGCGCGAACAAGCCCTCAGCGCGCTGGAAAGCGGGCAGATCGACCTCATCGGCACCGTCAATGGCACGG
ACGGCCGGCAACAGAGCCTGCGTCTGAGCGTTCCCTACGCCGCCGACCACCCGGTGATCGTCATGCCCATCGGCGCACGC
CACGTTCCAGCCTCGAACCTGGCCGGCCAGCGGCTGGCGGTCGACATCAACTACCTGCCCAAGGAAACGCTCGCACGGGC
CTACCCGCAGGCTACGCTGCATTACTTCCCCTCATCCGAGCAGGCGCTGGCCGCGGTGGCCTATGGGCAGGCCGACGTAT
TCATCGGCGATGCCCTGACCACCTCGCACCTCGTATCGCAAAGCTATTTCAATGACGTTCGCGTAGTCGCCCCGGCCCAT
ATCGCGACGGGCGGAGAATCCTTCGGCGTGCGCGCCGACAACACCCGCCTGCTGCGGGTGGTCAACGCCGTACTCGAAGC
CATTCCGCCTTCCGAACACCGCAGCCTGATCTACCGCTGGGGACTGGGCAGCAGCATTTCGCTCGATTTCGCGCACCCCG
CGTATTCCGCGCGCGAGCAGCAATGGATGGCAGACCACCCCGTCGTCAAGGTGGCGGTCCTGAATCTGTTCGCGCCCTTC
ACCCTGTTCCGCACCGACGAACAGTTCGGCGGGATCAGCGCCGCCGTGCTGCAGCTGCTGCAATTGCGCACCGGCCTGGA
CTTCGAGATCATCGGCGTCGACACGGTCGAGGAACTGATAGCCAAGCTGCGTTCGGGCGAAGCCGACATGGCCGGCGCCC
TGTTCGTCAACAGCGCGCGGGAGTCCTTCCTCAGTTTCAGCCGGCCGTATGTGCGCAATGGCATGGTGATCGTCACGCGC
CAGGACCCCGACGCGCCCGTCGACGCCGATCATCTGGACGGCCGCACGGTCGCGTTGGTGCGCAACAGCGCCGCCATTCC
CCTGCTGCAGCGGCGCTATCCCCAGGCGAAGGTGGTGACCGCCGACAACCCGAGCGAGGCGATGCTGATGGTGGCCAATG
GACAGGCCGACGCCGTCGTGCAGACGCAGATCAGCGCCAGCTATTACGTCAACCGCTACTTCGCCGGCAAGCTGCGCATC
GCCTCGGCGCTGGACCTGCCCCCGGCCGAGATCGCGCTGGCGACGACGCGCGGCCAGACCGAACTGATGTCCATCCTGAA
CAAGGCGCTCTACAGCATTTCGAACGACGAGCTCGCCTCCATCATCAGCCGCTGGCGCGGCAGCGACGGCGATCCGCGCA
CCTGGTACGCCTACCGCAACGAGATCTACCTGCTGATCGGGCTGGGCCTGTTGTCGGCCCTGCTGTTCCTGAGCTGGATC
GTCTACCTGCGGCGCCAGATCCGCCAGCGCAAGCGGGCCGAGCGGGCGCTGAACGACCAGCTGGAATTCATGCGCGTGCT
CATCGACGGCACGCCTAACCCCATCTATGTGCGCGATAAGGAAGGCCGCATGCTGTTGTGCAATGACGCCTACCTCGACA
CCTTTGGCGTGACTGCCGATGCGGTACTGGGCAAGACCATTCCGGAAGCCAACGTGGTGGGCGACCCGGCGCTGGCCCGC
GAAATGCACGAGTTCCTGCTCACGCGCGTGGCCGCCGAGCGCGAGCCGCGCTTCGAGGACCGCGATGTCACGCTGCACGG
CCGCACCCGCCATGTCTACCAGTGGACGATTCCGTACGGCGACTCGCTGGGCGAACTCAAGGGCATCATCGGCGGCTGGA
TCGACATCACCGAACGCGCCGAGCTGCTGCGCGAGCTGCACGACGCCAAGGAAAGCGCCGACGCCGCCAACCGGGCCAAG
ACCACGTTCCTGGCAACGATGAGCCACGAGATCCGCACGCCGATGAACGCGATCATCGGCATGCTGGAGCTGGCGCTGCT
CCGTCCGACCGACCAGGAGCCGGATCGCCAGTCCATCCAGGTCGCGTACGACTCGGCCCGCAGCCTGCTGGAGCTGATAG
GCGACATCCTGGACATTGCGAAGATCGAGGCGGGAAAATTCGACCTGGCGCCGGTGCGCACGGCGCTGCGCGTCCTGCCC
GAAGGGGCGATCCGCGTCTTCGACGGATTGGCGCGCCAAAAAGGCATAGAGCTGGTATTGAAGACCGACATCGTGGGCGT
CGACGATGTATTGATAGACCCCTTGCGCATGAAGCAAGTGCTCTCGAACCTGGTGGGCAACGCCATCAAGTTCACCACCG
AAGGCCAGGTTGTCCTTGCCGTGACCGCACGCCCCGACGGCGACGCCGCGCACGTGCAGTTCAGCGTGAGCGACACCGGC
TGCGGCATCAGCGAGGCCGACCAACGGCAGCTGTTCAAACCGTTCTCGCAAGTGGGTGGCAGCGCCGAGGCCGGGCCGGC
GCCGGGCACCGGCCTGGGCCTGTCCATCAGCCGGCGCCTCGTCGAATTGATGGGGGGAACGCTGGTCATGCGCAGCGCGC
CAGGGGTGGGCACAACGGTTTCGGTGGACCTGAGGCTGACCATGGTCGAAAAATCCGTGCAGGCCGCGCCGCCCGCTGCG
GCCACTGCGGCCACGCCGTCCAAGCCGCAGGTATCGCTGCGCGTGCTGGTCGTCGATGACCACAAACCCAACCTGATGCT
GCTGCGCCAGCAGCTGGACTACCTGGGCCAGCGTGTCATCGCCGCCGACTCCGGCGAAGCCGCCCTGGCCCTGTGGCGCG
AGCATGCGTTCGACGTCGTGATCACCGATTGCAACATGCCCGGTATCAGCGGCTACGAATTGGCGCGCCGCATACGCGCC
GCCGAGGCCGCGCCCGGTTACGGACGTACGCGGTGCATTCTGTTCGGCTTCACGGCTTCGGCGCAGATGGACGAAGCGCA
GCGCTGCCGCGCCGCCGGCATGGACGACTGCCTGTTCAAGCCGATCGGCGTGGACGCCTTGCGGCAACGCTTGAACGAAG
CCGTGGCACGGGCCGCGCTCCCCACGCCCCCCTCGCCCCAGGCTGCCGCGCCGGCCACGGACGACGCCACCCCGACGGCG
TTCTCGGCCGAGTCGATTCTTGCCTTGACGCAGAACGATGAGGCGCTGATCCGGCAATTGCTCGAAGAAGTGATTCGCAC
CAACCGGGCGGACGTCGACCAATTGCAAAAGCTGCACCAGCAGGCCGATTGGCCGAAGGTCTCGGACATGGCGCACAGGC
TGGCCGGCGGCGCGCGCGTGGTCGATGCCAAGGCCATGATAGACACTGTGCTGGCGCTGGAGAAAAAAGCGCAAGGCCAG
GCTGGCCCCTCACCCGAAATCGACGGCCTGGTACGTACGCTTGCGGCGCAGTCCGCCGCGCTGGAGACGCAACTGCGCGC
CTGGCTGGAGCAACGGCCGCATCAAGATCAGCCCTGA
>Bp_B0006.seq
ATGCCCGCCCCGCACCGCCTGTACCCCCGCAGTCTGATCTGCCTGGCTCAGGCGCTATTGGCATGGGCTTTGCTGGCATG
GGCGCCCGCGCAGGCAAGCCAGGAGCTGACCCTGGTCGGCAAGGCTGCCGTTCCCGACGTCGAGGTCGCGCTCGACGGCG
ACGACTGGCGTTGGCTGGCCCGCAAGCGGGTACTGACGCTGGGTGTGTACGCACCGGACATTCCTCCGTTCGACGTCACC
TATGGCGAACGCTACGAAGGCCTGACGGCCGACTACATGGCGATCATCGCGCACAACCTGGGGATGCAGGCGAAAGTGCT
GCGATACCCCACGCGCGAACAAGCCCTCAGCGCGCTGGAAAGCGGGCAGATCGACCTCATCGGCACCGTCAATGGCACGG
ACGGCCGGCAACAGAGCCTGCGTCTGAGCGTTCCCTACGCCGCCGACCACCCGGTGATCGTCATGCCCATCGGCGCACGC
CACGTTCCAGCCTCGAACCTGGCCGGCCAGCGGCTGGCGGTCGACATCAACTACCTGCCCAAGGAAACGCTCGCACGGGC
CTACCCGCAGGCTACGCTGCATTACTTCCCCTCATCCGAGCAGGCGCTGGCCGCGGTGGCCTATGGGCAGGCCGACGTAT
TCATCGGCGATGCCCTGACCACCTCGCACCTCGTATCGCAAAGCTATTTCAATGACGTTCGCGTAGTCGCCCCGGCCCAT
ATCGCGACGGGCGGAGAATCCTTCGGCGTGCGCGCCGACAACACCCGCCTGCTGCGGGTGGTCAACGCCGTACTCGAAGC
CATTCCGCCTTCCGAACACCGCAGCCTGATCTACCGCTGGGGACTGGGCAGCAGCATTTCGCTCGATTTCGCGCACCCCG
CGTATTCCGCGCGCGAGCAGCAATGGATGGCAGACCACCCCGTCGTCAAGGTGGCGGTCCTGAATCTGTTCGCGCCCTTC
ACCCTGTTCCGCACCGACGAACAGTTCGGCGGGATCAGCGCCGCCGTGCTGCAGCTGCTGCAATTGCGCACCGGCCTGGA
CTTCGAGATCATCGGCGTCGACACGGTCGAGGAACTGATAGCCAAGCTGCGTTCGGGCGAAGCCGACATGGCCGGCGCCC
TGTTCGTCAACAGCGCGCGGGAGTCCTTCCTCAGTTTCAGCCGGCCGTATGTGCGCAATGGCATGGTGATCGTCACGCGC
CAGGACCCCGACGCGCCCGTCGACGCCGATCATCTGGACGGCCGCACGGTCGCGTTGGTGCGCAACAGCGCCGCCATTCC
CCTGCTGCAGCGGCGCTATCCCCAGGCGAAGGTGGTGACCGCCGACAACCCGAGCGAGGCGATGCTGATGGTGGCCAATG
GACAGGCCGACGCCGTCGTGCAGACGCAGATCAGCGCCAGCTATTACGTCAACCGCTACTTCGCCGGCAAGCTGCGCATC
GCCTCGGCGCTGGACCTGCCCCCGGCCGAGATCGCGCTGGCGACGACGCGCGGCCAGACCGAACTGATGTCCATCCTGAA
CAAGGCGCTCTACAGCATTTCGAACGACGAGCTCGCCTCCATCATCAGCCGCTGGCGCGGCAGCGACGGCGATCCGCGCA
CCTGGTACGCCTACCGCAACGAGATCTACCTGCTGATCGGGCTGGGCCTGTTGTCGGCCCTGCTGTTCCTGAGCTGGATC
GTCTACCTGCGGCGCCAGATCCGCCAGCGCAAGCGGGCCGAGCGGGCGCTGAACGACCAGCTGGAATTCATGCGCGTGCT
CATCGACGGCACGCCTAACCCCATCTATGTGCGCGATAAGGAAGGCCGCATGCTGTTGTGCAATGACGCCTACCTCGACA
CCTTTGGCGTGACTGCCGATGCGGTACTGGGCAAGACCATTCCGGAAGCCAACGTGGTGGGCGACCCGGCGCTGGCCCGC
GAAATGCACGAGTTCCTGCTCACGCGCGTGGCCGCCGAGCGCGAGCCGCGCTTCGAGGACCGCGATGTCACGCTGCACGG
CCGCACCCGCCATGTCTACCAGTGGACGATTCCGTACGGCGACTCGCTGGGCGAACTCAAGGGCATCATCGGCGGCTGGA
TCGACATCACCGAACGCGCCGAGCTGCTGCGCGAGCTGCACGACGCCAAGGAAAGCGCCGACGCCGCCAACCGGGCCAAG
ACCACGTTCCTGGCAACGATGAGCCACGAGATCCGCACGCCGATGaACGCGATCATCGGCATGCTGGAGCTGGCGCTGCT
CCGTCCGACCGACCAGGAGCCGGATCGCCAGTCCATCCAGGTCGCGTACGACTCGGCCCGCAGCCTGCTGGAGCTGATAG
GCGACATCCTGGACATTGCGAAGATCGAGGCGGGAAAATTCGACCTGGCGCCGGTGCGCACGGCGCTGCGCGTCCTGCCC
GAAGGGGCGATCCGCGTCTTCGACGGATTGGCGCGCCAAAAAGGCATAGAGCTGGTATTGAAGACCGACATCGTGGGCGT
CGACGATGTATTGATAGACCCCTTGCGCATGAAGCAAGTGCTCTCGAACCTGGTGGGCAACGCCATCAAGTTCACCACCG
AAGGCCAGGTTGTCCTTGCCGTGACCGCACGCCCCGACGGCGACGCCGCGCACGTGCAGTTCAGCGTGAGCGACACCGGC
TGCGGCATCAGCGAGGCCGACCAACGGCAGCTGTTCAAACCGTTCTCGCAAGTGGGTGGCAGCGCCGAGGCCGGGCCGGC
GCCGGGCACCGGCCTGGGCCTGTCCATCAGCCGGCGCCTCGTCGAATTGATGGGGGGAACGCTGGTCATGCGCAGCGCGC
CAGGGGTGGGCACAACGGTTTCGGTGGACCTGAGGCTGACCATGGTCGAAAAATCCGTGCAGGCCGCGCCGCCCGCTGCG
GCCACTGCGGCCACGCCGTCCAAGCCGCAGGTATCGCTGCGCGTGCTGGTCGTCGATGACCACAAACCCAACCTGATGCT
GCTGCGCCAGCAGCTGGACTACCTGGGCCAGCGTGTCATCGCCGCCGACTCCGGCGAAGCCGCCCTGGCCCTGTGGCGCG
AGCATGCGTTCGACGTCGTGATCACCGATTGCAACATGCCCGGTATCAGCGGCTACGAATtGGCGCGCCGCAtACGCGCC
GCCGAGGCCGCGCCCGGTTACGGACGTACGCGGTGCATTCTGTTCGGCTTCACGGCTTCGGCGCAGATGGACGAAGCGCA
GCGCTGCCGCGCCGCCGGCATGGACGACTGCCTGTTCAAGCCGATCGGCGTGGACGCCTTGCGGCAACGCTTGAACGAAG
CCGTGGCACGGGCCGCGCTCCCCACGCCCCCCTCGCCCCAGGCTGCCGCGCCGGCCACGGACGACGCCACCCCGACGGCG
TTCTCGGCCGAGTCGATTCTTGCCTTGACGCAGAACGATGAGGCGCTGATCCGGCAATTGCTCGAAGAAGTGATTCGCAC
CAACCGGGCGGACGTCGACCAATTGCAAAAGCTGCACCAGCAGGCCGATTGGCCGAAGGTCTCGGACATGGCGCACAGGC
TGGCCGGCGGCGCGCGCGTGGTCGATGCCAAGGCCATGATAGACACTGTGCTGGCGCTGGAGAAAAAAGCGCAAGGCCAG
GCTGGCCCCTCACCCGAAATCGACGGCCTGGTACGTACGCTTGCGGCGCAGTCCGCCGCGCTGGAGACGCAACTGCGCGC
CTGGCTGGAGCAACGGCCGCATCAAGATCAGCCCTGA
>Bp_B0213.seq
ATGCCCGCCCCGCACCGCCTGTACCCCCGCAGTCTGATCTGCCTGGCTCAGGCGCTATTGGCATGGGCTTTGCTGGCATG
GGCGCCCGCGCAGGCAAGCCAGGAGCTGACCCTGGTCGGCAAGGCTGCCGTTCCCGACGTCGAGGTCGCGCTCGACGGCG
ACGACTGGCGTTGGCTGGCCCGCAAGCGGGTACTGACGCTGGGTGTGTACGCACCGGACATTCCTCCGTTCGACGTCACC
TATGGCGAACGCTACGAAGGCCTGACGGCCGACTACATGGCGATCATCGCGCACAACCTGGGGATGCAGGCGAAAGTGCT
GCGATACCCCACGCGCGAACAAGCCCTCAGCGCGCTGGAAAGCGGGCAGATCGACCTCATCGGCACCGTCAATGGCACGG
ACGGCCGGCAACAGAGCCTGCGTCTGAGCGTTCCCTACGCCGCCGACCACCCGGTGATCGTCATGCCCATCGGCGCACGC
CACGTTCCAGCCTCGAACCTGGCCGGCCAGCGGCTGGCGGTCGACATCAACTACCTGCCCAAGGAAACGCTCGCACGGGC
CTACCCGCAGGCTACGCTGCATTACTTCCCCTCATCCGAGCAGGCGCTGGCCGCGGTGGCCTATGGGCAGGCCGACGTAT
TCATCGGCGATGCCCTGACCACCTCGCACCTCGTATCGCAAAGCTATTTCAATGACGTTCGCGTAGTCGCCCCGGCCCAT
ATCGCGACGGGCGGAGAATCCTTCGGCGTGCGCGCCGACAACACCCGCCTGCTGCGGGTGGTCAACGCCGTACTCGAAGC
CATTCCGCCTTCCGAACACCGCAGCCTGATCTACCGCTGGGGACTGGGCAGCAGCATTTCGCTCGATTTCGCGCACCCCG
CGTATTCCGCGCGCGAGCAGCAATGGATGGCAGACCACCCCGTCGTCAAGGTGGCGGTCCTGAATCTGTTCGCGCCCTTC
ACCCTGTTCCGCACCGACGAACAGTTCGGCGGGATCAGCGCCGCCGTGCTGCAGCTGCTGCAATTGCGCACCGGCCTGGA
CTTCGAGATCATCGGCGTCGACACGGTCGAGGAACTGATAGCCAAGCTGCGTTCGGGCGAAGCCGACATGGCCGGCGCCC
TGTTCGTCAACAGCGCGCGGGAGTCCTTCCTCAGTTTCAGCCGGCCGTATGTGCGCAATGGCATGGTGATCGTCACGCGC
CAGGACCCCGACGCGCCCGTCGACGCCGATCATCTGGACGGCCGCACGGTCGCGTTGGTGCGCAACAGCGCCGCCATTCC
CCTGCTGCAGCGGCGCTATCCCCAGGCGAAGGTGGTGACCGCCGACAACCCGAGCGAGGCGATGCTGATGGTGGCCAATG
GACAGGCCGACGCCGTCGTGCAGACGCAGATCAGCGCCAGCTATTACGTCAACCGCTACTTCGCCGGCAAGCTGCGCATC
GCCTCGGCGCTGGACCTGCCCCCGGCCGAGATCGCGCTGGCGACGACGCGCGGCCAGACCGAACTGATGTCCATCCTGAA
CAAGGCGCTCTACAGCATTTCGAACGACGAGCTCGCCTCCATCATCAGCCGCTGGCGCGGCAGCGACGGCGATCCGCGCA
CCTGGTACGCCTACCGCAACGAGATCTACCTGCTGATCGGGCTGGGCCTGTTGTCGGCCCTGCTGTTCCTGAGCTGGATC
GTCTACCTGCGGCGCCAGATCCGCCAGCGCAAGCGGGCCGAGCGGGCGCTGAACGACCAGCTGGAATTCATGCGCGTGCT
CATCGACGGCACGCCTAACCCCATCTATGTGCGCGATAAGGAAGGCCGCATGCTGTTGTGCAATGACGCCTACCTCGACA
CCTTTGGCGTGACTGCCGATGCGGTACTGGGCAAGACCATTCCGGAAGCCAACGTGGTGGGCGACCCGGCGCTGGCCCGC
GAAATGCACGAGTTCCTGCTCACGCGCGTGGCCGCCGAGCGCGAGCCGCGCTTCGAGGACCGCGATGTCACGCTGCACGG
CCGCACCCGCCATGTCTACCAGTGGACGATTCCGTACGGCGACTCGCTGGGCGAACTCAAGGGCATCATCGGCGGCTGGA
TCGACATCACCGAACGCGCCGAGCTGCTGCGCAAGCTGCACGACGCCAAGGAAAGCGCCGACGCCGCCAACCGGGCCAAG
ACCACGTTCCTGGCAACGATGAGCCACGAGATCCGCACGCCGATGaACGCGATCATCGGCATGCTGGAGCTGGCGCTGCT
CCGTCCGACCGACCAGGAGCCGGATCGCCAGTCCATCCAGGTCGCGTACGACTCGGCCCGCAGCCTGCTGGAGCTGATAG
GCGACATCCTGGACATTGCGAAGATCGAGGCGGGAAAATTCGACCTGGCGCCGGTGCGCACGGCGCTGCGCGTCCTGCCC
GAAGGGGCGATCCGCGTCTTCGACGGATTGGCGCGCCAAAAAGGCATAGAGCTGGTATTGAAGACCGACATCGTGGGCGT
CGACGATGTATTGATAGACCCCTTGCGCATGAAGCAAGTGCTCTCGAACCTGGTGGGCAACGCCATCAAGTTCACCACCG
AAGGCCAGGTTGTCCTTGCCGTGACCGCACGCCCCGACGGCGACGCCGCGCACGTGCAGTTCAGCGTGAGCGACACCGGC
TGCGGCATCAGCGAGGCCGACCAACGGCAGCTGTTCAAACCGTTCTCGCAAGTGGGTGGCAGCGCCGAGGCCGGGCCGGC
GCCGGGCACCGGCCTGGGCCTGTCCATCAGCCGGCGCCTCGTCGAATTGATGGGGGGAACGCTGGTCATGCGCAGCGCGC
CAGGGGTGGGCACAACGGTTTCGGTGGACCTGAGGCTGACCATGGTCGAAAAATCCGTGCAGGCCGCGCCGCCCGCTGCG
GCCACTGCGGCCACGCCGTCCAAGCCGCAGGTATCGCTGCGCGTGCTGGTCGTCGATGACCACAAACCCAACCTGATGCT
GCTGCGCCAGCAGCTGGACTACCTGGGCCAGCGTGTCATCGCCGCCGACTCCGGCGAAGCCGCCCTGGCCCTGTGGCGCG
AGCATGCGTTCGACGTCGTGATCACCGATtGCAACATGCCCGGTATCAGCGGCTACGAATTGGCGCGCCGCATACGCGCC
GCCGAGGCCGCGCCCGGTTACGGACGTACGCGGTGCATTCTGTTCGGCTTCACGGCTTCGGCGCAGATGGACGAAGCGCA
GCGCTGCCGCGCCGCCGGCATGGACGACTGCCTGTTCAAGCCGATCGGCGTGGACGCCTTGCGGCAACGCTTGAACGAAG
CCGTGGCACGGGCCGCGCTCCCCACGCCCCCCTCGCCCCAGGCTGCCGCGCCGGCCACGGACGACGCCACCCCGACGGCG
TTCTCGGCCGAGTCGATTCTTGCCTTGACGCAGAACGATGAGGCGCTGATCCGGCAATTGCTCGAAGAAGTGATTCGCAC
CAACCGGGCGGACGTCGACCAATTGCAAAAGCTGCACCAGCAGGCCGATTGGCCGAAGGTCTCGGACATGGCGCACAGGC
TGGCCGGCGGCGCGCGCGTGGTCGATGCCAAGGCCATGATAGACACTGTGCTGGCGCTGGAGAAAAAAGCGCAAGGCCAG
GCTGGCCCCTCACCCGAAATCGACGGCCTGGTACGTACGCTTGCGGCGCAGTCCGCCGCGCTGGAGACGCAACTGCGCGC
CTGGCTGGAGCAACGGCCGCATCAAGATCAGCCCTGA
>Bp_B1443.seq
ATGCCCGCCCCGCACCGCCTGTACCCCCGCAGTCTGATCTGCCTGGCTCAGGCGCTATTGGCATGGGCTTTGCTGGCATG
GGCGCCCGCGCAGGCAAGCCAGGAGCTGACCCTGGTCGGCAAGGCTGCCGTTCCCGACGTCGAGGTCGCGCTCGACGGCG
ACGACTGGCGTTGGCTGGCCCGCAAGCGGGTACTGACGCTGGGTGTGTACGCACCGGACATTCCTCCGTTCGACGTCACC
TATGGCGAACGCTACGAAGGCCTGACGGCCGACTACATGGCGATCATCGCGCACAACCTGGGGATGCAGGCGAAAGTGCT
GCGATACCCCACGCGCGAACAAGCCCTCAGCGCGCTGGAAAGCGGGCAGATCGACCTCATCGGCACCGTCAATGGCACGG
ACGGCCGGCAACAGAGCCTGCGTCTGAGCGTTCCCTACGCCGCCGACCACCCGGTGATCGTCATGCCCATCGGCGCACGC
CACGTTCCAGCCTCGAACCTGGCCGGCCAGCGGCTGGCGGTCGACATCAACTACCTGCCCAAGGAAACGCTCGCACGGGC
CTACCCGCAGGCTACGCTGCATTACTTCCCCTCATCCGAGCAGGCGCTGGCCGCGGTGGCCTATGGGCAGGCCGACGTAT
TCATCGGCGATGCCCTGACCACCTCGCACCTCGTATCGCAAAGCTATTTCAATGACGTTCGCGTAGTCGCCCCGGCCCAT
ATCGCGACGGGCGGAGAATCCTTCGGCGTGCGCGCCGACAACACCCGCCTGCTGCGGGTGGTCAACGCCGTACTCGAAGC
CATTCCGCCTTCCGAACACCGCAGCCTGATCTACCGCTGGGGACTGGGCAGCAGCATTTCGCTCGATTTCGCGCACCCCG
CGTATTCCGCGCGCGAGCAGCAATGGATGGCAGACCACCCCGTCGTCAAGGTGGCGGTCCTGAATCTGTTCGCGCCCTTC
ACCCTGTTCCGCACCGACGAACAGTTCGGCGGGATCAGCGCCGCCGTGCTGCAGCTGCTGCAATTGCGCACCGGCCTGGA
CTTCGAGATCATCGGCGTCGACACGGTCGAGGAACTGATAGCCAAGCTGCGTTCGGGCGAAGCCGACATGGCCGGCGCCC
TGTTCGTCAACAGCGCGCGGGAGTCCTTCCTCAGTTTCAGCCGGCCGTATGTGCGCAATGGCATGGTGATCGTCACGCGC
CAGGACCCCGACGCGCCCGTCGACGCCGATCATCTGGACGGCCGCACGGTCGCGTTGGTGCGCAACAGCGCCGCCATTCC
CCTGCTGCAGCGGCGCTATCCCCAGGCGAAGGTGGTGACCGCCGACAACCCGAGCGAGGCGATGCTGATGGTGGCCAATG
GACAGGCCGACGCCGTCGTGCAGACGCAGATCAGCGCCAGCTATTACGTCAACCGCTACTTCGCCGGCAAGCTGCGCATC
GCCTCGGCGCTGGACCTGCCCCCGGCCGAGATCGCGCTGGCGACGACGCGCGGCCAGACCGAACTGATGTCCATCCTGAA
CAAGGCGCTCTACAGCATTTCGAACGACGAGCTCGCCTCCATCATCAGCCGCTGGCGCGGCAGCGACGGCGATCCGCGCA
CCTGGTACGCCTACCGCAACGAGATCTACCTGCTGATCGGGCTGGGCCTGTTGTCGGCCCTGCTGTTCCTGAGCTGGATC
GTCTACCTGCGGCGCCAGATCCGCCAGCGCAAGCGGGCCGAGCGGGCGCTGAACGACCAGCTGGAATTCATGCGCGTGCT
CATCGACGGCACGCCTAACCCCATCTATGTGCGCGATAAGGAAGGCCGCATGCTGTTGTGCAATGACGCCTACCTCGACA
CCTTTGGCGTGACTGCCGATGCGGTACTGGGCAAGACCATTCCGGAAGCCAACGTGGTGGGCGACCCGGCGCTGGCCCGC
GAAATGCACGAGTTCCTGCTCACGCGCGTGGCCGCCGAGCGCGAGCCGCGCTTCGAGGACCGCGATGTCACGCTGCACGG
CCGCACCCGCCATGTCTACCAGTGGACGATTCCGTACGGCGACTCGCTGGGCGAACTCAAGGGCATCATCGGCGGCTGGA
TCGACATCACCGAACGCGCCGAGCTGCTGCGCGAGCTGCACGACGCCAAGGAAAGCGCCGACGCCGCCAACCGGGCCAAG
ACCACGTTCCTGGCAACGATGAGCCACGAGATCCGCACGCCGATGaACGCGATCATCGGCATGCTGGAGCTGGCGCTGCT
CCGTCCGACCGACCAGGAGCCGGATCGCCAGTCCATCCAGGTCGCGTACGACTCGGCCCGCAGCCTGCTGGAGCTGATAG
GCGACATCCTGGACATTGCGAAGATCGAGGCGGGAAAATTCGACCTGGCGCCGGTGCGCACGGCGCTGCGCGTCCTGCCC
GAAGGGGCGATCCGCGTCTTCGACGGATTGGCGCGCCAAAAAGGCATAGAGCTGGTATTGAAGACCGACATCGTGGGCGT
CGACGATGTATTGATAGACCCCTTGCGCATGAAGCAAGTGCTCTCGAACCTGGTGGGCAACGCCATCAAGTTCACCACCG
AAGGCCAGGTTGTCCTTGCCGTGACCGCACGCCCCGACGGCGACGCCGCGCACGTGCAGTTCAGCGTGAGCGACACCGGC
TGCGGCATCAGCGAGGCCGACCAACGGCAGCTGTTCAAACCGTTCTCGCAAGTGGGTGGCAGCGCCGAGGCCGGGCCGGC
GCCGGGCACCGGCCTGGGCCTGTCCATCAGCCGGCGCCTCGTCGAATTGATGGGGGGAACGCTGGTCATGCGCAGCGCGC
CAGGGGTGGGCACAACGGTTTCGGTGGACCTGAGGCTGACCATGGTCGAAAAATCCGTGCAGGCCGCGCCGCCCGCTGCG
GCCACTGCGGCCACGCCGTCCAAGCCGCAGGTATCGCTGCGCGTGCTGGTCGTCGATGACCACAAACCCAACCTGATGCT
GCTGCGCCAGCAGCTGGACTACCTGGGCCAGCGTGTCATCGCCGCCGACTCCGGCGAAGCCGCCCTGGCCCTGTGGCGCG
AGCATGCGTTCGACGTCGTGATCACCGATTGCAACATGCCCGGTATCAGCGGCTACGAATTGGCGCGCCGCATACGCGCC
GCCGAGGCCGCGCCCGGTTACGGACGTACGCGGTGCATTCTGTTCGGCTTCACGGCTTCGGCGCAGATGGACGAAGCGCA
GCGCTGCCGCGCCGCCGGCATGGACGACTGCCTGTTCAAGCCGATCGGCGTGGACGCCTTGCGGCAACGCTTGAACGAAG
CCGTGGCACGGGCCGCGCTCCCCACGCCCCCCTCGCCCCAGGCTGCCGCGCCGGCCACGGACGACGCCACCCCGACGGCG
TTCTCGGCCGAGTCGATTCTTGCCTTGACGCAGAACGATGAGGCGCTGATCCGGCAATTGCTCGAAGAAGTGATTCGCAC
CAACCGGGCGGACGTCGACCAATTGCAAAAGCTGCACCAGCAGGCCGATTGGCCGAAGGTCTCGGACATGGCGCACAGGC
TGGCCGGCGGCGCGCGCGTGGTCGATGCCAAGGCCATGATAGACACTGTGCTGGCGCTGGAGAAAAAAGCGCAAGGCCAG
GCTGGCCCCTCACCCGAAATCGACGGCCTGGTACGTACGCTTGCGGCGCAGTCCGCCGCGCTGGAGACGCAACTGCGCGC
CTGGCTGGAGCAACGGCCGCATCAAGATCAGCCCTGA
>Bp_B1412.seq
ATGCCCGCCCCGCACCGCCTGTACCCCCGCAGTCTGATCTGCCTGGCTCAGGCGCTATTGGCATGGGCTTTGCTGGCATG
GGCGCCCGCGCAGGCAAGCCAGGAGCTGACCCTGGTCGGCAAGGCTGCCGTTCCCGACGTCGAGGTCGCGCTCGACGGCG
ACGACTGGCGTTGGCTGGCCCGCAAGCGGGTACTGACGCTGGGTGTGTACGCACCGGACATTCCTCCGTTCGACGTCACC
TATGGCGAACGCTACGAAGGCCTGACGGCCGACTACATGGCGATCATCGCGCACAACCTGGGGATGCAGGCGAAAGTGCT
GCGATACCCCACGCGCGAACAAGCCCTCAGCGCGCTGGAAAGCGGGCAGATCGACCTCATCGGCACCGTCAATGGCACGG
ACGGCCGGCAACAGAGCCTGCGTCTGAGCGTTCCCTACGCCGCCGACCACCCGGTGATCGTCATGCCCATCGGCGCACGC
CACGTTCCAGCCTCGAACCTGGCCGGCCAGCGGCTGGCGGTCGACATCAACTACCTGCCCAAGGAAACGCTCGCACGGGC
CTACCCGCAGGCTACGCTGCATTACTTCCCCTCATCCGAGCAGGCGCTGGCCGCGGTGGCCTATGGGCAGGCCGACGTAT
TCATCGGCGATGCCCTGACCACCTCGCACCTCGTATCGCAAAGCTATTTCAATGACGTTCGCGTAGTCGCCCCGGCCCAT
ATCGCGACGGGCGGAGAATCCTTCGGCGTGCGCGCCGACAACACCCGCCTGCTGCGGGTGGTCAACGCCGTACTCGAAGC
CATTCCGCCTTCCGAACACCGCAGCCTGATCTACCGCTGGGGACTGGGCAGCAGCATTTCGCTCGATTTCGCGCACCCCG
CGTATTCCGCGCGCGAGCAGCAATGGATGGCAGACCACCCCGTCGTCAAGGTGGCGGTCCTGAATCTGTTCGCGCCCTTC
ACCCTGTTCCGCACCGACGAACAGTTCGGCGGGATCAGCGCCGCCGTGCTGCAGCTGCTGCAATTGCGCACCGGCCTGGA
CTTCGAGATCATCGGCGTCGACACGGTCGAGGAACTGATAGCCAAGCTGCGTTCGGGCGAAGCCGACATGGCCGGCGCCC
TGTTCGTCAACAGCGCGCGGGAGTCCTTCCTCAGTTTCAGCCGGCCGTATGTGCGCAATGGCATGGTGATCGTCACGCGC
CAGGACCCCGACGCGCCCGTCGACGCCGATCATCTGGACGGCCGCACGGTCGCGTTGGTGCGCAACAGCGCCGCCATTCC
CCTGCTGCAGCGGCGCTATCCCCAGGCGAAGGTGGTGACCGCCGACAACCCGAGCGAGGCGATGCTGATGGTGGCCAATG
GACAGGCCGACGCCGTCGTGCAGACGCAGATCAGCGCCAGCTATTACGTCAACCGCTACTTCGCCGGCAAGCTGCGCATC
GCCTCGGCGCTGGACCTGCCCCCGGCCGAGATCGCGCTGGCGACGACGCGCGGCCAGACCGAACTGATGTCCATCCTGAA
CAAGGCGCTCTACAGCATTTCGAACGACGAGCTCGCCTCCATCATCAGCCGCTGGCGCGGCAGCGACGGCGATCCGCGCA
CCTGGTACGCCTACCGCAACGAGATCTACCTGCTGATCGGGCTGGGCCTGTTGTCGGCCCTGCTGTTCCTGAGCTGGATC
GTCTACCTGCGGCGCCAGATCCGCCAGCGCAAGCGGGCCGAGCGGGCGCTGAACGACCAGCTGGAATTCATGCGCGTGCT
CATCGACGGCACGCCTAACCCCATCTATGTGCGCGATAAGGAAGGCCGCATGCTGTTGTGCAATGACGCCTACCTCGACA
CCTTTGGCGTGACTGCCGATGCGGTACTGGGCAAGACCATTCCGGAAGCCAACGTGGTGGGCGACCCGGCGCTGGCCCGC
GAAATGCACGAGTTCCTGCTCACGCGCGTGGCCGCCGAGCGCGAGCCGCGCTTCGAGGACCGCGATGTCACGCTGCACGG
CCGCACCCGCCATGTCTACCAGTGGACGATTCCGTACGGCGACTCGCTGGGCGAACTCAAGGGCATCATCGGCGGCTGGA
TCGACATCACCGAACGCGCCGAGCTGCTGCGCGAGCTGCACGACGCCAAGGAAAGCGCCGACGCCGCCAACCGGGCCAAG
ACCACGTTCCTGGCAACGATGAGCCACGAGATCCGCACGCCGATGAACGCGATCATCGGCATGCTGGAGCTGGCGCTGCT
CCGTCCGACCGACCAGGAGCCGGATCGCCAGTCCATCCAGGTCGCGTACGACTCGGCCCGCAGCCTGCTGGAGCTGATAG
GCGACATCCTGGACATTGCGAAGATCGAGGCGGGAAAATTCGACCTGGCGCCGGTGCGCACGGCGCTGCGCGTCCTGCCC
GAAGGGGCGATCCGCGTCTTCGACGGATTGGCGCGCCAAAAAGGCATAGAGCTGGTATTGAAGACCGACATCGTGGGCGT
CGACGATGTATTGATAGACCCCTTGCGCATGAAGCAAGTGCTCTCGAACCTGGTGGGCAACGCCATCAAGTTCACCACCG
AAGGCCAGGTTGTCCTTGCCGTGACCGCACGCCCCGACGGCGACGCCGCGCACGTGCAGTTCAGCGTGAGCGACACCGGC
TGCGGCATCAGCGAGGCCGACCAACGGCAGCTGTTCAAACCGTTCTCGCAAGTGGGTGGCAGCGCCGAGGCCGGGCCGGC
GCCGGGCACCGGCCTGGGCCTGTCCATCAGCCGGCGCCTCGTCGAATTGATGGGGGGAACGCTGGTCATGCGCAGCGCGC
CAGGGGTGGGCACAACGGTTTCGGTGGACCTGAGGCTGACCATGGTCGAAAAATCCGTGCAGGCCGCGCCGCCCGCTGCG
GCCACTGCGGCCACGCCGTCCAAGCCGCAGGTATCGCTGCGCGTGCTGGTCGTCGATGACCACAAACCCAACCTGATGCT
GCTGCGCCAGCAGCTGGACTACCTGGGCCAGCGTGTCATCGCCGCCGACTCCGGCGAAGCCGCCCTGGCCCTGTGGCGCG
AGCATGCGTTCGACGTCGTGATCACCGATTGCAACATGCCCGGTATCAGCGGCTACGAATTGGCGCGCCGCATACGCGCC
GCCGAGGCCGCGCCCGGTTACGGACGTACGCGGTGCATTCTGTTCGGCTTCACGGCTTCGGCGCAGATGGACGAAGCGCA
GCGCTGCCGCGCCGCCGGCATGGACGACTGCCTGTTCAAGCCGATCGGCGTGGACGCCTTGCGGCAACGCTTGAACGAAG
CCGTGGCACGGGCCGCGCTCCCCACGCCCCCCTCGCCCCAGGCTGCCGCGCCGGCCACGGACGACGCCACCCCGACGGCG
TTCTCGGCCGAGTCGATTCTTGCCTTGACGCAGAACGATGAGGCGCTGATCCGGCAATTGCTCGAAGAAGTGATTCGCAC
CAACCGGGCGGACGTCGACCAATTGCAAAAGCTGCACCAGCAGGCCGATTGGCCGAAGGTCTCGGACATGGCGCACAGGC
TGGCCGGCGGCGCGCGCGTGGTCGATGCCAAGGCCATGATAGACACTGTGCTGGCGCTGGAGAAAAAAGCGCAAGGCCAG
GCTGGCCCCTCACCCGAAATCGACGGCCTGGTACGTACGCTTGCGGCGCAGTCCGCCGCGCTGGAGACGCAACTGCGCGC
CTGGCTGGAGCAACGGCCGCATCAAGATCAGCCCTGA
>Bp_B1419.seq
ATGCCCGCCCCGCACCGCCTGTACCCCCGCAGTCTGATCTGCCTGGCTCAGGCGCTATTGGCATGGGCTTTGCTGGCATG
GGCGCCCGCGCAGGCAAGCCAGGAGCTGACCCTGGTCGGCAAGGCTGCCGTTCCCGACGTCGAGGTCGCGCTCGACGGCG
ACGACTGGCGTTGGCTGGCCCGCAAGCGGGTACTGACGCTGGGTGTGTACGCACCGGACATTCCTCCGTTCGACGTCACC
TATGGCGAACGCTACGAAGGCCTGACGGCCGACTACATGGCGATCATCGCGCACAACCTGGGGATGCAGGCGAAAGTGCT
GCGATACCCCACGCGCGAACAAGCCCTCAGCGCGCTGGAAAGCGGGCAGATCGACCTCATCGGCACCGTCAATGGCACGG
ACGGCCGGCAACAGAGCCTGCGTCTGAGCGTTCCCTACGCCGCCGACCACCCGGTGATCGTCATGCCCATCGGCGCACGC
CACGTTCCAGCCTCGAACCTGGCCGGCCAGCGGCTGGCGGTCGACATCAACTACCTGCCCAAGGAAACGCTCGCACGGGC
CTACCCGCAGGCTACGCTGCATTACTTCCCCTCATCCGAGCAGGCGCTGGCCGCGGTGGCCTATGGGCAGGCCGACGTAT
TCATCGGCGATGCCCTGACCACCTCGCACCTCGTATCGCAAAGCTATTTCAATGACGTTCGCGTAGTCGCCCCGGCCCAT
ATCGCGACGGGCGGAGAATCCTTCGGCGTGCGCGCCGACAACACCCGCCTGCTGCGGGTGGTCAACGCCGTACTCGAAGC
CATTCCGCCTTCCGAACACCGCAGCCTGATCTACCGCTGGGGACTGGGCAGCAGCATTTCGCTCGATTTCGCGCACCCCG
CGTATTCCGCGCGCGAGCAGCAATGGATGGCAGACCACCCCGTCGTCAAGGTGGCGGTCCTGAATCTGTTCGCGCCCTTC
ACCCTGTTCCGCACCGACGAACAGTTCGGCGGGATCAGCGCCGCCGTGCTGCAGCTGCTGCAATTGCGCACCGGCCTGGA
CTTCGAGATCATCGGCGTCGACACGGTCGAGGAACTGATAGCCAAGCTGCGTTCGGGCGAAGCCGACATGGCCGGCGCCC
TGTTCGTCAACAGCGCGCGGGAGTCCTTCCTCAGTTTCAGCCGGCCGTATGTGCGCAATGGCATGGTGATCGTCACGCGC
CAGGACCCCGACGCGCCCGTCGACGCCGATCATCTGGACGGCCGCACGGTCGCGTTGGTGCGCAACAGCGCCGCCATTCC
CCTGCTGCAGCGGCGCTATCCCCAGGCGAAGGTGGTGACCGCCGACAACCCGAGCGAGGCGATGCTGATGGTGGCCAATG
GACAGGCCGACGCCGTCGTGCAGACGCAGATCAGCGCCAGCTATTACGTCAACCGCTACTTCGCCGGCAAGCTGCGCATC
GCCTCGGCGCTGGACCTGCCCCCGGCCGAGATCGCGCTGGCGACGACGCGCGGCCAGACCGAACTGATGTCCATCCTGAA
CAAGGCGCTCTACAGCATTTCGAACGACGAGCTCGCCTCCATCATCAGCCGCTGGCGCGGCAGCGACGGCGATCCGCGCA
CCTGGTACGCCTACCGCAACGAGATCTACCTGCTGATCGGGCTGGGCCTGTTGTCGGCCCTGCTGTTCCTGAGCTGGATC
GTCTACCTGCGGCGCCAGATCCGCCAGCGCAAGCGGGCCGAGCGGGCGCTGAACGACCAGCTGGAATTCATGCGCGTGCT
CATCGACGGCACGCCTAACCCCATCTATGTGCGCGATAAGGAAGGCCGCATGCTGTTGTGCAATGACGCCTACCTCGACA
CCTTTGGCGTGACTGCCGATGCGGTACTGGGCAAGACCATTCCGGAAGCCAACGTGGTGGGCGACCCGGCGCTGGCCCGC
GAAATGCACGAGTTCCTGCTCACGCGCGTGGCCGCCGAGCGCGAGCCGCGCTTCGAGGACCGCGATGTCACGCTGCACGG
CCGCACCCGCCATGTCTACCAGTGGACGATTCCGTACGGCGACTCGCTGGGCGAACTCAAGGGCATCATCGGCGGCTGGA
TCGACATCACCGAACGCGCCGAGCTGCTGCGCGAGCTGCACGACGCCAAGGAAAGCGCCGACGCCGCCAACCGGGCCAAG
ACCACGTTCCTGGCAACGATGAGCCACGAGATCCGCACGCCGATGAACGCGATCATCGGCATGCTGGAGCTGGCGCTGCT
CCGTCCGACCGACCAGGAGCCGGATCGCCAGTCCATCCAGGTCGCGTACGACTCGGCCCGCAGCCTGCTGGAGCTGATAG
GCGACATCCTGGACATTGCGAAGATCGAGGCGGGAAAATTCGACCTGGCGCCGGTGCGCACGGCGCTGCGCGTCCTGCCC
GAAGGGGCGATCCGCGTCTTCGACGGATTGGCGCGCCAAAAAGGCATAGAGCTGGTATTGAAGACCGACATCGTGGGCGT
CGACGATGTATTGATAGACCCCTTGCGCATGAAGCAAGTGCTCTCGAACCTGGTGGGCAACGCCATCAAGTTCACCACCG
AAGGCCAGGTTGTCCTTGCCGTGACCGCACGCCCCGACGGCGACGCCGCGCACGTGCAGTTCAGCGTGAGCGACACCGGC
TGCGGCATCAGCGAGGCCGACCAACGGCAGCTGTTCAAACCGTTCTCGCAAGTGGGTGGCAGCGCCGAGGCCGGGCCGGC
GCCGGGCACCGGCCTGGGCCTGTCCATCAGCCGGCGCCTCGTCGAATTGATGGGGGGAACGCTGGTCATGCGCAGCGCGC
CAGGGGTGGGCACAACGGTTTCGGTGGACCTGAGGCTGACCATGGTCGAAAAATCCGTGCAGGCCGCGCCGCCCGCTGCG
GCCACTGCGGCCACGCCGTCCAAGCCGCAGGTATCGCTGCGCGTGCTGGTCGTCGATGACCACAAACCCAACCTGATGCT
GCTGCGCCAGCAGCTGGACTACCTGGGCCAGCGTGTCATCGCCGCCGACTCCGGCGAAGCCGCCCTGGCCCTGTGGCGCG
AGCATGCGTTCGACGTCGTGATCACCGATTGCAACATGCCCGGTATCAGCGGCTACGAATTGGCGCGCCGCATACGCGCC
GCCGAGGCCGCGCCCGGTTACGGACGTACGCGGTGCATTCTGTTCGGCTTCACGGCTTCGGCGCAGATGGACGAAGCGCA
GCGCTGCCGCGCCGCCGGCATGGACGACTGCCTGTTCAAGCCGATCGGCGTGGACGCCTTGCGGCAACGCTTGAACGAAG
CCGTGGCACGGGCCGCGCTCCCCACGCCCCCCTCGCCCCAGGCTGCCGCGCCGGCCACGGACGACGCCACCCCGACGGCG
TTCTCGGCCGAGTCGATTCTTGCCTTGACGCAGAACGATGAGGCGCTGATCCGGCAATTGCTCGAAGAAGTGATTCGCAC
CAACCGGGCGGACGTCGACCAATTGCAAAAGCTGCACCAGCAGGCCGATTGGCCGAAGGTCTCGGACATGGCGCACAGGC
TGGCCGGCGGCGCGCGCGTGGTCGATGCCAAGGCCATGATAGACACTGTGCTGGCGCTGGAGAAAAAAGCGCAAGGCCAG
GCTGGCCCCTCACCCGAAATCGACGGCCTGGTACGTACGCTTGCGGCGCAGTCCGCCGCGCTGGAGACGCAACTGCGCGC
CTGGCTGGAGCAACGGCCGCATCAAGATCAGCCCTGA
>Bp_B1422.seq
ATGCCCGCCCCGCACCGCCTGTACCCCCGCAGTCTGATCTGCCTGGCTCAGGCGCTATTGGCATGGGCTTTGCTGGCATG
GGCGCCCGCGCAGGCAAGCCAGGAGCTGACCCTGGTCGGCAAGGCTGCCGTTCCCGACGTCGAGGTCGCGCTCGACGGCG
ACGACTGGCGTTGGCTGGCCCGCAAGCGGGTACTGACGCTGGGTGTGTACGCACCGGACATTCCTCCGTTCGACGTCACC
TATGGCGAACGCTACGAAGGCCTGACGGCCGACTACATGGCGATCATCGCGCACAACCTGGGGATGCAGGCGAAAGTGCT
GCGATACCCCACGCGCGAACAAGCCCTCAGCGCGCTGGAAAGCGGGCAGATCGACCTCATCGGCACCGTCAATGGCACGG
ACGGCCGGCAACAGAGCCTGCGTCTGAGCGTTCCCTACGCCGCCGACCACCCGGTGATCGTCATGCCCATCGGCGCACGC
CACGTTCCAGCCTCGAACCTGGCCGGCCAGCGGCTGGCGGTCGACATCAACTACCTGCCCAAGGAAACGCTCGCACGGGC
CTACCCGCAGGCTACGCTGCATTACTTCCCCTCATCCGAGCAGGCGCTGGCCGCGGTGGCCTATGGGCAGGCCGACGTAT
TCATCGGCGATGCCCTGACCACCTCGCACCTCGTATCGCAAAGCTATTTCAATGACGTTCGCGTAGTCGCCCCGGCCCAT
ATCGCGACGGGCGGAGAATCCTTCGGCGTGCGCGCCGACAACACCCGCCTGCTGCGGGTGGTCAACGCCGTACTCGAAGC
CATTCCGCCTTCCGAACACCGCAGCCTGATCTACCGCTGGGGACTGGGCAGCAGCATTTCGCTCGATTTCGCGCACCCCG
CGTATTCCGCGCGCGAGCAGCAATGGATGGCAGACCACCCCGTCGTCAAGGTGGCGGTCCTGAATCTGTTCGCGCCCTTC
ACCCTGTTCCGCACCGACGAACAGTTCGGCGGGATCAGCGCCGCCGTGCTGCAGCTGCTGCAATTGCGCACCGGCCTGGA
CTTCGAGATCATCGGCGTCGACACGGTCGAGGAACTGATAGCCAAGCTGCGTTCGGGCGAAGCCGACATGGCCGGCGCCC
TGTTCGTCAACAGCGCGCGGGAGTCCTTCCTCAGTTTCAGCCGGCCGTATGTGCGCAATGGCATGGTGATCGTCACGCGC
CAGGACCCCGACGCGCCCGTCGACGCCGATCATCTGGACGGCCGCACGGTCGCGTTGGTGCGCAACAGCGCCGCCATTCC
CCTGCTGCAGCGGCGCTATCCCCAGGCGAAGGTGGTGACCGCCGACAACCCGAGCGAGGCGATGCTGATGGTGGCCAATG
GACAGGCCGACGCCGTCGTGCAGACGCAGATCAGCGCCAGCTATTACGTCAACCGCTACTTCGCCGGCAAGCTGCGCATC
GCCTCGGCGCTGGACCTGCCCCCGGCCGAGATCGCGCTGGCGACGACGCGCGGCCAGACCGAACTGATGTCCATCCTGAA
CAAGGCGCTCTACAGCATTTCGAACGACGAGCTCGCCTCCATCATCAGCCGCTGGCGCGGCAGCGACGGCGATCCGCGCA
CCTGGTACGCCTACCGCAACGAGATCTACCTGCTGATCGGGCTGGGCCTGTTGTCGGCCCTGCTGTTCCTGAGCTGGATC
GTCTACCTGCGGCGCCAGATCCGCCAGCGCAAGCGGGCCGAGCGGGCGCTGAACGACCAGCTGGAATTCATGCGCGTGCT
CATCGACGGCACGCCTAACCCCATCTATGTGCGCGATAAGGAAGGCCGCATGCTGTTGTGCAATGACGCCTACCTCGACA
CCTTTGGCGTGACTGCCGATGCGGTACTGGGCAAGACCATTCCGGAAGCCAACGTGGTGGGCGACCCGGCGCTGGCCCGC
GAAATGCACGAGTTCCTGCTCACGCGCGTGGCCGCCGAGCGCGAGCCGCGCTTCGAGGACCGCGATGTCACGCTGCACGG
CCGCACCCGCCATGTCTACCAGTGGACGATTCCGTACGGCGACTCGCTGGGCGAACTCAAGGGCATCATCGGCGGCTGGA
TCGACATCACCGAACGCGCCGAGCTGCTGCGCGAGCTGCACGACGCCAAGGAAAGCGCCGACGCCGCCAACCGGGCCAAG
ACCACGTTCCTGGCAACGATGAGCCACGAGATCCGCACGCCGATGaACGCGATCATCGGCATGCTGGAGCTGGCGCTGCT
CCGTCCGACCGACCAGGAGCCGGATCGCCAGTCCATCCAGGTCGCGTACGACTCGGCCCGCAGCCTGCTGGAGCTGATAG
GCGACATCCTGGACATTGCGAAGATCGAGGCGGGAAAATTCGACCTGGCGCCGGTGCGCACGGCGCTGCGCGTCCTGCCC
GAAGGGGCGATCCGCGTCTTCGACGGATTGGCGCGCCAAAAAGGCATAGAGCTGGTATTGAAGACCGACATCGTGGGCGT
CGACGATGTATTGATAGACCCCTTGCGCATGAAGCAAGTGCTCTCGAACCTGGTGGGCAACGCCATCAAGTTCACCACCG
AAGGCCAGGTTGTCCTTGCCGTGACCGCACGCCCCGACGGCGACGCCGCGCACGTGCAGTTCAGCGTGAGCGACACCGGC
TGCGGCATCAGCGAGGCCGACCAACGGCAGCTGTTCAAACCGTTCTCGCAAGTGGGTGGCAGCGCCGAGGCCGGGCCGGC
GCCGGGCACCGGCCTGGGCCTGTCCATCAGCCGGCGCCTCGTCGAATTGATGGGGGGAACGCTGGTCATGCGCAGCGCGC
CAGGGGTGGGCACAACGGTTTCGGTGGACCTGAGGCTGACCATGGTCGAAAAATCCGTGCAGGCCGCGCCGCCCGCTGCG
GCCACTGCGGCCACGCCGTCCAAGCCGCAGGTATCGCTGCGCGTGCTGGTCGTCGATGACCACAAACCCAACCTGATGCT
GCTGCGCCAGCAGCTGGACTACCTGGGCCAGCGTGTCATCGCCGCCGACTCCGGCGAAGCCGCCCTGGCCCTGTGGCGCG
AGCATGCGTTCGACGTCGTGATCACCGATTGCAACATGCCCGGTATCAGCGGCTACGAATTGGCGCGCCGCATACGCGCC
GCCGAGGCCGCGCCCGGTTACGGACGTACGCGGTGCATTCTGTTCGGCTTCACGGCTTCGGCGCAGATGGACGAAGCGCA
GCGCTGCCGCGCCGCCGGCATGGACGACTGCCTGTTCAAGCCGATCGGCGTGGACGCCTTGCGGCAACGCTTGAACGAAG
CCGTGGCACGGGCCGCGCTCCCCACGCCCCCCTCGCCCCAGGCTGCCGCGCCGGCCACGGACGACGCCACCCCGACGGCG
TTCTCGGCCGAGTCGATTCTTGCCTTGACGCAGAACGATGAGGCGCTGATCCGGCAATTGCTCGAAGAAGTGATTCGCAC
CAACCGGGCGGACGTCGACCAATTGCAAAAGCTGCACCAGCAGGCCGATTGGCCGAAGGTCTCGGACATGGCGCACAGGC
TGGCCGGCGGCGCGCGCGTGGTCGATGCCAAGGCCATGATAGACACTGTGCTGGCGCTGGAGAAAAAAGCGCAAGGCCAG
GCTGGCCCCTCACCCGAAATCGACGGCCTGGTACGTACGCTTGCGGCGCAGTCCGCCGCGCTGGAGACGCAACTGCGCGC
CTGGCTGGAGCAACGGCCGCATCAAGATCAGCCCTGA
>Bp_B1424.seq
ATGCCCGCCCCGCACCGCCTGTACCCCCGCAGTCTGATCTGCCTGGCTCAGGCGCTATTGGCATGGGCTTTGCTGGCATG
GGCGCCCGCGCAGGCAAGCCAGGAGCTGACCCTGGTCGGCAAGGCTGCCGTTCCCGACGTCGAGGTCGCGCTCGACGGCG
ACGACTGGCGTTGGCTGGCCCGCAAGCGGGTACTGACGCTGGGTGTGTACGCACCGGACATTCCTCCGTTCGACGTCACC
TATGGCGAACGCTACGAAGGCCTGACGGCCGACTACATGGCGATCATCGCGCACAACCTGGGGATGCAGGCGAAAGTGCT
GCGATACCCCACGCGCGAACAAGCCCTCAGCGCGCTGGAAAGCGGGCAGATCGACCTCATCGGCACCGTCAATGGCACGG
ACGGCCGGCAACAGAGCCTGCGTCTGAGCGTTCCCTACGCCGCCGACCACCCGGTGATCGTCATGCCCATCGGCGCACGC
CACGTTCCAGCCTCGAACCTGGCCGGCCAGCGGCTGGCGGTCGACATCAACTACCTGCCCAAGGAAACGCTCGCACGGGC
CTACCCGCAGGCTACGCTGCATTACTTCCCCTCATCCGAGCAGGCGCTGGCCGCGGTGGCCTATGGGCAGGCCGACGTAT
TCATCGGCGATGCCCTGACCACCTCGCACCTCGTATCGCAAAGCTATTTCAATGACGTTCGCGTAGTCGCCCCGGCCCAT
ATCGCGACGGGCGGAGAATCCTTCGGCGTGCGCGCCGACAACACCCGCCTGCTGCGGGTGGTCAACGCCGTACTCGAAGC
CATTCCGCCTTCCGAACACCGCAGCCTGATCTACCGCTGGGGACTGGGCAGCAGCATTTCGCTCGATTTCGCGCACCCCG
CGTATTCCGCGCGCGAGCAGCAATGGATGGCAGACCACCCCGTCGTCAAGGTGGCGGTCCTGAATCTGTTCGCGCCCTTC
ACCCTGTTCCGCACCGACGAACAGTTCGGCGGGATCAGCGCCGCCGTGCTGCAGCTGCTGCAATTGCGCACCGGCCTGGA
CTTCGAGATCATCGGCGTCGACACGGTCGAGGAACTGATAGCCAAGCTGCGTTCGGGCGAAGCCGACATGGCCGGCGCCC
TGTTCGTCAACAGCGCGCGGGAGTCCTTCCTCAGTTTCAGCCGGCCGTATGTGCGCAATGGCATGGTGATCGTCACGCGC
CAGGACCCCGACGCGCCCGTCGACGCCGATCATCTGGACGGCCGCACGGTCGCGTTGGTGCGCAACAGCGCCGCCATTCC
CCTGCTGCAGCGGCGCTATCCCCAGGCGAAGGTGGTGACCGCCGACAACCCGAGCGAGGCGATGCTGATGGTGGCCAATG
GACAGGCCGACGCCGTCGTGCAGACGCAGATCAGCGCCAGCTATTACGTCAACCGCTACTTCGCCGGCAAGCTGCGCATC
GCCTCGGCGCTGGACCTGCCCCCGGCCGAGATCGCGCTGGCGACGACGCGCGGCCAGACCGAACTGATGTCCATCCTGAA
CAAGGCGCTCTACAGCATTTCGAACGACGAGCTCGCCTCCATCATCAGCCGCTGGCGCGGCAGCGACGGCGATCCGCGCA
CCTGGTACGCCTACCGCAACGAGATCTACCTGCTGATCGGGCTGGGCCTGTTGTCGGCCCTGCTGTTCCTGAGCTGGATC
GTCTACCTGCGGCGCCAGATCCGCCAGCGCAAGCGGGCCGAGCGGGCGCTGAACGACCAGCTGGAATTCATGCGCGTGCT
CATCGACGGCACGCCTAACCCCATCTATGTGCGCGATAAGGAAGGCCGCATGCTGTTGTGCAATGACGCCTACCTCGACA
CCTTTGGCGTGACTGCCGATGCGGTACTGGGCAAGACCATTCCGGAAGCCAACGTGGTGGGCGACCCGGCGCTGGCCCGC
GAAATGCACGAGTTCCTGCTCACGCGCGTGGCCGCCGAGCGCGAGCCGCGCTTCGAGGACCGCGATGTCACGCTGCACGG
CCGCACCCGCCATGTCTACCAGTGGACGATTCCGTACGGCGACTCGCTGGGCGAACTCAAGGGCATCATCGGCGGCTGGA
TCGACATCACCGAACGCGCCGAGCTGCTGCGCGAGCTGCACGACGCCAAGGAAAGCGCCGACGCCGCCAACCGGGCCAAG
ACCACGTTCCTGGCAACGATGAGCCACGAGATCCGCACGCCGATGaACGCGATCATCGGCATGCTGGAGCTGGCGCTGCT
CCGTCCGACCGACCAGGAGCCGGATCGCCAGTCCATCCAGGTCGCGTACGACTCGGCCCGCAGCCTGCTGGAGCTGATAG
GCGACATCCTGGACATTGCGAAGATCGAGGCGGGAAAATTCGACCTGGCGCCGGTGCGCACGGCGCTGCGCGTCCTGCCC
GAAGGGGCGATCCGCGTCTTCGACGGATTGGCGCGCCAAAAAGGCATAGAGCTGGTATTGAAGACCGACATCGTGGGCGT
CGACGATGTATTGATAGACCCCTTGCGCATGAAGCAAGTGCTCTCGAACCTGGTGGGCAACGCCATCAAGTTCACCACCG
AAGGCCAGGTTGTCCTTGCCGTGACCGCACGCCCCGACGGCGACGCCGCGCACGTGCAGTTCAGCGTGAGCGACACCGGC
TGCGGCATCAGCGAGGCCGACCAACGGCAGCTGTTCAAACCGTTCTCGCAAGTGGGTGGCAGCGCCGAGGCCGGGCCGGC
GCCGGGCACCGGCCTGGGCCTGTCCATCAGCCGGCGCCTCGTCGAATTGATGGGGGGAACGCTGGTCATGCGCAGCGCGC
CAGGGGTGGGCACAACGGTTTCGGTGGACCTGAGGCTGACCATGGTCGAAAAATCCGTGCAGGCCGCGCCGCCCGCTGCG
GCCACTGCGGCCACGCCGTCCAAGCCGCAGGTATCGCTGCGCGTGCTGGTCGTCGATGACCACAAACCCAACCTGATGCT
GCTGCGCCAGCAGCTGGACTACCTGGGCCAGCGTGTCATCGCCGCCGACTCCGGCGAAGCCGCCCTGGCCCTGTGGCGCG
AGCATGCGTTCGACGTCGTGATCACCGATtGCAACATGCCCGGTATCAGCGGCTACGAATTGGCGCGCCGCATACGCGCC
GCCGAGGCCGCGCCCGGTTACGGACGTACGCGGTGCATTCTGTTCGGCTTCACGGCTTCGGCGCAGATGGACGAAGCGCA
GCGCTGCCGCGCCGCCGGCATGGACGACTGCCTGTTCAAGCCGATCGGCGTGGACGCCTTGCGGCAACGCTTGAACGAAG
CCGTGGCACGGGCCGCGCTCCCCACGCCCCCCTCGCCCCAGGCTGCCGCGCCGGCCACGGACGACGCCACCCCGACGGCG
TTCTCGGCCGAGTCGATTCTTGCCTTGACGCAGAACGATGAGGCGCTGATCCGGCAATTGCTCGAAGAAGTGATTCGCAC
CAACCGGGCGGACGTCGACCAATTGCAAAAGCTGCACCAGCAGGCCGATTGGCCGAAGGTCTCGGACATGGCGCACAGGC
TGGCCGGCGGCGCGCGCGTGGTCGATGCCAAGGCCATGATAGACACTGTGCTGGCGCTGGAGAAAAAAGCGCAAGGCCAG
GCTGGCCCCTCACCCGAAATCGACGGCCTGGTACGTACGCTTGCGGCGCAGTCCGCCGCGCTGGAGACGCAACTGCGCGC
CTGGCTGGAGCAACGGCCGCATCAAGATCAGCCCTGA
>Bp_B1617.seq
ATGCCCGCCCCGCACCGCCTGTACCCCCGCAGTCTGATCTGCCTGGCTCAGGCGCTATTGGCATGGGCTTTGCTGGCATG
GGCGCCCGCGCAGGCAAGCCAGGAGCTGACCCTGGTCGGCAAGGCTGCCGTTCCCGACGTCGAGGTCGCGCTCGACGGCG
ACGACTGGCGTTGGCTGGCCCGCAAGCGGGTACTGACGCTGGGTGTGTACGCACCGGACATTCCTCCGTTCGACGTCACC
TATGGCGAACGCTACGAAGGCCTGACGGCCGACTACATGGCGATCATCGCGCACAACCTGGGGATGCAGGCGAAAGTGCT
GCGATACCCCACGCGCGAACAAGCCCTCAGCGCGCTGGAAAGCGGGCAGATCGACCTCATCGGCACCGTCAATGGCACGG
ACGGCCGGCAACAGAGCCTGCGTCTGAGCGTTCCCTACGCCGCCGACCACCCGGTGATCGTCATGCCCATCGGCGCACGC
CACGTTCCAGCCTCGAACCTGGCCGGCCAGCGGCTGGCGGTCGACATCAACTACCTGCCCAAGGAAACGCTCGCACGGGC
CTACCCGCAGGCTACGCTGCATTACTTCCCCTCATCCGAGCAGGCGCTGGCCGCGGTGGCCTATGGGCAGGCCGACGTAT
TCATCGGCGATGCCCTGACCACCTCGCACCTCGTATCGCAAAGCTATTTCAATGACGTTCGCGTAGTCGCCCCGGCCCAT
ATCGCGACGGGCGGAGAATCCTTCGGCGTGCGCGCCGACAACACCCGCCTGCTGCGGGTGGTCAACGCCGTACTCGAAGC
CATTCCGCCTTCCGAACACCGCAGCCTGATCTACCGCTGGGGACTGGGCAGCAGCATTTCGCTCGATTTCGCGCACCCCG
CGTATTCCGCGCGCGAGCAGCAATGGATGGCAGACCACCCCGTCGTCAAGGTGGCGGTCCTGAATCTGTTCGCGCCCTTC
ACCCTGTTCCGCACCGACGAACAGTTCGGCGGGATCAGCGCCGCCGTGCTGCAGCTGCTGCAATTGCGCACCGGCCTGGA
CTTCGAGATCATCGGCGTCGACACGGTCGAGGAACTGATAGCCAAGCTGCGTTCGGGCGAAGCCGACATGGCCGGCGCCC
TGTTCGTCAACAGCGCGCGGGAGTCCTTCCTCAGTTTCAGCCGGCCGTATGTGCGCAATGGCATGGTGATCGTCACGCGC
CAGGACCCCGACGCGCCCGTCGACGCCGATCATCTGGACGGCCGCACGGTCGCGTTGGTGCGCAACAGCGCCGCCATTCC
CCTGCTGCAGCGGCGCTATCCCCAGGCGAAGGTGGTGACCGCCGACAACCCGAGCGAGGCGATGCTGATGGTGGCCAATG
GACAGGCCGACGCCGTCGTGCAGACGCAGATCAGCGCCAGCTATTACGTCAACCGCTACTTCGCCGGCAAGCTGCGCATC
GCCTCGGCGCTGGACCTGCCCCCGGCCGAGATCGCGCTGGCGACGACGCGCGGCCAGACCGAACTGATGTCCATCCTGAA
CAAGGCGCTCTACAGCATTTCGAACGACGAGCTCGCCTCCATCATCAGCCGCTGGCGCGGCAGCGACGGCGATCCGCGCA
CCTGGTACGCCTACCGCAACGAGATCTACCTGCTGATCGGGCTGGGCCTGTTGTCGGCCCTGCTGTTCCTGAGCTGGATC
GTCTACCTGCGGCGCCAGATCCGCCAGCGCAAGCGGGCCGAGCGGGCGCTGAACGACCAGCTGGAATTCATGCGCGTGCT
CATCGACGGCACGCCTAACCCCATCTATGTGCGCGATAAGGAAGGCCGCATGCTGTTGTGCAATGACGCCTACCTCGACA
CCTTTGGCGTGACTGCCGATGCGGTACTGGGCAAGACCATTCCGGAAGCCAACGTGGTGGGCGACCCGGCGCTGGCCCGC
GAAATGCACGAGTTCCTGCTCACGCGCGTGGCCGCCGAGCGCGAGCCGCGCTTCGAGGACCGCGATGTCACGCTGCACGG
CCGCACCCGCCATGTCTACCAGTGGACGATTCCGTACGGCGACTCGCTGGGCGAACTCAAGGGCATCATCGGCGGCTGGA
TCGACATCACCGAACGCGCCGAGCTGCTGCGCGAGCTGCACGACGCCAAGGAAAGCGCCGACGCCGCCAACCGGGCCAAG
ACCACGTTCCTGGCAACGATGAGCCACGAGATCCGCACGCCGATGAACGCGATCATCGGCATGCTGGAGCTGGCGCTGCT
CCGTCCGACCGACCAGGAGCCGGATCGCCAGTCCATCCAGGTCGCGTACGACTCGGCCCGCAGCCTGCTGGAGCTGATAG
GCGACATCCTGGACATTGCGAAGATCGAGGCGGGAAAATTCGACCTGGCGCCGGTGCGCACGGCGCTGCGCGTCCTGCCC
GAAGGGGCGATCCGCGTCTTCGACGGATTGGCGCGCCAAAAAGGCATAGAGCTGGTATTGAAGACCGACATCGTGGGCGT
CGACGATGTATTGATAGACCCCTTGCGCATGAAGCAAGTGCTCTCGAACCTGGTGGGCAACGCCATCAAGTTCACCACCG
AAGGCCAGGTTGTCCTTGCCGTGACCGCACGCCCCGACGGCGACGCCGCGCACGTGCAGTTCAGCGTGAGCGACACCGGC
TGCGGCATCAGCGAGGCCGACCAACGGCAGCTGTTCAAACCGTTCTCGCAAGTGGGTGGCAGCGCCGAGGCCGGGCCGGC
GCCGGGCACCGGCCTGGGCCTGTCCATCAGCCGGCGCCTCGTCGAATTGATGGGGGGAACGCTGGTCATGCGCAGCGCGC
CAGGGGTGGGCACAACGGTTTCGGTGGACCTGAGGCTGACCATGGTCGAAAAATCCGTGCAGGCCGCGCCGCCCGCTGCG
GCCACTGCGGCCACGCCGTCCAAGCCGCAGGTATCGCTGCGCGTGCTGGTCGTCGATGACCACAAACCCAACCTGATGCT
GCTGCGCCAGCAGCTGGACTACCTGGGCCAGCGTGTCATCGCCGCCGACTCCGGCGAAGCCGCCCTGGCCCTGTGGCGCG
AGCATGCGTTCGACGTCGTGATCACCGATTGCAACATGCCCGGTATCAGCGGCTACGAATTGGCGCGCCGCATACGCGCC
GCCGAGGCCGCGCCCGGTTACGGACGTACGCGGTGCATTCTGTTCGGCTTCACGGCTTCGGCGCAGATGGACGAAGCGCA
GCGCTGCCGCGCCGCCGGCATGGACGACTGCCTGTTCAAGCCGATCGGCGTGGACGCCTTGCGGCAACGCTTGAACGAAG
CCGTGGCACGGGCCGCGCTCCCCACGCCCCCCTCGCCCCAGGCTGCCGCGCCGGCCACGGACGACGCCACCCCGACGGCG
TTCTCGGCCGAGTCGATTCTTGCCTTGACGCAGAACGATGAGGCGCTGATCCGGCAATTGCTCGAAGAAGTGATTCGCAC
CAACCGGGCGGACGTCGACCAATTGCAAAAGCTGCACCAGCAGGCCGATTGGCCGAAGGTCTCGGACATGGCGCACAGGC
TGGCCGGCGGCGCGCGCGTGGTCGATGCCAAGGCCATGATAGACACTGTGCTGGCGCTGGAGAAAAAAGCGCAAGGCCAG
GCTGGCCCCTCACCCGAAATCGACGGCCTGGTACGTACGCTTGCGGCGCAGTCCGCCGCGCTGGAGACGCAACTGCGCGC
CTGGCTGGAGCAACGGCCGCATCAAGATCAGCCCTGA
>Bp_B1834.seq
ATGCCCGCCCCGCACCGCCTGTACCCCCGCAGTCTGATCTGCCTGGCTCAGGCGCTATTGGCATGGGCTTTGCTGGCATG
GGCGCCCGCGCAGGCAAGCCAGGAGCTGACCCTGGTCGGCAAGGCTGCCGTTCCCGACGTCGAGGTCGCGCTCGACGGCG
ACGACTGGCGTTGGCTGGCCCGCAAGCGGGTACTGACGCTGGGTGTGTACGCACCGGACATTCCTCCGTTCGACGTCACC
TATGGCGAACGCTACGAAGGCCTGACGGCCGACTACATGGCGATCATCGCGCACAACCTGGGGATGCAGGCGAAAGTGCT
GCGATACCCCACGCGCGAACAAGCCCTCAGCGCGCTGGAAAGCGGGCAGATCGACCTCATCGGCACCGTCAATGGCACGG
ACGGCCGGCAACAGAGCCTGCGTCTGAGCGTTCCCTACGCCGCCGACCACCCGGTGATCGTCATGCCCATCGGCGCACGC
CACGTTCCAGCCTCGAACCTGGCCGGCCAGCGGCTGGCGGTCGACATCAACTACCTGCCCAAGGAAACGCTCGCACGGGC
CTACCCGCAGGCTACGCTGCATTACTTCCCCTCATCCGAGCAGGCGCTGGCCGCGGTGGCCTATGGGCAGGCCGACGTAT
TCATCGGCGATGCCCTGACCACCTCGCACCTCGTATCGCAAAGCTATTTCAATGACGTTCGCGTAGTCGCCCCGGCCCAT
ATCGCGACGGGCGGAGAATCCTTCGGCGTGCGCGCCGACAACACCCGCCTGCTGCGGGTGGTCAACGCCGTACTCGAAGC
CATTCCGCCTTCCGAACACCGCAGCCTGATCTACCGCTGGGGACTGGGCAGCAGCATTTCGCTCGATTTCGCGCACCCCG
CGTATTCCGCGCGCGAGCAGCAATGGATGGCAGACCACCCCGTCGTCAAGGTGGCGGTCCTGAATCTGTTCGCGCCCTTC
ACCCTGTTCCGCACCGACGAACAGTTCGGCGGGATCAGCGCCGCCGTGCTGCAGCTGCTGCAATTGCGCACCGGCCTGGA
CTTCGAGATCATCGGCGTCGACACGGTCGAGGAACTGATAGCCAAGCTGCGTTCGGGCGAAGCCGACATGGCCGGCGCCC
TGTTCGTCAACAGCGCGCGGGAGTCCTTCCTCAGTTTCAGCCGGCCGTATGTGCGCAATGGCATGGTGATCGTCACGCGC
CAGGACCCCGACGCGCCCGTCGACGCCGATCATCTGGACGGCCGCACGGTCGCGTTGGTGCGCAACAGCGCCGCCATTCC
CCTGCTGCAGCGGCGCTATCCCCAGGCGAAGGTGGTGACCGCCGACAACCCGAGCGAGGCGATGCTGATGGTGGCCAATG
GACAGGCCGACGCCGTCGTGCAGACGCAGATCAGCGCCAGCTATTACGTCAACCGCTACTTCGCCGGCAAGCTGCGCATC
GCCTCGGCGCTGGACCTGCCCCCGGCCGAGATCGCGCTGGCGACGACGCGCGGCCAGACCGAACTGATGTCCATCCTGAA
CAAGGCGCTCTACAGCATTTCGAACGACGAGCTCGCCTCCATCATCAGCCGCTGGCGCGGCAGCGACGGCGATCCGCGCA
CCTGGTACGCCTACCGCAACGAGATCTACCTGCTGATCGGGCTGGGCCTGTTGTCGGCCCTGCTGTTCCTGAGCTGGATC
GTCTACCTGCGGCGCCAGATCCGCCAGCGCAAGCGGGCCGAGCGGGCGCTGAACGACCAGCTGGAATTCATGCGCGTGCT
CATCGACGGCACGCCTAACCCCATCTATGTGCGCGATAAGGAAGGCCGCATGCTGTTGTGCAATGACGCCTACCTCGACA
CCTTTGGCGTGACTGCCGATGCGGTACTGGGCAAGACCATTCCGGAAGCCAACGTGGTGGGCGACCCGGCGCTGGCCCGC
GAAATGCACGAGTTCCTGCTCACGCGCGTGGCCGCCGAGCGCGAGCCGCGCTTCGAGGACCGCGATGTCACGCTGCACGG
CCGCACCCGCCATGTCTACCAGTGGACGATTCCGTACGGCGACTCGCTGGGCGAACTCAAGGGCATCATCGGCGGCTGGA
TCGACATCACCGAACGCGCCGAGCTGCTGCGCGAGCTGCACGACGCCAAGGAAAGCGCCGACGCCGCCAACCGGGCCAAG
ACCACGTTCCTGGCAACGATGAGCCACGAGATCCGCACGCCGATGaACGCGATCATCGGCATGCTGGAGCTGGCGCTGCT
CCGTCCGACCGACCAGGAGCCGGATCGCCAGTCCATCCAGGTCGCGTACGACTCGGCCCGCAGCCTGCTGGAGCTGATAG
GCGACATCCTGGACATTGCGAAGATCGAGGCGGGAAAATTCGACCTGGCGCCGGTGCGCACGGCGCTGCGCGTCCTGCCC
GAAGGGGCGATCCGCGTCTTCGACGGATTGGCGCGCCAAAAAGGCATAGAGCTGGTATTGAAGACCGACATCGTGGGCGT
CGACGATGTATTGATAGACCCCTTGCGCATGAAGCAAGTGCTCTCGAACCTGGTGGGCAACGCCATCAAGTTCACCACCG
AAGGCCAGGTTGTCCTTGCCGTGACCGCACGCCCCGACGGCGACGCCGCGCACGTGCAGTTCAGCGTGAGCGACACCGGC
TGCGGCATCAGCGAGGCCGACCAACGGCAGCTGTTCAAACCGTTCTCGCAAGTGGGTGGCAGCGCCGAGGCCGGGCCGGC
GCCGGGCACCGGCCTGGGCCTGTCCATCAGCCGGCGCCTCGTCGAATTGATGGGGGGAACGCTGGTCATGCGCAGCGCGC
CAGGGGTGGGCACAACGGTTTCGGTGGACCTGAGGCTGACCATGGTCGAAAAATCCGTGCAGGCCGCGCCGCCCGCTGCG
GCCACTGCGGCCACGCCGTCCAAGCCGCAGGTATCGCTGCGCGTGCTGGTCGTCGATGACCACAAACCCAACCTGATGCT
GCTGCGCCAGCAGCTGGACTACCTGGGCCAGCGTGTCATCGCCGCCGACTCCGGCGAAGCCGCCCTGGCCCTGTGGCGCG
AGCATGCGTTCGACGTCGTGATCACCGATTGCAACATGCCCGGTATCAGCGGCTACGAATtGGCGCGCCGCATACGCGCC
GCCGAGGCCGCGCCCGGTTACGGACGTACGCGGTGCATTCTGTTCGGCTTCACGGCTTCGGCGCAGATGGACGAAGCGCA
GCGCTGCCGCGCCGCCGGCATGGACGACTGCCTGTTCAAGCCGATCGGCGTGGACGCCTTGCGGCAACGCTTGAACGAAG
CCGTGGCACGGGCCGCGCTCCCCACGCCCCCCTCGCCCCAGGCTGCCGCGCCGGCCACGGACGACGCCACCCCGACGGCG
TTCTCGGCCGAGTCGATTCTTGCCTTGACGCAGAACGATGAGGCGCTGATCCGGCAATTGCTCGAAGAAGTGATTCGCAC
CAACCGGGCGGACGTCGACCAATTGCAAAAGCTGCACCAGCAGGCCGATTGGCCGAAGGTCTCGGACATGGCGCACAGGC
TGGCCGGCGGCGCGCGCGTGGTCGATGCCAAGGCCATGATAGACACTGTGCTGGCGCTGGAGAAAAAAGCGCAAGGCCAG
GCTGGCCCCTCACCCGAAATCGACGGCCTGGTACGTACGCTTGCGGCGCAGTCCGCCGCGCTGGAGACGCAACTGCGCGC
CTGGCTGGAGCAACGGCCGCATCAAGATCAGCCCTGA
>Bp_B1836.seq
ATGCCCGCCCCGCACCGCCTGTACCCCCGCAGTCTGATCTGCCTGGCTCAGGCGCTATTGGCATGGGCTTTGCTGGCATG
GGCGCCCGCGCAGGCAAGCCAGGAGCTGACCCTGGTCGGCAAGGCTGCCGTTCCCGACGTCGAGGTCGCGCTCGACGGCG
ACGACTGGCGTTGGCTGGCCCGCAAGCGGGTACTGACGCTGGGTGTGTACGCACCGGACATTCCTCCGTTCGACGTCACC
TATGGCGAACGCTACGAAGGCCTGACGGCCGACTACATGGCGATCATCGCGCACAACCTGGGGATGCAGGCGAAAGTGCT
GCGATACCCCACGCGCGAACAAGCCCTCAGCGCGCTGGAAAGCGGGCAGATCGACCTCATCGGCACCGTCAATGGCACGG
ACGGCCGGCAACAGAGCCTGCGTCTGAGCGTTCCCTACGCCGCCGACCACCCGGTGATCGTCATGCCCATCGGCGCACGC
CACGTTCCAGCCTCGAACCTGGCCGGCCAGCGGCTGGCGGTCGACATCAACTACCTGCCCAAGGAAACGCTCGCACGGGC
CTACCCGCAGGCTACGCTGCATTACTTCCCCTCATCCGAGCAGGCGCTGGCCGCGGTGGCCTATGGGCAGGCCGACGTAT
TCATCGGCGATGCCCTGACCACCTCGCACCTCGTATCGCAAAGCTATTTCAATGACGTTCGCGTAGTCGCCCCGGCCCAT
ATCGCGACGGGCGGAGAATCCTTCGGCGTGCGCGCCGACAACACCCGCCTGCTGCGGGTGGTCAACGCCGTACTCGAAGC
CATTCCGCCTTCCGAACACCGCAGCCTGATCTACCGCTGGGGACTGGGCAGCAGCATTTCGCTCGATTTCGCGCACCCCG
CGTATTCCGCGCGCGAGCAGCAATGGATGGCAGACCACCCCGTCGTCAAGGTGGCGGTCCTGAATCTGTTCGCGCCCTTC
ACCCTGTTCCGCACCGACGAACAGTTCGGCGGGATCAGCGCCGCCGTGCTGCAGCTGCTGCAATTGCGCACCGGCCTGGA
CTTCGAGATCATCGGCGTCGACACGGTCGAGGAACTGATAGCCAAGCTGCGTTCGGGCGAAGCCGACATGGCCGGCGCCC
TGTTCGTCAACAGCGCGCGGGAGTCCTTCCTCAGTTTCAGCCGGCCGTATGTGCGCAATGGCATGGTGATCGTCACGCGC
CAGGACCCCGACGCGCCCGTCGACGCCGATCATCTGGACGGCCGCACGGTCGCGTTGGTGCGCAACAGCGCCGCCATTCC
CCTGCTGCAGCGGCGCTATCCCCAGGCGAAGGTGGTGACCGCCGACAACCCGAGCGAGGCGATGCTGATGGTGGCCAATG
GACAGGCCGACGCCGTCGTGCAGACGCAGATCAGCGCCAGCTATTACGTCAACCGCTACTTCGCCGGCAAGCTGCGCATC
GCCTCGGCGCTGGACCTGCCCCCGGCCGAGATCGCGCTGGCGACGACGCGCGGCCAGACCGAACTGATGTCCATCCTGAA
CAAGGCGCTCTACAGCATTTCGAACGACGAGCTCGCCTCCATCATCAGCCGCTGGCGCGGCAGCGACGGCGATCCGCGCA
CCTGGTACGCCTACCGCAACGAGATCTACCTGCTGATCGGGCTGGGCCTGTTGTCGGCCCTGCTGTTCCTGAGCTGGATC
GTCTACCTGCGGCGCCAGATCCGCCAGCGCAAGCGGGCCGAGCGGGCGCTGAACGACCAGCTGGAATTCATGCGCGTGCT
CATCGACGGCACGCCTAACCCCATCTATGTGCGCGATAAGGAAGGCCGCATGCTGTTGTGCAATGACGCCTACCTCGACA
CCTTTGGCGTGACTGCCGATGCGGTACTGGGCAAGACCATTCCGGAAGCCAACGTGGTGGGCGACCCGGCGCTGGCCCGC
GAAATGCACGAGTTCCTGCTCACGCGCGTGGCCGCCGAGCGCGAGCCGCGCTTCGAGGACCGCGATGTCACGCTGCACGG
CCGCACCCGCCATGTCTACCAGTGGACGATTCCGTACGGCGACTCGCTGGGCGAACTCAAGGGCATCATCGGCGGCTGGA
TCGACATCACCGAACGCGCCGAGCTGCTGCGCGAGCTGCACGACGCCAAGGAAAGCGCCGACGCCGCCAACCGGGCCAAG
ACCACGTTCCTGGCAACGATGAGCCACGAGATCCGCACGCCGATGaACGCGATCATCGGCATGCTGGAGCTGGCGCTGCT
CCGTCCGACCGACCAGGAGCCGGATCGCCAGTCCATCCAGGTCGCGTACGACTCGGCCCGCAGCCTGCTGGAGCTGATAG
GCGACATCCTGGACATTGCGAAGATCGAGGCGGGAAAATTCGACCTGGCGCCGGTGCGCACGGCGCTGCGCGTCCTGCCC
GAAGGGGCGATCCGCGTCTTCGACGGATTGGCGCGCCAAAAAGGCATAGAGCTGGTATTGAAGACCGACATCGTGGGCGT
CGACGATGTATTGATAGACCCCTTGCGCATGAAGCAAGTGCTCTCGAACCTGGTGGGCAACGCCATCAAGTTCACCACCG
AAGGCCAGGTTGTCCTTGCCGTGACCGCACGCCCCGACGGCGACGCCGCGCACGTGCAGTTCAGCGTGAGCGACACCGGC
TGCGGCATCAGCGAGGCCGACCAACGGCAGCTGTTCAAACCGTTCTCGCAAGTGGGTGGCAGCGCCGAGGCCGGGCCGGC
GCCGGGCACCGGCCTGGGCCTGTCCATCAGCCGGCGCCTCGTCGAATTGATGGGGGGAACGCTGGTCATGCGCAGCGCGC
CAGGGGTGGGCACAACGGTTTCGGTGGACCTGAGGCTGACCATGGTCGAAAAATCCGTGCAGGCCGCGCCGCCCGCTGCG
GCCACTGCGGCCACGCCGTCCAAGCCGCAGGTATCGCTGCGCGTGCTGGTCGTCGATGACCACAAACCCAACCTGATGCT
GCTGCGCCAGCAGCTGGACTACCTGGGCCAGCGTGTCATCGCCGCCGACTCCGGCGAAGCCGCCCTGGCCCTGTGGCGCG
AGCATGCGTTCGACGTCGTGATCACCGATTGCAACATGCCCGGTATCAGCGGCTACGAATtGGCGCGCCGCATACGCGCC
GCCGAGGCCGCGCCCGGTTACGGACGTACGCGGTGCATTCTGTTCGGCTTCACGGCTTCGGCGCAGATGGACGAAGCGCA
GCGCTGCCGCGCCGCCGGCATGGACGACTGCCTGTTCAAGCCGATCGGCGTGGACGCCTTGCGGCAACGCTTGAACGAAG
CCGTGGCACGGGCCGCGCTCCCCACGCCCCCCTCGCCCCAGGCTGCCGCGCCGGCCACGGACGACGCCACCCCGACGGCG
TTCTCGGCCGAGTCGATTCTTGCCTTGACGCAGAACGATGAGGCGCTGATCCGGCAATTGCTCGAAGAAGTGATTCGCAC
CAACCGGGCGGACGTCGACCAATTGCAAAAGCTGCACCAGCAGGCCGATTGGCCGAAGGTCTCGGACATGGCGCACAGGC
TGGCCGGCGGCGCGCGCGTGGTCGATGCCAAGGCCATGATAGACACTGTGCTGGCGCTGGAGAAAAAAGCGCAAGGCCAG
GCTGGCCCCTCACCCGAAATCGACGGCCTGGTACGTACGCTTGCGGCGCAGTCCGCCGCGCTGGAGACGCAACTGCGCGC
CTGGCTGGAGCAACGGCCGCATCAAGATCAGCCCTGA
>Bp_B1917.seq
ATGCCCGCCCCGCACCGCCTGTACCCCCGCAGTCTGATCTGCCTGGCTCAGGCGCTATTGGCATGGGCTTTGCTGGCATG
GGCGCCCGCGCAGGCAAGCCAGGAGCTGACCCTGGTCGGCAAGGCTGCCGTTCCCGACGTCGAGGTCGCGCTCGACGGCG
ACGACTGGCGTTGGCTGGCCCGCAAGCGGGTACTGACGCTGGGTGTGTACGCACCGGACATTCCTCCGTTCGACGTCACC
TATGGCGAACGCTACGAAGGCCTGACGGCCGACTACATGGCGATCATCGCGCACAACCTGGGGATGCAGGCGAAAGTGCT
GCGATACCCCACGCGCGAACAAGCCCTCAGCGCGCTGGAAAGCGGGCAGATCGACCTCATCGGCACCGTCAATGGCACGG
ACGGCCGGCAACAGAGCCTGCGTCTGAGCGTTCCCTACGCCGCCGACCACCCGGTGATCGTCATGCCCATCGGCGCACGC
CACGTTCCAGCCTCGAACCTGGCCGGCCAGCGGCTGGCGGTCGACATCAACTACCTGCCCAAGGAAACGCTCGCACGGGC
CTACCCGCAGGCTACGCTGCATTACTTCCCCTCATCCGAGCAGGCGCTGGCCGCGGTGGCCTATGGGCAGGCCGACGTAT
TCATCGGCGATGCCCTGACCACCTCGCACCTCGTATCGCAaAGCTATTTCAATGACGTTCGCGTAGTCGCCCCGGCCCAT
ATCGCGACGGGCGGAGAATCCTTCGGCGTGCGCGCCGACAACACCCGCCTGCTGCGGGTGGTCAACGCCGTACTCGAAGC
CATTCCGCCTTCCGAACACCGCAGCCTGATCTACCGCTGGGGACTGGGCAGCAGCATTTCGCTCGATTTCGCGCACCCCG
CGTATTCCGCGCGCGAGCAGCAATGGATGGCAGACCACCCCGTCGTCAAGGTGGCGGTCCTGAATCTGTTCGCGCCCTTC
ACCCTGTTCCGCACCGACGAACAGTTCGGCGGGATCAGCGCCGCCGTGCTGCAGCTGCTGCAATTGCGCACCGGCCTGGA
CTTCGAGATCATCGGCGTCGACACGGTCGAGGAACTGATAGCCAAGCTGCGTTCGGGCGAAGCCGACATGGCCGGCGCCC
TGTTCGTCAACAGCGCGCGGGAGTCCTTCCTCAGTTTCAGCCGGCCGTATGTGCGCAATGGCATGGTGATCGTCACGCGC
CAGGACCCCGACGCGCCCGTCGACGCCGATCATCTGGACGGCCGCACGGTCGCGTTGGTGCGCAACAGCGCCGCCATTCC
CCTGCTGCAGCGGCGCTATCCCCAGGCGAAGGTGGTGACCGCCGACAACCCGAGCGAGGCGATGCTGATGGTGGCCAATG
GACAGGCCGACGCCGTCGTGCAGACGCAGATCAGCGCCAGCTATTACGTCAACCGCTACTTCGCCGGCAAGCTGCGCATC
GCCTCGGCGCTGGACCTGCCCCCGGCCGAGATCGCGCTGGCGACGACGCGCGGCCAGACCGAACTGATGTCCATCCTGAA
CAAGGCGCTCTACAGCATTTCGAACGACGAGCTCGCCTCCATCATCAGCCGCTGGCGCGGCAGCGACGGCGATCCGCGCA
CCTGGTACGCCTACCGCAACGAGATCTACCTGCTGATCGGGCTGGGCCTGTTGTCGGCCCTGCTGTTCCTGAGCTGGATC
GTCTACCTGCGGCGCCAGATCCGCCAGCGCAAGCGGGCCGAGCGGGCGCTGAACGACCAGCTGGAATTCATGCGCGTGCT
CATCGACGGCACGCCTAACCCCATCTATGTGCGCGATAAGGAAGGCCGCATGCTGTTGTGCAATGACGCCTACCTCGACA
CCTTTGGCGTGACTGCCGATGCGGTACTGGGCAAGACCATTCCGGAAGCCAACGTGGTGGGCGACCCGGCGCTGGCCCGC
GAAATGCACGAGTTCCTGCTCACGCGCGTGGCCGCCGAGCGCGAGCCGCGCTTCGAGGACCGCGATGTCACGCTGCACGG
CCGCACCCGCCATGTCTACCAGTGGACGATTCCGTACGGCGACTCGCTGGGCGAACTCAAGGGCATCATCGGCGGCTGGA
TCGACATCACCGAACGCGCCGAGCTGCTGCGCGAGCTGCACGACGCCAAGGAAAGCGCCGACGCCGCCAACCGGGCCAAG
ACCACGTTCCTGGCAACGATGAGCCACGAGATCCGCACGCCGATGaACGCGATCATCGGCATGCTGGAGCTGGCGCTGCT
CCGTCCGACCGACCAGGAGCCGGATCGCCAGTCCATCCAGGTCGCGTACGACTCGGCCCGCAGCCTGCTGGAGCTGATAG
GCGACATCCTGGACATTGCGAAGATCGAGGCGGGAAAATTCGACCTGGCGCCGGTGCGCACGGCGCTGCGCGTCCTGCCC
GAAGGGGCGATCCGCGTCTTCGACGGATTGGCGCGCCAAAAAGGCATAGAGCTGGTATTGAAGACCGACATCGTGGGCGT
CGACGATGTATTGATAGACCCCTTGCGCATGAAGCAAGTGCTCTCGAACCTGGTGGGCAACGCCATCAAGTTCACCACCG
AAGGCCAGGTTGTCCTTGCCGTGACCGCACGCCCCGACGGCGACGCCGCGCACGTGCAGTTCAGCGTGAGCGACACCGGC
TGCGGCATCAGCGAGGCCGACCAACGGCAGCTGTTCAAACCGTTCTCGCAAGTGGGTGGCAGCGCCGAGGCCGGGCCGGC
GCCGGGCACCGGCCTGGGCCTGTCCATCAGCCGGCGCCTCGTCGAATTGATGGGGGGAACGCTGGTCATGCGCAGCGCGC
CAGGGGTGGGCACAACGGTTTCGGTGGACCTGAGGCTGACCATGGTCGAAAAATCCGTGCAGGCCGCGCCGCCCGCTGCG
GCCACTGCGGCCACGCCGTCCAAGCCGCAGGTATCGCTGCGCGTGCTGGTCGTCGATGACCACAAACCCAACCTGATGCT
GCTGCGCCAGCAGCTGGACTACCTGGGCCAGCGTGTCATCGCCGCCGACTCCGGCGAAGCCGCCCTGGCCCTGTGGCGCG
AGCATGCGTTCGACGTCGTGATCACCGATTGCAACATGCCCGGTATCAGCGGCTACGAATtGGCGCGCCGCATACGCGCC
GCCGAGGCCGCGCCCGGTTACGGACGTACGCGGTGCATTCTGTTCGGCTTCACGGCTTCGGCGCAGATGGACGAAGCGCA
GCGCTGCCGCGCCGCCGGCATGGACGACTGCCTGTTCAAGCCGATCGGCGTGGACGCCTTGCGGCAACGCTTGAACGAAG
CCGTGGCACGGGCCGCGCTCCCCACGCCCCCCTCGCCCCAGGCTGCCGCGCCGGCCACGGACGACGCCACCCCGACGGCG
TTCTCGGCCGAGTCGATTCTTGCCTTGACGCAGAACGATGAGGCGCTGATCCGGCAATTGCTCGAAGAAGTGATTCGCAC
CAACCGGGCGGACGTCGACCAATTGCAAAAGCTGCACCAGCAGGCCGATTGGCCGAAGGTCTCGGACATGGCGCACAGGC
TGGCCGGCGGCGCGCGCGTGGTCGATGCCAAGGCCATGATAGACACTGTGCTGGCGCTGGAGAAAAAAGCGCAAGGCCAG
GCTGGCCCCTCACCCGAAATCGACGGCCTGGTACGTACGCTTGCGGCGCAGTCCGCCGCGCTGGAGACGCAACTGCGCGC
CTGGCTGGAGCAACGGCCGCATCAAGATCAGCCCTGA
>Bp_B1981.seq
ATGCCCGCCCCGCACCGCCTGTACCCCCGCAGTCTGATCTGCCTGGCTCAGGCGCTATTGGCATGGGCTTTGCTGGCATG
GGCGCCCGCGCAGGCAAGCCAGGAGCTGACCCTGGTCGGCAAGGCTGCCGTTCCCGACGTCGAGGTCGCGCTCGACGGCG
ACGACTGGCGTTGGCTGGCCCGCAAGCGGGTACTGACGCTGGGTGTGTACGCACCGGACATTCCTCCGTTCGACGTCACC
TATGGCGAACGCTACGAAGGCCTGACGGCCGACTACATGGCGATCATCGCGCACAACCTGGGGATGCAGGCGAAAGTGCT
GCGATACCCCACGCGCGAACAAGCCCTCAGCGCGCTGGAAAGCGGGCAGATCGACCTCATCGGCACCGTCAATGGCACGG
ACGGCCGGCAACAGAGCCTGCGTCTGAGCGTTCCCTACGCCGCCGACCACCCGGTGATCGTCATGCCCATCGGCGCACGC
CACGTTCCAGCCTCGAACCTGGCCGGCCAGCGGCTGGCGGTCGACATCAACTACCTGCCCAAGGAAACGCTCGCACGGGC
CTACCCGCAGGCTACGCTGCATTACTTCCCCTCATCCGAGCAGGCGCTGGCCGCGGTGGCCTATGGGCAGGCCGACGTAT
TCATCGGCGATGCCCTGACCACCTCGCACCTCGTATCGCAAAGCTATTTCAATGACGTTCGCGTAGTCGCCCCGGCCCAT
ATCGCGACGGGCGGAGAATCCTTCGGCGTGCGCGCCGACAACACCCGCCTGCTGCGGGTGGTCAACGCCGTACTCGAAGC
CATTCCGCCTTCCGAACACCGCAGCCTGATCTACCGCTGGGGACTGGGCAGCAGCATTTCGCTCGATTTCGCGCACCCCG
CGTATTCCGCGCGCGAGCAGCAATGGATGGCAGACCACCCCGTCGTCAAGGTGGCGGTCCTGAATCTGTTCGCGCCCTTC
ACCCTGTTCCGCACCGACGAACAGTTCGGCGGGATCAGCGCCGCCGTGCTGCAGCTGCTGCAATTGCGCACCGGCCTGGA
CTTCGAGATCATCGGCGTCGACACGGTCGAGGAACTGATAGCCAAGCTGCGTTCGGGCGAAGCCGACATGGCCGGCGCCC
TGTTCGTCAACAGCGCGCGGGAGTCCTTCCTCAGTTTCAGCCGGCCGTATGTGCGCAATGGCATGGTGATCGTCACGCGC
CAGGACCCCGACGCGCCCGTCGACGCCGATCATCTGGACGGCCGCACGGTCGCGTTGGTGCGCAACAGCGCCGCCATTCC
CCTGCTGCAGCGGCGCTATCCCCAGGCGAAGGTGGTGACCGCCGACAACCCGAGCGAGGCGATGCTGATGGTGGCCAATG
GACAGGCCGACGCCGTCGTGCAGACGCAGATCAGCGCCAGCTATTACGTCAACCGCTACTTCGCCGGCAAGCTGCGCATC
GCCTCGGCGCTGGACCTGCCCCCGGCCGAGATCGCGCTGGCGACGACGCGCGGCCAGACCGAACTGATGTCCATCCTGAA
CAAGGCGCTCTACAGCATTTCGAACGACGAGCTCGCCTCCATCATCAGCCGCTGGCGCGGCAGCGACGGCGATCCGCGCA
CCTGGTACGCCTACCGCAACGAGATCTACCTGCTGATCGGGCTGGGCCTGTTGTCGGCCCTGCTGTTCCTGAGCTGGATC
GTCTACCTGCGGCGCCAGATCCGCCAGCGCAAGCGGGCCGAGCGGGCGCTGAACGACCAGCTGGAATTCATGCGCGTGCT
CATCGACGGCACGCCTAACCCCATCTATGTGCGCGATAAGGAAGGCCGCATGCTGTTGTGCAATGACGCCTACCTCGACA
CCTTTGGCGTGACTGCCGATGCGGTACTGGGCAAGACCATTCCGGAAGCCAACGTGGTGGGCGACCCGGCGCTGGCCCGC
GAAATGCACGAGTTCCTGCTCACGCGCGTGGCCGCCGAGCGCGAGCCGCGCTTCGAGGACCGCGATGTCACGCTGCACGG
CCGCACCCGCCATGTCTACCAGTGGACGATTCCGTACGGCGACTCGCTGGGCGAACTCAAGGGCATCATCGGCGGCTGGA
TCGACATCACCGAACGCGCCGAGCTGCTGCGCGAGCTGCACGACGCCAAGGAAAGCGCCGACGCCGCCAACCGGGCCAAG
ACCACGTTCCTGGCAACGATGAGCCACGAGATCCGCACGCCGATGaACGCGATCATCGGCATGCTGGAGCTGGCGCTGCT
CCGTCCGACCGACCAGGAGCCGGATCGCCAGTCCATCCAGGTCGCGTACGACTCGGCCCGCAGCCTGCTGGAGCTGATAG
GCGACATCCTGGACATTGCGAAGATCGAGGCGGGAAAATTCGACCTGGCGCCGGTGCGCACGGCGCTGCGCGTCCTGCCC
GAAGGGGCGATCCGCGTCTTCGACGGATTGGCGCGCCAAAAAGGCATAGAGCTGGTATTGAAGACCGACATCGTGGGCGT
CGACGATGTATTGATAGACCCCTTGCGCATGAAGCAAGTGCTCTCGAACCTGGTGGGCAACGCCATCAAGTTCACCACCG
AAGGCCAGGTTGTCCTTGCCGTGACCGCACGCCCCGACGGCGACGCCGCGCACGTGCAGTTCAGCGTGAGCGACACCGGC
TGCGGCATCAGCGAGGCCGACCAACGGCAGCTGTTCAAACCGTTCTCGCAAGTGGGTGGCAGCGCCGAGGCCGGGCCGGC
GCCGGGCACCGGCCTGGGCCTGTCCATCAGCCGGCGCCTCGTCGAATTGATGGGGGGAACGCTGGTCATGCGCAGCGCGC
CAGGGGTGGGCACAACGGTTTCGGTGGACCTGAGGCTGACCATGGTCGAAAAATCCGTGCAGGCCGCGCCGCCCGCTGCG
GCCACTGCGGCCACGCCGTCCAAGCCGCAGGTATCGCTGCGCGTGCTGGTCGTCGATGACCACAAACCCAACCTGATGCT
GCTGCGCCAGCAGCTGGACTACCTGGGCCAGCGTGTCATCGCCGCCGACTCCGGCGAAGCCGCCCTGGcCCTGTGGCGCG
AGCATGCGTTCGACGTCGTGATCACCGATTGCAACATGCCCGGTATCAGCGGCTACGAATtGGCGCGCCGCATACGCGCC
GCCGAGGCCGCGCCCGGTTACGGACGTACGCGGTGCATTCTGTTCGGCTTCACGGCTTCGGCGCAGATGGACGAAGCGCA
GCGCTGCCGCGCCGCCGGCATGGACGACTGCCTGTTCAAGCCGATCGGCGTGGACGCCTTGCGGCAACGCTTGAACGAAG
CCGTGGCACGGGCCGCGCTCCCCACGCCCCCCTCGCCCCAGGCTGCCGCGCCGGCCACGGACGACGCCACCCCGACGGCG
TTCTCGGCCGAGTCGATTCTTGCCTTGACGCAGAACGATGAGGCGCTGATCCGGCAATTGCTCGAAGAAGTGATTCGCAC
CAACCGGGCGGACGTCGACCAATTGCAAAAGCTGCACCAGCAGGCCGATTGGCCGAAGGTCTCGGACATGGCGCACAGGC
TGGCCGGCGGCGCGCGCGTGGTCGATGCCAAGGCCATGATAGACACTGTGCTGGCGCTGGAGAAAAAAGCGCAAGGCCAG
GCTGGCCCCTCACCCGAAATCGACGGCCTGGTACGTACGCTTGCGGCGCAGTCCGCCGCGCTGGAGACGCAACTGCGCGC
CTGGCTGGAGCAACGGCCGCATCAAGATCAGCCCTGA
>Bpp_12822
atgcccgccccgcaccgcctgtacccccgcagtctgatctgcctggctcaggcgctattggtatgggctttgctggcatg
ggcgcccgcgcaggcaagccaggagctgaccctggtcggcaaggctgccgttcccgacgtcgagatcgcgctcgacggcg
acgactggcgctggctggcgcgcaagcgggtgctgacgctgggcgtgtacgcgccggacattcccccgttcgacgtcacc
tatgacgagcgctacgaaggcctgacggccgactatatggcgatcatcgcgcacaacctgggcgtccaggcaaaagtgct
gcgctaccccacgcgcgagcaagccgtcggcgcactggaaagcggacagatcgacctcatcggcaccgtcaatggcatcg
agggccggctgcagagcctgcgcctgagtgttccctacgcggccgaccacccggtgctggtcatgcccatcggcgcgcgc
cgcgctccgcccgcggacctggcaggccagcggttggcggttgacgccaactacttgcccagggaaacgctgcagcaggc
ctatccccaggcaacgctgcattacttcccatcgtccgagcaggcgctggccgcggtggcctatggacaagccgacgtgt
tcatcggcgatgcgctgaccacctcgcacctcgtttcgcaaagctacttcaacgacgttcgcgtcgtcgccccggcccag
atcgtgacgggcggggaatccttcggcgtgcgcgccgacaatacccgcctgctgcgggtggtcaatgccgtgctcgaagc
cattccggcctccgagcgccgcagcctgatctaccgctggggcctgggcagcagcatttcgctcgatttcgcgcgccccg
cctattcagcgcgcgagcagcagtggatggcaaaccatccggtcgtcaaggtggcggtcctgaacctgttcgcgcccttc
accctgtttcgcaccgatgaacagttcggcggcatcagtgccgccgtgctgcaactgctgcagttgcgcaccggcctgga
tttccagatcatcggcgtcgacacggtcgaggagctgatcgccaagctgcgctcgggcgaagccgacatggccggcgccc
tgttcgtcaatgccgcgcgggaatccgtcctcagcttcagccggccgtatgtgcgcaatggcatggtgatcgtcacgcgc
caggaccccgccgcgcccgccgacgccgatcacctcgacggccgcacgattgcgatggtgcgcaacagcgccgccatccc
gctcctgcagcagcgctatccccaggcgaaggtcgtgaccgccgacaacccgaccgaagccatgctgctggtggccgatg
gccaggccgacgccgtcgtgcagacgcagatcagcgccagctactacgtcaaccgctacttcgccggaaaactgcgcatt
gcctcggcgctggacctgccgccggccgagatcgcgctggcgacggcgcgcggccagaccgagctgatatccatcctgaa
caaggcgctctacagcatttcgaacgacgaactcgcctccatcgtcagccgctggcgcggcagcgacggcgatccgcgca
cctggtacgcctaccgcaacgagatctacctgctgatcgggctgggcctgttgtcggccctgctgttcctgagctggatc
gtctacctgcggcgccagatccgccagcgcaagcgggccgagcgggcgctgaacgaccagctggaattcatgcgcgtgct
catcgacggcacgcccaaccccatctatgtgcgcgataaggaaggccgcatgctgttgtgcaatgacgcctacctcgaca
cctttggcgtgactgccgatgcggtactgggcaagaccatcccggaggccaacgtggtgggcgacccggcgctggctcgc
gagatgcacgagttcctgctcacgcgcatggccgccgagcgcgagccgcgcttcgaggaccgcgatgtcacgctgcacgg
ccgcacccgccatgtctaccagtggacggttccgtacggcgactcgctgggcgaactcaagggcatcatcggcggctgga
tcgacattaccgaacgcgccgagctgctgcgcgagctgcacgacgccaaggaaagcgccgacgccgccaaccgggccaag
accacgttcctggcaacgatgagccacgagatccgcacgccgatgaacgcgatcatcggcatgctggagctggcgctgct
ccgtccggccgaccaggagccggaccgccagtccatccaggtcgcgtacgactcggcccgcagcctgctggagctgatag
gcgacatcctggacattgcgaagatcgaggcgggaaaattcgacctggcgccggtgcgcacggcgctgcgcgccctgccc
gaaggggcgatccgcctcttcgatggattggcgcgccagaaaggcatagagctggtattgaagaccgacatcgtgggcgt
ggacgatgtattgatagaccccttgcgcatgaagcaagtgctctcgaacctggtgggcaacgccatcaagttcaccaccg
aaggccaggttgtccttaccgtaaccgcgcgccccgacggcgaggccgcgcacgtgcagttcagcgtgagcgacaccggc
tgcggcatcagcgaggccgaccaacggcagctgttcaaaccgttctcgcaggtgggtggcagcgccgaggccgggccggc
gccgggcactggcctgggcctgtccatcagccgccgcctcgtcgaattgatggggggaacgctggtcatgcgcagcgcgc
caggcgtgggcacaacggtttcggtggacctgaggctgaccatgatcgaaaaatccgcgcaggccacgccgcccgctgcg
gccgctcaggccacgccgtccaagccgcaggtatcgctgcgcgtgctggtcgtcgatgaccacaagcccaacctgatgct
gctgcgccagcagctggactacctgggccagcgtgtcgtcgccgccgactccggcgaagccgccctggccctgtggcacg
agcatgcgttcgacgtcgtgatcaccgattgcaacatgcccggtatcaacggctacgaattggcgcgccgcatacgcgcc
gccgaggccgcgcccggttacggacgtacgcggtgcattctgttcggcttcacggcttcggcgcagatggacgaagcgca
gcgctgccgcgccgccggcatggacgactgcctgttcaagccgatcggcgtggacgccttgcggcaacgcttgaacgaag
ccgcggcacgggccgcgctccccacgcccccctcgccccaggctgccgcgccggccacgcacgacgccaccccggcggcg
ttctcggccgagtcgattcttgccctgacgcagaacgacgaggcgctgatccggcaattgctcgaagaagtgattcgcac
caaccgggcggacgtcgatcaattgcagaagctgcaccagcaggccgattggccgaaggtctcggacatggcgcacaggc
tggccggcggcgcgcgcgtggtcgatgccaaggccatgatagacactgcgctggcgctggagaaaaaagcgcaaggccag
gctggcccctcgcccgaaatcgacggcctggtacgtacgcttgcggcgcagtccgccgcgctggagacgcaactacgcgc
ctggctggagcaacggccgcatcaaggccagccctga
>Bpp_B0024.seq
ATGCCCGCCCCGCACCGCCTGTACCCCCGCAGTCTGATCTGCCTGGCTCAGGCGCTATTGGTATGGGCTTTGCTGGCATG
GGCGCCCGCGCAGGCAAGCCAGGAGCTGACCCTGGTCGGCAAGGCTGCCGTTCCCGACGTCGAGATCGCGCTCGACGGCG
ACGACTGGCGCTGGCTGGCGCGCAAGCGGGTGCTGACGCTGGGCGTGTACGCGCCGGACATTCCCCCGTTCGACGTCACC
TATGACGAGCGCTACGAAGGCCTGACGGCCGACTATATGGCGATCATCGCGCACAACCTGGGCGTCCAGGCAAAAGTGCT
GCGCTACCCCACGCGCGAGCAAGCCGTCGGCGCACTGGAAAGCGGACAGATCGACCTCATCGGCACCGTCAATGGCATCG
AGGGCCGGCTGCAGAGCCTGCGCCTGAGTGTTCCCTACGCGGCCGACCACCCGGTGCTGGTCATGCCCATCGGCGCGCGC
CGCGCTCCGCCCGCGGACCTGGCAGGCCAGCGGTTGGCGGTTGACGCCAACTACTTGCCCAGGGAAACGCTGCAGCAGGC
CTATCCCCAGGCAACGCTGCATTACTTCCCATCGTCCGAGCAGGCGCTGGCCGCGGTGGCCTATGGACAAGCCGACGTGT
TCATCGGCGATGCGCTGACCACCTCGCACCTCGTTTCGCAAAGCTACTTCAACGACGTTCGCGTCGTCGCCCCGGCCCAG
ATCGTGACGGGCGGGGAATCCTTCGGCGTGCGCGCCGACAATACCCGCCTGCTGCGGGTGGTCAATGCCGTGCTCGAAGC
CATTCCGGCCTCCGAGCGCCGCAGCCTGATCTACCGCTGGGGCCTGGGCAGCAGCATTTCGCTCGATTTCGCGCGCCCCG
CCTATTCAGCGCGCGAGCAGCAGTGGATGGCAAACCATCCGGTCGTCAAGGTGGCGGTCCTGAACCTGTTCGCGCCCTTC
ACCCTGTTTCGCACCGATGAACAGTTCGGCGGCATCAGTGCCGCCGTGCTGCAACTGCTGCAGTTGCGCACCGGCCTGGA
TTTCCAGATCATCGGCGTCGACACGGTCGAGGAGCTGATCGCCAAGCTGCGCTCGGGCGAAGCCGACATGGCCGGCGCCC
TGTTCGTCAATGCCGCGCGGGAATCCGTCCTCAGCTTCAGCCGGCCGTATGTGCGCAATGGCATGGTGATCGTCACGCGC
CAGGACCCCGCCGCGCCCGCCGACGCCGATCACCTCGACGGCCGCACGATTGCGATGGTGCGCAACAGCGCCGCCATCCC
GCTCCTGCAGCAGCGCTATCCCCAGGCGAAGGTCGTGACCGCCGACAACCCGACCGAAGCCATGCTGCTGGTGGCCGATG
GCCAGGCCGACGCCGTCGTGCAGACGCAGATCAGCGCCAGCTACTACGTCAACCGCTACTTCGCCGGAAAACTGCGCATT
GCCTCGGCGCTGGACCTGCCGCCGGCCGAGATCGCGCTGGCGACGGCGCGCGGCCAGACCGAGCTGATATCCATCCTGAA
CAAGGCGCTCTACAGCATTTCGAACGACGAACTCGCCTCCATCGTCAGCCGCTGGCGCGGCAGCGACGGCGATCCGCGCA
CCTGGTACGCCTACCGCAACGAGATCTACCTGCTGATCGGGCTGGGCCTGTTGTCGGCCCTGCTGTTCCTGAGCTGGATC
GTCTACCTGCGGCGCCAGATCCGCCAGCGCAAGCGGGCCGAGCGGGCGCTGAACGACCAGCTGGAATTCATGCGCGTGCT
CATCGACGGCACGCCCAACCCCATCTATGTGCGCGATAAGGAAGGCCGCATGCTGTTGTGCAATGACGCCTACCTCGACA
CCTTTGGCGTGACTGCCGATGCGGTACTGGGCAAGACCATCCCGGAGGCCAACGTGGTGGGCGACCCGGCGCTGGCTCGC
GAGATGCACGAGTTCCTGCTCACGCGCATGGCCGCCGAGCGCGAGCCGCGCTTCGAGGACCGCGATGTCACGCTGCACGG
CCGCACCCGCCATGTCTACCAGTGGACGGTTCCGTACGGCGACTCGCTGGGCGAACTCAAGGGCATCATCGGCGGCTGGA
TCGACATTACCGAACGCGCCGAGCTGCTGCGCGAGCTGCACGACGCCAAGGAAAGCGCCGACGCCGCCAACCGGGCCAAG
ACCACGTTCCTGGCAACGATGAGCCACGAGATCCGCACGCCGATGAACGCGATCATCGGCATGCTGGAGCTGGCGCTGCT
CCGTCCGGCCGACCAGGAGCCGGACCGCCAGTCCATCCAGGTCGCGTACGACTCGGCCCGCAGCCTGCTGGAGCTGATAG
GCGACATCCTGGACATTGCGAAGATCGAGGCGGGAAAATTCGACCTGGCGCCGGTGCGCACGGCGCTGCGCGCCCTGCCC
GAAGGGGCGATCCGCCTCTTCGATGGATTGGCGCGCCAGAAAGGCATAGAGCTGGTATTGAAGACCGACATCGTGGGCGT
GGACGATGTATTGATAGACCCCTTGCGCATGAAGCAAGTGCTCTCGAACCTGGTGGGCAACGCCATCAAGTTCACCACCG
AAGGCCAGGTTGTCCTTACCGTAACCGCGCGCCCCGACGGCGAGGCCGCGCACGTGCAGTTCAGCGTGAGCGACACCGGC
TGCGGCATCAGCGAGGCCGACCAACGGCAGCTGTTCAAACCGTTCTCGCAGGTGGGTGGCAGCGCCGAGGCCGGGCCGGC
GCCGGGCACTGGCCTGGGCCTGTCCATCAGCCGCCGCCTCGTCGAATTGATGGGGGGAACGCTGGTCATGCGCAGCGCGC
CAGGCGTGGGCACAACGGTTTCGGTGGACCTGAGGCTGACCATGATCGAAAAATCCGCGCAGGCCACGCCGCCCGCTGCG
GCCGCTCAGGCCACGCCGTCCAAGCCGCAGGTATCGCTGCGCGTGCTGGTCGTCGATGACCACAAGCCCAACCTGATGCT
GCTGCGCCAGCAGCTGGACTACCTGGGCCAGCGTGTCGTCGCCGCCGACTCCGGCGAAGCCGCCCTGGCCCTGTGGCACG
AGCATGCGTTCGACGTCGTGATCACCGATTGCAACATGCCCGGTATCAACGGCTACGAATtGGCGCGCCGCATACGCGCC
GCCGAGGCCGCGCCCGGTTACGGACGTACGCGGTGCATTCTGTTCGGCTTCACGGCTTCGGCGCAGATGGACGAAGCGCA
GCGCTGCCGCGCCGCCGGCATGGACGACTGCCTGTTCAAGCCGATCGGCGTGGACGCCTTGCGGCAACGCTTGAACGAAG
CCGCGGCACGGGCCGCGCTCCCCACGCCCCCCTCGCCCCAGGCTGCCGCGCCGGCCACGCACGACGCCACCCCGGCGGCG
TTCTCGGCCGAGTCGATTCTTGCCCTGACGCAGAACGACGAGGCGCTGATCCGGCAATTGCTCGAAGAAGTGATTCGCAC
CAACCGGGCGGACGTCGATCAATTGCAGAAGCTGCACCAGCAGGCCGATTGGCCGAAGGTCTCGGACATGGCGCACAGGC
TGGCCGGCGGCGCGCGCGTGGTCGATGCCAAGGCCATGATAGACACTGCGCTGGCGCTGGAGAAAAAAGCGCAAGGCCAG
GCTGGCCCCTCGCCCGAAATCGACGGCCTGGTACGTACGCTTGCGGCGCAGTCCGCCGCGCTGGAGACGCAACTACGCGC
CTGGCTGGAGCAACGGCCGCATCAAGGCCAGCCCTGA
>Bp_B1920.seq
ATGCCCGCCCCGCACCGCCTGTACCCCCGCAGTCTGATCTGCCTGGCTCAGGCGCTATTGGCATGGGCTTTGCTGGCATG
GGCGCCCGCGCAGGCAAGCCAGGAGCTGACCCTGGTCGGCAAGGCTGCCGTTCCCGACGTCGAGGTCGCGCTCGACGGCG
ACGACTGGCGTTGGCTGGCCCGCAAGCGGGTACTGACGCTGGGTGTGTACGCACCGGACATTCCTCCGTTCGACGTCACC
TATGGCGAACGCTACGAAGGCCTGACGGCCGACTACATGGCGATCATCGCGCACAACCTGGGGATGCAGGCGAAAGTGCT
GCGATACCCCACGCGCGAACAAGCCCTCAGCGCGCTGGAAAGCGGGCAGATCGACCTCATCGGCACCGTCAATGGCACGG
ACGGCCGGCAACAGAGCCTGCGTCTGAGCGTTCCCTACGCCGCCGACCACCCGGTGATCGTCATGCCCATCGGCGCACGC
CACGTTCCAGCCTCGAACCTGGCCGGCCAGCGGCTGGCGGTCGACATCAACTACCTGCCCAAGGAAACGCTCGCACGGGC
CTACCCGCAGGCTACGCTGCATTACTTCCCCTCATCCGAGCAGGCGCTGGCCGCGGTGGCCTATGGGCAGGCCGACGTAT
TCATCGGCGATGCCCTGACCACCTCGCACCTCGTATCGCAAAGCTATTTCAATGACGTTCGCGTAGTCGCCCCGGCCCAT
ATCGCGACGGGCGGAGAATCCTTCGGCGTGCGCGCCGACAACACCCGCCTGCTGCGGGTGGTCAACGCCGTACTCGAAGC
CATTCCGCCTTCCGAACACCGCAGCCTGATCTACCGCTGGGGACTGGGCAGCAGCATTTCGCTCGATTTCGCGCACCCCG
CGTATTCCGCGCGCGAGCAGCAATGGATGGCAGACCACCCCGTCGTCAAGGTGGCGGTCCTGAATCTGTTCGCGCCCTTC
ACCCTGTTCCGCACCGACGAACAGTTCGGCGGGATCAGCGCCGCCGTGCTGCAGCTGCTGCAATTGCGCACCGGCCTGGA
CTTCGAGATCATCGGCGTCGACACGGTCGAGGAACTGATAGCCAAGCTGCGTTCGGGCGAAGCCGACATGGCCGGCGCCC
TGTTCGTCAACAGCGCGCGGGAGTCCTTCCTCAGTTTCAGCCGGCCGTATGTGCGCAATGGCATGGTGATCGTCACGCGC
CAGGACCCCGACGCGCCCGTCGACGCCGATCATCTGGACGGCCGCACGGTCGCGTTGGTGCGCAACAGCGCCGCCATTCC
CCTGCTGCAGCGGCGCTATCCCCAGGCGAAGGTGGTGACCGCCGACAACCCGAGCGAGGCGATGCTGATGGTGGCCAATG
GACAGGCCGACGCCGTCGTGCAGACGCAGATCAGCGCCAGCTATTACGTCAACCGCTACTTCGCCGGCAAGCTGCGCATC
GCCTCGGCGCTGGACCTGCCCCCGGCCGAGATCGCGCTGGCGACGACGCGCGGCCAGACCGAACTGATGTCCATCCTGAA
CAAGGCGCTCTACAGCATTTCGAACGACGAGCTCGCCTCCATCATCAGCCGCTGGCGCGGCAGCGACGGCGATCCGCGCA
CCTGGTACGCCTACCGCAACGAGATCTACCTGCTGATCGGGCTGGGCCTGTTGTCGGCCCTGCTGTTCCTGAGCTGGATC
GTCTACCTGCGGCGCCAGATCCGCCAGCGCAAGCGGGCCGAGCGGGCGCTGAACGACCAGCTGGAATTCATGCGCGTGCT
CATCGACGGCACGCCTAACCCCATCTATGTGCGCGATAAGGAAGGCCGCATGCTGTTGTGCAATGACGCCTACCTCGACA
CCTTTGGCGTGACTGCCGATGCGGTACTGGGCAAGACCATTCCGGAAGCCAACGTGGTGGGCGACCCGGCGCTGGCCCGC
GAAATGCACGAGTTCCTGCTCACGCGCGTGGCCGCCGAGCGCGAGCCGCGCTTCGAGGACCGCGATGTCACGCTGCACGG
CCGCACCCGCCATGTCTACCAGTGGACGATTCCGTACGGCGACTCGCTGGGCGAACTCAAGGGCATCATCGGCGGCTGGA
TCGACATCACCGAACGCGCCGAGCTGCTGCGCGAGCTGCACGACGCCAAGGAAAGCGCCGACGCCGCCAACCGGGCCAAG
ACCACGTTCCTGGCAACGATGAGCCACGAGATCCGCACGCCGATGaACGCGATCATCGGCATGCTGGAGCTGGCGCTGCT
CCGTCCGACCGACCAGGAGCCGGATCGCCAGTCCATCCAGGTCGCGTACGACTCGGCCCGCAGCCTGCTGGAGCTGATAG
GCGACATCCTGGACATTGCGAAGATCGAGGCGGGAAAATTCGACCTGGCGCCGGTGCGCACGGCGCTGCGCGTCCTGCCC
GAAGGGGCGATCCGCGTCTTCGACGGATTGGCGCGCCAAAAAGGCATAGAGCTGGTATTGAAGACCGACATCGTGGGCGT
CGACGATGTATTGATAGACCCCTTGCGCATGAAGCAAGTGCTCTCGAACCTGGTGGGCAACGCCATCAAGTTCACCACCG
AAGGCCAGGTTGTCCTTGCCGTGACCGCACGCCCCGACGGCGACGCCGCGCACGTGCAGTTCAGCGTGAGCGACACCGGC
TGCGGCATCAGCGAGGCCGACCAACGGCAGCTGTTCAAACCGTTCTCGCAAGTGGGTGGCAGCGCCGAGGCCGGGCCGGC
GCCGGGCACCGGCCTGGGCCTGTCCATCAGCCGGCGCCTCGTCGAATTGATGGGGGGAACGCTGGTCATGCGCAGCGCGC
CAGGGGTGGGCACAACGGTTTCGGTGGACCTGAGGCTGACCATGGTCGAAAAATCCGTGCAGGCCGCGCCGCCCGCTGCG
GCCACTGCGGCCACGCCGTCCAAGCCGCAGGTATCGCTGCGCGTGCTGGTCGTCGATGACCACAAACCCAACCTGATGCT
GCTGCGCCAGCAGCTGGACTACCTGGGCCAGCGTGTCATCGCCGCCGACTCCGGCGAAGCCGCCCTGGCCCTGTGGCGCG
AGCATGCGTTCGACGTCGTGATCACCGATtGCAACATGCCCGGTATCAGCGGCTACGAATtGGCGCGCCGCATACGCGCC
GCCGAGGCCGCGCCCGGTTACGGACGTACGCGGTGCATTCTGTTCGGCTTCACGGCTTCGGCGCAGATGGACGAAGCGCA
GCGCTGCCGCGCCGCCGGCATGGACGACTGCCTGTTCAAGCCGATCGGCGTGGACGCCTTGCGGCAACGCTTGAACGAAG
CCGTGGCACGGGCCGCGCTCCCCACGCCCCCCTCGCCCCAGGCTGCCGCGCCGGCCACGGACGACGCCACCCCGACGGCG
TTCTCGGCCGAGTCGATTCTTGCCTTGACGCAGAACGATGAGGCGCTGATCCGGCAATTGCTCGAAGAAGTGATTCGCAC
CAACCGGGCGGACGTCGACCAATTGCAAAAGCTGCACCAGCAGGCCGATTGGCCGAAGGTCTCGGACATGGCGCACAGGC
TGGCCGGCGGCGCGCGCGTGGTCGATGCCAAGGCCATGATAGACACTGTGCTGGCGCTGGAGAAAAAAGCGCAAGGCCAG
GCTGGCCCCTCACCCGAAATCGACGGCCTGGTACGTACGCTTGCGGCGCAGTCCGCCGCGCTGGAGACGCAACTGCGCGC
CTGGCTGGAGCAACGGCCGCATCAAGATCAGCCCTGA
>Bp_B1979.seq
ATGCCCGCCCCGCACCGCCTGTACCCCCGCAGTCTGATCTGCCTGGCTCAGGCGCTATTGGCATGGGCTTTGCTGGCATG
GGCGCCCGCGCAGGCAAGCCAGGAGCTGACCCTGGTCGGCAAGGCTGCCGTTCCCGACGTCGAGGTCGCGCTCGACGGCG
ACGACTGGCGTTGGCTGGCCCGCAAGCGGGTACTGACGCTGGGTGTGTACGCACCGGACATTCCTCCGTTCGACGTCACC
TATGGCGAACGCTACGAAGGCCTGACGGCCGACTACATGGCGATCATCGCGCACAACCTGGGGATGCAGGCGAAAGTGCT
GCGATACCCCACGCGCGAACAAGCCCTCAGCGCGCTGGAAAGCGGGCAGATCGACCTCATCGGCACCGTCAATGGCACGG
ACGGCCGGCAACAGAGCCTGCGTCTGAGCGTTCCCTACGCCGCCGACCACCCGGTGATCGTCATGCCCATCGGCGCACGC
CACGTTCCAGCCTCGAACCTGGCCGGCCAGCGGCTGGCGGTCGACATCAACTACCTGCCCAAGGAAACGCTCGCACGGGC
CTACCCGCAGGCTACGCTGCATTACTTCCCCTCATCCGAGCAGGCGCTGGCCGCGGTGGCCTATGGGCAGGCCGACGTAT
TCATCGGCGATGCCCTGACCACCTCGCACCTCGTATCGCAAAGCTATTTCAATGACGTTCGCGTAGTCGCCCCGGCCCAT
ATCGCGACGGGCGGAGAATCCTTCGGCGTGCGCGCCGACAACACCCGCCTGCTGCGGGTGGTCAACGCCGTACTCGAAGC
CATTCCGCCTTCCGAACACCGCAGCCTGATCTACCGCTGGGGACTGGGCAGCAGCATTTCGCTCGATTTCGCGCACCCCG
CGTATTCCGCGCGCGAGCAGCAATGGATGGCAGACCACCCCGTCGTCAAGGTGGCGGTCCTGAATCTGTTCGCGCCCTTC
ACCCTGTTCCGCACCGACGAACAGTTCGGCGGGATCAGCGCCGCCGTGCTGCAGCTGCTGCAATTGCGCACCGGCCTGGA
CTTCGAGATCATCGGCGTCGACACGGTCGAGGAACTGATAGCCAAGCTGCGTTCGGGCGAAGCCGACATGGCCGGCGCCC
TGTTCGTCAACAGCGCGCGGGAGTCCTTCCTCAGTTTCAGCCGGCCGTATGTGCGCAATGGCATGGTGATCGTCACGCGC
CAGGACCCCGACGCGCCCGTCGACGCCGATCATCTGGACGGCCGCACGGTCGCGTTGGTGCGCAACAGCGCCGCCATTCC
CCTGCTGCAGCGGCGCTATCCCCAGGCGAAGGTGGTGACCGCCGACAACCCGAGCGAGGCGATGCTGATGGTGGCCAATG
GACAGGCCGACGCCGTCGTGCAGACGCAGATCAGCGCCAGCTATTACGTCAACCGCTACTTCGCCGGCAAGCTGCGCATC
GCCTCGGCGCTGGACCTGCCCCCGGCCGAGATCGCGCTGGCGACGACGCGCGGCCAGACCGAACTGATGTCCATCCTGAA
CAAGGCGCTCTACAGCATTTCGAACGACGAGCTCGCCTCCATCATCAGCCGCTGGCGCGGCAGCGACGGCGATCCGCGCA
CCTGGTACGCCTACCGCAACGAGATCTACCTGCTGATCGGGCTGGGCCTGTTGTCGGCCCTGCTGTTCCTGAGCTGGATC
GTCTACCTGCGGCGCCAGATCCGCCAGCGCAAGCGGGCCGAGCGGGCGCTGAACGACCAGCTGGAATTCATGCGCGTGCT
CATCGACGGCACGCCTAACCCCATCTATGTGCGCGATAAGGAAGGCCGCATGCTGTTGTGCAATGACGCCTACCTCGACA
CCTTTGGCGTGACTGCCGATGCGGTACTGGGCAAGACCATTCCGGAAGCCAACGTGGTGGGCGACCCGGCGCTGGCCCGC
GAAATGCACGAGTTCCTGCTCACGCGCGTGGCCGCCGAGCGCGAGCCGCGCTTCGAGGACCGCGATGTCACGCTGCACGG
CCGCACCCGCCATGTCTACCAGTGGACGATTCCGTACGGCGACTCGCTGGGCGAACTCAAGGGCATCATCGGCGGCTGGA
TCGACATCACCGAACGCGCCGAGCTGCTGCGCGAGCTGCACGACGCCAAGGAAAGCGCCGACGCCGCCAACCGGGCCAAG
ACCACGTTCCTGGCAACGATGAGCCACGAGATCCGCACGCCGATGaACGCGATCATCGGCATGCTGGAGCTGGCGCTGCT
CCGTCCGACCGACCAGGAGCCGGATCGCCAGTCCATCCAGGTCGCGTACGACTCGGCCCGCAGCCTGCTGGAGCTGATAG
GCGACATCCTGGACATTGCGAAGATCGAGGCGGGAAAATTCGACCTGGCGCCGGTGCGCACGGCGCTGCGCGTCCTGCCC
GAAGGGGCGATCCGCGTCTTCGACGGATTGGCGCGCCAAAAAGGCATAGAGCTGGTATTGAAGACCGACATCGTGGGCGT
CGACGATGTATTGATAGACCCCTTGCGCATGAAGCAAGTGCTCTCGAACCTGGTGGGCAACGCCATCAAGTTCACCACCG
AAGGCCAGGTTGTCCTTGCCGTGACCGCACGCCCCGACGGCGACGCCGCGCACGTGCAGTTCAGCGTGAGCGACACCGGC
TGCGGCATCAGCGAGGCCGACCAACGGCAGCTGTTCAAACCGTTCTCGCAAGTGGGTGGCAGCGCCGAGGCCGGGCCGGC
GCCGGGCACCGGCCTGGGCCTGTCCATCAGCCGGCGCCTCGTCGAATTGATGGGGGGAACGCTGGTCATGCGCAGCGCGC
CAGGGGTGGGCACAACGGTTTCGGTGGACCTGAGGCTGACCATGGTCGAAAAATCCGTGCAGGCCGCGCCGCCCGCTGCG
GCCACTGCGGCCACGCCGTCCAAGCCGCAGGTATCGCTGCGCGTGCTGGTCGTCGATGACCACAAACCCAACCTGATGCT
GCTGCGCCAGCAGCTGGACTACCTGGGCCAGCGTGTCATCGCCGCCGACTCCGGCGAAGCCGCCCTGGCCCTGTGGCGCG
AGCATGCGTTCGACGTCGTGATCACCGATTGCAACATGCCCGGTATCAGCGGCTACGAATTGGCGCGCCGCATACGCGCC
GCCGAGGCCGCGCCCGGTTACGGACGTACGCGGTGCATTCTGTTCGGCTTCACGGCTTCGGCGCAGATGGACGAAGCGCA
GCGCTGCCGCGCCGCCGGCATGGACGACTGCCTGTTCAAGCCGATCGGCGTGGACGCCTTGCGGCAACGCTTGAACGAAG
CCGTGGCACGGGCCGCGCTCCCCACGCCCCCCTCGCCCCAGGCTGCCGCGCCGGCCACGGACGACGCCACCCCGACGGCG
TTCTCGGCCGAGTCGATTCTTGCCTTGACGCAGAACGATGAGGCGCTGATCCGGCAATTGCTCGAAGAAGTGATTCGCAC
CAACCGGGCGGACGTCGACCAATTGCAAAAGCTGCACCAGCAGGCCGATTGGCCGAAGGTCTCGGACATGGCGCACAGGC
TGGCCGGCGGCGCGCGCGTGGTCGATGCCAAGGCCATGATAGACACTGTGCTGGCGCTGGAGAAAAAAGCGCAAGGCCAG
GCTGGCCCCTCACCCGAAATCGACGGCCTGGTACGTACGCTTGCGGCGCAGTCCGCCGCGCTGGAGACGCAACTGCGCGC
CTGGCTGGAGCAACGGCCGCATCAAGATCAGCCCTGA
>Bp_B1980.seq
ATGCCCGCCCCGCACCGCCTGTACCCCCGCAGTCTGATCTGCCTGGCTCAGGCGCTATTGGCATGGGCTTTGCTGGCATG
GGCGCCCGCGCAGGCAAGCCAGGAGCTGACCCTGGTCGGCAAGGCTGCCGTTCCCGACGTCGAGGTCGCGCTCGACGGCG
ACGACTGGCGTTGGCTGGCCCGCAAGCGGGTACTGACGCTGGGTGTGTACGCACCGGACATTCCTCCGTTCGACGTCACC
TATGGCGAACGCTACGAAGGCCTGACGGCCGACTACATGGCGATCATCGCGCACAACCTGGGGATGCAGGCGAAAGTGCT
GCGATACCCCACGCGCGAACAAGCCCTCAGCGCGCTGGAAAGCGGGCAGATCGACCTCATCGGCACCGTCAATGGCACGG
ACGGCCGGCAACAGAGCCTGCGTCTGAGCGTTCCCTACGCCGCCGACCACCCGGTGATCGTCATGCCCATCGGCGCACGC
CACGTTCCAGCCTCGAACCTGGCCGGCCAGCGGCTGGCGGTCGACATCAACTACCTGCCCAAGGAAACGCTCGCACGGGC
CTACCCGCAGGCTACGCTGCATTACTTCCCCTCATCCGAGCAGGCGCTGGCCGCGGTGGCCTATGGGCAGGCCGACGTAT
TCATCGGCGATGCCCTGACCACCTCGCACCTCGTATCGCAAAGCTATTTCAATGACGTTCGCGTAGTCGCCCCGGCCCAT
ATCGCGACGGGCGGAGAATCCTTCGGCGTGCGCGCCGACAACACCCGCCTGCTGCGGGTGGTCAACGCCGTACTCGAAGC
CATTCCGCCTTCCGAACACCGCAGCCTGATCTACCGCTGGGGACTGGGCAGCAGCATTTCGCTCGATTTCGCGCACCCCG
CGTATTCCGCGCGCGAGCAGCAATGGATGGCAGACCACCCCGTCGTCAAGGTGGCGGTCCTGAATCTGTTCGCGCCCTTC
ACCCTGTTCCGCACCGACGAACAGTTCGGCGGGATCAGCGCCGCCGTGCTGCAGCTGCTGCAATTGCGCACCGGCCTGGA
CTTCGAGATCATCGGCGTCGACACGGTCGAGGAACTGATAGCCAAGCTGCGTTCGGGCGAAGCCGACATGGCCGGCGCCC
TGTTCGTCAACAGCGCGCGGGAGTCCTTCCTCAGTTTCAGCCGGCCGTATGTGCGCAATGGCATGGTGATCGTCACGCGC
CAGGACCCCGACGCGCCCGTCGACGCCGATCATCTGGACGGCCGCACGGTCGCGTTGGTGCGCAACAGCGCCGCCATTCC
CCTGCTGCAGCGGCGCTATCCCCAGGCGAAGGTGGTGACCGCCGACAACCCGAGCGAGGCGATGCTGATGGTGGCCAATG
GACAGGCCGACGCCGTCGTGCAGACGCAGATCAGCGCCAGCTATTACGTCAACCGCTACTTCGCCGGCAAGCTGCGCATC
GCCTCGGCGCTGGACCTGCCCCCGGCCGAGATCGCGCTGGCGACGACGCGCGGCCAGACCGAACTGATGTCCATCCTGAA
CAAGGCGCTCTACAGCATTTCGAACGACGAGCTCGCCTCCATCATCAGCCGCTGGCGCGGCAGCGACGGCGATCCGCGCA
CCTGGTACGCCTACCGCAACGAGATCTACCTGCTGATCGGGCTGGGCCTGTTGTCGGCCCTGCTGTTCCTGAGCTGGATC
GTCTACCTGCGGCGCCAGATCCGCCAGCGCAAGCGGGCCGAGCGGGCGCTGAACGACCAGCTGGAATTCATGCGCGTGCT
CATCGACGGCACGCCTAACCCCATCTATGTGCGCGATAAGGAAGGCCGCATGCTGTTGTGCAATGACGCCTACCTCGACA
CCTTTGGCGTGACTGCCGATGCGGTACTGGGCAAGACCATTCCGGAAGCCAACGTGGTGGGCGACCCGGCGCTGGCCCGC
GAAATGCACGAGTTCCTGCTCACGCGCGTGGCCGCCGAGCGCGAGCCGCGCTTCGAGGACCGCGATGTCACGCTGCACGG
CCGCACCCGCCATGTCTACCAGTGGACGATTCCGTACGGCGACTCGCTGGGCGAACTCAAGGGCATCATCGGCGGCTGGA
TCGACATCACCGAACGCGCCGAGCTGCTGCGCGAGCTGCACGACGCCAAGGAAAGCGCCGACGCCGCCAACCGGGCCAAG
ACCACGTTCCTGGCAACGATGAGCCACGAGATCCGCACGCCGATGAACGCGATCATCGGCATGCTGGAGCTGGCGCTGCT
CCGTCCGACCGACCAGGAGCCGGATCGCCAGTCCATCCAGGTCGCGTACGACTCGGCCCGCAGCCTGCTGGAGCTGATAG
GCGACATCCTGGACATTGCGAAGATCGAGGCGGGAAAATTCGACCTGGCGCCGGTGCGCACGGCGCTGCGCGTCCTGCCC
GAAGGGGCGATCCGCGTCTTCGACGGATTGGCGCGCCAAAAAGGCATAGAGCTGGTATTGAAGACCGACATCGTGGGCGT
CGACGATGTATTGATAGACCCCTTGCGCATGAAGCAAGTGCTCTCGAACCTGGTGGGCAACGCCATCAAGTTCACCACCG
AAGGCCAGGTTGTCCTTGCCGTGACCGCACGCCCCGACGGCGACGCCGCGCACGTGCAGTTCAGCGTGAGCGACACCGGC
TGCGGCATCAGCGAGGCCGACCAACGGCAGCTGTTCAAACCGTTCTCGCAAGTGGGTGGCAGCGCCGAGGCCGGGCCGGC
GCCGGGCACCGGCCTGGGCCTGTCCATCAGCCGGCGCCTCGTCGAATTGATGGGGGGAACGCTGGTCATGCGCAGCGCGC
CAGGGGTGGGCACAACGGTTTCGGTGGACCTGAGGCTGACCATGGTCGAAAAATCCGTGCAGGCCGCGCCGCCCGCTGCG
GCCACTGCGGCCACGCCGTCCAAGCCGCAGGTATCGCTGCGCGTGCTGGTCGTCGATGACCACAAACCCAACCTGATGCT
GCTGCGCCAGCAGCTGGACTACCTGGGCCAGCGTGTCATCGCCGCCGACTCCGGCGAAGCCGCCCTGGCCCTGTGGCGCG
AGCATGCGTTCGACGTCGTGATCACCGATTGCAACATGCCCGGTATCAGCGGCTACGAATTGGCGCGCCGCATACGCGCC
GCCGAGGCCGCGCCCGGTTACGGACGTACGCGGTGCATTCTGTTCGGCTTCACGGCTTCGGCGCAGATGGACGAAGCGCA
GCGCTGCCGCGCCGCCGGCATGGACGACTGCCTGTTCAAGCCGATCGGCGTGGACGCCTTGCGGCAACGCTTGAACGAAG
CCGTGGCACGGGCCGCGCTCCCCACGCCCCCCTCGCCCCAGGCTGCCGCGCCGGCCACGGACGACGCCACCCCGACGGCG
TTCTCGGCCGAGTCGATTCTTGCCTTGACGCAGAACGATGAGGCGCTGATCCGGCAATTGCTCGAAGAAGTGATTCGCAC
CAACCGGGCGGACGTCGACCAATTGCAAAAGCTGCACCAGCAGGCCGATTGGCCGAAGGTCTCGGACATGGCGCACAGGC
TGGCCGGCGGCGCGCGCGTGGTCGATGCCAAGGCCATGATAGACACTGTGCTGGCGCTGGAGAAAAAAGCGCAAGGCCAG
GCTGGCCCCTCACCCGAAATCGACGGCCTGGTACGTACGCTTGCGGCGCAGTCCGCCGCGCTGGAGACGCAACTGCGCGC
CTGGCTGGAGCAACGGCCGCATCAAGATCAGCCCTGA
>Bpp_B0203.seq
ATGCCCGCCCCGCACCGCCTGTACCCCCGCAGTCTGATCTGCCTGGCTCAGGCGCTATTGGCATGGGCTTTGCTGGCATG
GGCGCCCGCGCAGGCAAGCCAGGAGCTGACCCTGGTCGGCAAGGCTGCCGTTCCCGACGTCGAGATCACGCTCGACGGCG
ACGACTGGCGCTGGCTGGCGCGCAAGCGGGTGCTGACGCTGGGCGTGTACGCGCCGGACATTCCCCCGTTCGACGTCACC
TATGACGAGCGCTACGAAGGCCTGACGGCCGACTACATGGCGATCATCGCGCACAACCTGGGCGTCCAGGCAAAAGTGCT
GCGCTACCCCACGCGCGAGCAAGCCGTCGGCGCACTGGAAAGCGGACAGATCGACCTCATCGGCACCGTCAATGGCATCG
AGGGCCGGCTGCAGAGCCTGCGCCTGAGTGTTCCCTACGCGGCCGACCACCCGGTGCTGGTCATGCCCATCGGCGCGCGC
CGCGCTCCGCCCGCGGACCTGGCAGGCCAGCGGTTGGCGGTTGACGCCAACTACTTGCCCAGGGAAACGCTGCAGCAGGC
CTATCCCCAGGCAACGCTGCATTACTTCCCATCATCCGAACAGGCGCTGGCCGCGGTGGCCTATGGACAAGCCGACGTGT
TCATCGGCGATGCGCTGACCACCTCGCACCTCGTTTCGCAAAGCTACTTCAACGACGTTCGCGTCGTCGCCCCGGCCCAG
ATCGTGACGGGCGGGGAATCCTTCGGCGTGCGCGCCGACAATACCCGCCTGCTGCGGGTGGTCAATGCCGTGCTCGAAGC
CATTCCGGCCTCCGAGCGCCGCAGCCTGATCTACCGCTGGGGCCTGGGCAGCAGCATTTCGCTCGATTTCGCGCGCCCCG
CCTATTCAGCGCGCGAGCAGCAGTGGATGGCAAACCATCCGGTCGTCAAGGTGGCGGTCCTGAACCTGTTCGCGCCCTTC
ACCCTGTTTCGCACCGATGAACAGTTCGGCGGCATCAGTGCCGCCGTGCTGCAACTGCTGCAGTTGCGCACCGGCCTGGA
TTTCCAGATCATCGGCGTCGACACGGTCGAGGAGCTGATCGCCAAGCTGCGCTCGGGCGAAGCCGACATGGCCGGCGCCC
TGTTCGTCAATGCCGCGCGGGAATCCGTCCTCAGCTTCAGCCGGCCGTATGTGCGCAATGGTCTGGTGATCGTCACGCGC
CAGGACCCCGCCGCGCCCGCCGACGCCGATCACCTCGACGGCCGCACGATTGCGATGGTGCGCAACAGCGCCGCCATCCC
GCTCCTGCAGCAGCGCTATCCCCAGGCGAAGGTCGTGACCGCCGACAACCCGACCGAAGCCATGCTGCTGGTGGCCGATG
GCCAGGCCGACGCCGTCGTGCAGACGCAGATCAGCGCCAGCTACTACGTCAACCGCTACTTCGCCGGAAAACTGCGCATT
GCCTCGGCGCTGGACCTGCCGCCGGCCGAGATCGCGCTGGCGACGGCGCGCGGCCAGACCGAGCTGATATCCATCCTGAA
CAAGGCGCTCTACAGCATTTCGAACGACGAACTCGCCTCCATCGTCAGCCGCTGGCGCGGCAGCGACGGCGATCCGCGCA
CCTGGTACGCCTACCGCAACGAAATCTACCTGCTGATCGGGCTGGGCCTGTTGTCGGCCCTGCTGTTCCTGAGCTGGATC
GTCTACCTGCGGCGCCAGATCCGCCAGCGCAAGCGGGCCGAGCGGGCGCTGAACGACCAGCTGGAATTCATGCGCGTGCT
CATCGACGGCACGCCCAACCCCATCTATGTGCGCGATAAGGAAGGCCGCATGCTGTTGTGCAATGACGCCTACCTCGACA
CCTTTGGCGTGACTGCCGATGCGGTACTGGGCAAGACCATCCCGGAGGCCAACGTGGTGGGCGACCCGGCGCTGGCTCGC
GAGATGCACGAGTTCCTGCTCACGCGCATGGCCGCCGAGCGCGAGCCGCGCTTCGAGGACCGCGATGTCACGCTGCACGG
CCGCACCCGCCATGTCTACCAGTGGACGGTTCCGTACGGCGACTCGCTGGGCGAACTCAAGGGCATCATCGGCGGCTGGA
TCGACATTACCGAACGCGCCGAGCTGCTGCGCGAGCTGCACGACGCCAAGGAAAGCGCCGACGCCGCCAACCGGGCCAAG
ACCACGTTCCTGGCAACGATGAGCCACGAGATCCGCACGCCGATGAACGCGATCATCGGCATGCTGGAGCTGGCGCTGCT
CCGTCCGGCCGACCAGGAGCCGGACCGCCAGTCCATCCAGGTCGCGTACGACTCGGCCCGCAGCCTGCTGGAGCTGATAG
GCGACATCCTGGACATTGCGAAGATCGAGGCGGGAAAATTCGACCTGGCGCCGGTGCGCACGGCGCTGCGCGCCCTGCCC
GAAGGGGCGATCCGCGTCTTCGACGGGTTGGCGCGCCAGAAAGGCATAGAGCTGGTATTGAAGACCGACATCGTGGGCGT
GGACGATGTATTGATAGACCCCTTGCGCATGAAGCAAGTGCTCTCGAGCCTGGTGGGCAACGCCATCAAGTTCACCACCG
AAGGCCAGGTTGTCCTTACCGTGACCGCGCGCCCCGACGGCGAGGCCGCGCACGTGCAGTTCAGCGTGAGCGACACCGGC
TGCGGCATCAGCGAGGCCGACCAACGGCAGCTGTTCAAACCGTTCTCGCAGGTGGGCGGCAGCGCCGAGGCCGGGCCGGC
GCCGGGCACTGGCCTGGGCCTGTCCATCAGCCGCCGCCTCGTCGAATTGATGGGGGGAACGCTGGTCATGCGCAGCGCGC
CAGGGGTGGGCACAACGGTTTCGGTGGACCTGAGGCTGACCATGGTCGAAAAATCCGCGCAGGCCACGCCGCCCGCTGCG
GCCGCTCAGGCCACGCCATCCAAGCCGCAGGTATCGCTGCGCGTGCTGGTCGTCGATGACCACAAGCCCAACCTGATGCT
GCTGCGCCAGCAGCTGGACTACCTGGGCCAGCGTGTCGTCGCCGCCGACTCCGGCGAAGCCGCCCTGGCCCTGTGGCACG
AGCATGCGTTCGACGTCGTGATCACCGATTGCAACATGCCCGGTATCAACGGCTACGAATTGGCGCGCCGCATACGCGCC
GCCGAGGCCGCGCCCGGTTACGGACGTACGCGGTGCATTCTGTTCGGCTTCACGGCTTCGGCGCAGATGGACGAAGCGCA
GCGCTGCCGCGCCGCCGGCATGGACGACTGCCTGTTCAAGCCGATCGGCGTGGACGCCTTGCGGCAACGCTTGAACGAAG
CCGCGGCACGGGCCGCGCTCCCCACGCCCCCCTCGCCCCAGGCTGCCGCGCCGGCCACGCACGACGCCACCCCGGCGGCG
TTCTCGGCCGAGTCGATTCTTGCCCTGACGCAGAACGATGAGGCGCTGATCCGGCAATTGCTCGAAGAAGTGATTCGCAC
CAACCGGGCGGACGTCGATCAATTGCAGAAGCTGCACCAGCAGGCCGATTGGCCGAAGGTCTCGGACATGGCGCACAGGC
TGGCCGGCGGCGCGCGCGTGGTCGATGCCAAGGCCATGATAGACACTGCGCTGGCGCTGGAGAAAAAAGCGCAAGGCCAG
GCTGGCCCCTCGCCCGAAATCGACGGCATGGTACGTACGCTTGCGGCGCAGTCCGCCGCGCTGGAGACGCAACTACGCGC
CTGGCTGGAGCAACGGCCGCATCAAGGCCAGCCCTGA
>Bpp_B0204.seq
ATGCCCGCCCCGCACCGCCTGTACCCCCGCAGTCTGATCTGCCTGGCTCAGGCGCTATTGGCATGGGCTTTGCTGGCATG
GCCGCCCGCGCAGGCAAGCCAGGAGCTGACCCTGGTCGGCAAGGCTGCCGTTCCCGACGTCGAGGTCGCGCTCGACGGCG
ACGACTGGCGCTGGCTGGCGCGCAAGCGGGTGCTGACGCTGGGCGTGTACGCGCCGGACATTCCCCCGTTCGACGTCACC
TATGACAAGCGCTACGAAGGCCTGACGGCCGACTACATGGCGATCATCGCGCACAACCTGGGCGTCCAGGCAAAAGTGCT
GCGCTACCCCACGCGCGAGCAAGCCGTCGGCGCACTGGAAAACGGGCAGATCGACCTCATCGGCACCGTCAATGGCATCG
AGGGCCGGCAGCAGAGCCTGCGCCTGAGTGTTCCCTACGCGGCCGACCACCCGGTGCTGGTCATGCCCATCGGCGCGCGC
CGCGCTCCGCCCGCGGACCTGGCAGGCCAGCGGTTGGCGGTTGACGCCAACTACTTGCCCAGGGAAACGCTGCAGCAGGC
CTATCCCCAGGCAACGCTGCATTACTTCCCATCGTCCGAACAGGCGCTGGCCGCGGTGGCCTATGGACAAGCCGACGTGT
TCATCGGCGATGCGCTGACCACCTCGCACCTCGTTTCGCAAAGCTACTTCAACGACGTTCGCGTCGTCGCCCCGGCCCAG
ATCGTGACGGGCGGGGAATCCTTCGGCGTGCGCGCCGACAATACCCGCCTGCTGCGGGTGGTCAATGCCGTGCTCGAAGC
CATTCCGGCCTCCGAGCGCCGCAGCCTGATCTACCGCTGGGGCCTGGGCAGCAGCATTTCGCTCGATTTCGCGCGCCCCG
CCTATTCAGCGCGCGAGCAGCAGTGGATGACAAACCATCCGGTCGTCAAGGTGGCGGTCCTGAACCTGTTCGCGCCCTTC
ACCCTGTTTCGCACCGATGAACAGTTCGGCGGCATCAGTGCCGCCGTGCTGCAACTGCTGCAGTTGCGCACCGGCCTGGA
TTTCCAGATCATCGGCGTCGACACGGTCGAGGAGCTGATCGCCAAGCTGCGCTCGGGCGAAGCCGACATGGCCGGCGCCC
TGTTCGTCAATGCCGCGCGGGAATCCGTCCTCAGCTTCAGCCGGCCGTATGTGCGCAATGGTCTGGTGATCGTCACGCGC
CAGGACCCCGCCGCGCCCGCCGACGCCGATCACCTCGACGGCCGCACGATTGCGATGGTGCGCAACAGCGCCGCCATCCC
GCTCCTGCAGCAGCGCTATCCCCAGGCGAAGGTCGTGACCGCCGACAACCCGACCGAAGCCATGCTGCTGGTGGCCGATG
GCCAGGCCGACGCCGTCGTGCAGACGCAGATCAGCGCCAGCTACTACGTCAACCGCTACTTCGCCGGAAAACTGCGCATT
GCCTCGGCGCTGGACCTCCCTCCGGCCGAGATCGCGCTGGCGACGGCGCGCGGCCAGACCGAGCTGATATCCATCCTGAA
CAAGGCGCTCTACAGCATTTCGAACGACGAACTCGCCTCCATCGTCAGCCGCTGGCGTGGCAGCGACGGCGATCCGCGCA
CCTGGTACGCCTACCGCAACGAGATCTACCTGCTGATCGCGCTGGGCCTGTTGTCGGCCCTGCTGTTCCTGAGCTGGATC
GTCTACCTGCGGCGCCAGATCCGCCAACGCAAGCGGGCCGAGCGGGCGCTGAACGACCAGCTGGAATTCATGCGCGTGCT
CATCGACGGCACGCCCAACCCCATCTATGTGCGCGATAAGGAAGGCCGCATGCTGTTGTGCAATGACGCCTACCTCGACA
CCTTTGGCGTGACTGCCGATGCGGTACTGGGCAAGACCATCCCGGAGGCCAACGTGGTGGGCGACCCGGCGCTGGCTCGC
GAGATGCACGAGTTCCTGCTCACGCGCATGGCCGCCGAGCGCGAGTCGCGCTTCGAGGACCGCGATGTCACGCTGCACGG
CCGCACCCGCCATGTCTACCAGTGGACGGTTCCGTACGGCGACTCGCTGGGCGAACTCAAGGGCATCATCGGCGGCTGGA
TCGACATTACCGAACGCGCCGAGCTGCTGCGCGAGCTGCACGACGCCAAGGAAAGCGCCGACGCCGCCAACCGGGCCAAG
ACCACGTTCCTGGCAACGATGAGCCACGAGATCCGCACGCCGATGAACGCGATCATCGGCATGCTGGAGCTGGCGCTGCT
CCGTCCGGCCGACCAGGAGCCGGACCGCCAGTCCATCCAGGTCGCGTACGACTCGGCCCGCAGCCTGCTGGAGCTGATAG
GCGACATCCTGGACATTACGAAGATCGAGGCGGGAAAATTCGACCTGGCGCCGGTGCGCACGGCGCTGCGCGCCCTGCCC
GAAGGGGCGATCCGCGTCTTCGATGGATTGGCGCGCCAGAAAGGCATAGAGCTGGTATTGAAGACCGACATCGTGGGCGT
GGACGATGTATTGATAGACCCCTTGCGCATGAAGCAAGTGCTCTCGAACCTGGTGGGCAACGCCATCAAGTTCACCACCG
AAGGCCAGGTTGTCCTTACCGTGACCGCGCGCCCCGACGGCGAGGCCGCGCACGTGCAGTTCAGCGTGAGCGACACCGGC
TGCGGCATCAGCGAGGCCGACCAACGGCAGCTGTTCAAACCGTTCTCGCAGGTGGGTGGCAGCGCCGAGGCCGGGCCGGC
GCCGGGCACTGGCCTGGGCCTGTCCATCAGCCGCCGCCTCGTCGAATTGATGGGGGGAACGCTGGTCATGCGCAGCGCGC
CAGGGGTGGGCACAACGGTTTCGGTGGACCTGAGGCTGACCATGATCGAAAAATCCGCGCAGGCCACGCCGCCCGCTGCG
GCCGCTCAGGCCACGCCGTCCAAGCCGCAGGTATCGCTGCGCGTGCTGGTCGTCGATGACCACAAGCCCAACCTGATGCT
GCTGCGCCAGCAGCTGGACTACCTGGGCCAGCGTGTCGTCGCCGCCGACTCCGGCGAAGCCGCCCTGGCCCTGTGGCACG
AGCATGCGTTCGACGTCGTGATCACCGATTGCAACATGCCCGGTATCAACGGCTACGAATTGGCGCGCCGCATACGCGCC
GCCGAGGCCGCGCCCGGTTACGGACGTACGCGGTGCATTCTGTTCGGCTTCACGGCTTCGGCGCAGATGGACGAAGTGCA
GCGCTGCCGCGCCGCCGGCATGGACGACTGCCTGTTCAAGCCGATCGGCGTGGACGCCTTGCGGCAACGCTTGAACGAAG
CCGCGGCACGGGCCGCGCTCCCCACGCCCCCCTCGCCCCAGGCTGCCGCGCCGGCCACGCACGACGCCACCCCGGCGGCG
TTCTCGGCCGAGTCGATTCTTGCCCTGACGCAGAACGATGAGGCGCTCATCCGGCAATTGCTCGAAGAAGTGATTCGCAC
CAACCGGGCGGACGTCGATCAATTGCAGAAGCTGCACCAGCAGGCCGATTGGCCGAAGGTCTCGGACATGGCGCACAGGC
TGGCCGGCGGCGCGCGCGTGGTCGATGCCAAGGCCATGATAGACACTGCGCTGGCGCTGGAGAAAAAAGCGCAAGGCCAG
GCTGGCCCCTCGCCCGAAATCGACGGCCTGGTACGTACGCTTGCGGCGCAGTCCGCCGCGCTGGAGACGCAACTACGCGC
CTGGCTGGAGCAACGGCCGCATCAAGGCCAGCCCTGA
>Bpp_B0207.seq
ATGCCCGCCCCGCACCGCCTGTACCCCCGCAGTCTGATCTGCCTGGCTCAGGCGCTATTGGCATGGGCTTTGCTGGCATG
GCCGCCCGCGCAGGCAAGCCAGGAGCTGACCCTGGTCGGCAAGGCTGCCGTTCCCGACGTCGAGGTCGCGCTCGACGGCG
ACGACTGGCGCTGGCTGGCGCGCAAGCGGGTGCTGACGCTGGGCGTGTACGCGCCGGACATTCCCCCGTTCGACGTCACC
TATGACAAGCGCTACGAAGGCCTGACGGCCGACTACATGGCGATCATCGCGCACAACCTGGGCGTCCAGGCAAAAGTGCT
GCGCTACCCCACGCGCGAGCAAGCCGTCGGCGCACTGGAAAACGGGCAGATCGACCTCATCGGCACCGTCAATGGCATCG
AGGGCCGGCAGCAGAGCCTGCGCCTGAGTGTTCCCTACGCGGCCGACCACCCGGTGCTGGTCATGCCCATCGGCGCGCGC
CGCGCTCCGCCCGCGGACCTGGCAGGCCAGCGGTTGGCGGTTGACGCCAACTACTTGCCCAGGGAAACGCTGCAGCAGGC
CTATCCCCAGGCAACGCTGCATTACTTCCCATCGTCCGAACAGGCGCTGGCCGCGGTGGCCTATGGACAAGCCGACGTGT
TCATCGGCGATGCGCTGACCACCTCGCACCTCGTTTCGCAAAGCTACTTCAACGACGTTCGCGTCGTCGCCCCGGCCCAG
ATCGTGACGGGCGGGGAATCCTTCGGCGTGCGCGCCGACAATACCCGCCTGCTGCGGGTGGTCAATGCCGTGCTCGAAGC
CATTCCGGCCTcCGAGCGCCGCAGCCTGATCTACCGCTGGGGCCTGGGCAGCAGCATTTCGCTCGATTTCGCGCGCCCCG
CCTATTCAGCGCGCGAGCAGCAGTGGATGACAAACCATCCGGTCGTCAAGGTGGCGGTCCTGAACCTGTTCGCGCCCTTC
ACCCTGTTTCGCACCGATGAACAGTTCGGCGGCATCAGTGCCGCCGTGCTGCAACTGCTGCAGTTGCGCACCGGCCTGGA
TTTCCAGATCATCGGCGTCGACACGGTCGAGGAGCTGATCGCCAAGCTGCGCTCGGGCGAAGCCGACATGGCCGGCGCCC
TGTTCGTCAATGCCGCGCGGGAATCCGTCCTCAGCTTCAGCCGGCCGTATGTGCGCAATGGTCTGGTGATCGTCACGCGC
CAGGACCCCGCCGCGCCCGCCGACGCCGATCACCTCGACGGCCGCACGATTGCGATGGTGCGCAACAGCGCCGCCATCCC
GCTCCTGCAGCAGCGCTATCCCCAGGCGAAGGTCGTGACCGCCGACAACCCGACCGAAGCCATGCTGCTGGTGGCCGATG
GCCAGGCCGACGCCGTCGTGCAGACGCAGATCAGCGCCAGCTACTACGTCAACCGCTACTTCGCCGGAAAACTGCGCATT
GCCTCGGCGCTGGACCTCCCTCCGGCCGAGATCGCGCTGGCGACGGCGCGCGGCCAGACCGAGCTGATATCCATCCTGAA
CAAGGCGCTCTACAGCATTTCGAACGACGAACTCGCCTCCATCGTCAGCCGCTGGCGTGGCAGCGACGGCGATCCGCGCA
CCTGGTACGCCTACCGCAACGAGATCTACCTGCTGATCGCGCTGGGCCTGTTGTCGGCCCTGCTGTTCCTGAGCTGGATC
GTCTACCTGCGGCGCCAGATCCGCCAACGCAAGCGGGCCGAGCGGGCGCTGAACGACCAGCTGGAATTCATGCGCGTGCT
CATCGACGGCACGCCCAACCCCATCTATGTGCGCGATAAGGAAGGCCGCATGCTGTTGTGCAATGACGCCTACCTCGACA
CCTTTGGCGTGACTGCCGATGCGGTACTGGGCAAGACCATCCCGGAGGCCAACGTGGTGGGCGACCCGGCGCTGGCTCGC
GAGATGCACGAGTTCCTGCTCACGCGCATGGCCGCCGAGCGCGAGTCGCGCTTCGAGGACCGCGATGTCACGCTGCACGG
CCGCACCCGCCATGTCTACCAGTGGACGGTTCCGTACGGCGACTCGCTGGGCGAACTCAAGGGCATCATCGGCGGCTGGA
TCGACATTACCGAACGCGCCGAGCTGCTGCGCGAGCTGCACGACGCCAAGGAAAGCGCCGACGCCGCCAACCGGGCCAAG
ACCACGTTCCTGGCAACGATGAGCCACGAGATCCGCACGCCGATGAACGCGATCATCGGCATGCTGGAGCTGGCGCTGCT
CCGTCCGGCCGACCAGGAGCCGGACCGCCAGTCCATCCAGGTCGCGTACGACTCGGCCCGCAGCCTGCTGGAGCTGATAG
GCGACATCCTGGACATTACGAAGATCGAGGCGGGAAAATTCGACCTGGCGCCGGTGCGCACGGCGCTGCGCGCCCTGCCC
GAAGGGGCGATCCGCGTCTTCGATGGATTGGCGCGCCAGAAAGGCATAGAGCTGGTATTGAAGACCGACATCGTGGGCGT
GGACGATGTATTGATAGACCCCTTGCGCATGAAGCAAGTGCTCTCGAACCTGGTGGGCAACGCCATCAAGTTCACCACCG
AAGGCCAGGTTGTCCTTACCGTGACCGCGCGCCCCGACGGCGAGGCCGCGCACGTGCAGTTCAGCGTGAGCGACACCGGC
TGCGGCATCAGCGAGGCCGACCAACGGCAGCTGTTCAAACCGTTCTCGCAGGTGGGTGGCAGCGCCGAGGCCGGGCCGGC
GCCGGGCACTGGCCTGGGCCTGTCCATCAGCCGCCGCCTCGTCGAATTGATGGGGGGAACGCTGGTCATGCGCAGCGCGC
CAGGGGTGGGCACAACGGTTTCGGTGGACCTGAGGCTGACCATGATCGAAAAATCCGCGCAGGCCACGCCGCCCGCTGCG
GCCGCTCAGGCCACGCCGTCCAAGCCGCAGGTATCGCTGCGCGTGCTGGTCGTCGATGACCACAAGCCCAACCTGATGCT
GCTGCGCCAGCAGCTGGACTACCTGGGCCAGCGTGTCGTCGCCGCCGACTCCGGCGAAGCCGCCCTGGCCCTGTGGCACG
AGCATGCGTTCGACGTCGTGATCACCGATTGCAACATGCCCGGTATCAACGGCTACGAATTGGCGCGCCGCATACGCGCC
GCCGAGGCCGCGCCCGGTTACGGACGTACGCGGTGCATTCTGTTCGGCTTCACGGCTTCGGCGCAGATGGACGAAGTGCA
GCGCTGCCGCGCCGCCGGCATGGACGACTGCCTGTTCAAGCCGATCGGCGTGGACGCCTTGCGGCAACGCTTGAACGAAG
CCGCGGCACGGGCCGCGCTCCCCACGCCCCCCTCGCCCCAGGCTGCCGCGCCGGCCACGCACGACGCCACCCCGGCGGCG
TTCTCGGCCGAGTCGATTCTTGCCCTGACGCAGAACGATGAGGCGCTCATCCGGCAATTGCTCGAAGAAGTGATTCGCAC
CAACCGGGCGGACGTCGATCAATTGCAGAAGCTGCACCAGCAGGCCGATTGGCCGAAGGTCTCGGACATGGCGCACAGGC
TGGCCGGCGGCGCGCGCGTGGTCGATGCCAAGGCCATGATAGACACTGCGCTGGCGCTGGAGAAAAAAGCGCAAGGCCAG
GCTGGCCCCTCGCCCGAAATCGACGGCCTGGTACGTACGCTTGCGGCGCAGTCCGCCGCGCTGGAGACGCAACTACGCGC
CTGGCTGGAGCAACGGCCGCATCAAGGCCAGCCCTGA
>Bpp_B0267.seq
ATGCCCGCCCCGCACCGCCTGTACCCCCGCAGTCTGATCTGCCTGGCTCAGGCGCTATTGGCATGGGCTTTGCTGGCATG
GGCGCCCGCGCAGGCAAGCCAGGAGCTGACCCTGGTCGGCAAGGCTGCCGTTCCCGACGTCGAGATCGCGCTCGACGGCG
ACGACTGGCGCTGGCTGGCGCGCAAGCGGGTGCTGACGCTGGGCGTGTACGCGCCGGACATTCCCCCGTTCGACGTCACC
TATGACGAGCGCTACGAAGGCCTGACGGCCGACTACATGGCGATCATCGCACACAACCTGGGCGTCCAGGCAAAAGTGCT
GCGCTACCCCACGCGCGAGCAAGCCGTCGGCGCACTGGAAAGCGGACAGATCGACCTCATCGGCACCGTCAATGGCATCG
AGGGCCGGCTGCAGAGCCTGCGCCTGAGTGTTCCCTACGCGGCCGACCACCCGGTGCTGGTCATGCCCATCGGCGCGCGC
CGCGCTCCGCCCGCGGACCTGGCAGGCCAGCGGTTGGCGGTTGACGCCAACTACTTGCCCAGGGAAACGCTGCAGCAGGC
CTATCCCCAGGCAACGCTGCATTACTTCCCATCGTCCGAACAGGCGCTGGCCGCGGTGGCCTATGGACAAGCCGACGTGT
TCATCGGCGATGCGCTGACCACCTCGCACCTCGTTTCGCAAAGCTACTTCAACGACGTTCGCGTCGTCGCCCCGGCCCAG
ATCGTGACGGGCGGGGAATCCTTCGGCGTGCGCGCCGACAATACCCGCCTGCTGCGGGTGGTCAATGCCGTACTCGAAGC
CATTCCGGCCTCCGAGCGCCGCAGCCTGATCTACCGCTGGGGCCTGGGCAGCAGCATTTCGCTCGATTTCGCGCGCCCCG
CCTATTCAGCGCGCGAGCAGCAGTGGATGGCAAACCATCCGGTCGTCAAGGTGGCGGTCCTGAACCTGTTCGCGCCCTTC
ACCCTGTTTCGCACCGATGAACAGTTCGGCGGCATCAGTGCCGCCGTGCTGCAACTGCTGCAGTTGCGCACCGGCCTGGA
TTTCCAGATCATCGGCGTCGACACGGTCGAGGAGCTGATCGCCAAGCTGCGCTCGGGCGAAGCCGACATGGCCGGCGCCC
TGTTCGTCAATGCCGCGCGGGAATCCGTCCTCAGCTTCAGCCGGCCGTATGTGCGCAATGGCATGGTGATCGTCACGCGC
CAGGACCCCGCCGCGCCCGCCGACGCCGATCACCTCGACGGCCGCACGATTGCGATGGTGCGCAACAGCGCCGCCATCCC
GCTCCTGCAGCAGCGCTATCCCCAGGCGAAGGTCGTGACCGCCGACAACCCGACCGAAGCCATGCTGCTGGTGGCCGATG
GCCAGGCCGACGCCGTCGTGCAGACGCAGATCAGCGCCAGCTACTACGTCAACCGCTACTTCGCCGGAAAACTGCGCATT
GCCTCGGCGCTGGACCTGCCGCCGGCCGAGATCGCGCTGGCGACGGCGCGCGGCCAGACCGAGCTGATATCCATCCTGAA
CAAGGCGCTCTACAGCATTTCGAACGACGAACTCGCCTCCATCGTCAGCCGCTGGCGCGGCAGCGACGGCGATCCGCGCA
CCTGGTACGCCTACCGCAACGAGATCTACCTGCTGATCGGGCTGGGCCTGTTGTCGGCCCTGCTGTTCCTGAGCTGGATC
GTCTACCTGCGGCGCCAGATCCGCCAGCGCAAGCGGGCCGAGCGGGCGCTGAACGACCAGCTGGAATTCATGCGCGTGCT
CATCGACGGCACGCCCAACCCCATCTATGTGCGCGATAAGGAAGGCCGCATGCTGTTGTGCAATGACGCCTACCTCGACA
CCTTTGGCGTGACTGCCGATGCGGTACTGGGCAAGACCATCCCGGAGGCCAACGTGGTGGGCGACCCGGCGCTGGCTCGC
GAGATGCACGAGTTCCTGCTCACGCGCATGTCCGCCGAGCGCGAGCCGCGCTTCGAGGACCGCGATGTCACGCTGCACGG
CCGCACCCGCCATGTCTACCAGTGGACGGTTCCGTACGGCGACTCGCTGGGCGAACTCAAGGGCATCATCGGCGGCTGGA
TCGACATTACCGAACGCGCCGAGCTGCTGCGCGAGCTGCACGACGCCAAGGAAAGCGCCGACGCCGCCAACCGGGCCAAG
ACCACGTTCCTGGCAACGATGAGCCACGAGATCCGCACGCCGATGAACGCGATCATCGGCATGCTGGAGCTGGCGCTGCT
CCGTCCGGCCGACCAGGAGCCGGACCGCCAGTCCATCCAGGTCGCGTACGACTCGGCCCGCAGCCTGCTGGAGCTGATAG
GCGACATCCTGGACATTGCGAAGATCGAGGCGGGAAAATTCGACCTGGCGCCGGTGCGCACGGCGCTGCGCGCCCTACCC
GAAGGGGCGATCCGCGTCTTCGACGGGTTGGCGCGCCAGAAAGGCATAGAGCTGGTATTGAAGACCGACATCGTGGGCGT
GGACGATGTATTGATAGACCCCTTGCGCATGAAGCAAGTGCTCTCGAACCTGGTGGGCAACGCCATCAAGTTCACCACCG
AAGGCCAGGTTGTCCTTACCGTGACCGCGCGCCCCGACGGCGAGGCCGCGCACGTGCAGTTCAGCGTGAGCGACACCGGC
TGCGGCATCAGCGAGGCCGACCAACGGCAGCTGTTCAAACCGTTCTCGCAGGTGGGCGGCAGCGCCGAGGCCGGGCCGGC
GCCGGGCACTGGCCTGGGCCTGTCCATCAGCCGCCGCCTCGTCGAATTGATGGGGGGAACGCTGGTCATGCGCAGCGCGC
CAGGGGTGGGCACAACGGTTTCGGTGGACCTGAGGCTGACCATGGTCGAAAAATCCGCGCAGGCCACGCCGCCCGCTGCG
GCCGCTCAGGCCACGCCATCCAAGCCGCAGGTATCGCTGCGCGTGCTGGTCGTCGATGACCACAAGCCCAACCTGATGCT
GCTGCGCCAGCAGCTGGACTACCTGGGCCAGCGTGTCGTCGCCGCCGACTCCGGCGAAGCCGCCCTGGCCCTGTGGCACG
AGCATGCGTTCGACGTCGTGATCACCGATTGCAACATGCCCGGTATCAACGGCTACGAATTGGCGCGCCGCATACGCGCC
GCCGAGGCCGCGCCCGGTTACGGACGTACGCGGTGCATTCTGTTCGGCTTCACGGCTTCGGCGCAGATGGACGAAGCGCA
GCGCTGCCGCGCCGCCGGCATGGACGACTGCCTGTTCAAGCCGATCGGCGTGGACGCCTTGCGGCAACGCTTGAACGAAG
CCGCGGCACGGGCCGCGCTCCCCACGCCCCCCTCGCCCCAGGCTGCCGCGCCGGCCACGCACGACGCCACCCCGGCGGCG
TTCTCGGCCGAGTCGATTCTTGCCCTGACGCAGAACGATGAGGCGCTCATCCGGCAATTGCTCGAAGAACTGATTCGCAC
CAACCGGGCGGACGTCGATCAATTGCAGAAGCTGCACCAGCAGGCCGATTGGCCGAAGGTCTCGGACATGGCGCACAGGC
TGGCCGGCGGCGCGCGCGTGGTCGATGCCAAGGCCATGATAGACACTGCGCTGGCGCTGGAAAAAAAAGCGCAAGGCCAG
GCTGGCCCCTCGCCCGAAATCGACGGCATGGTACGTACGCTTGCGGCGCAGTCCGCCGCGCTGGAGACGCAACTACGCGC
CTGGCTGGAGCAACGGCCGCATCAAGGCCAGCCCTGA
>Bpp_B0273.seq
ATGCCCGCCCCGCACCGCCTGTACCCCCGCAGTCTGATCTGCCTGGCTCAGGCGCTATTGGCATGGGCTTTGCTGGCATG
GGCGCCCGCGCAGGCAAGCCAGGAGCTGACCCTGGTCGGCAAGGCTGCCGTTCCCGACGTCGAGATCGCGCTCGACGGCG
ACGACTGGCGCTGGCTGGCGCGCAAGCGGGTGCTGACGCTGGGCGTGTACGCGCCGGACATTCCCCCGTTCGACGTCACC
TATGACGAGCGCTACGAAGGCCTGACGGCCGACTACATGGCGATCATCGCACACAACCTGGGCGTCCAGGCAAAAGTGCT
GCGCTACCCCACGCGCGAGCAAGCCGTCGGCGCACTGGAAAGCGGACAGATCGACCTCATCGGCACCGTCAATGGCATCG
AGGGCCGGCTGCAGAGCCTGCGCCTGAGTGTTCCCTACGCGGCCGACCACCCGGTGCTGGTCATGCCCATCGGCGCGCGC
CGCGCTCCGCCCGCGGACCTGGCAGGCCAGCGGTTGGCGGTTGACGCCAACTACTTGCCCAGGGAAACGCTGCAGCAGGC
CTATCCCCAGGCAACGCTGCATTACTTCCCATCGTCCGAACAGGCGCTGGCCGCGGTGGCCTATGGACAAGCCGACGTGT
TCATCGGCGATGCGCTGACCACCTCGCACCTCGTTTCGCAAAGCTACTTCAACGACGTTCGCGTCGTCGCCCCGGCCCAG
ATCGTGACGGGCGGGGAATCCTTCGGCGTGCGCGCCGACAATACCCGCCTGCTGCGGGTGGTCAATGCCGTACTCGAAGC
CATTCCGGCCTCCGAGCGCCGCAGCCTGATCTACCGCTGGGGCCTGGGCAGCAGCATTTCGCTCGATTTCGCGCGCCCCG
CCTATTCAGCGCGCGAGCAGCAGTGGATGGCAAACCATCCGGTCGTCAAGGTGGCGGTCCTGAACCTGTTCGCGCCCTTC
ACCCTGTTTCGCACCGATGAACAGTTCGGCGGCATCAGTGCCGCCGTGCTGCAACTGCTGCAGTTGCGCACCGGCCTGGA
TTTCCAGATCATCGGCGTCGACACGGTCGAGGAGCTGATCGCCAAGCTGCGCTCGGGCGAAGCCGACATGGCCGGCGCCC
TGTTCGTCAATGCCGCGCGGGAATCCGTCCTCAGCTTCAGCCGGCCGTATGTGCGCAATGGCATGGTGATCGTCACGCGC
CAGGACCCCGCCGCGCCCGCCGACGCCGATCACCTCGACGGCCGCACGATTGCGATGGTGCGCAACAGCGCCGCCATCCC
GCTCCTGCAGCAGCGCTATCCCCAGGCGAAGGTCGTGACCGCCGACAACCCGACCGAAGCCATGCTGCTGGTGGCCGATG
GCCAGGCCGACGCCGTCGTGCAGACGCAGATCAGCGCCAGCTACTACGTCAACCGCTACTTCGCCGGAAAACTGCGCATT
GCCTCGGCGCTGGACCTGCCGCCGGCCGAGATCGCGCTGGCGACGGCGCGCGGCCAGACCGAGCTGATATCCATCCTGAA
CAAGGCGCTCTACAGCATTTCGAACGACGAACTCGCCTCCATCGTCAGCCGCTGGCGCGGCAGCGACGGCGATCCGCGCA
CCTGGTACGCCTACCGCAACGAGATCTACCTGCTGATCGGGCTGGGCCTGTTGTCGGCCCTGCTGTTCCTGAGCTGGATC
GTCTACCTGCGGCGCCAGATCCGCCAGCGCAAGCGGGCCGAGCGGGCGCTGAACGACCAGCTGGAATTCATGCGCGTGCT
CATCGACGGCACGCCCAACCCCATCTATGTGCGCGATAAGGAAGGCCGCATGCTGTTGTGCAATGACGCCTACCTCGACA
CCTTTGGCGTGACTGCCGATGCGGTACTGGGCAAGACCATCCCGGAGGCCAACGTGGTGGGCGACCCGGCGCTGGCTCGC
GAGATGCACGAGTTCCTGCTCACGCGCATGTCCGCCGAGCGCGAGCCGCGCTTCGAGGACCGCGATGTCACGCTGCACGG
CCGCACCCGCCATGTCTACCAGTGGACGGTTCCGTACGGCGACTCGCTGGGCGAACTCAAGGGCATCATCGGCGGCTGGA
TCGACATTACCGAACGCGCCGAGCTGCTGCGCGAGCTGCACGACGCCAAGGAAAGCGCCGACGCCGCCAACCGGGCCAAG
ACCACGTTCCTGGCAACGATGAGCCACGAGATCCGCACGCCGATGAACGCGATCATCGGCATGCTGGAGCTGGCGCTGCT
CCGTCCGGCCGACCAGGAGCCGGACCGCCAGTCCATCCAGGTCGCGTACGACTCGGCCCGCAGCCTGCTGGAGCTGATAG
GCGACATCCTGGACATTGCGAAGATCGAGGCGGGAAAATTCGACCTGGCGCCGGTGCGCACGGCGCTGCGCGCCCTACCC
GAAGGGGCGATCCGCGTCTTCGACGGGTTGGCGCGCCAGAAAGGCATAGAGCTGGTATTGAAGACCGACATCGTGGGCGT
GGACGATGTATTGATAGACCCCTTGCGCATGAAGCAAGTGCTCTCGAACCTGGTGGGCAACGCCATCAAGTTCACCACCG
AAGGCCAGGTTGTCCTTACCGTGACCGCGCGCCCCGACGGCGAGGCCGCGCACGTGCAGTTCAGCGTGAGCGACACCGGC
TGCGGCATCAGCGAGGCCGACCAACGGCAGCTGTTCAAACCGTTCTCGCAGGTGGGCGGCAGCGCCGAGGCCGGGCCGGC
GCCGGGCACTGGCCTGGGCCTGTCCATCAGCCGCCGCCTCGTCGAATTGATGGGGGGAACGCTGGTCATGCGCAGCGCGC
CAGGGGTGGGCACAACGGTTTCGGTGGACCTGAGGCTGACCATGGTCGAAAAATCCGCGCAGGCCACGCCGCCCGCTGCG
GCCGCTCAGGCCACGCCATCCAAGCCGCAGGTATCGCTGCGCGTGCTGGTCGTCGATGACCACAAGCCCAACCTGATGCT
GCTGCGCCAGCAGCTGGACTACCTGGGCCAGCGTGTCGTCGCCGCCGACTCCGGCGAAGCCGCCCTGGCCCTGTGGCACG
AGCATGCGTTCGACGTCGTGATCACCGATTGCAACATGCCCGGTATCAACGGCTACGAATTGGCGCGCCGCATACGCGCC
GCCGAGGCCGCGCCCGGTTACGGACGTACGCGGTGCATTCTGTTCGGCTTCACGGCTTCGGCGCAGATGGACGAAGCGCA
GCGCTGCCGCGCCGCCGGCATGGACGACTGCCTGTTCAAGCCGATCGGCGTGGACGCCTTGCGGCAACGCTTGAACGAAG
CCGCGGCACGGGCCGCGCTCCCCACGCCCCCCTCGCCCCAGGCTGCCGCGCCGGCCACGCACGACGCCACCCCGGCGGCG
TTCTCGGCCGAGTCGATTCTTGCCCTGACGCAGAACGACGAGGCGCTGATCCGGCAATTGCTCGAAGAAGTGATTCGCAC
CAACCGGGCGGACGTCGATCAATTGCAGAAGCTGCACCAGCAGGCCGATTGGCCGAAGGTCTCGGACATGGCGCACAGGC
TGGCCGGCGGCGCGCGCGTGGTCGATGCCAAGGCCATGATAGACACTGCGCTGGCGCTGGAGAAAAAAGCGCAAGGCCAG
GCTGGCCCCTCGCCCGAAATCGACGGCCTGGTACGTACGCTTGCGGCGCAGTCCGCCGCGCTGGAGACGCAACTACGCGC
CTGGCTGGAGCAACGGCCGCATCAAGGCCAGCCCTGA
>Bpp_B0285.seq
ATGCCCGCCCCGCACCGCCTGTACCCCCGCAGTCTGATCTGCCTGGCTCAGGCGCTATTGGTATGGGCTTTGCTGGCATG
GGCGCCCGCGCAGGCAAGCCAGGAGCTGACCCTGGTCGGCAAGGCTGCCGTTCCCGACGTCGAGATCGCGCTCGACGGCG
ACGACTGGCGCTGGCTGGCGCGCAAGCGGGTGCTGACGCTGGGCGTGTACGCGCCGGACATTCCCCCGTTCGACGTCACC
TATGACGAGCGCTACGAAGGCCTGACGGCCGACTATATGGCGATCATCGCGCACAACCTGGGCGTCCAGGCAAAAGTGCT
GCGCTACCCCACGCGCGAGCAAGCCGTCGGCGCACTGGAAAGCGGACAGATCGACCTCATCGGCACCGTCAATGGCATCG
AGGGCCGGCTGCAGAGCCTGCGCCTGAGTGTTCCCTACGCGGCCGACCACCCGGTGCTGGTCATGCCCATCGGCGCGCGC
CGCGCTCCGCCCGCGGACCTGGCAGGCCAGCGGTTGGCGGTTGACGCCAACTACTTGCCCAGGGAAACGCTGCAGCAGGC
CTATCCCCAGGCAACGCTGCATTACTTCCCATCGTCCGAGCAGGCGCTGGCCGCGGTGGCCTATGGACAAGCCGACGTGT
TCATCGGCGATGCGCTGACCACCTCGCACCTCGTTTCGCAAAGCTACTTCAACGACGTTCGCGTCGTCGCCCCGGCCCAG
ATCGTGACGGGCGGGGAATCCTTCGGCGTGCGCGCCGACAATACCCGCCTGCTGCGGGTGGTCAATGCCGTGCTCGAAGC
CATTCCGGCCTCCGAGCGCCGCAGCCTGATCTACCGCTGGGGCCTGGGCAGCAGCATTTCGCTCGATTTCGCGCGCCCCG
CCTATTCAGCGCGCGAGCAGCAGTGGATGGCAAACCATCCGGTCGTCAAGGTGGCGGTCCTGAACCTGTTCGCGCCCTTC
ACCCTGTTTCGCACCGATGAACAGTTCGGCGGCATCAGTGCCGCCGTGCTGCAACTGCTGCAGTTGCGCACCGGCCTGGA
TTTCCAGATCATCGGCGTCGACACGGTCGAGGAGCTGATCGCCAAGCTGCGCTCGGGCGAAGCCGACATGGCCGGCGCCC
TGTTCGTCAATGCCGCGCGGGAATCCGTCCTCAGCTTCAGCCGGCCGTATGTGCGCAATGGCATGGTGATCGTCACGCGC
CAGGACCCCGCCGCGCCCGCCGACGCCGATCACCTCGACGGCCGCACGATTGCGATGGTGCGCAACAGCGCCGCCATCCC
GCTCCTGCAGCAGCGCTATCCCCAGGCGAAGGTCGTGACCGCCGACAACCCGACCGAAGCCATGCTGCTGGTGGCCGATG
GCCAGGCCGACGCCGTCGTGCAGACGCAGATCAGCGCCAGCTACTACGTCAACCGCTACTTCGCCGGAAAACTGCGCATT
GCCTCGGCGCTGGACCTGCCGCCGGCCGAGATCGCGCTGGCGACGGCGCGCGGCCAGACCGAGCTGATATCCATCCTGAA
CAAGGCGCTCTACAGCATTTCGAACGACGAACTCGCCTCCATCGTCAGCCGCTGGCGCGGCAGCGACGGCGATCCGCGCA
CCTGGTACGCCTACCGCAACGAGATCTACCTGCTGATCGGGCTGGGCCTGTTGTCGGCCCTGCTGTTCCTGAGCTGGATC
GTCTACCTGCGGCGCCAGATCCGCCAGCGCAAGCGGGCCGAGCGGGCGCTGAACGACCAGCTGGAATTCATGCGCGTGCT
CATCGACGGCACGCCCAACCCCATCTATGTGCGCGATAAGGAAGGCCGCATGCTGTTGTGCAATGACGCCTACCTCGACA
CCTTTGGCGTGACTGCCGATGCGGTACTGGGCAAGACCATCCCGGAGGCCAACGTGGTGGGCGACCCGGCGCTGGCTCGC
GAGATGCACGAGTTCCTGCTCACGCGCATGGCCGCCGAGCGCGAGCCGCGCTTCGAGGACCGCGATGTCACGCTGCACGG
CCGCACCCGCCATGTCTACCAGTGGACGGTTCCGTACGGCGACTCGCTGGGCGAACTCAAGGGCATCATCGGCGGCTGGA
TCGACATTACCGAACGCGCCGAGCTGCTGCGCGAGCTGCACGACGCCAAGGAAAGCGCCGACGCCGCCAACCGGGCCAAG
ACCACGTTCCTGGCAACGATGAGCCACGAGATCCGCACGCCGATGAACGCGATCATCGGCATGCTGGAGCTGGCGCTGCT
CCGTCCGGCCGACCAGGAGCCGGACCGCCAGTCCATCCAGGTCGCGTACGACTCGGCCCGCAGCCTGCTGGAGCTGATAG
GCGACATCCTGGACATTGCGAAGATCGAGGCGGGAAAATTCGACCTGGCGCCGGTGCGCACGGCGCTGCGCGCCCTGCCC
GAAGGGGCGATCCGCCTCTTCGATGGATTGGCGCGCCAGAAAGGCATAGAGCTGGTATTGAAGACCGACATCGTGGGCGT
GGACGATGTATTGATAGACCCCTTGCGCATGAAGCAAGTGCTCTCGAACCTGGTGGGCAACGCCATCAAGTTCACCACCG
AAGGCCAGGTTGTCCTTACCGTAACCGCGCGCCCCGACGGCGAGGCCGCGCACGTGCAGTTCAGCGTGAGCGACACCGGC
TGCGGCATCAGCGAGGCCGACCAACGGCAGCTGTTCAAACCGTTCTCGCAGGTGGGTGGCAGCGCCGAGGCCGGGCCGGC
GCCGGGCACTGGCCTGGGCCTGTCCATCAGCCGCCGCCTCGTCGAATTGATGGGGGGAACGCTGGTCATGCGCAGCGCGC
CAGGCGTGGGCACAACGGTTTCGGTGGACCTGAGGCTGACCATGATCGAAAAATCCGCGCAGGCCACGCCGCCCGCTGCG
GCCGCTCAGGCCACGCCGTCCAAGCCGCAGGTATCGCTGCGCGTGCTGGTCGTCGATGACCACAAGCCCAACCTGATGCT
GCTGCGCCAGCAGCTGGACTACCTGGGCCAGCGTGTCGTCGCCGCCGACTCCGGCGAAGCCGCCCTGGCCCTGTGGCACG
AGCATGCGTTCGACGTCGTGATCACCGATTGCAACATGCCCGGTATCAACGGCTACGAATTGGCGCGCCGCATACGCGCC
GCCGAGGCCGCGCCCGGTTACGGACGTACGCGGTGCATTCTGTTCGGCTTCACGGCTTCGGCGCAGATGGACGAAGCGCA
GCGCTGCCGCGCCGCCGGCATGGACGACTGCCTGTTCAAGCCGATCGGCGTGGACGCCTTGCGGCAACGCTTGAACGAAG
CCGCGGCACGGGCCGCGCTCCCCACGCCCCCCTCGCCCCAGGCTGCCGCGCCGGCCACGCACGACGCCACCCCGGCGGCG
TTCTCGGCCGAGTCGATTCTTGCCCTGACGCAGAACGACGAGGCGCTGATCCGGCAATTGCTCGAAGAAGTGATTCGCAC
CAACCGGGCGGACGTCGATCAATTGCAGAAGCTGCACCAGCAGGCCGATTGGCCGAAGGTCTCGGACATGGCGCACAGGC
TGGCCGGCGGCGCGCGCGTGGTCGATGCCAAGGCCATGATAGACACTGCGCTGGCGCTGGAGAAAAAAGCGCAAGGCCAG
GCTGGCCCCTCGCCCGAAATCGACGGCCTGGTACGTACGCTTGCGGCGCAGTCCGCCGCGCTGGAGACGCAACTACGCGC
CTGGCTGGAGCAACGGCCGCATCAAGGCCAGCCCTGA
>Bpp_B0291.seq
ATGCCCGCCCCGCACCGCCTGTACCCCCGCAGTCTGATCTGCCTGGCTCAGGCGCTATTGGTATGGGCTTTGCTGGCATG
GGCGCCCGCGCAGGCAAGCCAGGAGCTGACCCTGGTCGGCAAGGCTGCCGTTCCCGACGTCGAGATCGCGCTCGACGGCG
ACGACTGGCGCTGGCTGGCGCGCAAGCGGGTGCTGACGCTGGGCGTGTACGCGCCGGACATTCCCCCGTTCGACGTCACC
TATGACGAGCGCTACGAAGGCCTGACGGCCGACTATATGGCGATCATCGCGCACAACCTGGGCGTCCAGGCAAAAGTGCT
GCGCTACCCCACGCGCGAGCAAGCCGTCGGCGCACTGGAAAGCGGACAGATCGACCTCATCGGCACCGTCAATGGCATCG
AGGGCCGGCTGCAGAGCCTGCGCCTGAGTGTTCCCTACGCGGCCGACCACCCGGTGCTGGTCATGCCCATCGGCGCGCGC
CGCGCTCCGCCCGCGGACCTGGCAGGCCAGCGGTTGGCGGTTGACGCCAACTACTTGCCCAGGGAAACGCTGCAGCAGGC
CTATCCCCAGGCAACGCTGCATTACTTCCCATCGTCCGAGCAGGCGCTGGCCGCGGTGGCCTATGGACAAGCCGACGTGT
TCATCGGCGATGCGCTGACCACCTCGCACCTCGTTTCGCAAAGCTACTTCAACGACGTTCGCGTCGTCGCCCCGGCCCAG
ATCGTGACGGGCGGGGAATCCTTCGGCGTGCGCGCCGACAATACCCGCCTGCTGCGGGTGGTCAATGCCGTGCTCGAAGC
CATTCCGGCCTCCGAGCGCCGCAGCCTGATCTACCGCTGGGGCCTGGGCAGCAGCATTTCGCTCGATTTCGCGCGCCCCG
CCTATTCAGCGCGCGAGCAGCAGTGGATGGCAAACCATCCGGTCGTCAAGGTGGCGGTCCTGAACCTGTTCGCGCCCTTC
ACCCTGTTTCGCACCGATGAACAGTTCGGCGGCATCAGTGCCGCCGTGCTGCAACTGCTGCAGTTGCGCACCGGCCTGGA
TTTCCAGATCATCGGCGTCGACACGGTCGAGGAGCTGATCGCCAAGCTGCGCTCGGGCGAAGCCGACATGGCCGGCGCCC
TGTTCGTCAATGCCGCGCGGGAATCCGTCCTCAGCTTCAGCCGGCCGTATGTGCGCAATGGCATGGTGATCGTCACGCGC
CAGGACCCCGCCGCGCCCGCCGACGCCGATCACCTCGACGGCCGCACGATTGCGATGGTGCGCAACAGCGCCGCCATCCC
GCTCCTGCAGCAGCGCTATCCCCAGGCGAAGGTCGTGACCGCCGACAACCCGACCGAAGCCATGCTGCTGGTGGCCGATG
GCCAGGCCGACGCCGTCGTGCAGACGCAGATCAGCGCCAGCTACTACGTCAACCGCTACTTCGCCGGAAAACTGCGCATT
GCCTCGGCGCTGGACCTGCCGCCGGCCGAGATCGCGCTGGCGACGGCGCGCGGCCAGACCGAGCTGATATCCATCCTGAA
CAAGGCGCTCTACAGCATTTCGAACGACGAACTCGCCTCCATCGTCAGCCGCTGGCGCGGCAGCGACGGCGATCCGCGCA
CCTGGTACGCCTACCGCAACGAGATCTACCTGCTGATCGGGCTGGGCCTGTTGTCGGCCCTGCTGTTCCTGAGCTGGATC
GTCTACCTGCGGCGCCAGATCCGCCAGCGCAAGCGGGCCGAGCGGGCGCTGAACGACCAGCTGGAATTCATGCGCGTGCT
CATCGACGGCACGCCCAACCCCATCTATGTGCGCGATAAGGAAGGCCGCATGCTGTTGTGCAATGACGCCTACCTCGACA
CCTTTGGCGTGACTGCCGATGCGGTACTGGGCAAGACCATCCCGGAGGCCAACGTGGTGGGCGACCCGGCGCTGGCTCGC
GAGATGCACGAGTTCCTGCTCACGCGCATGGCCGCCGAGCGCGAGCCGCGCTTCGAGGACCGCGATGTCACGCTGCACGG
CCGCACCCGCCATGTCTACCAGTGGACGGTTCCGTACGGCGACTCGCTGGGCGAACTCAAGGGCATCATCGGCGGCTGGA
TCGACATTACCGAACGCGCCGAGCTGCTGCGCGAGCTGCACGACGCCAAGGAAAGCGCCGACGCCGCCAACCGGGCCAAG
ACCACGTTCCTGGCAACGATGAGCCACGAGATCCGCACGCCGATGAACGCGATCATCGGCATGCTGGAGCTGGCGCTGCT
CCGTCCGGCCGACCAGGAGCCGGACCGCCAGTCCATCCAGGTCGCGTACGACTCGGCCCGCAGCCTGCTGGAGCTGATAG
GCGACATCCTGGACATTGCGAAGATCGAGGCGGGAAAATTCGACCTGGCGCCGGTGCGCACGGCGCTGCGCGCCCTGCCC
GAAGGGGCGATCCGCCTCTTCGATGGATTGGCGCGCCAGAAAGGCATAGAGCTGGTATTGAAGACCGACATCGTGGGCGT
GGACGATGTATTGATAGACCCCTTGCGCATGAAGCAAGTGCTCTCGAACCTGGTGGGCAACGCCATCAAGTTCACCACCG
AAGGCCAGGTTGTCCTTACCGTAACCGCGCGCCCCGACGGCGAGGCCGCGCACGTGCAGTTCAGCGTGAGCGACACCGGC
TGCGGCATCAGCGAGGCCGACCAACGGCAGCTGTTCAAACCGTTCTCGCAGGTGGGTGGCAGCGCCGAGGCCGGGCCGGC
GCCGGGCACTGGCCTGGGCCTGTCCATCAGCCGCCGCCTCGTCGAATTGATGGGGGGAACGCTGGTCATGCGCAGCGCGC
CAGGCGTGGGCACAACGGTTTCGGTGGACCTGAGGCTGACCATGATCGAAAAATCCGCGCAGGCCACGCCGCCCGCTGCG
GCCGCTCAGGCCACGCCGTCCAAGCCGCAGGTATCGCTGCGCGTGCTGGTCGTCGATGACCACAAGCCCAACCTGATGCT
GCTGCGCCAGCAGCTGGACTACCTGGGCCAGCGTGTCGTCGCCGCCGACTCCGGCGAAGCCGCCCTGGCCCTGTGGCACG
AGCATGCGTTCGACGTCGTGATCACCGATTGCAACATGCCCGGTATCAACGGCTACGAATtGGCGCGCCGCATACGCGCC
GCCGAGGCCGCGCCCGGTTACGGACGTACGCGGTGCATTCTGTTCGGCTTCACGGCTTCGGCGCAGATGGACGAAGCGCA
GCGCTGCCGCGCCGCCGGCATGGACGACTGCCTGTTCAAGCCGATCGGCGTGGACGCCTTGCGGCAACGCTTGAACGAAG
CCGCGGCACGGGCCGCGCTCCCCACGCCCCCCTCGCCCCAGGCTGCCGCGCCGGCCACGCACGACGCCACCCCGGCGGCG
TTCTCGGCCGAGTCGATTCTTGCCCTGACGCAGAACGACGAGGCGCTGATCCGGCAATTGCTCGAAGAAGTGATTCGCAC
CAACCGGGCGGACGTCGATCAATTGCAGAAGCTGCACCAGCAGGCCGATTGGCCGAAGGTCTCGGACATGGCGCACAGGC
TGGCCGGCGGCGCGCGCGTGGTCGATGCCAAGGCCATGATAGACACTGCGCTGGCGCTGGAGAAAAAAGCGCAAGGCCAG
GCTGGCCCCTCGCCCGAAATCGACGGCCTGGTACGTACGCTTGCGGCGCAGTCCGCCGCGCTGGAGACGCAACTACGCGC
CTGGCTGGAGCAACGGCCGCATCAAGGCCAGCCCTGA
