# Supplementary material for: Molecular Evolution of the Two-Component System BvgAS Involved in Virulence Regulation in Bordetella
Source: PLoS One. 2009 Sep 14;4(9):e6996. doi: 10.1371/journal.pone.0006996 (PMC2737282; doi:10.1371/journal.pone.0006996)
Supplement: Table S3 — bvgA sequences (0.04 MB RTF) [file pone.0006996.s003.rtf]

>Bb_B0230
ATGTACAACAAAGTCCTCATCATTGACGATCACCCTGTACTGAGATTCGCCGTCCGGGTCCTGATGGAAAAGGAAGGATT
CGAAGTCATCGGCGAAACCGATAACGGTATTGACGGGCTCAAGATCGCCCGCGAGAAAATTCCCAACCTCGTCGTACTCG
ACATCGGCATTCCCAAGCTGGACGGGCTGGAAGTCATCGCCCGGCTGCAATCGCTGGGGTTGCCGCTGCGCGTTCTGGTG
CTGACCGGCCAGCCGCCTTCGCTGTTCGCCCGCCGCTGCCTGAACTCCGGCGCCGCAGGCTTCGTGTGCAAACACGAGAA
CCTGCACGAGGTCATCAATGCCGCCAAGGCGGTGATGGCCGGCTACACCTACTTCCCCAGCACCACGCTCAGCGAGATGC
GCATGGGCGACAACGCCAAGAGCGACAGTACGCTCATCAGCGTGTTGTCCAACCGCGAACTGACCGTCCTGCAACTGCTG
GCGCAAGGCATGTCCAACAAGGACATCGCCGACAGCATGTTCCTCAGCAACAAGACCGTCAGCACCTACAAGACGCGCCT
GCTGCAGAAGCTGAACGCCACGTCGCTGGTGGAACTGATAGACCTCGCCAAACGCAACAATCTCGCCTAG
>Bb_B0231
ATGTACAACAAAGTCCTCATCATTGACGATCACCCTGTACTGAGATTCGCCGTCCGGGTCCTGATGGAAAAGGAAGGATT
CGAAGTCATCGGCGAAACCGATAACGGTATCGACGGGCTCAAGATCGCCCGCGAGAAAATTCCCAACCTCGTCGTACTCG
ACATCGGCATTCCCAAGCTGGACGGGCTGGAAGTCATCGCCCGGCTGCAATCGCTGGGGTTGCCGCTGCGCGTTCTGGTG
CTGACCGGCCAGCCGCCTTCGCTGTTCGCCCGCCGCTGCCTGAACTCCGGCGCCGCAGGCTTCGTGTGCAAACACGAGAA
CCTGCACGAGGTCATCAATGCCGCCAAGGCGGTGATGGCCGGCTACACCTACTTCCCCAGCACCACGCTCAGCGAGATGC
GCATGGGCGACAACGCCAAGAGCGACAGTACGCTCATCAGCGTGTTGTCCAACCGCGAACTGACCGTCCTGCAACTGCTG
GCGCAAGGCATGTCCAACAAGGACATCGCCGACAGCATGTTCCTCAGCAACAAGACCGTCAGCACCTACAAGACGCGCCT
GCTGCAGAAGCTGAACGCCACGTCGCTGGTGGAACTGATAGACCTCGCCAAACGCAACAATCTCGCCTAG
>Bb_B0232
ATGTACAACAAAGTCCTCATCATTGACGATCACCCTGTACTGAGATTCGCCGTCCGGGTCCTGATGGAAAAGGAAGGATT
CGAAGTCATCGGCGAAACCGATAACGGCATTGACGGGCTCAAGATCGCCCGCGAGAAAATTCCCAACCTCGTCGTACTCG
ACATCGGCATTCCCAAGCTGGACGGGCTGGAAGTCATCGCCCGGCTGCAATCGCTGGGGTTGCCGCTGCGCGTGCTGGTG
CTGACCGGCCAGCCGCCTTCGCTGTTCGCCCGCCGCTGCCTGAACTCCGGCGCCGCAGGCTTCGTGTGCAAACACGAGAA
CCTGCACGAGGTCATCAATGCCGCCAAGGCGGTGATGGCCGGCTACACCTACTTCCCCAGCACCACGCTCAGCGAGATGC
GCATGGGCGACAACGCCAAGAGCGACAGTACGCTCATCAGCGTGTTGTCCAACCGCGAACTGACCGTCCTGCAACTGCTG
GCGCAAGGCATGTCCAACAAGGACATCGCGGACAGCATGTTCCTCAGCAACAAGACCGTCAGCACCTACAAGACGCGCCT
GCTGCAGAAGCTGAACGCCACGTCGCTGGTGGAACTGATAGACCTCGCCAAACGCAACAATCTCGCCTAG
>Bb_B0236
ATGTACAACAAAGTCCTCATCATTGACGATCACCCTGTACTGAGATTCGCCGTCCGGGTCCTGATGGAAAAGGAAGGATT
CGAAGTCATCGGCGAAACCGATAACGGTATCGACGGGCTCAAGATCGCCCGCGAGAAAATTCCCAACCTCGTCGTACTCG
ACATCGGCATTCCCAAGCTGGACGGGCTGGAAGTCATCGCCCGGCTGCAATCGCTGGGGTTGCCGCTACGCGTTCTGGTG
CTGACCGGCCAGCCGCCTTCGCTGTTCGCCCGCCGCTGCCTGAACTCCGGCGCCGCAGGCTTCGTGTGCAAACACGAGAA
CCTGCACGAGGTCATCAATGCCGCCAAGGCGGTGATGGCCGGCTACACCTACTTCCCCAGCACCACGCTCAGCGAGATGC
GCATGGGCGACAACGCCAAGAGCGACAGTACGCTCATCAGCGTGTTGTCCAACCGCGAACTGACCGTCCTGCAACTGCTG
GCGCAAGGCATGTCCAACAAGGACATCGCCGACAGCATGTTCCTCAGCAACAAGACCGTCAGCACCTACAAGACGCGCCT
GCTGCAGAAGCTGAACGCCACGTCGCTGGTGGAACTGATAGACCTCGCCAAACGCAACAATCTCGCCTAG
>Bb_B0243
ATGTACAACAAAGTCCTCATCATTGACGATCACCCTGTACTGAGATTCGCCGTCCGGGTCCTGATGGAAAAGGAAGGATT
CGAAGTCATCGGCGAAACCGATAACGGCATTGACGGGCTCAAGATCGCCCGCGAGAAAATTCCCAACCTCGTCGTACTCG
ACATCGGCATTCCCAAGCTGGACGGGCTGGAAGTCATCGCCCGGCTGCAATCGCTGGGGTTGCCGCTGCGCGTGCTGGTG
CTGACCGGCCAGCCGCCTTCGCTGTTCGCCCGCCGCTGCCTGAACTCCGGCGCCGCAGGCTTCGTGTGCAAACACGAGAA
CCTGCACGAGGTCATCAATGCCGCCAAGGCGGTGATGGCCGGCTACACCTACTTCCCCAGCACCACGCTCAGCGAGATGC
GCATGGGCGACAACGCCAAGAGCGACAGTACGCTCATCAGCGTGTTGTCCAACCGCGAACTGACCGTCCTGCAACTGCTG
GCGCAAGGCATGTCCAACAAGGACATCGCGGACAGCATGTTCCTCAGCAACAAGACCGTCAGCACCTACAAGACGCGCCT
GCTGCAGAAGCTGAACGCCACGTCGCTGGTGGAACTGATAGACCTCGCCAAACGCAACAATCTCGCCTAG
>Bb_B0246
ATGTACAACAAAGTCCTCATCATTGACGATCACCCTGTACTGAGATTCGCCGTCCGGGTCCTGATGGAAAAGGAAGGATT
CGAAGTCATCGGCGAAACCGATAACGGTATCGACGGGCTCAAGATCGCCCGCGAGAAAATTCCCAACCTCGTCGTACTCG
ACATCGGCATTCCCAAGCTGGACGGGCTGGAAGTCATCGCCCGGCTGCAATCGCTGGGGTTGCCGCTGCGCGTTCTGGTG
CTGACCGGCCAGCCGCCTTCGCTGTTCGCCCGCCGCTGCCTGAACTCCGGCGCCGCAGGCTTCGTGTGCAAACACGAGAA
CCTGCACGAGGTCATCAATGCCGCCAAGGCGGTGATGGCCGGCTACACCTACTTCCCCAGCACCACGCTCAGCGAGATGC
GCATGGGCGACAACGCCAAGAGCGACAGTACGCTCATCAGCGTGTTGTCCAACCGCGAACTGACCGTCCTGCAACTGCTG
GCGCAAGGCATGTCCAACAAGGACATCGCCGACAGCATGTTCCTCAGCAACAAGACCGTCAGCACCTACAAGACGCGCCT
GCTGCAGAAGCTGAACGCCACGTCGCTGGTGGAACTGATAGACCTCGCCAAACGCAACAATCTCGCCTAG
>Bb_B0247
ATGTACAACAAAGTCCTCATCATTGACGATCACCCTGTACTGAGATTCGCCGTCCGGGTCCTGATGGAAAAGGAAGGATT
CGAAGTCATCGGCGAAACCGATAACGGTATCGACGGGCTCAAGATCGCCCGCGAGAAAATTCCCAACCTCGTCGTACTCG
ACATCGGCATTCCCAAGCTGGACGGGCTGGAAGTCATCGCCCGGCTGCAATCGCTGGGGTTGCCGCTGCGCGTTCTGGTG
CTGACCGGCCAGCCGCCTTCGCTGTTCGCCCGCCGCTGCCTGAACTCCGGCGCCGCAGGCTTCGTGTGCAAACACGAGAA
CCTGCACGAGGTCATCAATGCCGCCAAGGCGGTGATGGCCGGCTACACCTACTTCCCCAGCACCACGCTCAGCGAGATGC
GCATGGGCGACAACGCCAAGAGCGACAGTACGCTCATCAGCGTGTTGTCCAACCGCGAACTGACCGTCCTGCAACTGCTG
GCGCAAGGCATGTCCAACAAGGACATCGCCGACAGCATGTTCCTCAGCAACAAGACCGTCAGCACCTACAAGACGCGCCT
GCTGCAGAAGCTGAACGCCACGTCGCTGGTGGAACTGATAGACCTCGCCAAACGCAACAATCTCGCCTAG
>Bb_B0260
ATGTACAACAAAGTCCTCATCATTGACGATCACCCTGTACTGAGATTCGCCGTCCGGGTCCTGATGGAAAAGGAAGGATT
CGAAGTCATCGGCGAAACCGATAACGGTATCGACGGGCTCAAGATCGCCCGCGAGAAAATTCCCAACCTCGTCGTACTCG
ACATCGGCATTCCCAAGCTGGACGGGCTGGAAGTCATCGCCCGGCTGCAATCGCTGGGGTTGCCGCTGCGCGTTCTGGTG
CTGACCGGCCAGCCGCCTTCGCTGTTCGCCCGCCGCTGCCTGAACTCCGGCGCCGCAGGCTTCGTGTGCAAACACGAGAA
CCTGCACGAGGTCATCAATGCCGCCAAGGCGGTGATGGCCGGCTACACCTACTTCCCCAGCACCACGCTCAGCGAGATGC
GCATGGGCGACAACGCCAAGAGCGACAGTACGCTCATCAGCGTGTTGTCCAACCGCGAACTGACCGTCCTGCAACTGCTG
GCGCAAGGCATGTCCAACAAGGACATCGCCGACAGCATGTTCCTCAGCAACAAGACCGTCAGCACCTACAAGACGCGCCT
GCTGCAGAAGCTGAACGCCACGTCGCTGGTGGAACTGATAGACCTCGCCAAACGCAACAATCTCGCCTAG
>Bb_B0188
ATGTACAACAAAGTCCTCATCATTGACGATCACCCTGTACTGAGATTCGCCGTCCGGGTCCTGATGGAAAAGGAAGGATT
CGAAGTCATCGGCGAAACCGATAACGGTATCGACGGGCTCAAGATCGCCCGCGAGAAAATTCCCAACCTCGTCGTACTCG
ACATCGGCATTCCCAAGCTGGACGGGCTGGAAGTCATCGCCCGGCTGCAATCGCTGGGGTTGCCGCTGCGCGTTCTGGTG
CTGACCGGCCAGCCGCCTTCGCTGTTCGCCCGCCGCTGCCTGAACTCCGGCGCCGCAGGCTTCGTGTGCAAACACGAGAA
CCTGCACGAGGTCATCAATGCCGCCAAGGCGGTGATGGCCGGCTACACCTACTTCCCCAGCACCACGCTCAGCGAGATGC
GCATGGGCGACAACGCCAAGAGCGACAGTACGCTCATCAGCGTGTTGTCCAACCGCGAACTGACCGTCCTGCAACTGCTG
GCGCAAGGCATGTCCAACAAGGACATCGCCGACAGCATGTTCCTCAGCAACAAGACCGTCAGCACCTACAAGACGCGCCT
GCTGCAGAAGCTGAACGCCACGTCGCTGGTGGAACTGATAGACCTCGCCAAACGCAACAATCTCGCCTAG
>Bb_B0189
ATGTACAACAAAGTCCTCATCATTGACGATCACCCTGTACTGAGATTCGCCGTCCGGGTCCTGATGGAAAAGGAAGGATT
CGAAGTCATCGGCGAAACCGATAACGGTATCGACGGGCTCAAGATCGCCCGCGAGAAAATTCCCAACCTCGTCGTACTCG
ACATCGGCATTCCCAAGCTGGACGGGCTGGAAGTCATCGCCCGGCTGCAATCGCTGGGGTTGCCGCTGCGCGTTCTGGTG
CTGACCGGCCAGCCGCCTTCGCTGTTCGCCCGCCGCTGCCTGAACTCCGGCGCCGCAGGCTTCGTGTGCAAACACGAGAA
CCTGCACGAGGTCATCAATGCCGCCAAGGCGGTGATGGCCGGCTACACCTACTTCCCCAGCACCACGCTCAGCGAGATGC
GCATGGGCGACAACGCCAAGAGCGACAGTACGCTCATCAGCGTGTTGTCCAACCGCGAACTGACCGTCCTGCAACTGCTG
GCGCAAGGCATGTCCAACAAGGACATCGCCGACAGCATGTTCCTCAGCAACAAGACCGTCAGCACCTACAAGACGCGCCT
GCTGCAGAAGCTGAACGCCACGTCGCTGGTGGAACTGATAGACCTCGCCAAACGCAACAATCTCGCCTAG
>Bb_B0223
ATGTACAACAAAGTCCTCATCATTGACGATCACCCTGTACTGAGATTCGCCGTCCGGGTCCTGATGGAAAAGGAAGGATT
CGAAGTCATCGGCGAAACCGATAACGGTATCGACGGACTCAAGATCGCCCGCGAGAAAATTCCCAACCTCGTCGTACTCG
ACATCGGCATTCCCAAGCTGGACGGGCTGGAAGTCATCGCCCGGCTGCAATCGCTGGGGTTGCCGCTGCGCGTTCTGGTG
CTGACCGGCCAGCCGCCTTCGCTGTTCGCCCGCCGCTGCCTGAACTCCGGCGCCGCAGGCTTCGTGTGCAAACACGAGAA
CCTGCACGAGGTCATCAATGCCGCCAAGGCGGTGATGGCCGGCTACACCTACTTCCCCAGCACCACGCTCAGCGAGATGC
GCATGGGCGACAACGCCAAGAGCGACAGTACGCTCATCAGCGTGTTGTCCAACCGCGAACTGACCGTCCTGCAACTGCTG
GCGCAAGGCATGTCCAACAAGGACATCGCCGACAGCATGTTCCTCAGCAACAAGACCGTCAGCACCTACAAGACGCGCCT
GCTGCAGAAGCTGAACGCCACGTCGCTGGTGGAACTGATAGACCTCGCCAAACGCAACAATCTCGCCTAG
>Bb_B0224
ATGTACAACAAAGTCCTCATCATTGACGATCACCCTGTACTGAGATTCGCCGTCCGGGTCCTGATGGAAAAGGAAGGATT
CGAAGTCATCGGCGAAACCGATAACGGTATCGACGGGCTCAAGATCGCCCGCGAGAAAATTCCCAACCTCGTCGTACTCG
ACATCGGCATTCCCAAGCTGGACGGGCTGGAAGTCATCGCCCGGCTGCAATCGCTGGGGTTGCCGCTGCGCGTTCTGGTG
CTGACCGGCCAGCCGCCTTCGCTGTTCGCCCGCCGCTGCCTGAACTCCGGCGCCGCAGGCTTCGTGTGCAAACACGAGAA
CCTGCACGAGGTCATCAATGCCGCCAAGGCGGTGATGGCCGGCTACACCTACTTCCCCAGCACCACGCTCAGCGAGATGC
GCATGGGCGACAACGCCAAGAGCGACAGTACGCTCATCAGCGTGTTGTCCAACCGCGAACTGACCGTCCTGCAACTGCTG
GCGCAAGGCATGTCCAACAAGGACATCGCCGACAGCATGTTCCTCAGCAACAAGACCGTCAGCACCTACAAGACGCGCCT
GCTGCAGAAGCTGAACGCCACGTCGCTGGTGGAACTGATAGACCTCGCCAAACGCAACAATCTCGCCTAG
>Bb_B0226
ATGTACAACAAAGTCCTCATCATTGACGATCACCCTGTACTGAGATTCGCCGTCCGGGTCCTGATGGAAAAGGAAGGATT
CGAAGTCATCGGCGAAACCGATAACGGTATCGACGGGCTCAAGATCGCCCGCGAGAAAATTCCCAACCTCGTCGTACTCG
ACATCGGCATTCCCAAGCTGGACGGGCTGGAAGTCATCGCCCGGCTGCAATCGCTGGGGTTGCCGCTGCGCGTTCTGGTG
CTGACCGGCCAGCCGCCTTCGCTGTTCGCCCGCCGCTGCCTGAACTCCGGCGCCGCAGGCTTCGTGTGCAAACACGAGAA
CCTGCACGAGGTCATCAATGCCGCCAAGGCGGTGATGGCCGGCTACACCTACTTCCCCAGCACCACGCTCAGCGAGATGC
GCATGGGCGACAACGCCAAGAGCGACAGTACGCTCATCAGCGTGTTGTCCAACCGCGAACTGACCGTCCTGCAACTGCTG
GCGCAAGGCATGTCCAACAAGGACATCGCCGACAGCATGTTCCTCAGCAACAAGACCGTCAGCACCTACAAGACGCGCCT
GCTGCAGAAGCTGAACGCCACGTCGCTGGTGGAACTGATAGACCTCGCCAAACGCAACAATCTCGCCTAG
>Bb_B0228
ATGTACAACAAAGTCCTCATCATTGACGATCACCCTGTACTGAGATTCGCCGTCCGGGTCCTGATGGAAAAGGAAGGATT
CGAAGTCATCGGCGAAACCGATAACGGTATCGACGGGCTCAAGATCGCCCGCGAGAAAATTCCCAACCTCGTCGTACTCG
ACATCGGCATTCCCAAGCTGGACGGGCTGGAAGTCATCGCCCGGCTGCAATCGCTGGGGTTGCCGCTGCGCGTTCTGGTG
CTGACCGGCCAGCCGCCTTCGCTGTTCGCCCGCCGCTGCCTGAACTCCGGCGCCGCAGGCTTCGTGTGCAAACACGAGAA
CCTGCACGAGGTCATCAATGCCGCCAAGGCGGTGATGGCCGGCTACACCTACTTCCCCAGCACCACGCTCAGCGAGATGC
GCATGGGCGACAACGCCAAGAGCGACAGTACGCTCATCAGCGTGTTGTCCAACCGCGAACTGACCGTCCTGCAACTGCTG
GCGCAAGGCATGTCCAACAAGGACATCGCCGACAGCATGTTCCTCAGCAACAAGACCGTCAGCACCTACAAGACGCGCCT
GCTGCAGAAGCTGAACGCCACGTCGCTGGTGGAACTGATAGACCTCGCCAAACGCAACAATCTCGCCTAG
>Bb_B0084
ATGTACAACAAAGTCCTCATCATTGACGATCACCCTGTACTGAGATTCGCCGTCCGGGTCCTAATGGAAAAGGAAGGATT
CGAAGTCATCGGCGAAACCGATAACGGTATCGACGGGCTCAAGATCGCCCGCGAGAAAATTCCCAACCTCGTCGTACTCG
ACATCGGCATTCCCAAGCTGGACGGGCTGGAAGTCATCGCCCGGCTGCAATCGCTGGGGTTGCCGCTGCGCGTTCTGGTG
CTGACCGGCCAGCCGCCTTCGCTGTTCGCCCGCCGCTGCCTGAACTCCGGCGCCGCAGGCTTCGTGTGCAAACACGAGAA
CCTGCACGAGGTCATCAATGCCGCCAAGGCGGTGATGGCCGGCTACACCTACTTCCCCAGCACCACGCTCAGCGAGATGC
GCATGGGCGACAACGCCAAGAGCGACAGTACGCTCATCAGCGTGTTGTCCAACCGCGAACTGACCGTCCTGCAACTGCTG
GCGCAAGGCATGTCCAACAAGGACATCGCCGACAGCATGTTCCTCAGCAACAAGACCGTCAGCACCTACAAGACGCGCCT
GCTGCAGAAGCTGAACGCCACGTCGCTGGTGGAACTGATAGACCTCGCCAAACGCAACAATCTCGCCTAG
>Bb_B1977
ATGTACAACAAAGTCCTCATCATTGACGATCACCCTGTACTGAGATTCGCCGTCCGGGTCCTGATGGAAAAGGAAGGATT
CGAAGTCATCGGCGAAACCGATAACGGTATCGACGGGCTCAAGATCGCCCGCGAGAAAATTCCCAACCTCGTCGTACTCG
ACATCGGCATTCCCAAGCTGGACGGGCTGGAAGTCATCGCCCGGCTGCAATCGCTGGGGTTGCCGCTACGCGTTCTGGTG
CTGACCGGCCAGCCGCCTTCGCTGTTCGCCCGCCGCTGCCTGAACTCCGGCGCCGCAGGCTTCGTGTGCAAACACGAGAA
CCTGCACGAGGTCATCAATGCCGCCAAGGCGGTGATGGCCGGCTACACCTACTTCCCCAGCACCACGCTCAGCGAGATGC
GCATGGGCGACAACGCCAAGAGCGACAGTACGCTCATCAGCGTGTTGTCCAACCGCGAACTGACCGTCCTGCAACTGCTG
GCGCAAGGCATGTCCAACAAGGACATCGCCGACAGCATGTTCCTCAGCAACAAGACCGTCAGCACCTACAAGACGCGCCT
GCTGCAGAAGCTGAACGCCACGTCGCTGGTGGAACTGATAGACCTCGCCAAACGCAACAATCTCGCCTAG
>Bb_B1978
ATGTACAACAAAGTCCTCATCATTGACGATCACCCTGTACTGAGATTCGCCGTCCGGGTCCTGATGGAAAAGGAAGGATT
CGAAGTCATCGGCGAAACCGATAACGGTATCGACGGGCTCAAGATCGCCCGCGAGAAAATTCCCAACCTCGTCGTACTCG
ACATCGGCATTCCCAAGCTGGACGGGCTGGAAGTCATCGCCCGGCTGCAATCGCTGGGGTTGCCGCTACGCGTTCTGGTG
CTGACCGGCCAGCCGCCTTCGCTGTTCGCCCGCCGCTGCCTGAACTCCGGCGCCGCAGGCTTCGTGTGCAAACACGAGAA
CCTGCACGAGGTCATCAATGCCGCCAAGGCGGTGATGGCCGGCTACACCTACTTCCCCAGCACCACGCTCAGCGAGATGC
GCATGGGCGACAACGCCAAGAGCGACAGTACGCTCATCAGCGTGTTGTCCAACCGCGAACTGACCGTCCTGCAACTGCTG
GCGCAAGGCATGTCCAACAAGGACATCGCCGACAGCATGTTCCTCAGCAACAAGACCGTCAGCACCTACAAGACGCGCCT
GCTGCAGAAGCTGAACGCCACGTCGCTGGTGGAACTGATAGACCTCGCCAAACGCAACAATCTCGCCTAG
>Bb_B0261
ATGTACAACAAAGTCCTCATCATTGACGATCACCCTGTACTGAGATTCGCCGTCCGGGTCCTGATGGAAAAGGAAGGATT
CGAAGTCATCGGCGAAACCGATAACGGTATCGACGGGCTCAAGATCGCCCGCGAGAAAATTCCCAACCTCGTCGTACTCG
ACATCGGCATTCCCAAGCTGGACGGGCTGGAAGTCATCGCCCGGCTGCAATCGCTGGGGTTGCCGCTACGCGTTCTGGTG
CTGACCGGCCAGCCGCCTTCGCTGTTCGCCCGCCGCTGCCTGAACTCCGGCGCCGCAGGCTTCGTGTGCAAACACGAGAA
CCTGCACGAGGTCATCAATGCCGCCAAGGCGGTGATGGCCGGCTACACCTACTTCCCCAGCACCACGCTCAGCGAGATGC
GCATGGGCGACAACGCCAAGAGCGACAGTACGCTCATCAGCGTGTTGTCCAACCGCGAACTGACCGTCCTGCAACTGCTG
GCGCAAGGCATGTCCAACAAGGACATCGCCGACAGCATGTTCCTCAGCAACAAGACCGTCAGCACCTACAAGACGCGCCT
GCTGCAGAAGCTGAACGCCACGTCGCTGGTGGAACTGATAGACCTCGCCAAACGCAACAATCTCGCCTAG
>Bb_B0505
ATGTACAACAAAGTCCTCATCATTGACGATCACCCTGTACTGAGATTCGCCGTCCGGGTCCTGATGGAAAAGGAAGGATT
CGAAGTCATCGGCGAAACCGATAACGGTATCGACGGGCTCAAGATCGCCCGCGAGAAAATTCCCAACCTCGTCGTACTCG
ACATCGGCATTCCCAAGCTGGACGGGCTGGAAGTCATCGCCCGGCTGCAATCGCTGGGGTTGCCGCTACGCGTTCTGGTG
CTGACCGGCCAGCCGCCTTCGCTGTTCGCCCGCCGCTGCCTGAACTCCGGCGCCGCAGGCTTCGTGTGCAAACACGAGAA
CCTGCACGAGGTCATCAATGCCGCCAAGGCGGTGATGGCCGGCTACACCTACTTCCCCAGCACCACGCTCAGCGAGATGC
GCATGGGCGACAACGCCAAGAGCGACAGTACGCTCATCAGCGTGTTGTCCAACCGCGAACTGACCGTCCTGCAACTGCTG
GCGCAAGGCATGTCCAACAAGGACATCGCCGACAGCATGTTCCTCAGCAACAAGACCGTCAGCACCTACAAGACGCGCCT
GCTGCAGAAGCTGAACGCCACGTCGCTGGTGGAACTGATAGACCTCGCCAAACGCAACAATCTCGCCTAG
>Bb_B1965
ATGTACAACAAAGTCCTCATCATTGACGATCACCCTGTACTGAGATTCGCCGTCCGGGTCCTGATGGAAAAGGAAGGATT
CGAAGTCATCGGCGAAACCGATAACGGTATCGACGGGCTCAAGATCGCCCGCGAGAAAATTCCCAACCTCGTCGTACTCG
ACATCGGCATTCCCAAGCTGGACGGGCTGGAAGTCATCGCCCGGCTGCAATCGCTGGGGTTGCCGCTGCGCGTTCTGGTG
CTGACCGGCCAGCCGCCTTCGCTGTTCGCCCGCCGCTGCCTGAACTCCGGCGCCGCAGGCTTCGTGTGCAAACACGAGAA
CCTGCACGAGGTCATCAATGCCGCCAAGGCGGTGATGGCCGGCTACACCTACTTCCCCAGCACCACGCTCAGCGAGATGC
GCATGGGCGACAACGCCAAGAGCGACAGTACGCTCATCAGCGTGTTGTCCAACCGCGAACTGACCGTCCTGCAACTGCTG
GCGCAAGGCATGTCCAACAAGGACATCGCCGACAGCATGTTCCTCAGCAACAAGACCGTCAGCACCTACAAGACGCGCCT
GCTGCAGAAGCTGAACGCCACGTCGCTGGTGGAACTGATAGACCTCGCCAAACGCAACAATCTCGCCTAG
>Bb_B1968
ATGTACAACAAAGTCCTCATCATTGACGATCACCCTGTACTGAGATTCGCCGTCCGGGTCCTGATGGAAAAGGAAGGATT
CGAAGTCATCGGCGAAACCGATAACGGCATTGACGGGCTCAAGATCGCCCGCGAGAAAATTCCCAACCTCGTCGTACTCG
ACATCGGCATTCCCAAGCTGGACGGGCTGGAAGTCATCGCCCGGCTGCAATCGCTGGGGTTGCCGCTGCGCGTGCTGGTG
CTGACCGGCCAGCCGCCTTCGCTGTTCGCCCGCCGCTGCCTGAACTCCGGCGCCGCAGGCTTCGTGTGCAAACACGAGAA
CCTGCACGAGGTCATCAATGCCGCCAAGGCGGTGATGGCCGGCTACACCTACTTCCCCAGCACCACGCTCAGCGAGATGC
GCATGGGCGACAACGCCAAGAGCGACAGTACGCTCATCAGCGTGTTGTCCAACCGCGAACTGACCGTCCTGCAACTGCTG
GCGCAAGGCATGTCCAACAAGGACATCGCGGACAGCATGTTCCTCAGCAACAAGACCGTCAGCACCTACAAGACGCGCCT
GCTGCAGAAGCTGAACGCCACGTCGCTGGTGGAACTGATAGACCTCGCCAAACGCAACAATCTCGCCTAG
>Bb_B1969
ATGTACAACAAAGTCCTCATCATTGACGATCACCCTGTACTGAGATTCGCCGTCCGGGTCCTGATGGAAAAGGAAGGATT
CGAAGTCATCGGCGAAACCGATAACGGCATTGACGGGCTCAAGATCGCCCGCGAGAAAATTCCCAACCTCGTCGTACTCG
ACATCGGCATTCCCAAGCTGGACGGGCTGGAAGTCATCGCCCGGCTGCAATCGCTGGGGTTGCCGCTGCGCGTGCTGGTG
CTGACCGGCCAGCCGCCTTCGCTGTTCGCCCGCCGCTGCCTGAACTCCGGCGCCGCAGGCTTCGTGTGCAAACACGAGAA
CCTGCACGAGGTCATCAATGCCGCCAAGGCGGTGATGGCCGGCTACACCTACTTCCCCAGCACCACGCTCAGCGAGATGC
GCATGGGCGACAACGCCAAGAGCGACAGTACGCTCATCAGCGTGTTGTCCAACCGCGAACTGACCGTCCTGCAACTGCTG
GCGCAAGGCATGTCCAACAAGGACATCGCGGACAGCATGTTCCTCAGCAACAAGACCGTCAGCACCTACAAGACGCGCCT
GCTGCAGAAGCTGAACGCCACGTCGCTGGTGGAACTGATAGACCTCGCCAAACGCAACAATCTCGCCTAG
>Bb_B1973
ATGTACAACAAAGTCCTCATCATTGACGATCACCCTGTACTGAGATTCGCCGTCCGGGTCCTGATGGAAAAGGAAGGATT
CGAAGTCATCGGCGAAACCGATAACGGTATCGACGGGCTCAAGATCGCCCGCGAGAAAATTCCCAACCTCGTCGTACTCG
ACATCGGCATTCCCAAGCTGGACGGGCTGGAAGTCATCGCCCGGCTGCAATCGCTGGGGTTGCCGCTGCGCGTTCTGGTG
CTGACCGGCCAGCCGCCTTCGCTGTTCGCCCGCCGCTGCCTGAACTCCGGCGCCGCAGGCTTCGTGTGCAAACACGAGAA
CCTGCACGAGGTCATCAATGCCGCCAAGGCGGTGATGGCCGGCTACACCTACTTCCCCAGCACCACGCTCAGCGAGATGC
GCATGGGCGACAACGCCAAGAGCGACAGTACGCTCATCAGCGTGTTGTCCAACCGCGAACTGACCGTCCTGCAACTGCTG
GCGCAAGGCATGTCCAACAAGGACATCGCCGACAGCATGTTCCTCAGCAACAAGACCGTCAGCACCTACAAGACGCGCCT
GCTGCAGAAGCTGAACGCCACGTCGCTGGAGGAACTGATAGACCTCGCCAAACGCAACAATCTCGCCTAG
>Bb_B1985
ATGTACAACAAAGTCCTCATCATTGACGATCACCCTGTACTGAGATTCGCCGTCCGGGTCCTGATGGAAAAGGAAGGATT
CGAAGTCATCGGCGAAACCGATAACGGTATCGACGGGCTCAAGATCGCCCGCGAGAAAATTCCCAACCTCGTCGTACTCG
ACATCGGCATTCCCAAGCTGGACGGGCTGGAAGTCATCGCCCGGCTGCAATCGCTGGGGTTGCCGCTGCGCGTTCTGGTG
CTGACCGGCCAGCCGCCTTCGCTGTTCGCCCGCCGCTGCCTGAACTCCGGCGCCGCAGGCTTCGTGTGCAAACACGAGAA
CCTGCACGAGGTCATCAATGCCGCCAAGGCGGTGATGGCCGGCTACACCTACTTCCCCAGCACCACGCTCAGCGAGATGC
GCATGGGCGACAACGCCAAGAGCGACAGTACGCTCATCAGCGTGTTGTCCAACCGCGAACTGACCGTCCTGCAACTGCTG
GCGCAAGGCATGTCCAACAAGGACATCGCCGACAGCATGTTCCTCAGCAACAAGACCGTCAGCACCTACAAGACGCGCCT
GCTGCAGAAGCTGAACGCCACGTCGCTGGTGGAACTGATAGACCTCGCCAAACGCAACAATCTCGCCTAG
>Bb_B1986
ATGTACAACAAAGTCCTCATCATTGACGATCACCCTGTACTGAGATTCGCCGTCCGGGTCCTGATGGAAAAGGAAGGATT
CGAAGTCATCGGCGAAACCGATAACGGTATCGACGGGCTCAAGATCGCCCGCGAGAAAATTCCCAACCTCGTCGTACTCG
ACATCGGCATTCCCAAGCTGGACGGGCTGGAAGTCATCGCCCGGCTGCAATCGCTGGGGTTGCCGCTGCGCGTTCTGGTG
CTGACCGGCCAGCCGCCTTCGCTGTTCGCCCGCCGCTGCCTGAACTCCGGCGCCGCAGGCTTCGTGTGCAAACACGAGAA
CCTGCACGAGGTCATCAATGCCGCCAAGGCGGTGATGGCCGGCTACACCTACTTCCCCAGCACCACGCTCAGCGAGATGC
GCATGGGCGACAACGCCAAGAGCGACAGTACGCTCATCAGCGTGTTGTCCAACCGCGAACTGACCGTCCTGCAACTGCTG
GCGCAAGGCATGTCCAACAAGGACATCGCCGACAGCATGTTCCTCAGCAACAAGACCGTCAGCACCTACAAGACGCGCCT
GCTGCAGAAGCTGAACGCCACGTCGCTGGTGGAACTGATAGACCTCGCCAAACGCAACAATCTCGCCTAG
>Bb_B1987
ATGTACAACAAAGTCCTCATCATTGACGATCACCCTGTACTGAGATTCGCCGTCCGGGTCCTGATGGAAAAGGAAGGATT
CGAAGTCATCGGCGAAACCGATAACGGTATCGACGGGCTCAAGATCGCCCGCGAGAAAATTCCCAACCTCGTCGTACTCG
ACATCGGCATTCCCAAGCTGGACGGGCTGGAAGTCATCGCCCGGCTGCAATCGCTGGGGTTGCCGCTGCGCGTTCTGGTG
CTGACCGGCCAGCCGCCTTCGCTGTTCGCCCGCCGCTGCCTGAACTCCGGCGCCGCAGGCTTCGTGTGCAAACACGAGAA
CCTGCACGAGGTCATCAATGCCGCCAAGGCGGTGATGGCCGGCTACACCTACTTCCCCAGCACCACGCTCAGCGAGATGC
GCATGGGCGACAACGCCAAGAGCGACAGTACGCTCATCAGCGTGTTGTCCAACCGCGAACTGACCGTCCTGCAACTGCTG
GCGCAAGGCATGTCCAACAAGGACATCGCCGACAGCATGTTCCTCAGCAACAAGACCGTCAGCACCTACAAGACGCGCCT
GCTGCAGAAGCTGAACGCCACGTCGCTGGTGGAACTGATAGACCTCGCCAAACGCAACAATCTCGCCTAG
>Bb_B2104
ATGTACAACAAAGTCCTCATCATTGACGATCACCCTGTACTGAGATTCGCCGTCCGGGTCCTGATGGAAAAGGAAGGATT
CGAAGTCATCGGCGAAACCGATAACGGTATCGACGGGCTCAAGATCGCCCGCGAGAAAATTCCCAACCTCGTCGTACTCG
ACATCGGCATTCCCAAGCTGGACGGGCTGGAAGTCATCGCCCGGCTGCAATCGCTGGGGTTGCCGCTGCGCGTTCTGGTG
CTGACCGGCCAGCCGCCTTCGCTGTTCGCCCGCCGCTGCCTGAACTCCGGCGCCGCAGGCTTCGTGTGCAAACACGAGAA
CCTGCACGAGGTCATCAATGCCGCCAAGGCGGTGATGGCCGGCTACACCTACTTCCCCAGCACCACGCTCAGCGAGATGC
GCATGGGCGACAACGCCAAGAGCGACAGTACGCTCATCAGCGTGTTGTCCAACCGCGAACTGACCGTCCTGCAACTGCTG
GCGCAAGGCATGTCCAACAAGGACATCGCCGACAGCATGTTCCTCAGCAACAAGACCGTCAGCACCTACAAGACGCGCCT
GCTGCAGAAGCTGAACGCCACGTCGCTGGTGGAACTGATAGACCTCGCCAAACGCAACAATCTCGCCTAG
>Bb_B2105
ATGTACAACAAAGTCCTCATCATTGACGATCACCCTGTACTGAGATTCGCCGTCCGGGTCCTGATGGAAAAGGAAGGATT
CGAAGTCATCGGCGAAACCGATAACGGTATCGACGGGCTCAAGATCGCCCGCGAGAAAATTCCCAACCTCGTCGTACTCG
ACATCGGCATTCCCAAGCTGGACGGGCTGGAAGTCATCGCCCGGCTGCAATCGCTGGGGTTGCCGCTGCGCGTTCTGGTG
CTGACCGGCCAGCCGCCTTCGCTGTTCGCCCGCCGCTGCCTGAACTCCGGCGCCGCAGGCTTCGTGTGCAAACACGAGAA
CCTGCACGAGGTCATCAATGCCGCCAAGGCGGTGATGGCCGGCTACACCTACTTCCCCAGCACCACGCTCAGCGAGATGC
GCATGGGCGACAACGCCAAGAGCGACAGTACGCTCATCAGCGTGTTGTCCAACCGCGAACTGACCGTCCTGCAACTGCTG
GCGCAAGGCATGTCCAACAAGGACATCGCCGACAGCATGTTCCTCAGCAACAAGACCGTCAGCACCTACAAGACGCGCCT
GCTGCAGAAGCTGAACGCCACGTCGCTGGTGGAACTGATAGACCTCGCCAAACGCAACAATCTCGCCTAG
>Bb_B2108
ATGTACAACAAAGTCCTCATCATTGACGATCACCCTGTACTGAGATTCGCCGTCCGGGTCCTGATGGAAAAGGAAGGATT
CGAAGTCATCGGCGAAACCGATAACGGTATCGACGGGCTCAAGATCGCCCGCGAGAAAATTCCCAACCTCGTCGTACTCG
ACATCGGCATTCCCAAGCTGGACGGGCTGGAAGTCATCGCCCGGCTGCAATCGCTGGGGTTGCCGCTGCGCGTTCTGGTG
CTGACCGGCCAGCCGCCTTCGCTGTTCGCCCGCCGCTGCCTGAACTCCGGCGCCGCAGGCTTCGTGTGCAAACACGAGAA
CCTGCACGAGGTCATCAATGCCGCCAAGGCGGTGATGGCCGGCTACACCTACTTCCCCAGCACCACGCTCAGCGAGATGC
GCATGGGCGACAACGCCAAGAGCGACAGTACGCTCATCAGCGTGTTGTCCAACCGCGAACTGACCGTCCTGCAACTGCTG
GCGCAAGGCATGTCCAACAAGGACATCGCCGACAGCATGTTCCTCAGCAACAAGACCGTCAGCACCTACAAGACGCGCCT
GCTGCAGAAGCTGAACGCCACGTCGCTGGTGGAACTGATAGACCTCGCCAAACGCAACAATCTCGCCTAG
>Bb_B2112
ATGTACAACAAAGTCCTCATCATTGACGATCACCCTGTACTGAGATTCGCCGTCCGGGTCCTGATGGAAAAGGAAGGATT
CGAAGTCATCGGCGAAACCGATAACGGTATCGACGGGCTCAAGATCGCCCGCGAGAAAATTCCCAACCTCGTCGTACTCG
ACATCGGCATTCCCAAGCTGGACGGGCTGGAAGTCATCGCCCGGCTGCAATCGCTGGGGTTGCCGCTGCGCGTTCTGGTG
CTGACCGGCCAGCCGCCTTCGCTGTTCGCCCGCCGCTGCCTGAACTCCGGCGCCGCAGGCTTCGTGTGCAAACACGAGAA
CCTGCACGAGGTCATCAATGCCGCCAAGGCGGTGATGGCCGGCTACACCTACTTCCCCAGCACCACGCTCAGCGAGATGC
GCATGGGCGACAACGCCAAGAGCGACAGTACGCTCATCAGCGTGTTGTCCAACCGCGAACTGACCGTCCTGCAACTGCTG
GCGCAAGGCATGTCCAACAAGGACATCGCCGACAGCATGTTCCTCAGCAACAAGACCGTCAGCACCTACAAGACGCGCCT
GCTGCAGAAGCTGAACGCCACGTCGCTGGTGGAACTGATAGACCTCGCCAAACGCAACAATCTCGCCTAG
>Bb_B2114
ATGTACAACAAAGTCCTCATCATTGACGATCACCCTGTACTGAGATTCGCCGTCCGGGTCCTGATGGAAAAGGAAGGATT
CGAAGTCATCGGCGAAACCGATAACGGCATTGACGGGCTCAAGATCGCCCGCGAGAAAATTCCCAACCTCGTCGTACTCG
ACATCGGCATTCCCAAGCTGGACGGGCTGGAAGTCATCGCCCGGCTGCAATCGCTGGGGTTGCCGCTGCGCGTGCTGGTG
CTGACCGGCCAGCCGCCTTCGCTGTTCGCCCGCCGCTGCCTGAACTCCGGCGCCGCAGGCTTCGTGTGCAAACACGAGAA
CCTGCACGAGGTCATCAATGCCGCCAAGGCGGTGATGGCCGGCTACACCTACTTCCCCAGCACCACGCTCAGCGAGATGC
GCATGGGCGACAACGCCAAGAGCGACAGTACGCTCATCAGCGTGTTGTCCAACCGCGAACTGACCGTCCTGCAACTGCTG
GCGCAAGGCATGTCCAACAAGGACATCGCGGACAGCATGTTCCTCAGCAACAAGACCGTCAGCACCTACAAGACGCGCCT
GCTGCAGAAGCTGAACGCCACGTCGCTGGTGGAACTGATAGACCTCGCCAAACGCAACAATCTCGCCTAG
>Bb_B2115
ATGTACAACAAAGTCCTCATCATTGACGATCACCCTGTACTGAGATTCGCCGTCCGGGTCCTGATGGAAAAGGAAGGATT
CGAAGTCATCGGCGAAACCGATAACGGTATCGACGGGCTCAAGATCGCCCGCGAGAAAATTCCCAACCTCGTCGTACTCG
ACATCGGCATTCCCAAGCTGGACGGGCTGGAAGTCATCGCCCGGCTGCAATCGCTGGGGTTGCCGCTGCGCGTTCTGGTG
CTGACCGGCCAGCCGCCTTCGCTGTTCGCCCGCCGCTGCCTGAACTCCGGCGCCGCAGGCTTCGTGTGCAAACACGAGAA
CCTGCACGAGGTCATCAATGCCGCCAAGGCGGTGATGGCCGGCTACACCTACTTCCCCAGCACCACGCTCAGCGAGATGC
GCATGGGCGGCAACGCCAAGAGCGACAGTACGCTCATCAGCGTGTTGTCCAACCGCGAACTGACCGTCCTGCAACTGCTG
GCGCAAGGCATGTCCAACAAGGACATCGCCGACAGCATGTTCCTCAGCAACAAGACCGTCAGCACCTACAAGACGCGCCT
GCTGCAGAAGCTGAACGCCACGTCGCTGGTGGAACTGATAGACCTCGCCAAACGCAACAATCTCGCCTAG
>Bb_B2116
ATGTACAACAAAGTCCTCATCATTGACGATCACCCTGTACTGAGATTCGCCGTCCGGGTCCTGATGGAAAAGGAAGGATT
CGAAGTCATCGGCGAAACCGATAACGGTATCGACGGGCTCAAGATCGCCCGCGAGAAAATTCCCAACCTCGTCGTACTCG
ACATCGGCATTCCCAAGCTGGACGGGCTGGAAGTCATCGCCCGGCTGCAATCGCTGGGGTTGCCGCTGCGCGTTCTGGTG
CTGACCGGCCAGCCGCCTTCGCTGTTCGCCCGCCGCTGCCTGAACTCCGGCGCCGCAGGCTTCGTGTGCAAACACGAGAA
CCTGCACGAGGTCATCAATGCCGCCAAGGCGGTGATGGCCGGCTACACCTACTTCCCCAGCACCACGCTCAGCGAGATGC
GCATGGGCGACAACGCCAAGAGCGACAGTACGCTCATCAGCGTGTTGTCCAACCGCGAACTGACCGTCCTGCAACTGCTG
GCGCAAGGCATGTCCAACAAGGACATCGCCGACAGCATGTTCCTCAGCAACAAGACCGTCAGCACCTACAAGACGCGCCT
GCTGCAGAAGCTGAACGCCACGTCGCTGGTGGAACTGATAGACCTCGCCAAACGCAACAATCTCGCCTAG
>Bb_B2490
ATGTACAACAAAGTCCTCATCATTGACGATCACCCTGTACTGAGATTCGCCGTCCGGGTCCTGATGGAAAAGGAAGGATT
CGAAGTCATCGGCGAAACCGATAACGGCATTGACGGGCTCAAGATCGCCCGCGAGAAAATTCCCAACCTCGTCGTACTCG
ACATCGGCATTCCCAAGCTGGACGGGCTGGAAGTCATCGCCCGGCTGCAATCGCTGGGGTTGCCGCTGCGCGTGCTGGTG
CTGACCGGCCAGCCGCCTTCGCTGTTCGCCCGCCGCTGCCTGAACTCCGGCGCCGCAGGCTTCGTGTGCAAACACGAGAA
CCTGCACGAGGTCATCAATGCCGCCAAGGCGGTGATGGCCGGCTACACCTACTTCCCCAGCACTACGCTCAGCGAGATGC
GCATGGGCGACAACGCCAAGAGCGACAGTACGCTCATCAGCGTGTTGTCCAACCGCGAACTGACCGTCCTGCAACTGCTG
GCGCAAGGCATGTCCAACAAGGACATCGCGGACAGCATGTTCCTCAGCAACAAGACCGTCAGCACCTACAAGACGCGCCT
GCTGCAGAAGCTGAACGCCACGTCGCTGGTGGAACTGATAGACCTCGCCAAACGCAACAATCTCGCCTAG
>Bb_B2491
ATGTACAACAAAGTCCTCATCATTGACGATCACCCTGTACTGAGATTCGCCGTCCGGGTCCTGATGGAAAAGGAAGGATT
CGAAGTCATCGGCGAAACCGATAACGGCATTGACGGGCTCAAGATCGCCCGCGAGAAAATTCCCAATCTCGTCGTACTCG
ACATCGGCATTCCCAAGCTGGACGGGCTGGAAGTCATCGCCCGGCTGCAATCGCTGGGGTTGCCGCTGCGCGTGCTGGTG
CTGACCGGCCAGCCGCCTTCGCTGTTCGCCCGCCGCTGCCTGAACTCCGGCGCCGCAGGCTTCGTGTGCAAACACGAGAA
CCTGCACGAGGTCATCAATGCCGCCAAGGCGGTGATGGCCGGCTACACCTACTTCCCCAGCACCACGCTCAGCGAGATGC
GCATGGGCGACAACGCCAAGAGCGACAGTACGCTCATCAGCGTGTTGTCCAACCGCGAACTGACCGTCCTGCAACTGCTG
GCGCAAGGCATGTCCAACAAGGACATCGCGGACAGCATGTTCCTCAGCAACAAGACCGTCAGCACCTACAAGACGCGCCT
GCTGCAGAAGCTGAACGCCACGTCGCTGGTGGAACTGATAGACCTCGCCAAACGCAACAATCTCGCCTAG
>Bb_B2492
ATGTACAACAAAGTCCTCATCATTGACGATCACCCTGTACTGAGATTCGCCGTCCGGGTCCTGATGGAAAAGGAAGGATT
CGAAGTCATCGGCGAAACCGATAACGGTATTGACGGGCTCAAGATCGCCCGCGAGAAAATTCCCAACCTCGTCGTACTCG
ACATCGGCATTCCCAAGCTGGACGGGCTGGAAGTCATCGCCCGGCTGCAATCGCTGGGGTTGCCGCTGCGCGTGCTGGTG
CTGACCGGCCAGCCGCCTTCGCTGTTCGCCCGCCGCTGCCTGAACTCCGGCGCCGCAGGCTTCGTGTGCAAACACGAGAA
CCTGCACGAGGTCATCAATGCCGCCAAGGCGGTGATGGCCGGCTACACCTACTTCCCCAGCACCACGCTCAGCGAGATGC
GCATGGGCGACAACGCCAAGAGCGACAGTACGCTCATCAGCGTGTTGTCCAACCGCGAACTGACCGTCCTGCAACTGCTG
GCGCAAGGCATGTCCAACAAGGACATCGCTGACAGCATGTTCCTCAGCAACAAGACTGTCAGCACCTACAAGACGCGCCT
GCTGCAGAAGCTGAACGCCACGTCGCTGGTGGAACTGATAGACCTCGCCAAACGCAACAATCTCGCCTAG
>Bb_B2493
ATGTACAACAAAGTCCTCATCATTGACGATCACCCTGTACTGAGATTCGCCGTCCGGGTCCTGATGGAAAAGGAAGGATT
CGAAGTCATCGGCGAAACCGATAACGGTATCGACGGGCTCAAGATCGCCCGCGAGAAAATTCCCAACCTCGTCGTACTCG
ACATCGGCATTCCCAAGCTGGACGGGCTGGAAGTCATCGCCCGGCTGCAATCGCTGGGGTTGCCGCTGCGCGTTCTGGTG
CTGACCGGCCAGCCGCCTTCGCTGTTCGCCCGCCGCTGCCTGAACTCCGGCGCCGCAGGCTTCGTGTGCAAACACGAGAA
CCTGCACGAGGTCATCAATGCCGCCAAGGCGGTGATGGCCGGCTACACCTACTTCCCCAGCACCACGCTCAGCGAGATGC
GCATGGGCGACAACGCCAAGAGCGACAGTACGCTCATCAGCGTGTTGTCCAACCGCGAACTGACCGTCCTGCAACTGCTG
GCGCAAGGCATGTCCAACAAGGACATCGCCGACAGCATGTTCCTCAGCAACAAGACCGTCAGCACCTACAAGACGCGCCT
GCTGCAGAAGCTGAACGCCACGTCGCTGGTGGAACTGATAGACCTCGCCAAACGCAACAATCTCGCCTAG
>Bb_B2494
ATGTACAACAAAGTCCTCATCATTGACGATCACCCTGTACTGAGATTCGCCGTCCGGGTCCTGATGGAAAAGGAAGGATT
CGAAGTCATCGGCGAAACCGATAACGGCATTGACGGGCTCAAGATCGCCCGCGAGAAAATTCCCAATCTCGTCGTACTCG
ACATCGGCATTCCCAAGCTGGACGGGCTGGAAGTCATCGCCCGGCTGCAATCGCTGGGGTTGCCGCTGCGCGTGCTGGTG
CTGACCGGCCAGCCGCCTTCGCTGTTCGCCCGCCGCTGCCTGAACTCCGGCGCCGCAGGCTTCGTGTGCAAACACGAGAA
CCTGCACGAGGTCATCAATGCCGCCAAGGCGGTGATGGCCGGCTACACCTACTTCCCCAGCACCACGCTCAGCGAGATGC
GCATGGGCGACAACGCCAAGAGCGACAGTACGCTCATCAGCGTGTTGTCCAACCGCGAACTGACCGTCCTGCAACTGCTG
GCGCAAGGCATGTCCAACAAGGACATCGCGGACAGCATGTTCCTCAGCAACAAGACCGTCAGCACCTACAAGACGCGCCT
GCTGCAGAAGCTGAACGCCACGTCGCTGGTGGAACTGATAGACCTCGCCAAACGCAACAATCTCGCCTAG
>Bb_B2495
ATGTACAACAAAGTCCTCATCATTGACGATCACCCTGTACTGAGATTCGCCGTCCGGGTCCTGATGGAAAAGGAAGGATT
CGAAGTCATCGGCGAAACCGATAACGGTATTGACGGGCTCAAGATCGCCCGCGAGAAAATTCCCAACCTCGTCGTACTCG
ACATCGGCATTCCCAAGCTGGACGGGCTGGAAGTCATCGCCCGGCTGCAATCGCTGGGGTTGCCGCTGCGCGTGCTGGTG
CTGACCGGCCAGCCGCCTTCGCTGTTCGCCCGCCGCTGCCTGAACTCCGGCGCCGCAGGCTTCGTGTGCAAACACGAGAA
CCTGCACGAGGTCATCAATGCCGCCAAGGCGGTGATGGCCGGCTACACCTACTTCCCCAGCACCACGCTCAGCGAGATGC
GCATGGGCGACAACGCCAAGAGCGACAGTACGCTCATCAGCGTGTTGTCCAACCGCGAACTGACCGTCCTGCAACTGCTG
GCGCAAGGCATGTCCAACAAGGACATCGCGGACAGCATGTTCCTCAGCAACAAGACCGTCAGCACCTACAAGACGCGCCT
GCTGCAGAAGCTGAACGCCACGTCGCTGGTGGAACTGATAGACCTCGCCAAACGCAACAATCTCGCCTAG
>Bb_B2498
ATGTACAACAAAGTCCTCATCATTGACGATCACCCTGTACTGAGATTCGCCGTCCGGGTCCTGATGGAAAAGGAAGGATT
CGAAGTCATCGGCGAAACCGATAACGGTATCGACGGGCTCAAGATCGCCCGCGAGAAAATTCCCAACCTCGTCGTACTCG
ACATCGGCATTCCCAAGCTGGACGGGCTGGAAGTCATCGCCCGGCTGCAATCGCTGGGGTTGCCGCTGCGCGTTCTGGTG
CTGACCGGCCAGCCGCCTTCGCTGTTCGCCCGCCGCTGCCTGAACTCCGGCGCCGCAGGCTTCGTGTGCAAACACGAGAA
CCTGCACGAGGTCATCAATGCCGCCAAGGCGGTGATGGCCGGCTACACCTACTTCCCCAGCACCACGCTCAGCGAGATGC
GCATGGGCGACAACGCCAAGAGCGACAGTACGCTCATCAGCGTGTTGTCCAACCGCGAACTGACCGTCCTGCAACTGCTG
GCGCAAGGCATGTCCAACAAGGACATCGCCGACAGCATGTTCCTCAGCAACAAGACCGTCAGCACCTACAAGACGCGCCT
GCTGCAGAAGCTGAACGCCACGTCGCTGGTGGAACTGATAGACCTCGCCAAACGCAACAATCTCGCCTAG
>Bb_B2501
ATGTACAACAAAGTCCTCATCATTGACGATCACCCTGTACTGAGATTCGCCGTCCGGGTCCTGATGGAAAAGGAAGGATT
CGAAGTCATCGGCGAAACCGATAACGGTATCGACGGGCTCAAGATCGCCCGCGAGAAAATTCCCAACCTCGTCGTACTCG
ACATCGGCATTCCCAAGCTGGACGGGCTGGAAGTCATCGCCCGGCTGCAATCGCTGGGGTTGCCGCTGCGCGTTCTGGTG
CTGACCGGCCAGCCGCCTTCGCTGTTCGCCCGCCGCTGCCTGAACTCCGGCGCCGCAGGCTTCGTGTGCAAACACGAGAA
CCTGCACGAGGTCATCAATGCCGCCAAGGCGGTGATGGCCGGCTACACCTACTTCCCCAGCACCACGCTCAGCGAGATGC
GCATGGGCGACAACGCCAAGAGCGACAGTACGCTCATCAGCGTGTTGTCCAACCGCGAACTGACCGTCCTGCAACTGCTG
GCGCAAGGCATGTCCAACAAGGACATCGCCGACAGCATGTTCCTCAGCAACAAGACCGTCAGCACCTACAAGACGCGCCT
GCTGCAGAAGCTGAACGCCACGTCGCTGGTGGAACTGATAGACCTCGCCAAACGCAACAATCTCGCCTAG
>Bb_B2506
ATGTACAACAAAGTCCTCATCATTGACGATCACCCTGTACTGAGATTCGCCGTCCGGGTCCTGATGGAAAAGGAAGGATT
CGAAGTCATCGGCGAAACCGATAACGGCATTGACGGGCTCAAGATCGCCCGCGAGAAAATTCCCAACCTCGTCGTACTCG
ACATCGGCATTCCCAAGCTGGACGGGCTGGAAGTCATCGCCCGGCTGCAATCGCTGGGGTTGCCGCTGCGCGTGCTGGTG
CTGACCGGCCAGCCGCCTTCGCTGTTCGCCCGCCGCTGCCTGAACTCCGGCGCCGCAGGCTTCGTGTGCAAACACGAGAA
CCTGCACGAGGTCATCAATGCCGCCAAGGCGGTGATGGCCGGCTACACCTACTTCCCCAGCACTACGCTCAGCGAGATGC
GCATGGGCGACAACGCCAAGAGCGACAGTACGCTCATCAGCGTGTTGTCCAACCGCGAACTGACCGTCCTGCAACTGCTG
GCGCAAGGCATGTCCAACAAGGACATCGCGGACAGCATGTTCCTCAGCAACAAGACCGTCAGCACCTACAAGACGCGCCT
GCTGCAGAAGCTGAACGCCACGTCGCTGGTGGAAcTGATAGACCTCGCCAAACGCAACAATCTCGCCTAG
>Bb_B2508
ATGTACAACAAAGTCCTCATCATTGACGATCACCCTGTACTGAGATTCGCCGTCCGGGTCCTGATGGAAAAGGAAGGATT
CGAAGTCATCGGCGAAACCGATAACGGTATCGACGGGCTCAAGATCGCCCGCGAGAAAATTCCCAACCTCGTCGTACTCG
ACATCGGCATTCCCAAGCTGGACGGGCTGGAAGTCATCGCCCGGCTGCAATCGCTGGGGTTGCCGCTGCGCGTTCTGGTG
CTGACCGGCCAGCCGCCTTCGCTGTTCGCCCGCCGCTGCCTGAACTCCGGCGCCGCAGGCTTCGTGTGCAAACACGAGAA
CCTGCACGAGGTCATCAATGCCGCCAAGGCGGTGATGGCCGGCTACACCTACTTCCCCAGCACCACGCTCAGCGAGATGC
GCATGGGCGACAACGCCAAGAGCGACAGTACGCTCATCAGCGTGTTGTCCAACCGCGAACTGACCGTCCTGCAACTGCTG
GCGCAAGGCATGTCCAACAAGGACATCGCCGACAGCATGTTCCTCAGCAACAAGACCGTCAGCACCTACAAGACGCGCCT
GCTGCAGAAGCTGAACGCCACGTCGCTGGTGGAACTGATAGACCTCGCCAAACGCAACAATCTCGCCTAG
>Bb_B2509
ATGTACAACAAAGTCCTCATCATTGACGATCACCCTGTACTGAGATTCGCCGTCCGGGTCCTGATGGAAAAGGAAGGATT
CGAAGTCATCGGCGAAACCGATAACGGTATCGACGGGCTCAAGATCGCCCGCGAGAAAATTCCCAACCTCGTCGTACTCG
ACATCGGCATTCCCAAGCTGGACGGGCTGGAAGTCATCGCCCGGCTGCAATCGCTGGGGTTGCCGCTACGCGTTCTGGTG
CTGACCGGCCAGCCGCCTTCGCTGTTCGCCCGCCGCTGCCTGAACTCCGGCGCCGCAGGCTTCGTGTGCAAACACGAGAA
CCTGCACGAGGTCATCAATGCCGCCAAGGCGGTGATGGCCGGCTACACCTACTTCCCCAGCACCACGCTCAGCGAGATGC
GCATGGGCGACAACGCCAAGAGCGACAGTACGCTCATCAGCGTGTTGTCCAACCGCGAACTGACCGTCCTGCAACTGCTG
GCGCAAGGCATGTCCAACAAGGACATCGCCGACAGCATGTTCCTCAGCAACAAGACCGTCAGCACCTACAAGACGCGCCT
GCTGCAGAAGCTGAACGCCACGTCGCTGGTGGAACTGATAGACCTCGCCAAACGCAACAATCTCGCCTAG
>Bb_B2511
ATGTACAACAAAGTCCTCATCATTGACGATCACCCTGTACTGAGATTCGCCGTCCGGGTCCTGATGGAAAAGGAAGGATT
CGAAGTCATCGGCGAAACCGATAACGGTATCGACGGGCTCAAGATCGCCCGCGAGAAAATTCCCAACCTCGTCGTACTCG
ACATCGGCATTCCCAAGCTGGACGGGCTGGAAGTCATCGCCCGGCTGCAATCGCTGGGGTTGCCGCTGCGCGTTCTGGTG
CTGACCGGCCAGCCGCCTTCGCTGTTCGCCCGCCGCTGCCTGAACTCCGGCGCCGCAGGCTTCGTGTGCAAACACGAGAA
CCTGCACGAGGTCATCAATGCCGCCAAGGCGGTGATGGCCGGCTACACCTACTTCCCCAGCACCACGCTCAGCGAGATGC
GCATGGGCGACAACGCCAAGAGCGACAGTACGCTCATCAGCGTGTTGTCCAACCGCGAACTGACCGTCCTGCAACTGCTG
GCGCAAGGCATGTCCAACAAGGACATCGCCGACAGCATGTTCCTCAGCAACAAGACCGTCAGCACCTACAAGACGCGCCT
GCTGCAGAAGCTGAACGCCACGTCGCTGGTGGAACTGATAGACCTCGCCAAACGCAACAATCTCGCCTAG
>Bb_B2586
ATGTACAACAAAGTCCTCATCATTGACGATCACCCTGTACTGAGATTCGCCGTCCGGGTCCTGATGGAAAAGGAAGGATT
CGAAGTCATCGGCGAAACCGATAACGGCATTGACGGGCTCAAGATCGCCCGCGAGAAAATTCCCAACCTCGTCGTACTCG
ACATCGGCATTCCCAAGCTGGACGGGCTGGGAGTCATCGCCCGGCTGCAATCGCTGGGGTTGCCGCTGCGCGTGCTGGTG
CTGACCGGCCAGCCGCCTTCGCTGTTCGCCCGCCGCTGCCTGAACTCCGGCGCCGCAGGCTTCGTGTGCAAACACGAGAA
CCTGCACGAGGTCATCAATGCCGCCAAGGCGGTGATGGCCGGCTACACCTACTTCCCCAGCACCACGCTCAGCGAGATGC
GCATGGGCGACAACGCCAAGAGCGACAGTACGCTCATCAGCGTGTTGTCCAACCGCGAACTGACCGTCCTGCAACTGCTG
GCGCAAGGCATGTCCAACAAGGACATCGCGGACAGCATGTTCCTCAGCAACAAGACCGTCAGCACCTACAAGACGCGCCT
GCTGCAGAAGCTGAACGCCACGTCGCTGGTGGAACTGATAGACCTCGCCAAACGCAACAATCTCGCCTAG
>Bb_B2588
ATGTACAACAAAGTCCTCATCATTGACGATCACCCTGTACTGAGATTCGCCGTCCGGGTCCTGATGGAAAAGGAAGGATT
CGAAGTCATCGGCGAAACCGATAACGGCATTGACGGGCTCAAGATCGCCCGCGAGAAAATTCCCAACCTCGTCGTACTCG
ACATCGGCATTCCCAAGCTGGACGGGCTGGAAGTCATCGCCCGGCTGCAATCGCTGGGGTTGCCGCTGCGCGTGCTGGTG
CTGACCGGCCAGCCGCCTTCGCTGTTCGCCCGCCGCTGCCTGAACTCCGGCGCCGCAGGCTTCGTGTGCAAACACGAGAA
CCTGCACGAGGTCATCAATGCCGCCAAGGCGGTGATGGCCGGCTACACCTACTTCCCCAGCACCACGCTCAGCGAGATGC
GCATGGGCGACAACGCCAAGAGCGACAGTACGCTCATCAGCGTGTTGTCCAACCGCGAACTGACCGTCCTGCAACTGCTG
GCGCAAGGCATGTCCAACAAGGACATCGCGGACAGCATGTTCCTCAGCAACAAGACCGTCAGCACCTACAAGACGCGCCT
GCTGCAGAAGCTGAACGCCACGTCGCTGGTGGAACTGATAGACCTCGCCAAACGCAACAATCTCGCCTAG
>Bb_RB50
ATGTACAACAAAGTCCTCATCATTGACGATCACCCTGTACTGAGATTCGCCGTCCGGGTCCTGATGGAAAAGGAAGGATT
CGAAGTCATCGGCGAAACCGATAACGGTATCGACGGGCTCAAGATCGCCCGCGAGAAAATTCCCAACCTCGTCGTACTCG
ACATCGGCATTCCCAAGCTGGACGGGCTGGAAGTCATCGCCCGGCTGCAATCGCTGGGGTTGCCGCTACGCGTTCTGGTG
CTGACCGGCCAGCCGCCTTCGCTGTTCGCCCGCCGCTGCCTGAACTCCGGCGCCGCAGGCTTCGTGTGCAAACACGAGAA
CCTGCACGAGGTCATCAATGCCGCCAAGGCGGTGATGGCCGGCTACACCTACTTCCCCAGCACCACGCTCAGCGAGATGC
GCATGGGCGACAACGCCAAGAGCGACAGTACGCTCATCAGCGTGTTGTCCAACCGCGAACTGACCGTCCTGCAACTGCTG
GCGCAAGGCATGTCCAACAAGGACATCGCCGACAGCATGTTCCTCAGCAACAAGACCGTCAGCACCTACAAGACGCGCCT
GCTGCAGAAGCTGAACGCCACGTCGCTGGTGGAACTGATAGACCTCGCCAAACGCAACAATCTCGCCTAG
>Bp_18323
ATGTACAACAAAGTCCTCATCATTGACGATCACCCTGTACTGAGATTCGCCGTCCGGGTCCTGATGGAAAAGGAAGGATT
CGAAGTCATCGGCGAAACCGATAACGGCATTGACGGGCTCAAGATCGCCCGCGAGAAAATTCCCAACCTCGTCGTACTCG
ACATCGGCATTCCCAAGCTGGACGGGCTGGAAGTCATCGCCCGGCTGCAATCGCTGGGGTTGCCGCTGCGCGTGCTGGTG
CTGACCGGCCAGCCGCCTTCGCTGTTCGCCCGCCGCTGCCTGAACTCCGGCGCCGCAGGCTTCGTGTGCAAACACGAGAA
CCTGCACGAGGTCATCAATGCCGCCAAGGCGGTGATGGCCGGCTACACCTACTTCCCCAGCACCACGCTCAGCGAGATGC
GCATGGGCGACAACGCCAAGAGCGACAGTACGCTCATCAGCGTGTTGTCCAACCGCGAACTGACCGTCCTGCAACTGCTG
GCGCAAGGCATGTCCAACAAGGACATCGCTGACAGCATGTTCCTCAGCAACAAGACCGTCAGCACCTACAAGACGCGCCT
GCTGCAGAAGCTGAACGCCACGTCGCTGGTGGAACTGATAGACCTCGCCAAACGCAACAATCTCGCCTAG
>Bp_TohI
ATGTACAACAAAGTCCTCATCATTGACGATCACCCTGTACTGAGATTCGCCGTCCGGGTCCTGATGGAAAAGGAAGGATT
CGAAGTCATCGGCGAAACCGATAACGGCATTGACGGGCTCAAGATCGCCCGCGAGAAAATTCCCAACCTCGTCGTACTCG
ACATCGGCATTCCCAAGCTGGACGGGCTGGAAGTCATCGCCCGGCTGCAATCGCTGGGGTTGCCGCTACGCGTGCTGGTG
CTGACCGGCCAGCCGCCTTCGCTGTTCGCCCGCCGCTGCCTGAACTCCGGCGCCGCAGGCTTCGTGTGCAAACACGAGAA
CCTGCACGAGGTCATCAATGCCGCCAAGGCGGTGATGGCCGGCTACACCTACTTCCCCAGCACCACGCTCAGCGAGATGC
GCATGGGCGACAACGCCAAGAGCGACAGTACGCTCATCAGCGTGTTGTCCAACCGCGAACTGACCGTCCTGCAACTGCTG
GCGCAAGGCATGTCCAACAAGGACATCGCTGACAGCATGTTCCTCAGCAACAAGACCGTCAGCACCTACAAGACGCGCCT
GCTGCAGAAGCTGAACGCCACGTCGCTGGTGGAACTGATAGACCTCGCCAAACGCAACAATCTCGCCTAG
>Bpp_12822
ATGTACAACAAAGTCCTCATCATTGACGATCACCCTGTACTGAGATTCGCCGTCCGGGTCCTGATGGAAAAGGAAGGATT
CGAAGTCATCGGCGAAACCGATAACGGTATCGACGGGCTCAAGATCGCCCGCGAGAAAATTCCCAACCTCGTCGTACTCG
ACATCGGCATTCCCAAGCTGGACGGGCTGGAAGTCATCGCCCGGCTGCAATCGCTGGGGTTGCCGCTGCGCGTTCTGGTG
CTGACCGGCCAGCCGCCTTCGCTGTTCGCCCGCCGCTGCCTGAACTCCGGCGCCGCAGGCTTCGTGTGCAAACACGAGAA
CCTGCACGAGGTCATCAATGCCGCCAAGGCGGTGATGGCCGGCTACACCTACTTCCCCAGCACCACGCTCAGCGAGATGC
GCATGGGCGACAACGCCAAGAGCGACAGTACGCTCATCAGCGTGTTGTCCAACCGCGAACTGACCGTCCTACAACTGCTG
GCGCAAGGCATGTCCAACAAGGACATCGCCGACAGCATGTTCCTCAGCAACAAGACCGTCAGCACCTACAAGACGCGCCT
GCTGCAGAAGCTGAACGCCACGTCGCTGGTGGAACTGATAGACCTCGCCAAACGCAACAATCTCGCCTAG
>Bpp_B0024
ATGTACAACAAAGTCCTCATCATTGACGATCACCCTGTACTGAGATTCGCCGTCCGGGTCCTGATGGAAAAGGAAGGATT
CGAAGTCATCGGCGAAACCGATAACGGTATCGACGGGCTCAAGATCGCCCGCGAGAAAATTCCCAACCTCGTCGTACTCG
ACATCGGCATTCCCAAGCTGGACGGGCTGGAAGTCATCGCCCGGCTGCAATCGCTGGGGTTGCCGCTGCGCGTTCTGGTG
CTGACCGGCCAGCCGCCTTCGCTGTTCGCCCGCCGCTGCCTGAACTCCGGCGCCGCAGGCTTCGTGTGCAAACACGAGAA
CCTGCACGAGGTCATCAATGCCGCCAAGGCGGTGATGGCCGGCTACACCTACTTCCCCAGCACCACGCTCAGCGAGATGC
GCATGGGCGACAACGCCAAGAGCGACAGTACGCTCATCAGCGTGTTGTCCAACCGCGAACTGACCGTCCTACAACTGCTG
GCGCAAGGCATGTCCAACAAGGACATCGCCGACAGCATGTTCCTCAGCAACAAGACCGTCAGCACCTACAAGACGCGCCT
GCTGCAGAAGCTGAACGCCACGTCGCTGGTGGAACTGATAGACCTCGCCAAACGCAACAATCTCGCCTAG
>Bpp_B0203
ATGTACAACAAAGTCCTCATCATTGACGATCACCCTGTACTGAGATTCGCCGTCCGGGTCCTGATGGAAAAGGAAGGATT
CGAAGTCATCGGCGAAACCGATAACGGTATCGACGGGCTCAAGATCGCCCGCGAGAAAATTCCCAACCTCGTCGTACTCG
ACATCGGCATTCCCAAGCTGGACGGGCTGGAAGTCATCGCCCGGCTGCAATCGCTGGGGTTGCCGCTGCGCGTTCTGGTG
CTGACCGGCCAGCCGCCTTCGCTGTTCGCCCGCCGCTGCCTGAACTCCGGCGCCGCAGGCTTCGTGTGCAAACACGAGAA
CCTGCACGAGGTCATCAATGCCGCCAAGGCGGTGATGGCCGGCTACACCTACTTCCCCAGCACCACGCTCAGCGAGATGC
GCATGGGCGACAACGCCAAGAGCGACAGTACGCTCATCAGCGTGTTGTCCAACCGCGAACTGACCGTCCTGCAACTGCTG
GCGCAAGGCATGTCCAACAAGGACATCGCCGACAGCATGTTCCTCAGCAACAAGACCGTCAGCACCTACAAGACGCGCCT
GCTGCAGAAGCTGAACGCCACGTCGCTGGTGGAACTGATAGACCTCGCCAAACGCAACAATCTCGCCTAG
>Bpp_B0204
ATGTACAACAAAGTCCTCATCATTGACGATCACCCTGTACTGAGATTCGCCGTCCGGGTCCTGATGGAAAAGGAAGGATT
CGAAGTCATCGGCGAAACCGATAACGGTATTGACGGGCTCAAGATCGCCCGCGAGAAAATTCCCAACCTCGTCGTACTCG
ACATCGGCATTCCCAAGCTGGACGGGCTGGAAGTCATCGCCCGGCTGCAATCGCTGGGGTTGCCGCTGCGCGTTCTGGTG
CTGACCGGCCAGCCGCCTTCGCTGTTCGCCCGCCGCTGCCTGAACTCCGGCGCCGCAGGCTTCGTGTGCAAACACGAGAA
CCTGCACGAGGTCATCAATGCCGCCAAGGCGGTGATGGCCGGCTACACCTACTTCCCCAGCACCACGCTCAGCGAGATGC
GCATGGGCGACAACGCCAAGAGCGACAGTACGCTCATCAGCGTGTTGTCCAACCGCGAACTGACCGTCCTGCAACTGCTG
GCGCAAGGCATGTCCAACAAGGACATCGCCGACAGCATGTTCCTCAGCAACAAGACCGTCAGCACCTACAAGACGCGCCT
GCTGCAGAAGCTGAACGCCACGTCGCTGGTGGAACTGATAGACCTCGCCAAACGCAACAATCTCGCCTAG
>Bpp_B0207
ATGTACAACAAAGTCCTCATCATTGACGATCACCCTGTACTGAGATTCGCCGTCCGGGTCCTGATGGAAAAGGAAGGATT
CGAAGTCATCGGCGAAACCGATAACGGTATTGACGGGCTCAAGATCGCCCGCGAGAAAATTCCCAACCTCGTCGTACTCG
ACATCGGCATTCCCAAGCTGGACGGGCTGGAAGTCATCGCCCGGCTGCAATCGCTGGGGTTGCCGCTGCGCGTTCTGGTG
CTGACCGGCCAGCCGCCTTCGCTGTTCGCCCGCCGCTGCCTGAACTCCGGCGCCGCAGGCTTCGTGTGCAAACACGAGAA
CCTGCACGAGGTCATCAATGCCGCCAAGGCGGTGATGGCCGGCTACACCTACTTCCCCAGCACCACGCTCAGCGAGATGC
GCATGGGCGACAACGCCAAGAGCGACAGTACGCTCATCAGCGTGTTGTCCAACCGCGAACTGACCGTCCTGCAACTGCTG
GCGCAAGGCATGTCCAACAAGGACATCGCCGACAGCATGTTCCTCAGCAACAAGACCGTCAGCACCTACAAGACGCGCCT
GCTGCAGAAGCTGAACGCCACGTCGCTGGTGGAACTGATAGACCTCGCCAAACGCAACAATCTCGCCTAG
>Bpp_B0267
ATGTACAACAAAGTCCTCATCATTGACGATCACCCTGTACTGAGATTCGCCGTCCGGGTCCTGATGGAAAAGGAAGGATT
CGAAGTCATCGGCGAAACCGATAACGGTATCGACGGGCTCAAGATCGCCCGCGAGAAAATTCCCAACCTCGTCGTACTCG
ACATCGGCATTCCCAAGCTGGACGGGCTGGAAGTCATCGCCCGGCTGCAATCGCTGGGGTTGCCGCTACGCGTTCTGGTG
CTGACCGGCCAGCCGCCTTCGCTGTTCGCCCGCCGCTGCCTGAACTCCGGCGCCGCAGGCTTCGTGTGCAAACACGAGAA
CCTGCACGAGGTCATCAATGCCGCCAAGGCGGTGATGGCCGGCTACACCTACTTCCCCAGCACCACGCTCAGCGAGATGC
GCATGGGCGACAACGCCAAGAGCGACAGTACGCTCATCAGCGTGTTGTCCAACCGCGAACTGACCGTCCTGCAACTGCTG
GCGCAAGGCATGTCCAACAAGGACATCGCCGACAGCATGTTCCTCAGCAACAAGACCGTCAGCACCTACAAGACGCGCCT
GCTGCAGAAGCTGAACGCCACGTCGCTGGTGGAACTGATAGACCTCGCCAAACGCAACAATCTCGCCTAG
>Bpp_B0273
ATGTACAACAAAGTCCTCATCATTGACGATCACCCTGTACTGAGATTCGCCGTCCGGGTCCTGATGGAAAAGGAAGGATT
CGAAGTCATCGGCGAAACCGATAACGGTATCGACGGGCTCAAGATCGCCCGCGAGAAAATTCCCAACCTCGTCGTACTCG
ACATCGGCATTCCCAAGCTGGACGGGCTGGAAGTCATCGCCCGGCTGCAATCGCTGGGGTTGCCGCTACGCGTTCTGGTG
CTGACCGGCCAGCCGCCTTCGCTGTTCGCCCGCCGCTGCCTGAACTCCGGCGCCGCAGGCTTCGTGTGCAAACACGAGAA
CCTGCACGAGGTCATCAATGCCGCCAAGGCGGTGATGGCCGGCTACACCTACTTCCCCAGCACCACGCTCAGCGAGATGC
GCATGGGCGACAACGCCAAGAGCGACAGTACGCTCATCAGCGTGTTGTCCAACCGCGAACTGACCGTCCTGCAACTGCTG
GCGCAAGGCATGTCCAACAAGGACATCGCCGACAGCATGTTCCTCAGCAACAAGACCGTCAGCACCTACAAGACGCGCCT
GCTGCAGAAGCTGAACGCCACGTCGCTGGTGGAACTGATAGACCTCGCCAAACGCAACAATCTCGCCTAG
>Bpp_B0285
ATGTACAACAAAGTCCTCATCATTGACGATCACCCTGTACTGAGATTCGCCGTCCGGGTCCTGATGGAAAAGGAAGGATT
CGAAGTCATCGGCGAAACCGATAACGGTATCGACGGGCTCAAGATCGCCCGCGAGAAAATTCCCAACCTCGTCGTACTCG
ACATCGGCATTCCCAAGCTGGACGGGCTGGAAGTCATCGCCCGGCTGCAATCGCTGGGGTTGCCGCTGCGCGTTCTGGTG
CTGACCGGCCAGCCGCCTTCGCTGTTCGCCCGCCGCTGCCTGAACTCCGGCGCCGCAGGCTTCGTGTGCAAACACGAGAA
CCTGCACGAGGTCATCAATGCCGCCAAGGCGGTGATGGCCGGCTACACCTACTTCCCCAGCACCACGCTCAGCGAGATGC
GCATGGGCGACAACGCCAAGAGCGACAGTACGCTCATCAGCGTGTTGTCCAACCGCGAACTGACCGTCCTACAACTGCTG
GCGCAAGGCATGTCCAACAAGGACATCGCCGACAGCATGTTCCTCAGCAACAAGACCGTCAGCACCTACAAGACGCGCCT
GCTGCAGAAGCTGAACGCCACGTCGCTGGTGGAACTGATAGACCTCGCCAAACGCAACAATCTCGCCTAG
>Bpp_B0291
ATGTACAACAAAGTCCTCATCATTGACGATCACCCTGTACTGAGATTCGCCGTCCGGGTCCTGATGGAAAAGGAAGGATT
CGAAGTCATCGGCGAAACCGATAACGGTATCGACGGGCTCAAGATCGCCCGCGAGAAAATTCCCAACCTCGTCGTACTCG
ACATCGGCATTCCCAAGCTGGACGGGCTGGAAGTCATCGCCCGGCTGCAATCGCTGGGGTTGCCGCTGCGCGTTCTGGTG
CTGACCGGCCAGCCGCCTTCGCTGTTCGCCCGCCGCTGCCTGAACTCCGGCGCCGCAGGCTTCGTGTGCAAACACGAGAA
CCTGCACGAGGTCATCAATGCCGCCAAGGCGGTGATGGCCGGCTACACCTACTTCCCCAGCACCACGCTCAGCGAGATGC
GCATGGGCGACAACGCCAAGAGCGACAGTACGCTCATCAGCGTGTTGTCCAACCGCGAACTGACCGTCCTACAACTGCTG
GCGCAAGGCATGTCCAACAAGGACATCGCCGACAGCATGTTCCTCAGCAACAAGACCGTCAGCACCTACAAGACGCGCCT
GCTGCAGAAGCTGAACGCCACGTCGCTGGTGGAACTGATAGACCTCGCCAAACGCAACAATCTCGCCTAG
